# A robust neuromuscular system protects rat and human skeletal muscle from sarcopenia

**DOI:** 10.18632/aging.100926

**Published:** 2016-03-24

**Authors:** Alice Pannérec, Margherita Springer, Eugenia Migliavacca, Alex Ireland, Mathew Piasecki, Sonia Karaz, Guillaume Jacot, Sylviane Métairon, Esther Danenberg, Frédéric Raymond, Patrick Descombes, Jamie S. McPhee, Jerome N. Feige

**Affiliations:** ^1^ Nestlé Institute of Health Sciences, EPFL Innovation Park, 1015 Lausanne, Switzerland; ^2^ School of Healthcare Science, Manchester Metropolitan University, Manchester, UK

**Keywords:** skeletal muscle, neuromuscular junction, sarcopenia, physical frailty

## Abstract

Declining muscle mass and function is one of the main drivers of loss of independence in the elderly. Sarcopenia is associated with numerous cellular and endocrine perturbations, and it remains challenging to identify those changes that play a causal role and could serve as targets for therapeutic intervention. In this study, we uncovered a remarkable differential susceptibility of certain muscles to age-related decline. Aging rats specifically lose muscle mass and function in the hindlimbs, but not in the forelimbs. By performing a comprehensive comparative analysis of these muscles, we demonstrate that regional susceptibility to sarcopenia is dependent on neuromuscular junction fragmentation, loss of motoneuron innervation, and reduced excitability. Remarkably, muscle loss in elderly humans also differs in vastus lateralis and tibialis anterior muscles in direct relation to neuromuscular dysfunction. By comparing gene expression in susceptible and non-susceptible muscles, we identified a specific transcriptomic signature of neuromuscular impairment. Importantly, differential molecular profiling of the associated peripheral nerves revealed fundamental changes in cholesterol biosynthetic pathways. Altogether our results provide compelling evidence that susceptibility to sarcopenia is tightly linked to neuromuscular decline in rats and humans, and identify dysregulation of sterol metabolism in the peripheral nervous system as an early event in this process.

## INTRODUCTION

Skeletal muscle mass and function decline gradually with age and become debilitating in 5-20% of elderly in populations aged over 65 [1-3]. This syndrome is commonly referred to as sarcopenia or physical frailty [4], and represents a major risk factor for falls, loss of independence in activities of daily living, nursing home admissions and, ultimately, mortality [1]. Sarcopenia is caused by multi-factorial endocrine and cellular processes that lead to defects in the contractile function of skeletal muscle. Reduced levels of anabolic hormones, degradation of the muscle contractile protein machinery, loss of regenerative capacity and stem cell function, altered neuronal excitation and mitochondrial dysfunction have all been associated with sarcopenia [5-8]. However the exact causes and consequences of each of these aspects are not fully understood and require further mechanistic investigation at the molecular and cellular level in order to identify which mechanisms can be targeted for intervention.

Altered neuromuscular communication during aging is receiving increasing attention as a potential cause of sarcopenia [8-10]. In mammals, motor innervation of skeletal muscle is propagated via motoneurons which elongate from the spinal cord along peripheral nerves to innervate highly specialized cholinergic synapses termed neuromuscular junctions (NMJs). Defects in the neuromuscular system have been demonstrated during aging in both rodents and humans [11-13]. In particular, loss of motoneurons and peripheral nerve axons as well as post-synaptic fragmentation of NMJs are hallmarks of aging that have been indirectly associated to impaired muscle functionality [11, 14-20]. Recent work has shown that qualitative alterations of the neuromuscular system occur before the loss of muscle mass, and suggests that a decline in motor unit numbers could be one of the early triggers in the pathophysiology of sarcopenia [6, 21, 22]. Therapeutic strategies to protect the neuromuscular system in the context of aging are, however, currently limited as only lifestyle interventions such as caloric restriction or exercise training have been demonstrated to slow-down the decline of NMJs with age [18].

At the molecular level, NMJs are structured via a highly specialized molecular scaffold which enables nicotinic acetylcholine receptors (AchR) to localize specifically to the post-synaptic region [23]. AchRs cluster on post-mitotic endplates in response to motoneuron inputs via a signaling cascade involving binding of neural agrin to the LRP4/MuSK complex. The transcription of genes controlling NMJ structure and function, including AchR subunits and *Musk*, is under tight spatial control through MuSK signaling in order to restrict expression of these genes specifically to the sub-synaptic myonuclei [24-26], while repressing it in extra-synaptic myonuclei [27]. Consequently, one of the molecular manifestations of sarcopenia is a gene signature for functional denervation and neuromuscular dysfunction [8], that most likely follows the de-repression of extra-synaptic NMJ gene transcription. However, whether neuromuscular perturbations are causal in the age-related decline in muscle mass and strength remains unclear [20].

In the present study we report that in rats and humans, age-related neuromuscular decline and the susceptibility to sarcopenia differ between muscles. This regional susceptibility can be used to uncouple sarcopenia from general aging mechanisms and uncover molecular and cellular events in muscle and nerve that are specific to sarcopenia. In particular, we identify cholesterol biosynthetic pathways to be strongly deregulated in nerves innervating sarcopenic muscles.

## RESULTS

### Rat forelimb muscles are resistant to sarcopenia

**Figure 1:**
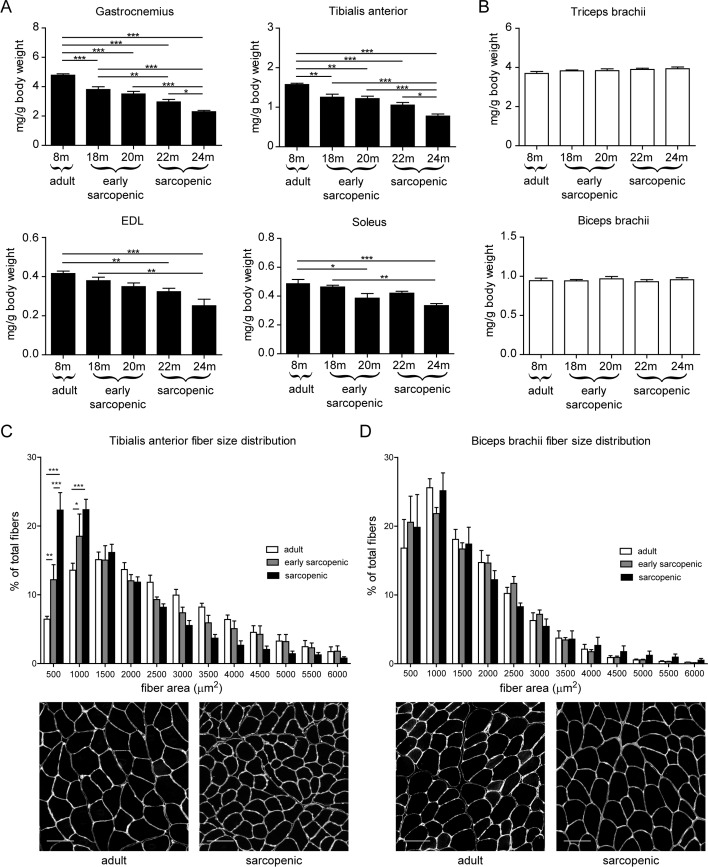
Rat forelimb muscles are resistant to age-related skeletal muscle atrophy (**A-B**) Hindlimb (**A**) or forelimb (**B**) skeletal muscles weight, normalized to body weight, isolated from 8, 18, 20, 22, or 24 month old male wistar rats. (**C-D**) Tibialis anterior (**C**) and biceps brachii (**D**) fiber cross-sectional area distribution measured from a laminin staining. Representative images for adult and sarcopenic conditions are shown. n=9-10 per group for muscle weights, n=4-5 per group for histology. For histology, >1000 fibers per section were analyzed. * p<0.05 ** p<0.01 *** p<0.001 with a one-way ANOVA test. Scale bars represent 100 μm.

In order to characterize how aging differentially affects various locomotor muscles, we performed a cross-sectional analysis of muscle mass in male Wistar rats, where skeletally mature adult controls of 8 months of age were compared to aged animals ranging from 18 months to 24 months. As previously reported [8], hindlimb muscle mass progressively declined with age, reaching approximately 50% atrophy by 24 months of age (Figure 1A). Fast type II myofibers are more susceptible to muscle wasting with age [28, 29]. Consistently, age-dependent muscle atrophy was more pronounced in fast twitch than slow twitch muscles. Nevertheless, all hindlimb muscles analyzed had a similar kinetic of muscle mass decline with age regardless of their fiber type composition and of their contractile involvement in planta- or dorsiflexion, suggesting that a mechanism independent of the contractile phenotype could be an upstream determinant of the susceptibility to age-related muscle atrophy. In contrast, forelimb muscles were protected from age-related muscle atrophy and showed stable muscle mass even at old age (Figure 1B). This striking differential phenotype between hind- and forelimbs suggests that anatomical differences are the primary driver for the susceptibility to sarcopenia. In order to uncover the molecular and cellular mechanisms underlying this differential susceptibility to sarcopenia, we separated the decline in hindlimb muscle mass in 2 phases: an early-sarcopenic phase between 18-20 months of age, where the decline is mild; and a sarcopenic phase between 22-24 months of age where the decline is severe. We then analyzed the fiber size distribution of tibialis anterior (TA) and biceps brachii as prototypical hind- and forelimb muscles, respectively. Fiber cross-sectional area specifically decreased with advancing age in TA while remaining stable in biceps brachii, further demonstrating that forelimb muscles do not undergo aged-related atrophy (Figure 1C-D). Finally, analysis of fiber types revealed that the proportion of slow fibers had slightly increased with age in TA, but not in biceps brachii (Supplementary Figure 1). Cross-sectional area analysis for each fiber type confirmed that fast type IIB fibers were the most prone to age-induced atrophy, and fiber IIB atrophy was only observed in the TA and not the biceps brachii of aged sarcopenic rats (Supplementary Figure 1).

**Figure 2:**
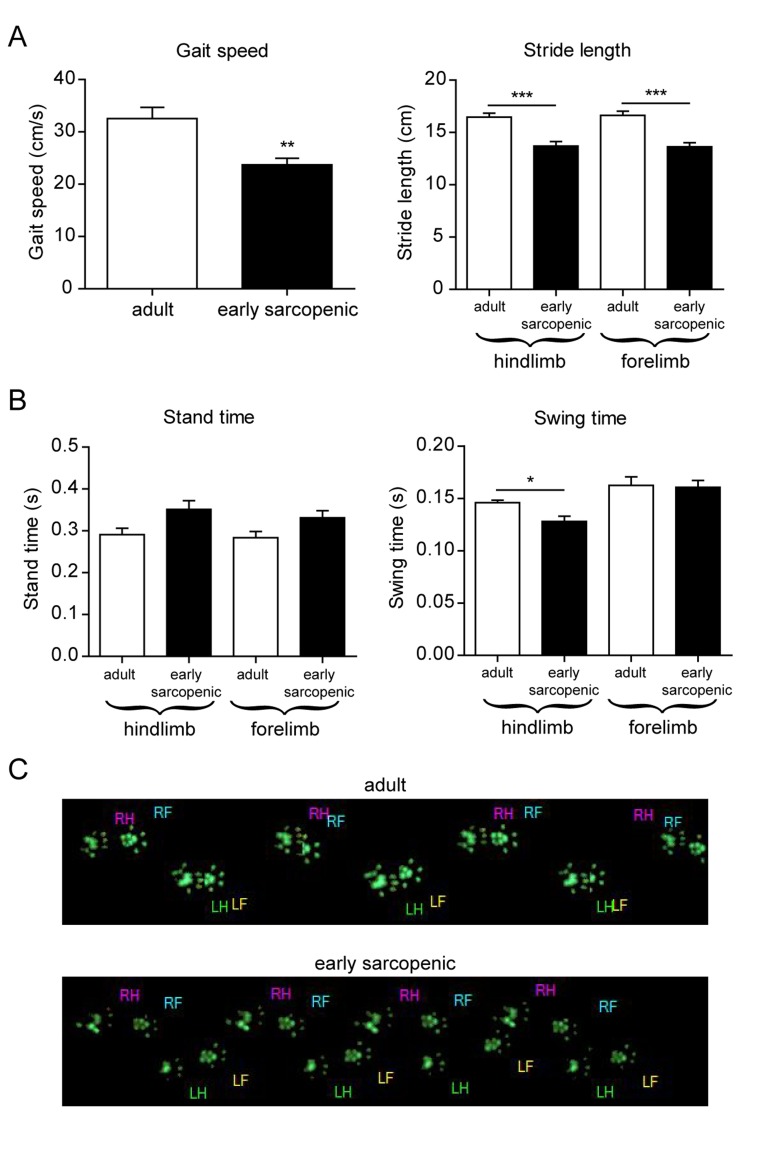
Gait analysis reveals impaired hindlimb function with age Gait parameters were analyzed using a catwalk device in 8-10 months old (adult) and 18-20 months old (early sarcopenic) rats. (**A**) Gait speed and stride length (**B**) Time rats keep their front or hind paws in contact with the surface (stand time), or not in contact with the surface (swing time). (**C**) Representative paw prints from adult and early sarcopenic rats. LH: left hind, RH: right hind, LF: left front, RF: right front. For all graphs n=9-15 per group. * p<0.05 ** p<0.01 *** p<0.001 with a one-way ANOVA test.

Sarcopenia is diagnosed clinically using both muscle mass and function, with gait speed used as one of the clinical measures for function [30]. In order to assess muscle function in the aging rats, we performed a catwalk analysis to evaluate the natural gait of the animals. Gait speed was significantly decreased with age in early sarcopenic rats, due to both a shorter stride length and increased stand time (i.e. the duration of paw contact on the ground) (Figure 2). Differential analysis of hind- and forelimbs further revealed that aged rats have more difficulties to lift their hindlimbs, but not their forelimbs, as indicated by a reduced swing time with age that is specific for hindlimbs (Figure 2B). Muscle function is therefore impaired in aged rats, and differences in the susceptibility to muscle wasting between forelimbs and hindlimbs directly translate into functional locomotor deficits. Altogether, these data demonstrate that rat sarcopenia is highly regional within the body and that the difference in the susceptibility to sarcopenia can be used to uncouple the mechanisms of aging from those of sarcopenia.

**Figure 3:**
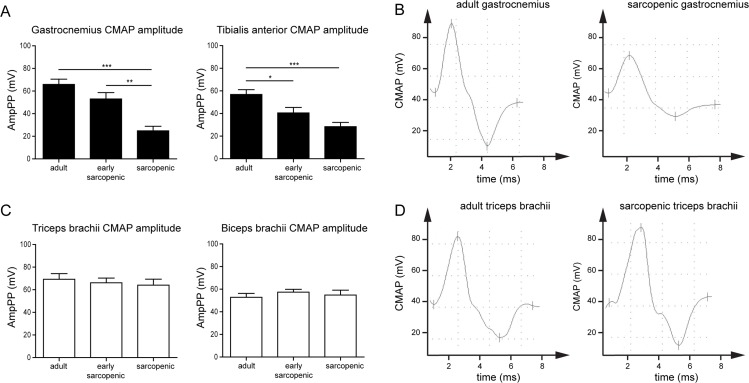
Neuromuscular transmission declines specifically with age in hindlimb but not forelimb muscles in rats Compound muscle action potential (CMAP) was measured by electromyography in hindlimb (**A**) or forelimb (**C**) muscles of adult, early-sarcopenic or sarcopenic rats after stimulation of sciatic (**A**) and radial nerves (**C**). (**B**, **D**) Representative examples of CMAP from adult and sarcopenic gastrocnemius and triceps brachii muscles. For all graphs n=9-12 per group. * p<0.05 ** p<0.01 *** p<0.001 with a one-way ANOVA test.

One major difference between hindlimb and forelimb muscles is that they are innervated by fully distinct sets of nerves, which emanate from different regions of the spinal cord. To test whether differential neuromuscular dysfunction could explain the different susceptibility to sarcopenia, we assessed electromyographic responses in a subset of hindlimb and forelimb muscles from adult, early-sarcopenic and sarcopenic rats. The maximal amplitude of the compound muscle action potential (CMAP) was recorded with needle electrodes inserted in muscles from the hindlimb and forelimb, after stimulating the sciatic and radial nerves, respectively. The maximal CMAP amplitude progressively declined with advancing age in the hindlimb gastrocnemius and TA muscles (Figure 3A-B) but was fully preserved in the forelimb biceps brachii and triceps brachii muscles (Figure 3C-D). These results demonstrate that neuromuscular transmission declines specifically with age in muscles susceptible to sarcopenia, and suggests that a causal relationship may exist between impaired neuromuscular function and muscle wasting during aging.

### Transcriptomic profiling reveals a neuromuscular signature specifically regulated in muscles susceptible to sarcopenia

**Figure 4:**
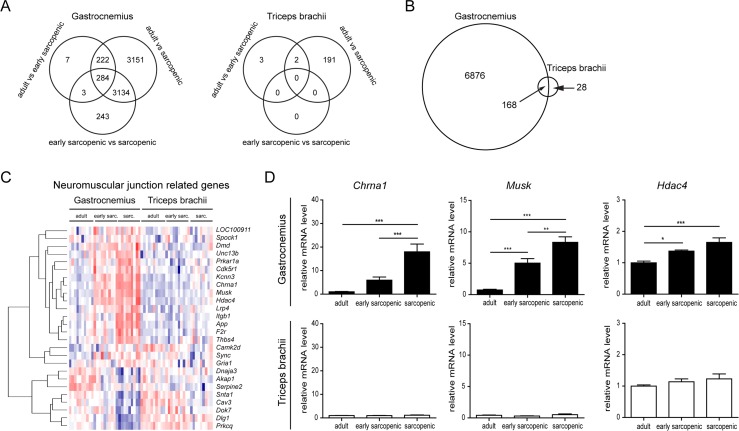
RNA-sequencing reveals specific changes in hindlimb muscles with age RNA-Seq profiling was performed comparing gastrocnemius and triceps brachii from adult, early sarcopenic or sarcopenic rats. (**A**) Venn diagrams of genes significantly regulated between the 3 age groups in gastrocnemius and triceps brachii for the two muscles separately. Genes were considered significantly regulated when the absolute fold change was > 2 and the adjusted p-value < 0.05. (**B**) Venn diagram comparing the overlap of gene regulation with age between gastrocnemius and triceps brachii. Genes were considered significantly regulated when the absolute fold change was > 2 and the adjusted p-value < 0.05. (**C**) Heat map for neuromuscular function related genes isolated from Gene Ontology enrichment analysis. (**D**) Validation of key genes expression by qPCR, using HPRT expression for normalization. For all graphs n=9-10 per group. * p<0.05 ** p<0.01 *** p<0.001 with a one-way ANOVA test.

Given the regional susceptibility to sarcopenia in rats (Figure 1), differential profiling of hindlimb and forelimb muscle gene expression by RNA sequencing during aging provided a way to dissociate the pathways which are specifically regulated during sarcopenia only in hindlimb muscles, from those commonly regulated with age in both hindlimb and forelimb muscles. Many genes were progressively regulated with age in gastrocnemius with more than 3000 genes regulated during early-sarcopenia and more than 6000 genes regulated in sarcopenic hindlimb muscle (Figure 4A). Gene ontology analyses on aged gastrocnemius muscles confirmed that aging alters important biological processes such as neuromuscular function, extra-cellular matrix remodeling & fibrosis, energy homeostasis & mitochondrial function, and inflammation (supplementary table S1), in agreement with previously reported molecular profiling of sarcopenia in the hindlimb [6, 8]. Strikingly, in the triceps brachii muscle, only 196 genes were significantly regulated with age, out of which almost all were regulated at the oldest age (Figure 4B). In addition, nearly all genes for which expression was affected by aging in triceps brachii were also similarly affected by aging in gastrocnemius (Figure 4B). This highly specific molecular signature of aging in gastrocnemius over triceps brachii therefore confirmed that rat sarcopenia is also regional at the molecular level and demonstrated that molecular perturbations in muscle are linked to sarcopenia rather than age (see supplementary table S2 for the full list of genes differentially regulated by age in gastrocnemius and triceps brachii). Gene ontology analysis of the interaction between age and muscle type, which uncouples sarcopenia from aging, demonstrated that sarcopenia specifically alters neuromuscular function, extra-cellular matrix remodeling and energy homeostasis (supplementary table S3). In contrast, genes commonly regulated in both muscles map to inflammation and immune response categories (supplementary table S4), suggesting that at least this subset of chronic inflammatory signaling is not sufficient to drive sarcopenia.

Aging promotes a global up-regulation of many genes controlling NMJ assembly and function in hindlimb muscles, likely through de-repression of extra-synaptic gene inhibition [8]. Given that both neuromuscular decline and altered gene expression correlated with regional muscle wasting, we further analyzed the molecular perturbations in muscle linked to the NMJ. When we focused on genes regulated with age involved in neuromuscular function (Gene Ontology GO:0031594), we could identify 2 major clusters of genes either up- or down-regulated with age specifically in gastrocnemius (Figure 4C). Importantly, none of these genes were regulated with age in triceps brachii in the genome wide profiling and in the follow-up validation by quantitative PCR (Figure 4D), demonstrating that deregulation of neuromuscular genes during aging occurs specifically in the muscle prone to sarcopenia.

### Neuromuscular defects with age are directly associated with the susceptibility to sarcopenia

**Figure 5:**
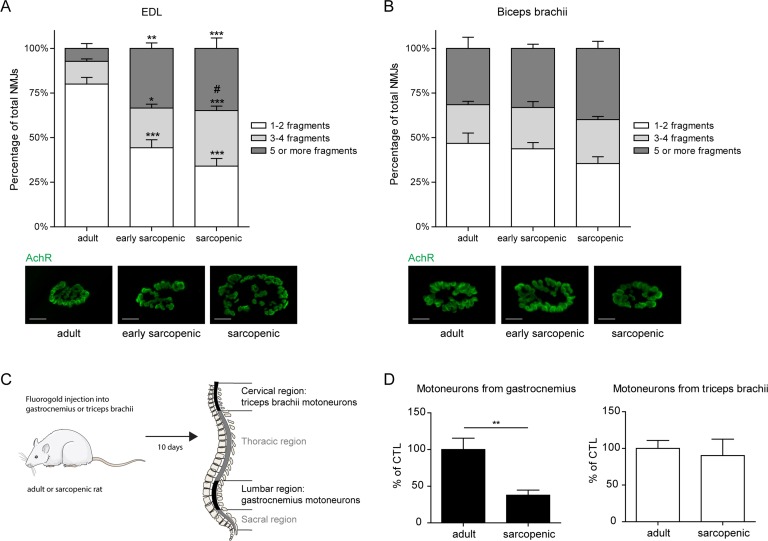
Defects in motor innervation with age are specific to hindlimb muscles (**A**-**B**) Neuromuscular fragmentation was measured on EDL (**A**) and biceps brachii (**B**) from adult, early sarcopenic or sarcopenic rats after staining for acetylcholine receptors (AchR) using fluorescently conjugat α–bungarotoxin. Representative images from each condition are shown. Scale bars represent 20μm. (**C**) Experimental design for retrotracer labeling studies. Motor innervation in gastrocnemius and triceps brachii was evaluated by injecting fluorogold into each muscle and quantifying its spinal cord accumulation 10 days later. (**D**) Number of motoneurons specifically innervating gastrocnemius and triceps brachii in adult and sarcopenic rats. For all graphs n=6-7 per group and an average of 50 NMJs were counted for each sample. * p<0.05 ** p<0.01 *** p<0.001 compared to the same category in adults, and # p<0.05 compared to the same category in early sarcopenic, using a one-way ANOVA test.

The molecular and electrophysiological defects of regional neuromuscular decline between hindlimbs and forelimbs were then investigated at the cellular level. Increased fragmentation of the neuromuscular endplates through dispersion of acetylcholine receptors is believed to be a hallmark of impaired NMJ functionality [31]. NMJ fragmentation in forelimb versus hindlimb muscles was assessed by isolating fiber bundles from biceps brachii and Extensor Digitorum Longus (EDL), respectively, and scoring the level of fragmentation from the images where acetylcholine receptors were stained with fluorescently labeled α-bungarotoxin. The fragmentation level of the hindlimb EDL muscle increased with age from ∼20% in adult muscle to ∼70% in sarcopenic muscle (Figure 5A), consistent with pre-vious reports demonstrating hindlimb NMJ fragmentation in aged rodents [31]. In biceps brachii, however, the proportion of fragmented junctions remained stable at all ages (Figure 5B), despite a much higher baseline fragmentation that likely arises from inherent NMJ architecture in different muscles [32, 33]. In order to confirm that differential fragmentation in hindlimb and forelimb muscles were associated with functional differences during neuromuscular aging, we performed retro-labeling studies by injecting the fluorescent retrotracer fluorogold into the gastrocnemius and the triceps brachii of adult or sarcopenic rats and quantifying labeled motoneurons in the spinal cord (Figure 5C). The number of labeled spinal cord motoneurons projecting to the gastrocnemius hindlimb muscle declined dramatically by more than 50% in sarcopenic vs adult rats, while motoneurons innervating the triceps brachii maintained a normal connectivity with age (Figure 5D). These results demonstrate that both the morphology of NMJs and the connectivity of motoneurons from the spinal cord are specifically altered in sarcopenic hindlimb muscles. Taken together, they further highlight a tight correlation between age-related neuromuscular decline and sarcopenia, which suggests that alterations in the neuromuscular system may be causal in the initiation and progression of sarcopenia.

### Cholesterol biosynthesis declines differentially in nerves according to muscle susceptibility to sarcopenia

Our results demonstrated that defects in the connectivity from the spinal cord to muscle are correlated with impaired neuromuscular transmission and sarcopenia.

**Figure 6:**
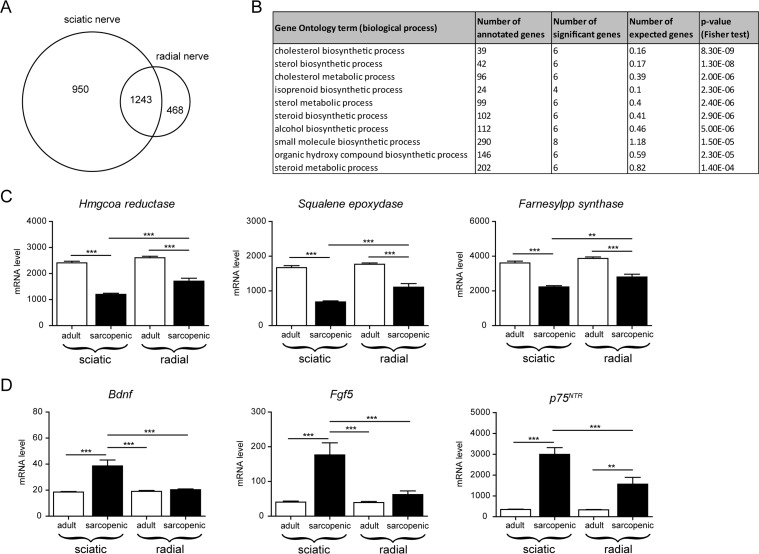
Transcriptional profiling of nerves in aged rats identifies specific molecular signatures associated with the severity of neuromuscular decline (**A**) Venn diagram comparing the overlap of gene regulation with age between sciatic and radial nerves using a linear model. Genes were considered significantly regulated when the absolute fold change was > 2 and the adjusted p-value < 0.05 (FDR = 5%). (**B**) Gene Ontology enrichment analysis of genes responding differently to age in the two nerve types (interaction term in the 2 by 2-factorial design). (**C**) Gene expression level for key enzymes regulating cholesterol synthesis. (**D**) Gene expression level for neutrophic genes: BDNF, FGF5 and the neurotrophin receptor p75^NTR^. For all graphs n=8 per group. * p<0.05 ** p<0.01 *** p<0.001 with a one-way ANOVA test.

To further investigate whether intrinsic molecular defects in nerves could contribute to the susceptibility to sarcopenia, we performed a gene expression analysis comparing hindlimb sciatic nerve to forelimb radial nerve in adult and sarcopenic animals. As shown in Figure 6A, many genes were regulated with age in both nerves, providing a general signature of nerve aging. Gene ontology analysis of genes differentially regulated with age in the 2 nerve types (i.e. the interaction term of the two by two factorial design) converged on many GO terms associated with cholesterol biosynthesis (Figure 6B). Nerve electric signal conduction and velocity are largely dependent on the lipid-rich myelin sheath produced by Schwann cells around the motoneuron axons [34]. Cholesterol is the major constituent of myelin as it accounts for 25% of total myelin lipids, and is essential for proper myelin synthesis and maintenance [35, 36]. Cholesterol is synthesized via a 37-step process regulated by key rate-limiting enzymes such as HMG-CoA reductase, squalene epoxidase and farnesyl-PP-synthase. When looking specifically at the gene expression level of these enzymes, we found a decrease with age in both sciatic and radial nerves, which decrease was significantly stronger in the sciatic nerve (Figure 6C). These data suggest that cholesterol synthesis is impaired with age in correlation with neuromuscular defects, suggesting that defects in cholesterol synthesis could be an early event in the age-related neuromuscular dysfunction. Interestingly, a substantial number of genes showed stronger regulation with age in the sciatic nerve (Table 1), thereby providing potential mechanisms that could drive the differences in neuromuscular severity between hindlimbs and forelimbs. Of particular interest, some neurotrophic factors and regulators of myelination such as Bdnf and Fgf5 were specifically up-regulated with age in the sciatic but not the radial nerve (Figure 6D), most likely because of compensatory mechanisms that are secondary to the severity of nerve alterations. The stronger up-regulation of the neurotrophin receptor p75NTR in aged sciatic nerve further confirmed that nerves afferent to sarcopenic muscles re-activate neurotrophic gene expression programs as p75NTR controls myelination development and is upregulated in demyelinating motoneuron diseases and nerve injury [37].

**Table 1:** List of genes differentially regulated in sciatic and radial nerve with age Genes responding differentially to age in sciatic and radial nerves were filtered based on the interaction term in the 2 by 2-factorial design. Genes were considered significantly regulated when the adjusted p-value < 0.05 (FDR = 5%). Log2 fold change for age, nerve and the interaction age:nerve correspond to the β1, β 2 and β3 coefficients of the 2 by 2 factorial design, respectively.

RefSeq Transcript ID	Gene symbol	Gene name	log2FC Age	log2FC Nerve	log2FC Age:Nerve	adj. p-value Age:Nerve
NM_001107709	*Ap4b1*	adaptor-related protein complex 4, beta 1 subunit	−0.377	−0.352	0.537	3.894E-04
NM_001107839	*Rc3h2*	ring finger and CCCH-type domains 2	−0.507	−0.093	0.572	3.894E-04
NM_001270630	*Bdnf*	brain-derived neurotrophic factor	0.098	−0.033	0.896	6.835E-03
NM_001104613	*Hnrnpa2b1*	heterogeneous nuclear ribonucleoprotein A2/B1	−0.102	−0.541	0.587	1.023E-02
NM_017268	*Hmgcs1*	3-hydroxy-3-methylglutaryl-CoA synthase 1 (soluble)	−0.726	0.004	−0.805	1.023E-02
NM_053539	*Idi1*	isopentenyl-diphosphate delta isomerase 1	−0.853	−0.011	−0.808	1.023E-02
XR_145741	*LOC100909757*	uncharacterized LOC100909757	0.391	0.235	−0.560	1.023E-02
NM_013092	*Cma1*	chymase 1, mast cell	−0.595	0.689	−1.090	1.023E-02
XM_003751331	*LOC685158*	similar to CG8138-PA	0.760	0.128	1.058	1.023E-02
NM_019300	*Cpa3*	carboxypeptidase A3, mast cell	0.100	0.791	−1.170	1.515E-02
NM_053554	*Picalm*	phosphatidylinositol binding clathrin assembly protein	−0.425	−0.196	0.463	1.687E-02
NM_053589	*Rab14*	RAB14, member RAS oncogene family	0.365	0.050	−0.443	1.915E-02
NM_001170487	*Myrf*	myelin regulatory factor	−0.308	−0.192	0.585	1.915E-02
NM_134383	*Elovl6*	ELOVL fatty acid elongase 6	−0.959	−0.059	−0.838	1.915E-02
NM_001257349	*Gng2*	guanine nucleotide binding protein (G protein), gamma 2	−0.739	−0.277	0.853	1.915E-02
NM_012845	*Ms4a2*	membrane-spanning 4-domains, subfamily A, member 2	0.048	0.819	−1.000	1.915E-02
NM_001277668	*Mcpt1l1*	mast cell protease 1-like 1	0.065	0.454	−0.966	2.140E-02
NM_001012738	*Ckmt1b*	creatine kinase, mitochondrial 1B	−0.623	−0.494	−0.811	2.140E-02
NM_017053	*Tacr3*	tachykinin receptor 3	−1.318	−1.472	1.056	2.140E-02
NM_019238	*Fdft1*	farnesyl diphosphate farnesyl transferase 1	−0.772	−0.036	−0.674	2.213E-02
NM_012941	*Cyp51*	cytochrome P450, family 51	−0.620	0.041	−0.604	2.618E-02
NM_001107278	*Fndc3a*	fibronectin type III domain containing 3a	0.448	0.127	−0.435	2.734E-02
NM_020100	*Ramp3*	receptor (G protein-coupled) activity modifying protein 3	1.021	0.130	0.766	3.313E-02
NM_001077356	*Clca4l*	chloride channel calcium activated 4-like	0.646	0.105	−0.578	3.313E-02
NM_053539	*Idi1*	isopentenyl-diphosphate delta isomerase 1	−0.990	−0.129	−0.838	3.356E-02
NM_172023	*Osbpl1a*	oxysterol binding protein-like 1A	−0.369	−0.090	0.350	3.801E-02
NM_012941	*Cyp51*	cytochrome P450, family 51	−0.664	0.027	−0.635	3.801E-02
NM_001025414	*Hars*	histidyl-tRNA synthetase	−0.365	−0.333	0.557	3.801E-02
NM_019180	*Tpsb2*	tryptase beta 2	0.277	0.716	−1.118	3.801E-02
NM_031091	*Rab3b*	RAB3B, member RAS oncogene family	−0.119	0.023	0.958	3.801E-02
NM_022211	*Fgf5*	fibroblast growth factor 5	0.570	0.050	1.403	3.816E-02
NM_001107052	*Arl4d*	ADP-ribosylation factor-like 4D	−1.487	0.223	−0.984	3.866E-02
NM_013034	*Slc6a4*	solute carrier family 6 (neurotransmitter transporter), member 4	−0.030	0.297	−0.849	3.866E-02
XM_001059693	*Tmcc2*	transmembrane and coiled-coil domain family 2	−0.680	−0.620	0.497	4.842E-02
NM_001108202	*Dlk2*	delta-like 2 homolog (Drosophila)	−0.285	−0.165	0.439	4.842E-02
XM_001073064	*LOC686240*	N(alpha)-acetyltransferase 16, NatA auxiliary subunit	0.297	0.086	−0.385	4.855E-02
NM_022618	*Akap6*	A kinase (PRKA) anchor protein 6	−0.154	−0.242	0.506	4.855E-02
NM_001106899	*Plekhb2*	pleckstrin homology domain containing, family B (evectins) member 2	−0.224	−0.147	0.498	4.988E-02

Model: Gene ∼ β0 + β1Age + β2Nerve + β3Age:Nerve

Reference group: adult radial nerve

### Neuromuscular dysfunction correlates with loss of muscle mass in elderly humans

**Figure 7:**
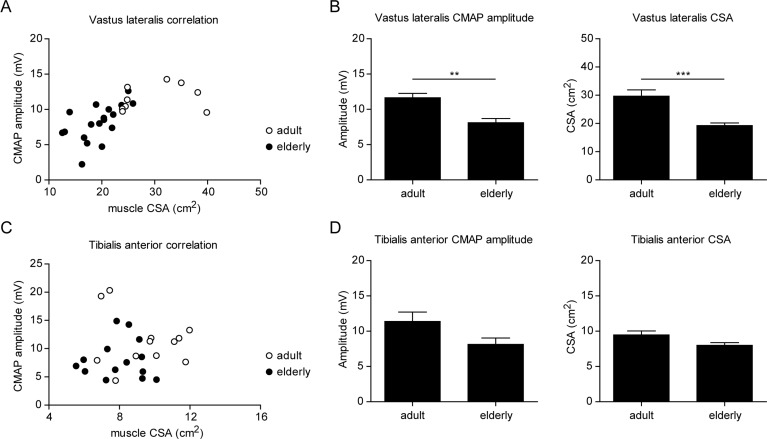
Neuromuscular dysfunction correlates with skeletal muscle atrophy in elderly men (**A**,**C**) Graphs showing the correlation between muscle cross-sectional area (CSA) and CMAP negative peak amplitude in vastus lateralis (**A**) and tibialis anterior (**C**) from adult (i.e 20-35 years) and elderly (i.e >65 years) men. (**B**,**D**) CMAP negative peak amplitude and muscle size in vastus lateralis (**B**) and tibialis anterior (**D**). For all graphs n=9-18 per group. * p<0.05 ** p<0.01 *** p<0.001 with a one-way ANOVA test.

Our results in the rat model of sarcopenia revealed a strong correlation between neuromuscular dysfunction and skeletal muscle atrophy during aging. To test this hypothesis in humans, we evaluated muscle mass and CMAP in the vastus lateralis, a muscle that is particularly susceptible to sarcopenia, and tibialis anterior, which does not normally experience muscle mass decline with advancing age [38]. The decline in muscle size with increasing age in the vastus lateralis (VL) muscle was strongly correlated to decreased CMAP amplitude, demonstrating that both loss of muscle and neuromuscular defects affect this muscle in the elderly (Figure 7A-B). In the tibialis anterior, however, neither muscle size nor CMAP amplitude significantly decreased with increasing age (Figure 7C-D). These results demonstrate that, similar to our observations in the rat, human skeletal muscles with intact neuromuscular transmission are protected from sarcopenia.

## DISCUSSION

Loss of muscle mass and strength is a disabling factor in the aging population for which the prevention and therapeutic options are currently limited. One of the challenges to maintain mobility and physical function in elderly arises from the overlap of a wide range of molecular, cellular, endocrine and nutritional perturbations occurring with age. These multi-factorial contributions to sarcopenia overlap within individual patients, and can be confounded by other manifestations of aging that do not directly play a role in muscle pathophysiology.

Our study establishes a direct and robust association between neuromuscular dysfunction and sarcopenia and characterizes molecular and cellular perturbations of the neuromuscular system that are specifically altered during sarcopenia. In particular, we demonstrate that the susceptibility to sarcopenia differs across different muscles within individuals, both in rats and humans. In humans, the vastus lateralis muscle cross-sectional area was decreased by approximately 40% in the elderly cohort investigated, which is in line with our previous findings in older men [39]. Importantly, the decline in vastus lateralis size in aged individuals directly correlated with the decline in CMAP amplitude measured during electrophysiological assessments. In contrast, the tibialis anterior muscle showed preservation of both muscle cross-sectional area and CMAP amplitude. The CMAP is a clinical assessment of neuromuscular function that is widely used to examine a range of motoneuron and muscle diseases because it indicates the sum of excitable tissue, although it does not necessarily indicate the numbers of motoneurons per se. Interestingly, both the vastus lateralis (VL) and the TA muscles have been shown to lose motor units with advancing old age in humans [40]. Therefore, the relative preservation of muscle mass and CMAP in TA compared with vastus lateralis (VL) suggests that the susceptibility to muscle loss and thus, sarcopenia, can be mediated by local nerve-muscle interactions. The direct correlation between sarcopenia and neuro-muscular dysfunction was also observed in Wistar rats, where muscle mass, gait and neuromuscular function specifically declined in all hindlimb but not forelimb muscles that were analyzed. Interestingly, a similar regional decline in muscle mass has also been reported in humans when considering the entire musculature of the limb, as muscle mass declined faster with age in the legs than in the upper body [41]. Consistent with previous reports in various strains [7, 28], all rat hindlimb muscles including tibialis anterior were affected by sarcopenia, possibly differing from the human phenotype because of rodent-specific posture and locomotion. Altogether, the fact that both rats and humans show regional decline in muscle mass across the different anatomical locations demonstrates that endocrine and systemic factors alone are not the primary mechanism driving sarcopenia. Together with the observation that neuromuscular decline precedes loss of muscle mass [21], our results on the tight association of neuromuscular function and sarcopenia in symptomatic and non-symptomatic muscles of a given individual strongly suggest that neuromuscular dysfunction could be one of the primary drivers of sarcopenia.

The absence of sarcopenia in the forelimb muscles of aged rats also provides a unique model to tease out the molecular and cellular processes that are deregulated during aging without directly contributing to sarcopenia from those that are directly associated to the loss of muscle mass during aging. At the molecular level, inflammatory gene signatures were changed with age in both the muscles prone and resistant to sarcopenia. In contrast, gene networks for neuromuscular function, mitochondria and extra-cellular matrix remodeling and fibrosis were primarily altered with age in the muscle prone to sarcopenia. Experimental models of denervation via nerve resection in young animals have demonstrated that loss of motoneuron innervation impairs mitochondrial function and alters the extra-cellular matrix both at the gene expression and functional level [42-45]. Thus, it is likely that neuromuscular alterations are priming events in the initiation of sarcopenia and lead to downstream perturbations of muscle metabolism and fibrosis by perturbing muscle contractibility. In addition, NMJ fragmentation and motoneuron retro-labeling clearly demonstrated that age specifically affects the neuromuscular system only in the muscles sensitive to sarcopenia, further highlighting that neuromuscular dysfunction is most likely required to initiate the loss of muscle mass.

The effects of age on the neuromuscular system were observed at the level of the NMJ as previously reported [18, 32], but also on the direct connectivity of motoneurons projecting from the spinal cord to muscle fibers. Axons themselves are also affected by aging as axonal diameter increases while myelination is adversely affected in old sciatic nerves [21, 46, 47]. Interestingly, these nerve fiber perturbations during aging seem restricted to nerves innervating hindlimb muscles while sparing nerves innervating the forelimb [47], suggesting that perturbations in the nerve could be one of the upstream determinants of sarcopenia. The priming causes leading to differential functional manifestations of aging in the different nerves still remain unclear. One possibility is that proximal to distal location of motoneuron cellular bodies in the spinal cord could modulate their sensitivity to aging since the sciatic nerve, which is more sensitive to aging, spans from the lumbar region, while the radial nerve spans from the cervical region. Another possibility is that surviving nerves within muscles that are less susceptible to sarcopenia have a higher capacity to perform axonal sprouting and re-innervate fibers in order to compensate for the loss of motoneurons. It is not yet clear which neuromuscular environments might favor reinnervation over loss of fibers, but there is evidence of such muscle-specific responses in humans. For instance, motoneuron loss in the vastus lateralis (VL) [40] is accompanied by extensive fiber loss [29], while motoneuron loss of in human TA is almost completely compensated by fiber reinnervation resulting in fewer, but considerably larger, motor units [48] and no loss of muscle mass (Fig 7).

At the molecular level, we identified transcriptional signatures of nerve aging that are specific to the sciatic over the radial nerve. Gene expression analysis has previously suggested a role for lipid metabolism and inflammation as mechanisms of nerve aging [46]. Our analyses of sciatic and radial nerves unequivocally identified cholesterol biosynthesis as the key pathway differentially regulated in the sciatic nerves innervating the sarcopenia-prone leg muscles. All rate limiting enzymes of cholesterol biosynthesis were down-regulated with age with a larger amplitude in the sciatic than the radial nerve. Cholesterol is the major lipid constituent of myelin and genetic deletion of cholesterol biosynthetic enzymes or cholesterol sensors leads to strong hypo-myelination phenotypes and locomotor deficits [49, 50]. In addition, statins, which are widely used clinically to mitigate cardiovascular risks by reducing cholesterol levels through HMG-CoA reductase inhibition, have been proposed to increase the risk for peripheral neuropathy [51], on top of their well-known side effects on muscle [52]. Down-regulation of cholesterol biosynthesis in aged nerves therefore likely alters myelination to induce neuromuscular dysfunction and sarcopenia. The gene signature specifically regulated with age in the sciatic nerve was also enriched in genes coding for secreted factors such as BDNF and FGF5, as well as the neutrophin receptor p75NTR, raising the possibility that neurotrophic paracrine signaling could also contribute. Given that these factors have previously been shown to protect from neuro-muscular dysfunction or to be induced upon denervation [37, 53-55], it is, however, more likely that these molecular perturbations are compensatory adaptations secondary to the effects that drive neuromuscular aging. Given that compensatory mechanisms at the gene expression level often indicate functional deficiencies, these observations could highlight local loss of neurotrophic input via either lower growth factor secretion or intra-cellular growth factor resistance.

Altogether, our study brings comprehensive evidence that the severity of sarcopenia is tightly linked to neuromuscular dysfunction in rats and humans, with direct evidence in both species that susceptibility to sarcopenia is strongly influenced by local rather than systemic cues. Electrophysiological measurements, NMJ morphology, and molecular profiling all converge to demonstrate that neuromuscular input plays a key role in the susceptibility to sarcopenia, and that altered cholesterol metabolism in aged nerves is a mechanism upstream to sarcopenia.

## METHODS

### Animal experiments

All experimental procedures on animals were in agreement with Swiss and EU ethical guidelines and approved by the local animal experimentation committee of the Canton of Vaud (license number VD2630). Male Wistar rats aged 8 months to 24 months were obtained from Janvier Labs (Le Genest-Saint-Isle, France). Upon arrival all animals were housed by two in standard type 4 cages with *ad libitum* access to food and water on a 12 hour light/dark cycle at a temperature between 20-24°C and a relative humidity between 50-60%. All rats labeled by age were grouped by date of birth within one month, and further grouping was then based on the muscle phenotype in hindlimbs under the following categories: adult (8-10 months of age), early-sarcopenic (18-20 months of age), and sarcopenic (22-24 months of age). Rats were sacrificed by exsanguination under isoflurane anesthesia and skeletal muscles were dissected free of fat, weighed and snap frozen in liquid nitrogen or processed for histology as described below. A piece of about 1cm of sciatic and radial nerves was dissected out from the mid-thigh or arm region and snap frozen in liquid nitrogen.

Electromyography (EMG) measurements were performed on rats under isoflurane anesthesia. The left limbs were shaved and recording needle electrodes (twisted pairs, wire 150cm, needle 0.4 × 13mm, Neurolite, Switzerland) were sequentially placed into the gastrocnemius, tibialis anterior, triceps brachii and biceps brachii muscles. Supra-maximal electric stimulation was achieved via stimulating needle electrodes sequentially placed around the sciatic nerve and the radial nerve, and the resulting compound muscle action potential (CMAP) was recorded using the Keypoint software (Neurolite, Switzerland).

Gait parameters were measured using the Catwalk XT system (Noldus, Netherlands). Animals were placed to spontaneously walk on a glass lane with tangential illumination and each step was recorded by detecting light diffraction using a camera below the lane. At least 3 runs per animal were acquired with 10 minutes break in between. Locomotion patterns and gait speed were then analyzed from smooth runs, which were defined as runs during which the rat maintained constant speed for at least 3-4 prints per paw, using the automated Catwalk analysis software. All runs were required, manually edited for correct detection of the paws.

### Immunofluorescence staining and microscopy

For fiber type analysis, tibialis anterior and biceps brachii were frozen in isopentane cooled in liquid nitrogen, stored at −80°C and cryo-sectioned at 10μm. Sections were incubated 2h in blocking solution made with 4% IgG-free bovine serum albumin (001-000-162, Jackson immunoresearch) + 1% fetal bovine serum in PBS and subsequently incubated with mouse MHC2B (clone BFF3, DSHB) and rabbit laminin (L9393, Sigma aldrich) antibodies overnight. After washing, sections were incubated with donkey anti-mouse-IgM-Cy3 (715-165-020, Jackson immunoresearch) + goat anti-rabbit-A405 (A-31556, Thermo Fisher) secondary antibodies for 1h. A second round of incubations were then performed using MHC2A (clone SC71, DSHB) + MHC1 (clone BAD5, DSHB), and then goat anti-mouse-IgG1-A647 (A-21240, Thermo Fisher) + goat anti-mouse-IgG2b-A488 (A-21141, Thermo Fisher). Images were acquired using a slide scanner (VS-120, Olympus) and analyzed using an in-house MetaXpress journal (Molecular Devices, Sunnyvale, USA). Briefly, fibers were segmented based on laminin staining using a combination of morphological operators and thresholding. Segmentation results were manually controlled and corrected prior to create the final segmentation masks and extract individual fiber cross-sectional area. Intensity thresholds for fiber type staining were manually chosen for each image individually before final quantification. Fibers where the average intensity in the channel of interest was above the threshold were annotated positive for this specific marker. Fibers that were negative for MHC1, 2A and 2B were considered positive for MHC2X. Subsequent data processing was performed by a custom workflow using KNIME Analytic Platform (http://www.knime.org) [56].

For neuromuscular junction analyses, muscles were pinned on cork in PBS and injected with α-bungarotoxin-A488 (1:5000, B-13422 Thermo Fischer) for 30min to stain for acetylcholine receptors. Muscles were then rinsed with PBS and injected with 2% paraformaldehyde solution (P6148, Sigma Aldrich) for 15min, rinsed again and stored at 4°C. Muscles were then separated into bundles of 20-30 fibers, mounted with fluorescence mounting medium (S302380, Dako) and pressed overnight at 4°C. Images were acquired on a Leica DMI 6000B microscope. For each muscle, approximately 50 neuromuscular junctions were imaged and each junction was classified into 3 classes (1-2 fragments, 3-4 fragments or ≥5 fragments) according to the number of fragments counted manually.

### Motoneuron retrotracing study

Rats were anesthesized using isoflurane and the right legs were shaved and disinfected with ethanol. Fluorogold (Fluorochrome LLC) was diluted at 2% (w/v) and injected into the gastrocnemius and triceps brachii at 4 to 6 points of injection. The total volume of injection was 60-80 μL per muscle. Ten days later, animals were sacrificed by intracardial perfusion of paraformaldehyde and the spinal cord was dissected out, placed in paraformaldehyde solution overnight at 4°C and subsequently placed in 20% sucrose solution for 3-4 days. The cervical and lumbar regions of each spinal cord were then removed and frozen in OCT embedding medium (361603E, VWR Chemicals). Each sample was cryo-sectioned at 40μm on its entire length and 1 out of 4 sections were examined to count the number of labeled motoneurons.

### Gene expression profiling

#### Muscle RNA-Seq analysis

Total RNA was extracted using the miRNeasy Mini Kit (Qiagen) according to the manufacturer's instruction. RNA quantity was measured with Ribogreen (Life Technologies) and RNA quality was assessed on a Bioanalyzer (Agilent Technologies). Sequencing libraries were prepared from 250ng total RNA using the TruSeq RNA Library Preparation Kit v2 following the manufacturer's protocol. The procedure was automated on a Sciclone NGS Workstation (Perkin Elmer). Purified libraries were quantified with Picogreen (Life Technologies) and the size pattern was controlled with the DNA High Sensitivity Reagent kit on a LabChip GX (Perkin Elmer). Libraries were then pooled and clustered at a concentration of 8pmol on a v3 paired-end sequencing flow cell (Illumina). Sequencing was performed for 2 × 100 cycles on a HiSeq 2000 strictly following Illumina's recommendations. Fastq files were mapped to the Rattus Norvegicus genome Rnor_6 with the alignment software RNAStar (version 2.3.0) using the following parameters: outFilterMultimapNmax = 3, outFilter MismatchNoverLmax = 0.3, outFilterMulti mapScore Range = 1. Mapped sequences with mapping scores lower than 30 (samtools-0.1.19 with option -q 30) were filtered out from further analysis, and mapped sequences that passed QC criteria were exported as one bam file per sample. The number of reads mapped within genes was quantified by HTSeq (option union, version HTSeq-0.5.4p3). Genes with very low counts (less than 20 reads in each sample) were discarded. The gene counts were normalized using edgeR TMM (trimmed mean of M values) prior to voom (variance modelling at the observational level) analysis in Limma [57, 58]. We provided an estimate of the within-animal correlation via the duplicate Correlation function available in Limma since we had two muscles from each animal [59]. The voom method was used to estimate the mean-variance relationship of log2 counts per million (log-cpm) for each sample. These estimates were taken into consideration by the empirical Bayes Limma models. We analyzed the data set as a two (muscle types: triceps brachii and gastrocnemius) by three (age categories: 8, 18, and 24 months) factorial design. Raw data are available in GEO (http://www.ncbi.nlm.nih.gov/geo/) under accession number GSE78702.

#### Nerve micro-array analysis

Total RNA was extracted using the miRNeasy Mini Kit (Qiagen) according to the manufacturer's instruction. The quality of RNA samples was checked using the Standard Sensitivity RNA Analysis Kit on a Fragment Analyzer (Advanced Analytical Technologies). All cRNA targets were synthesized using the Illumina TotalPrep-96 RNA amplification Kit (Life Technologies) and fragmented according to the Affymetrix protocol, based on the Eberwine T7 procedure. Briefly, 200ng of total RNA were used to produce double-stranded cDNA, followed by in vitro transcription, and cRNA labeling with biotin. Then, 7.5μg of cRNA were hybridized on Affymetrix Rat 230 PM 96-Array in an Affymetrix GeneTitan device (Affymetrix, CA, USA) directly followed by staining and scanning in the same instrument. Affymetrix probes were normalized and summarized to probe sets using the Robust Multi-array Average (RMA) approach. We applied a non-specific filter to discard probe sets with low variability and retained 15549 probe sets whose SD was greater than median of SD of all probe sets. Genes (represented by 15549 probe sets) were tested for differential expression using Limma for age, nerve type, and interaction term between age and nerve type [57]. We took into account that for each animal we had two nerve types by providing an estimate of the within-animal correlation via the duplicate Correlation function available in Limma [59]. We defined probe sets whose Benjamini-Hoechberd adjusted p-value was lower than 0.05 as differentially expressed and exploited topGO to assess whether the DE probe sets were related to specific pathways. Raw data are available in GEO (http://www.ncbi.nlm.nih.gov/geo/) under accession number GSE77022.

### Reverse transcription and qPCR

Total RNA was extracted using a commercial kit (miRNeasy Mini Kit, Qiagen) according to the manufacturer's instruction, and the concentration was measured using a NanoDrop device. Total RNA extracts were diluted at 20ng/ml and reverse transcribed into cDNA using the High Capacity cDNA Reverse Transcription Kit (Applied Biosystems) and then diluted 1/2. Quantitative PCR reactions were performed using LightCycler DNA Green Master (Roche) on a LightCycler 384 Real-Time PCR System thermocycler (Roche). The amplification curves were analyzed by the LightCycler 480 SW 1.5 software. HPRT was amplified as qPCR normalization gene.

### Human clinical study

The human clinical study was approved by the University of Manchester Research Ethics Committee and was conducted in accordance with the *Declaration of Helsinki.* All participants provided written informed consent prior to inclusion in the study. Volunteers were included if they were male, and for the vastus lateralis aged between 18-35 years (mean (SD) age 25.2 (4.6) years, height 1.77 (0.06) m, body mass 74.8 (11.1) kg and BMI 23.7 (2.9) kg/m^2^) or 65-90 years (mean (SD) age 71.6 (6.2) years, height 1.72 (0.07) m, body mass 73.2 (8.7) kg and BMI 24.6 (2.7) kg/m^2^). For the tibialis anterior (TA) they were aged between 18-35 years (mean (SD) age 27.5 (44.6) years, height 1.77 (0.05) m, body mass 76.7 (10.9) kg and BMI 24.5 (3.7) kg/m^2^) or 65-90 years (mean (SD) age 74.8 (7.6) years, height 1.71 (0.06) m, body mass 74.7 (7.6) kg and BMI 25.9 (4.02) kg/m^2^). All participants were physically active and living independently. They were free from neuromuscular, metabolic and cardiovascular diseases (except for controlled hypertension), had not suffered a broken bone within the past two years and reported no mobility problems that affected their ability to complete usual activities of daily living.

Muscle anatomical cross sectional area. Magnetic resonance imaging (MRI) was used to measure peak VL and TA muscle anatomical cross sectional areas using a T1-weighted turbo 3D sequence on a 0.25-T G-Scan (Esaote, Genoe, Italy). The motor point over the VL muscle belly was marked, and contiguous transverse-plane slices of 6mm thickness were collected. Images were analyzed off-line using Osirix imaging software (OsiriX medical imaging, OsiriX, Atlanta, USA) and the cross-sectional at the motor point was recorded.

### Surface EMG

The maximal compound muscle action potential (CMAP) was evoked by a manually triggered stimulator (model DS7A; Digitimer, Welwyn Garden City, Hertfordshire, UK) using percutaneous stimulation (Medserve, Daventry, UK). For VL, stimulation was applied to the femoral nerve and a carbon-rubber anode electrode (Dermatrode self-adhering electrode, 5.08cm diameter, Farmadomo, NL) was placed over the skin overlying the gluteus muscle. For TA, the anode was placed on the medial knee joint cleft and stimulation was applied to a superficial part of the common peroneal nerve on the lateral aspect of the shank close to the head of the fibula. For each muscle, the recording surface-EMG electrode was positioned over the motor point of the respective muscle belly to ensure the largest CMAP (negative peak amplitude) with fastest rise-time. For both VL and TA, the stimulator voltage was fixed at 400V and the pulse width at 50μS, and the current was then increased incrementally until the CMAP negative peak amplitude plateaued. At this point the current was increased again by 30mA to ensure supra-maximal stimulation (usually occurring between 100 and 200mA for VL and 80-120mA for TA).

### Statistics

All genome wide statistical analyses were performed using R version 3.1.3 and relevant Bioconductor packages as described in the gene expression profiling section. All other statistical analyses were performed using the GraphPad Prism software, and statistical significance was assessed by the Mann-Whitney test for binary comparisons. For comparison of more than 2 groups, one-way ANOVA followed by Bonferroni's multiple comparison test was used. All data are expressed as mean value +/− s.e.m.

## SUPPLEMENTARY DATA FIGURE AND TABLES










