# Supplementary material for: A robust neuromuscular system protects rat and human skeletal muscle from sarcopenia
Source: Aging (Albany NY). 2016 Mar 24;8(4):712–28. doi: 10.18632/aging.100926 (PMC4925824; doi:10.18632/aging.100926)
Supplement: Supplementary file 1 [file aging-08-712-s001.pdf]

SUPPLEMENTARY DATA

Please browse Full text version to see Supplementary Tables 1-4 of this manuscript.

Supplementary Table 1. Gene Ontology (GO) of genes regulated with age in the sarcopenic gastrocnemius muscle by RNAseq.

Supplementary Table 2. List of genes differentially regulated with age in Gastrocnemius and Triceps brachii using a 2 by 3 by factorial analysis of muscle RNAseq. (Log2FC: Log2 fold change).

Supplementary Table 3. Gene Ontology (GO) of genes differentially regulated with age in Gastrocnemius and Triceps brachii using a 2 by 3 by factorial analysis of muscle RNAseq.

Supplementary Table 4. Gene Ontology (GO) of genes commonly regulated with age in Gastrocnemius and Triceps brachii using a 2 by 3 by factorial analysis of muscle RNAseq.

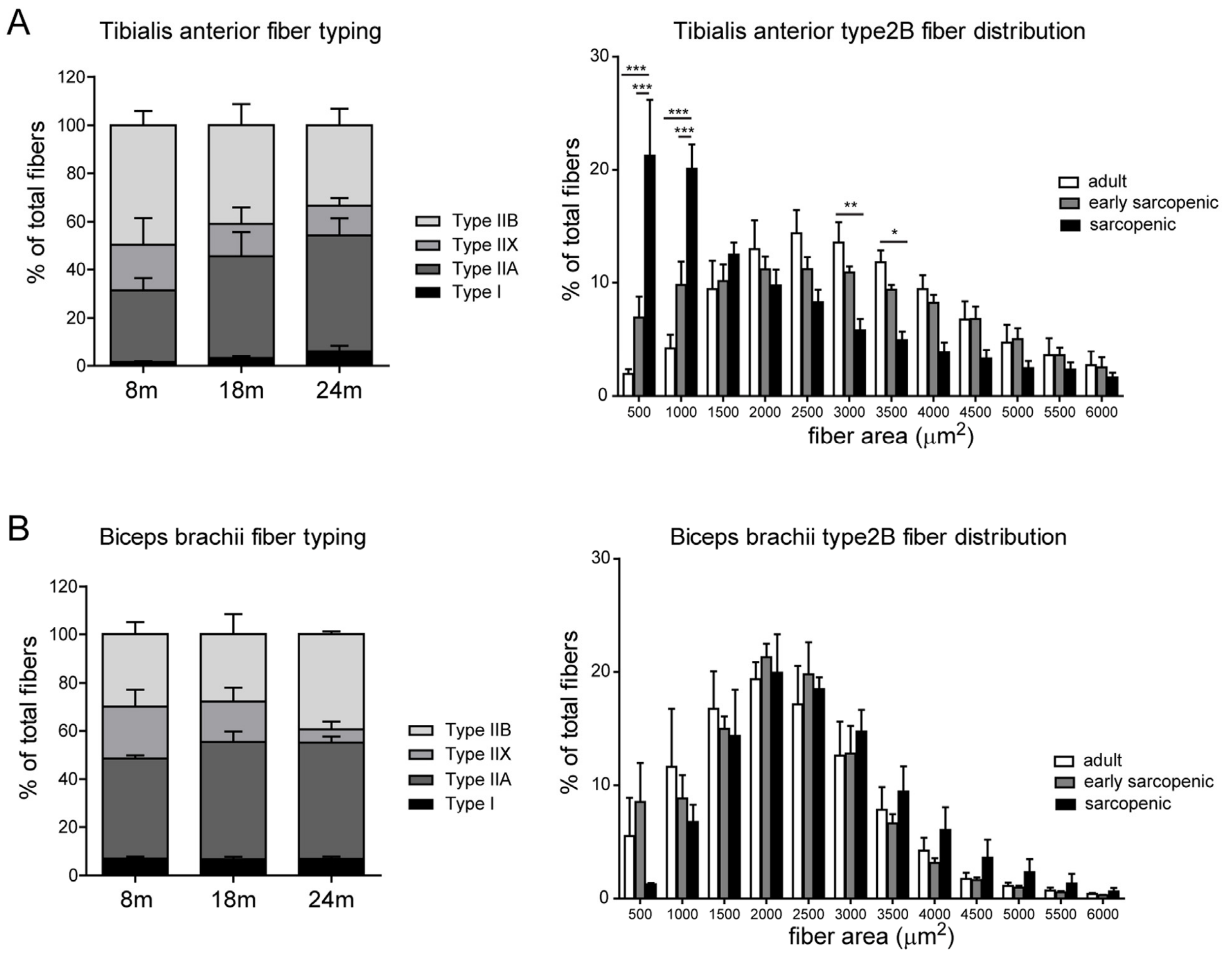

Supplementary Figure 1. Fiber type composition and fiber type IIB cross-sectional area in aged rats. Tibialis anterior (**A**) and biceps brachii (**B**) fiber type composition and type IIB fiber cross-sectional area distribution. *n=4-5 per group, >1000 fibers per section were analyzed. * p<0.05 ** p<0.01 *** p<0.001 with a two-way ANOVA test.*
