# Supplementary material for: A robust neuromuscular system protects rat and human skeletal muscle from sarcopenia
Source: Aging (Albany NY). 2016 Mar 24;8(4):712–28. doi: 10.18632/aging.100926 (PMC4925824; doi:10.18632/aging.100926)
Supplement: Supplementary file 2 [file aging-08-712-s002.pdf]

Gene Ontology ID	Gene Ontology term (Biological Process)	Number of annotated genes	Number of significant genes	Number of expected genes	p-value (Fisher test)
GO:0006955	immune response	590	224	121.25	7.70E-24
GO:0002376	immune system process	1200	376	246.61	3.30E-21
GO:0044699	single-organism process	7904	1774	1624.35	1.30E-20
GO:0002684	positive regulation of immune system pro...	394	156	80.97	8.80E-19
GO:0050778	positive regulation of immune response	248	110	50.97	9.90E-18
GO:0002682	regulation of immune system process	634	219	130.29	1.60E-17
GO:0050776	regulation of immune response	337	136	69.26	2.60E-17
GO:0009605	response to external stimulus	1216	364	249.9	7.20E-17
GO:0045321	leukocyte activation	437	160	89.81	1.60E-15
GO:0042110	T cell activation	253	107	51.99	1.60E-15
GO:0002252	immune effector process	328	129	67.41	2.00E-15
GO:0001775	cell activation	519	182	106.66	2.20E-15
GO:0006952	defense response	609	205	125.16	4.40E-15
GO:0002250	adaptive immune response	164	78	33.7	5.90E-15
GO:0050900	leukocyte migration	156	73	32.06	1.20E-13
GO:0002460	adaptive immune response based on somati...	153	72	31.44	1.30E-13
GO:0046649	lymphocyte activation	367	135	75.42	2.00E-13
GO:0006811	ion transport	837	255	172.01	8.80E-13
GO:0002253	activation of immune response	176	77	36.17	2.00E-12
GO:0046651	lymphocyte proliferation	161	72	33.09	2.90E-12
GO:0032943	mononuclear cell proliferation	162	72	33.29	4.20E-12
GO:0044763	single-organism cellular process	7050	1574	1448.84	5.30E-12
GO:0042098	T cell proliferation	117	57	24.04	7.90E-12
GO:0070661	leukocyte proliferation	167	73	34.32	8.10E-12
GO:0001816	cytokine production	350	125	71.93	1.70E-11
GO:0006812	cation transport	627	197	128.85	2.40E-11
GO:0044707	single-multicellular organism process	3634	876	746.82	2.80E-11
GO:0042221	response to chemical	2104	543	432.39	3.00E-11
GO:0002443	leukocyte mediated immunity	178	75	36.58	3.70E-11
GO:0030001	metal ion transport	451	151	92.68	3.70E-11
GO:0032501	multicellular organismal process	3754	899	771.48	6.40E-11
GO:0030595	leukocyte chemotaxis	104	51	21.37	7.30E-11
GO:0070887	cellular response to chemical stimulus	1351	369	277.64	8.60E-11
GO:0045087	innate immune response	267	100	54.87	8.90E-11
GO:0050670	regulation of lymphocyte proliferation	126	58	25.89	9.40E-11
GO:0002449	lymphocyte mediated immunity	142	63	29.18	1.00E-10
GO:0097530	granulocyte migration	62	36	12.74	1.00E-10

GO:0002456	T cell mediated immunity	65	37	13.36	1.20E-10
GO:0048002	antigen processing and presentation of p...	52	32	10.69	1.30E-10
GO:0032944	regulation of mononuclear cell prolifera...	127	58	26.1	1.40E-10
GO:0043207	response to external biotic stimulus	461	151	94.74	2.30E-10
GO:0051707	response to other organism	461	151	94.74	2.30E-10
GO:0042129	regulation of T cell proliferation	92	46	18.91	2.70E-10
GO:0097529	myeloid leukocyte migration	89	45	18.29	2.70E-10
GO:0070663	regulation of leukocyte proliferation	129	58	26.51	3.00E-10
GO:0019882	antigen processing and presentation	81	42	16.65	3.80E-10
GO:0001817	regulation of cytokine production	302	108	62.06	3.80E-10
GO:0034220	ion transmembrane transport	488	157	100.29	4.40E-10
GO:0071621	granulocyte chemotaxis	59	34	12.13	4.40E-10
GO:1990266	neutrophil migration	49	30	10.07	6.10E-10
GO:0002819	regulation of adaptive immune response	100	48	20.55	6.70E-10
GO:0009607	response to biotic stimulus	477	153	98.03	9.50E-10
GO:0050896	response to stimulus	4607	1067	946.78	1.70E-09
GO:0030593	neutrophil chemotaxis	48	29	9.86	1.80E-09
GO:0002764	immune response-regulating signaling pat...	158	65	32.47	2.40E-09
GO:0050863	regulation of T cell activation	155	64	31.85	2.70E-09
GO:0002757	immune response-activating signal transd...	146	61	30	3.70E-09
GO:0048878	chemical homeostasis	593	180	121.87	3.80E-09
GO:0002822	regulation of adaptive immune response b...	95	45	19.52	3.90E-09
GO:0060326	cell chemotaxis	140	59	28.77	4.50E-09
GO:0002821	positive regulation of adaptive immune r...	63	34	12.95	4.60E-09
GO:0034097	response to cytokine	409	133	84.05	4.80E-09
GO:0098660	inorganic ion transmembrane transport	343	115	70.49	8.00E-09
GO:0006954	inflammatory response	328	111	67.41	8.10E-09
GO:0072676	lymphocyte migration	35	23	7.19	8.80E-09
GO:0002521	leukocyte differentiation	303	104	62.27	1.00E-08
GO:0002694	regulation of leukocyte activation	259	92	53.23	1.10E-08
GO:0002824	positive regulation of adaptive immune r...	62	33	12.74	1.20E-08
GO:0002697	regulation of immune effector process	174	68	35.76	1.30E-08
GO:0050865	regulation of cell activation	282	98	57.95	1.40E-08
GO:0048584	positive regulation of response to stimu...	1028	282	211.26	1.50E-08

GO:0002703	regulation of leukocyte mediated immunit...	108	48	22.2	1.60E-08
GO:0051249	regulation of lymphocyte activation	222	81	45.62	2.20E-08
GO:0051239	regulation of multicellular organismal p...	1570	405	322.65	2.60E-08
GO:0098655	cation transmembrane transport	365	119	75.01	2.70E-08
GO:0032879	regulation of localization	1325	349	272.3	3.00E-08
GO:0010033	response to organic substance	1643	421	337.65	3.10E-08
GO:0009611	response to wounding	484	149	99.47	3.40E-08
GO:2000106	regulation of leukocyte apoptotic proces...	59	31	12.13	5.10E-08
GO:0050801	ion homeostasis	404	128	83.03	5.30E-08
GO:0071310	cellular response to organic substance	1085	292	222.98	5.90E-08
GO:0072678	T cell migration	21	16	4.32	6.80E-08
GO:0032844	regulation of homeostatic process	253	88	51.99	7.20E-08
GO:0006091	generation of precursor metabolites and ...	202	74	41.51	7.30E-08
GO:0051049	regulation of transport	950	260	195.23	7.30E-08
GO:0002706	regulation of lymphocyte mediated immuni...	90	41	18.5	7.60E-08
GO:0050870	positive regulation of T cell activation	106	46	21.78	7.70E-08
GO:0030217	T cell differentiation	136	55	27.95	8.20E-08
GO:0002705	positive regulation of leukocyte mediate...	63	32	12.95	8.80E-08
GO:0055085	transmembrane transport	679	195	139.54	9.60E-08
GO:0070228	regulation of lymphocyte apoptotic proce...	41	24	8.43	1.10E-07
GO:0042592	homeostatic process	924	253	189.89	1.10E-07
GO:0002709	regulation of T cell mediated immunity	52	28	10.69	1.10E-07
GO:0031347	regulation of defense response	278	94	57.13	1.20E-07
GO:0051240	positive regulation of multicellular org...	430	133	88.37	1.40E-07
GO:0046631	alpha-beta T cell activation	76	36	15.62	1.40E-07
GO:0006950	response to stress	1987	493	408.35	1.50E-07
GO:0030098	lymphocyte differentiation	191	70	39.25	1.60E-07
GO:0032101	regulation of response to external stimu...	368	117	75.63	1.60E-07
GO:0055082	cellular chemical homeostasis	384	121	78.92	1.70E-07
GO:0002683	negative regulation of immune system pro...	167	63	34.32	2.00E-07
GO:0098662	inorganic cation transmembrane transport	304	100	62.47	2.20E-07

GO:0042102	positive regulation of T cell proliferat...	62	31	12.74	2.20E-07
GO:0001819	positive regulation of cytokine producti...	171	64	35.14	2.30E-07
GO:0043269	regulation of ion transport	335	108	68.85	2.30E-07
GO:0007584	response to nutrient	189	69	38.84	2.30E-07
GO:0002711	positive regulation of T cell mediated i...	45	25	9.25	2.40E-07
GO:0009617	response to bacterium	324	105	66.59	2.50E-07
GO:0002685	regulation of leukocyte migration	84	38	17.26	2.80E-07
GO:0098542	defense response to other organism	176	65	36.17	3.20E-07
GO:0002699	positive regulation of immune effector p...	108	45	22.2	4.40E-07
GO:0002708	positive regulation of lymphocyte mediat...	61	30	12.54	5.50E-07
GO:0022610	biological adhesion	628	179	129.06	5.80E-07
GO:0046633	alpha-beta T cell proliferation	21	15	4.32	7.20E-07
GO:0071345	cellular response to cytokine stimulus	315	101	64.74	7.40E-07
GO:1901700	response to oxygen-containing compound	1026	272	210.85	7.40E-07
GO:2000107	negative regulation of leukocyte apoptot...	36	21	7.4	7.50E-07
GO:0007159	leukocyte cell-cell adhesion	36	21	7.4	7.50E-07
GO:0001910	regulation of leukocyte mediated cytotox...	50	26	10.28	7.90E-07
GO:0002696	positive regulation of leukocyte activat...	162	60	33.29	8.10E-07
GO:0006090	pyruvate metabolic process	62	30	12.74	8.60E-07
GO:0001913	T cell mediated cytotoxicity	39	22	8.01	9.10E-07
GO:0006875	cellular metal ion homeostasis	278	91	57.13	9.70E-07
GO:0007155	cell adhesion	621	176	127.62	1.10E-06
GO:0002474	antigen processing and presentation of p...	34	20	6.99	1.10E-06
GO:0070838	divalent metal ion transport	233	79	47.88	1.10E-06
GO:0006935	chemotaxis	291	94	59.8	1.20E-06
GO:0055080	cation homeostasis	357	111	73.37	1.20E-06
GO:0050867	positive regulation of cell activation	171	62	35.14	1.20E-06
GO:0050671	positive regulation of lymphocyte prolif...	85	37	17.47	1.30E-06
GO:0001909	leukocyte mediated cytotoxicity	63	30	12.95	1.30E-06
GO:0032609	interferon-gamma production	63	30	12.95	1.30E-06
GO:0031341	regulation of cell killing	54	27	11.1	1.30E-06
GO:0072511	divalent inorganic cation transport	238	80	48.91	1.40E-06
GO:0048534	hematopoietic or lymphoid organ developm...	532	154	109.33	1.40E-06
GO:0042330	taxis	292	94	60.01	1.50E-06

GO:0044765	single-organism transport	2107	511	433.01	1.80E-06
GO:0071887	leukocyte apoptotic process	70	32	14.39	1.80E-06
GO:0002367	cytokine production involved in immune r...	43	23	8.84	1.80E-06
GO:0032946	positive regulation of mononuclear cell ...	86	37	17.67	1.80E-06
GO:0030003	cellular cation homeostasis	301	96	61.86	1.80E-06
GO:0072503	cellular divalent inorganic cation homeo...	232	78	47.68	1.80E-06
GO:0030335	positive regulation of cell migration	247	82	50.76	1.80E-06
GO:0045088	regulation of innate immune response	127	49	26.1	2.10E-06
GO:0032649	regulation of interferon-gamma productio...	58	28	11.92	2.10E-06
GO:0002687	positive regulation of leukocyte migrati...	61	29	12.54	2.10E-06
GO:0055114	oxidation-reduction process	632	177	129.88	2.30E-06
GO:0040017	positive regulation of locomotion	260	85	53.43	2.30E-06
GO:0006873	cellular ion homeostasis	307	97	63.09	2.50E-06
GO:0055065	metal ion homeostasis	323	101	66.38	2.70E-06
GO:0030097	hemopoiesis	509	147	104.6	2.80E-06
GO:0001912	positive regulation of leukocyte mediate...	47	24	9.66	3.20E-06
GO:0046640	regulation of alpha-beta T cell prolifer...	18	13	3.7	3.40E-06
GO:0006941	striated muscle contraction	98	40	20.14	3.60E-06
GO:0070665	positive regulation of leukocyte prolife...	88	37	18.08	3.60E-06
GO:0051251	positive regulation of lymphocyte activa...	147	54	30.21	3.80E-06
GO:0006816	calcium ion transport	217	73	44.6	3.80E-06
GO:0001914	regulation of T cell mediated cytotoxici...	36	20	7.4	3.90E-06
GO:2000147	positive regulation of cell motility	252	82	51.79	4.30E-06
GO:2000403	positive regulation of lymphocyte migrat...	16	12	3.29	4.40E-06
GO:0030334	regulation of cell migration	419	124	86.11	4.70E-06
GO:0009615	response to virus	148	54	30.42	4.80E-06
GO:0072507	divalent inorganic cation homeostasis	245	80	50.35	4.90E-06
GO:0030155	regulation of cell adhesion	249	81	51.17	5.00E-06
GO:0002690	positive regulation of leukocyte chemota...	45	23	9.25	5.00E-06
GO:0031343	positive regulation of cell killing	51	25	10.48	5.20E-06
GO:0098602	single organism cell adhesion	269	86	55.28	5.60E-06
GO:0050921	positive regulation of chemotaxis	73	32	15	5.60E-06

GO:0002520	immune system development	562	158	115.5	6.50E-06
GO:0019725	cellular homeostasis	451	131	92.68	7.20E-06
GO:0070227	lymphocyte apoptotic process	52	25	10.69	8.10E-06
GO:0044259	multicellular organismal macromolecule m...	52	25	10.69	8.10E-06
GO:0046634	regulation of alpha-beta T cell activati...	49	24	10.07	8.20E-06
GO:0070482	response to oxygen levels	291	91	59.8	8.30E-06
GO:0051272	positive regulation of cellular componen...	260	83	53.43	8.60E-06
GO:0002495	antigen processing and presentation of p...	19	13	3.9	8.70E-06
GO:0002504	antigen processing and presentation of p...	19	13	3.9	8.70E-06
GO:0051704	multi-organism process	1034	267	212.5	9.20E-06
GO:2000401	regulation of lymphocyte migration	24	15	4.93	9.20E-06
GO:0002274	myeloid leukocyte activation	105	41	21.58	1.00E-05
GO:0016337	single organismal cell-cell adhesion	230	75	47.27	1.00E-05
GO:0051238	sequestering of metal ion	75	32	15.41	1.10E-05
GO:0001916	positive regulation of T cell mediated c...	35	19	7.19	1.10E-05
GO:0002704	negative regulation of leukocyte mediate...	17	12	3.49	1.20E-05
GO:0001906	cell killing	72	31	14.8	1.20E-05
GO:0003008	system process	854	225	175.5	1.20E-05
GO:0050777	negative regulation of immune response	50	24	10.28	1.30E-05
GO:0032963	collagen metabolic process	50	24	10.28	1.30E-05
GO:0051283	negative regulation of sequestering of c...	69	30	14.18	1.30E-05
GO:0051209	release of sequestered calcium ion into ...	69	30	14.18	1.30E-05
GO:0002768	immune response-regulating cell surface ...	96	38	19.73	1.40E-05
GO:0002429	immune response-activating cell surface ...	86	35	17.67	1.50E-05
GO:0007600	sensory perception	217	71	44.6	1.50E-05
GO:0002369	T cell cytokine production	15	11	3.08	1.60E-05
GO:0030574	collagen catabolic process	15	11	3.08	1.60E-05
GO:0036293	response to decreased oxygen levels	260	82	53.43	1.60E-05
GO:0002218	activation of innate immune response	73	31	15	1.70E-05
GO:0044236	multicellular organismal metabolic proce...	60	27	12.33	1.70E-05
GO:0048513	organ development	2062	493	423.76	1.70E-05
GO:0045089	positive regulation of innate immune res...	97	38	19.93	1.90E-05
GO:0051282	regulation of sequestering of calcium io...	70	30	14.39	1.90E-05
GO:0002455	humoral immune response mediated by circ...	25	15	5.14	1.90E-05

GO:0002688	regulation of leukocyte chemotaxis	54	25	11.1	1.90E-05
GO:0071219	cellular response to molecule of bacteri...	118	44	24.25	1.90E-05
GO:0002478	antigen processing and presentation of e...	20	13	4.11	2.00E-05
GO:0046641	positive regulation of alpha-beta T cell...	13	10	2.67	2.00E-05
GO:2000406	positive regulation of T cell migration	13	10	2.67	2.00E-05
GO:0008016	regulation of heart contraction	101	39	20.76	2.20E-05
GO:0051270	regulation of cellular component movemen...	490	138	100.7	2.30E-05
GO:0032729	positive regulation of interferon-gamma ...	28	16	5.75	2.30E-05
GO:1903034	regulation of response to wounding	235	75	48.29	2.30E-05
GO:0003015	heart process	119	44	24.46	2.40E-05
GO:0060047	heart contraction	119	44	24.46	2.40E-05
GO:0001666	response to hypoxia	255	80	52.4	2.50E-05
GO:0051208	sequestering of calcium ion	71	30	14.59	2.60E-05
GO:2000021	regulation of ion homeostasis	123	45	25.28	2.70E-05
GO:0042493	response to drug	392	114	80.56	2.70E-05
GO:0007166	cell surface receptor signaling pathway	1541	377	316.69	2.80E-05
GO:0070229	negative regulation of lymphocyte apopto...	23	14	4.73	2.80E-05
GO:0032611	interleukin-1 beta production	34	18	6.99	2.90E-05
GO:0048518	positive regulation of biological proces...	2990	690	614.47	2.90E-05
GO:2000145	regulation of cell motility	438	125	90.01	2.90E-05
GO:0002718	regulation of cytokine production involv...	34	18	6.99	2.90E-05
GO:0006096	glycolytic process	46	22	9.45	3.10E-05
GO:0031349	positive regulation of defense response	142	50	29.18	3.20E-05
GO:0050672	negative regulation of lymphocyte prolif...	40	20	8.22	3.20E-05
GO:0032945	negative regulation of mononuclear cell ...	40	20	8.22	3.20E-05
GO:0015980	energy derivation by oxidation of organi...	146	51	30	3.50E-05
GO:0071222	cellular response to lipopolysaccharide	110	41	22.61	3.70E-05
GO:0031667	response to nutrient levels	350	103	71.93	3.80E-05
GO:0002237	response to molecule of bacterial origin	250	78	51.38	3.90E-05
GO:0006874	cellular calcium ion homeostasis	219	70	45.01	4.10E-05
GO:0002707	negative regulation of lymphocyte mediat...	16	11	3.29	4.20E-05

GO:0044243	multicellular organismal catabolic proce...	16	11	3.29	4.20E-05
GO:0055074	calcium ion homeostasis	227	72	46.65	4.20E-05
GO:2000404	regulation of T cell migration	16	11	3.29	4.20E-05
GO:0050850	positive regulation of calcium-mediated ...	21	13	4.32	4.30E-05
GO:0033993	response to lipid	653	175	134.2	4.40E-05
GO:0032663	regulation of interleukin-2 production	32	17	6.58	4.60E-05
GO:0050920	regulation of chemotaxis	97	37	19.93	4.90E-05
GO:0046635	positive regulation of alpha-beta T cell...	38	19	7.81	5.00E-05
GO:0070664	negative regulation of leukocyte prolife...	41	20	8.43	5.00E-05
GO:0006928	cellular component movement	986	251	202.63	5.10E-05
GO:0070232	regulation of T cell apoptotic process	24	14	4.93	5.50E-05
GO:0002695	negative regulation of leukocyte activat...	87	34	17.88	5.50E-05
GO:0019886	antigen processing and presentation of e...	14	10	2.88	5.80E-05
GO:0051179	localization	3260	744	669.96	5.90E-05
GO:0009991	response to extracellular stimulus	370	107	76.04	6.00E-05
GO:0002440	production of molecular mediator of immu...	91	35	18.7	6.30E-05
GO:0016477	cell migration	713	188	146.53	6.30E-05
GO:0002698	negative regulation of immune effector p...	51	23	10.48	6.60E-05
GO:0032103	positive regulation of response to exter...	138	48	28.36	6.60E-05
GO:0048583	regulation of response to stimulus	2056	486	422.53	7.10E-05
GO:0002437	inflammatory response to antigenic stimu...	33	17	6.78	7.60E-05
GO:0007186	G-protein coupled receptor signaling pat...	364	105	74.81	7.90E-05
GO:0051607	defense response to virus	92	35	18.91	8.20E-05
GO:0034765	regulation of ion transmembrane transpor...	173	57	35.55	8.20E-05
GO:0051674	localization of cell	760	198	156.19	8.40E-05
GO:0048870	cell motility	760	198	156.19	8.40E-05
GO:1901701	cellular response to oxygen-containing c...	584	157	120.02	9.20E-05
GO:0050663	cytokine secretion	96	36	19.73	9.20E-05
GO:0031348	negative regulation of defense response	82	32	16.85	9.30E-05
GO:0050707	regulation of cytokine secretion	82	32	16.85	9.30E-05
GO:0014070	response to organic cyclic compound	683	180	140.36	9.30E-05

GO:0051234	establishment of localization	2620	606	538.43	9.40E-05
GO:0015672	monovalent inorganic cation transport	280	84	57.54	9.50E-05
GO:0002224	toll-like receptor signaling pathway	49	22	10.07	1.00E-04
GO:0019884	antigen processing and presentation of e...	25	14	5.14	1.00E-04
GO:0050727	regulation of inflammatory response	159	53	32.68	1.00E-04
GO:0006810	transport	2535	587	520.97	1.10E-04
GO:0050866	negative regulation of cell activation	93	35	19.11	1.10E-04
GO:0032612	interleukin-1 production	37	18	7.6	1.20E-04
GO:0019221	cytokine-mediated signaling pathway	175	57	35.96	1.20E-04
GO:0051250	negative regulation of lymphocyte activa...	76	30	15.62	1.20E-04
GO:0040012	regulation of locomotion	476	131	97.82	1.20E-04
GO:0042108	positive regulation of cytokine biosynth...	37	18	7.6	1.20E-04
GO:0022407	regulation of cell-cell adhesion	76	30	15.62	1.20E-04
GO:0002673	regulation of acute inflammatory respons...	37	18	7.6	1.20E-04
GO:0002758	innate immune response-activating signal...	66	27	13.56	1.30E-04
GO:0051480	cytosolic calcium ion homeostasis	164	54	33.7	1.30E-04
GO:0033630	positive regulation of cell adhesion med...	15	10	3.08	1.40E-04
GO:0002526	acute inflammatory response	80	31	16.44	1.40E-04
GO:0016064	immunoglobulin mediated immune response	70	28	14.39	1.50E-04
GO:0071216	cellular response to biotic stimulus	131	45	26.92	1.50E-04
GO:0006099	tricarboxylic acid cycle	23	13	4.73	1.60E-04
GO:0006959	humoral immune response	60	25	12.33	1.60E-04
GO:0060402	calcium ion transport into cytosol	84	32	17.26	1.60E-04
GO:0007568	aging	256	77	52.61	1.70E-04
GO:0002366	leukocyte activation involved in immune ...	113	40	23.22	1.70E-04
GO:0002263	cell activation involved in immune respo...	113	40	23.22	1.70E-04
GO:0002700	regulation of production of molecular me...	57	24	11.71	1.70E-04
GO:0071396	cellular response to lipid	285	84	58.57	1.80E-04
GO:0050851	antigen receptor-mediated signaling path...	74	29	15.21	1.80E-04
GO:0033077	T cell differentiation in thymus	54	23	11.1	1.90E-04
GO:0032623	interleukin-2 production	38	18	7.81	1.90E-04
GO:0045058	T cell selection	29	15	5.96	1.90E-04

GO:0019724	B cell mediated immunity	71	28	14.59	2.00E-04
GO:0009306	protein secretion	159	52	32.68	2.00E-04
GO:0042130	negative regulation of T cell proliferat...	32	16	6.58	2.00E-04
GO:0048520	positive regulation of behavior	85	32	17.47	2.00E-04
GO:0060401	cytosolic calcium ion transport	85	32	17.47	2.00E-04
GO:0002027	regulation of heart rate	51	22	10.48	2.10E-04
GO:0006979	response to oxidative stress	307	89	63.09	2.20E-04
GO:0048871	multicellular organismal homeostasis	175	56	35.96	2.20E-04
GO:0007154	cell communication	3249	735	667.7	2.30E-04
GO:0034762	regulation of transmembrane transport	183	58	37.61	2.30E-04
GO:1903035	negative regulation of response to wound...	68	27	13.97	2.30E-04
GO:0007275	multicellular organismal development	2952	672	606.66	2.40E-04
GO:0033273	response to vitamin	93	34	19.11	2.50E-04
GO:0002221	pattern recognition receptor signaling p...	65	26	13.36	2.60E-04
GO:0034341	response to interferon-gamma	55	23	11.3	2.70E-04
GO:0040011	locomotion	881	222	181.05	2.70E-04
GO:0002701	negative regulation of production of mol...	11	8	2.26	2.80E-04
GO:0045076	regulation of interleukin-2 biosynthetic...	11	8	2.26	2.80E-04
GO:0023052	signaling	3188	721	655.16	2.90E-04
GO:0044700	single organism signaling	3188	721	655.16	2.90E-04
GO:0032496	response to lipopolysaccharide	236	71	48.5	2.90E-04
GO:0045333	cellular respiration	83	31	17.06	3.00E-04
GO:0032651	regulation of interleukin-1 beta product...	27	14	5.55	3.00E-04
GO:0000302	response to reactive oxygen species	169	54	34.73	3.00E-04
GO:0071804	cellular potassium ion transport	83	31	17.06	3.00E-04
GO:0071805	potassium ion transmembrane transport	83	31	17.06	3.00E-04
GO:0002902	regulation of B cell apoptotic process	16	10	3.29	3.10E-04
GO:0048247	lymphocyte chemotaxis	16	10	3.29	3.10E-04
GO:0005996	monosaccharide metabolic process	173	55	35.55	3.10E-04
GO:0048731	system development	2703	618	555.49	3.10E-04
GO:0048771	tissue remodeling	113	39	23.22	3.70E-04
GO:0050795	regulation of behavior	128	43	26.31	3.70E-04
GO:0071706	tumor necrosis factor superfamily cytoki...	63	25	12.95	3.90E-04
GO:0032602	chemokine production	43	19	8.84	3.90E-04

GO:0050864	regulation of B cell activation	77	29	15.82	4.00E-04
GO:0097285	cell-type specific apoptotic process	341	96	70.08	4.00E-04
GO:0030278	regulation of ossification	136	45	27.95	4.00E-04
GO:0051050	positive regulation of transport	489	131	100.49	4.10E-04
GO:0046916	cellular transition metal ion homeostasi...	53	22	10.89	4.10E-04
GO:0016052	carbohydrate catabolic process	88	32	18.08	4.20E-04
GO:0001818	negative regulation of cytokine producti...	88	32	18.08	4.20E-04
GO:0032642	regulation of chemokine production	40	18	8.22	4.20E-04
GO:0050708	regulation of protein secretion	125	42	25.69	4.30E-04
GO:0035710	CD4-positive, alpha-beta T cell activati...	37	17	7.6	4.50E-04
GO:1902622	regulation of neutrophil migration	22	12	4.52	4.50E-04
GO:0051279	regulation of release of sequestered cal...	50	21	10.28	4.60E-04
GO:0042035	regulation of cytokine biosynthetic proc...	50	21	10.28	4.60E-04
GO:0051046	regulation of secretion	359	100	73.78	4.70E-04
GO:0070233	negative regulation of T cell apoptotic ...	14	9	2.88	4.70E-04
GO:0031294	lymphocyte costimulation	14	9	2.88	4.70E-04
GO:0031295	T cell costimulation	14	9	2.88	4.70E-04
GO:0050798	activated T cell proliferation	25	13	5.14	4.80E-04
GO:0045123	cellular extravasation	28	14	5.75	4.90E-04
GO:0001503	ossification	293	84	60.21	4.90E-04
GO:0050730	regulation of peptidyl-tyrosine phosphor...	145	47	29.8	5.00E-04
GO:0006813	potassium ion transport	111	38	22.81	5.20E-04
GO:0007204	positive regulation of cytosolic calcium...	149	48	30.62	5.20E-04
GO:0051716	cellular response to stimulus	3680	821	756.27	5.30E-04
GO:0080134	regulation of response to stress	622	161	127.83	5.40E-04
GO:0019318	hexose metabolic process	161	51	33.09	5.50E-04
GO:0009628	response to abiotic stimulus	798	201	164	5.50E-04
GO:0045785	positive regulation of cell adhesion	134	44	27.54	5.50E-04
GO:0050868	negative regulation of T cell activation	54	22	11.1	5.60E-04
GO:0008283	cell proliferation	1170	284	240.45	5.60E-04
GO:0046903	secretion	566	148	116.32	5.70E-04
GO:0032680	regulation of tumor necrosis factor prod...	61	24	12.54	5.90E-04
GO:0007165	signal transduction	2889	654	593.72	6.00E-04

GO:0050702	interleukin-1 beta secretion	17	10	3.49	6.10E-04
GO:0071624	positive regulation of granulocyte chemo...	17	10	3.49	6.10E-04
GO:0090023	positive regulation of neutrophil chemot...	17	10	3.49	6.10E-04
GO:1902624	positive regulation of neutrophil migrat...	17	10	3.49	6.10E-04
GO:0044724	single-organism carbohydrate catabolic p...	86	31	17.67	6.20E-04
GO:0097305	response to alcohol	316	89	64.94	6.40E-04
GO:0006936	muscle contraction	170	53	34.94	6.60E-04
GO:0042113	B cell activation	143	46	29.39	6.90E-04
GO:0032102	negative regulation of response to exter...	120	40	24.66	6.90E-04
GO:0009060	aerobic respiration	35	16	7.19	7.00E-04
GO:0042554	superoxide anion generation	20	11	4.11	7.10E-04
GO:0046632	alpha-beta T cell differentiation	55	22	11.3	7.50E-04
GO:0032652	regulation of interleukin-1 production	29	14	5.96	7.70E-04
GO:0051048	negative regulation of secretion	113	38	23.22	7.70E-04
GO:0032640	tumor necrosis factor production	62	24	12.74	7.70E-04
GO:0042742	defense response to bacterium	87	31	17.88	7.70E-04
GO:0031960	response to corticosteroid	175	54	35.96	7.70E-04
GO:0032613	interleukin-10 production	29	14	5.96	7.70E-04
GO:0030199	collagen fibril organization	29	14	5.96	7.70E-04
GO:0045667	regulation of osteoblast differentiation	87	31	17.88	7.70E-04
GO:0060537	muscle tissue development	297	84	61.04	7.80E-04
GO:0032502	developmental process	3447	770	708.39	7.90E-04
GO:0018108	peptidyl-tyrosine phosphorylation	232	68	47.68	8.60E-04
GO:0071407	cellular response to organic cyclic comp...	269	77	55.28	8.70E-04
GO:0042089	cytokine biosynthetic process	59	23	12.13	8.80E-04
GO:0051384	response to glucocorticoid	164	51	33.7	8.80E-04
GO:0010043	response to zinc ion	39	17	8.01	9.40E-04
GO:0007605	sensory perception of sound	70	26	14.39	9.90E-04
GO:0000041	transition metal ion transport	56	22	11.51	1.00E-03
GO:0002719	negative regulation of cytokine producti...	10	7	2.06	1.02E-03
GO:0071361	cellular response to ethanol	10	7	2.06	1.02E-03
GO:0050877	neurological system process	500	131	102.75	1.07E-03
GO:0065008	regulation of biological quality	1960	453	402.8	1.09E-03
GO:0009719	response to endogenous stimulus	1037	252	213.11	1.09E-03

GO:0018212	peptidyl-tyrosine modification	234	68	48.09	1.10E-03
GO:0050715	positive regulation of cytokine secretio...	53	21	10.89	1.14E-03
GO:0001776	leukocyte homeostasis	60	23	12.33	1.16E-03
GO:0050728	negative regulation of inflammatory resp...	60	23	12.33	1.16E-03
GO:0030154	cell differentiation	2209	506	453.97	1.17E-03
GO:0055076	transition metal ion homeostasis	78	28	16.03	1.19E-03
GO:0010324	membrane invagination	27	13	5.55	1.22E-03
GO:0045061	thymic T cell selection	21	11	4.32	1.22E-03
GO:0034121	regulation of toll-like receptor signali...	27	13	5.55	1.22E-03
GO:0045471	response to ethanol	127	41	26.1	1.22E-03
GO:0071622	regulation of granulocyte chemotaxis	27	13	5.55	1.22E-03
GO:0090022	regulation of neutrophil chemotaxis	21	11	4.32	1.22E-03
GO:0070296	sarcoplasmic reticulum calcium ion trans...	24	12	4.93	1.24E-03
GO:0045937	positive regulation of phosphate metabol...	576	148	118.37	1.25E-03
GO:0010562	positive regulation of phosphorus metabo...	576	148	118.37	1.25E-03
GO:1990267	response to transition metal nanoparticl...	93	32	19.11	1.26E-03
GO:0042100	B cell proliferation	50	20	10.28	1.29E-03
GO:0048856	anatomical structure development	3156	706	648.59	1.31E-03
GO:0071346	cellular response to interferon-gamma	40	17	8.22	1.33E-03
GO:0071453	cellular response to oxygen levels	97	33	19.93	1.33E-03
GO:0044767	single-organism developmental process	3429	763	704.69	1.37E-03
GO:0001932	regulation of protein phosphorylation	661	167	135.84	1.40E-03
GO:0002573	myeloid leukocyte differentiation	132	42	27.13	1.47E-03
GO:0002820	negative regulation of adaptive immune r...	13	8	2.67	1.49E-03
GO:0034123	positive regulation of toll-like recepto...	13	8	2.67	1.49E-03
GO:0042094	interleukin-2 biosynthetic process	13	8	2.67	1.49E-03
GO:0006006	glucose metabolic process	136	43	27.95	1.50E-03
GO:0042107	cytokine metabolic process	61	23	12.54	1.50E-03
GO:0010959	regulation of metal ion transport	196	58	40.28	1.55E-03
GO:0008284	positive regulation of cell proliferatio...	544	140	111.8	1.57E-03
GO:0006754	ATP biosynthetic process	34	15	6.99	1.61E-03
GO:0009409	response to cold	34	15	6.99	1.61E-03

GO:0023056	positive regulation of signaling	810	200	166.46	1.63E-03
GO:0044057	regulation of system process	283	79	58.16	1.65E-03
GO:0000086	G2/M transition of mitotic cell cycle	58	22	11.92	1.72E-03
GO:0044770	cell cycle phase transition	246	70	50.56	1.73E-03
GO:0050848	regulation of calcium-mediated signaling	31	14	6.37	1.74E-03
GO:0045824	negative regulation of innate immune res...	16	9	3.29	1.79E-03
GO:0006958	complement activation, classical pathway	16	9	3.29	1.79E-03
GO:0002833	positive regulation of response to bioti...	16	9	3.29	1.79E-03
GO:0042060	wound healing	263	74	54.05	1.81E-03
GO:1902105	regulation of leukocyte differentiation	153	47	31.44	1.82E-03
GO:0030888	regulation of B cell proliferation	41	17	8.43	1.84E-03
GO:0070098	chemokine-mediated signaling pathway	28	13	5.75	1.86E-03
GO:0042325	regulation of phosphorylation	835	205	171.6	1.92E-03
GO:0032675	regulation of interleukin-6 production	62	23	12.74	1.93E-03
GO:0050701	interleukin-1 secretion	19	10	3.9	1.94E-03
GO:0006911	phagocytosis, engulfment	19	10	3.9	1.94E-03
GO:0050852	T cell receptor signaling pathway	48	19	9.86	1.96E-03
GO:0097306	cellular response to alcohol	84	29	17.26	1.97E-03
GO:0086010	membrane depolarization during action po...	22	11	4.52	1.98E-03
GO:1901698	response to nitrogen compound	644	162	132.35	1.99E-03
GO:0044772	mitotic cell cycle phase transition	239	68	49.12	2.00E-03
GO:0006119	oxidative phosphorylation	38	16	7.81	2.06E-03
GO:0007517	muscle organ development	260	73	53.43	2.06E-03
GO:0036294	cellular response to decreased oxygen le...	88	30	18.08	2.07E-03
GO:0014706	striated muscle tissue development	281	78	57.75	2.08E-03
GO:0010035	response to inorganic substance	400	106	82.2	2.08E-03
GO:0042127	regulation of cell proliferation	949	230	195.03	2.10E-03
GO:0001649	osteoblast differentiation	166	50	34.11	2.11E-03
GO:0003012	muscle system process	223	64	45.83	2.12E-03
GO:0009967	positive regulation of signal transducti...	774	191	159.06	2.13E-03
GO:0030316	osteoclast differentiation	59	22	12.13	2.22E-03
GO:0010647	positive regulation of cell communicatio...	815	200	167.49	2.23E-03

GO:0050714	positive regulation of protein secretion	81	28	16.65	2.28E-03
GO:0070231	T cell apoptotic process	35	15	7.19	2.29E-03
GO:0042116	macrophage activation	35	15	7.19	2.29E-03
GO:0097067	cellular response to thyroid hormone sti...	11	7	2.26	2.31E-03
GO:0002823	negative regulation of adaptive immune r...	11	7	2.26	2.31E-03
GO:0048875	chemical homeostasis within a tissue	11	7	2.26	2.31E-03
GO:0043383	negative T cell selection	11	7	2.26	2.31E-03
GO:0045060	negative thymic T cell selection	11	7	2.26	2.31E-03
GO:0002724	regulation of T cell cytokine production	11	7	2.26	2.31E-03
GO:0071900	regulation of protein serine/threonine k...	282	78	57.95	2.31E-03
GO:0042095	interferon-gamma biosynthetic process	11	7	2.26	2.31E-03
GO:0051924	regulation of calcium ion transport	131	41	26.92	2.35E-03
GO:0045859	regulation of protein kinase activity	436	114	89.6	2.36E-03
GO:0072358	cardiovascular system development	674	168	138.51	2.51E-03
GO:0072359	circulatory system development	674	168	138.51	2.51E-03
GO:0050709	negative regulation of protein secretion	32	14	6.58	2.52E-03
GO:0072376	protein activation cascade	32	14	6.58	2.52E-03
GO:0051241	negative regulation of multicellular org...	262	73	53.84	2.55E-03
GO:0060341	regulation of cellular localization	639	160	131.32	2.60E-03
GO:0043549	regulation of kinase activity	472	122	97	2.62E-03
GO:0042327	positive regulation of phosphorylation	507	130	104.19	2.63E-03
GO:0001934	positive regulation of protein phosphory...	420	110	86.31	2.64E-03
GO:0098656	anion transmembrane transport	97	32	19.93	2.73E-03
GO:0050954	sensory perception of mechanical stimulu...	82	28	16.85	2.80E-03
GO:0044839	cell cycle G2/M phase transition	60	22	12.33	2.84E-03
GO:0002275	myeloid cell activation involved in immu...	39	16	8.01	2.84E-03
GO:0002577	regulation of antigen processing and pre...	14	8	2.88	2.86E-03
GO:0051235	maintenance of location	201	58	41.31	2.93E-03
GO:0071456	cellular response to hypoxia	86	29	17.67	2.93E-03
GO:0009168	purine ribonucleoside monophosphate bios...	53	20	10.89	2.94E-03

GO:0009127	purine nucleoside monophosphate biosynth...	53	20	10.89	2.94E-03
GO:0006942	regulation of striated muscle contractio...	53	20	10.89	2.94E-03
GO:0006956	complement activation	26	12	5.34	2.94E-03
GO:0032722	positive regulation of chemokine product...	26	12	5.34	2.94E-03
GO:0033555	multicellular organismal response to str...	46	18	9.45	2.96E-03
GO:0045860	positive regulation of protein kinase ac...	268	74	55.08	3.09E-03
GO:0042391	regulation of membrane potential	268	74	55.08	3.09E-03
GO:0032635	interleukin-6 production	64	23	13.15	3.10E-03
GO:0032633	interleukin-4 production	23	11	4.73	3.10E-03
GO:0034113	heterotypic cell-cell adhesion	23	11	4.73	3.10E-03
GO:0097009	energy homeostasis	17	9	3.49	3.12E-03
GO:0002675	positive regulation of acute inflammator...	17	9	3.49	3.12E-03
GO:0071495	cellular response to endogenous stimulus	633	158	130.09	3.15E-03
GO:0001783	B cell apoptotic process	20	10	4.11	3.18E-03
GO:0014808	release of sequestered calcium ion into ...	20	10	4.11	3.18E-03
GO:0043270	positive regulation of ion transport	129	40	26.51	3.18E-03
GO:0002286	T cell activation involved in immune res...	36	15	7.4	3.18E-03
GO:0051897	positive regulation of protein kinase B ...	57	21	11.71	3.28E-03
GO:0032874	positive regulation of stress-activated ...	57	21	11.71	3.28E-03
GO:0016310	phosphorylation	1351	316	277.64	3.39E-03
GO:0051047	positive regulation of secretion	194	56	39.87	3.39E-03
GO:0002260	lymphocyte homeostasis	50	19	10.28	3.39E-03
GO:0009725	response to hormone	657	163	135.02	3.53E-03
GO:0032760	positive regulation of tumor necrosis fa...	33	14	6.78	3.55E-03
GO:0071214	cellular response to abiotic stimulus	178	52	36.58	3.55E-03
GO:0071229	cellular response to acid chemical	118	37	24.25	3.61E-03
GO:0032940	secretion by cell	485	124	99.67	3.63E-03
GO:0001101	response to acid chemical	257	71	52.82	3.65E-03
GO:0030198	extracellular matrix organization	134	41	27.54	3.71E-03
GO:0009187	cyclic nucleotide metabolic process	95	31	19.52	3.80E-03
GO:0060048	cardiac muscle contraction	65	23	13.36	3.89E-03
GO:0050853	B cell receptor signaling pathway	30	13	6.17	3.94E-03
GO:0006826	iron ion transport	30	13	6.17	3.94E-03

GO:0071398	cellular response to fatty acid	30	13	6.17	3.94E-03
GO:0009743	response to carbohydrate	175	51	35.96	4.07E-03
GO:0007162	negative regulation of cell adhesion	84	28	17.26	4.13E-03
GO:0007229	integrin-mediated signaling pathway	69	24	14.18	4.15E-03
GO:0070304	positive regulation of stress-activated ...	58	21	11.92	4.16E-03
GO:0010243	response to organonitrogen compound	602	150	123.72	4.25E-03
GO:0072593	reactive oxygen species metabolic proces...	123	38	25.28	4.25E-03
GO:0051896	regulation of protein kinase B signaling	88	29	18.08	4.28E-03
GO:0006164	purine nucleotide biosynthetic process	143	43	29.39	4.29E-03
GO:0043062	extracellular structure organization	135	41	27.74	4.30E-03
GO:0033280	response to vitamin D	27	12	5.55	4.32E-03
GO:0032653	regulation of interleukin-10 production	27	12	5.55	4.32E-03
GO:0046688	response to copper ion	27	12	5.55	4.32E-03
GO:0051051	negative regulation of transport	263	72	54.05	4.39E-03
GO:0006937	regulation of muscle contraction	92	30	18.91	4.41E-03
GO:0032570	response to progesterone	44	17	9.04	4.46E-03
GO:0010522	regulation of calcium ion transport into...	62	22	12.74	4.51E-03
GO:0015698	inorganic anion transport	62	22	12.74	4.51E-03
GO:0071731	response to nitric oxide	12	7	2.47	4.56E-03
GO:0043491	protein kinase B signaling	108	34	22.2	4.78E-03
GO:0008285	negative regulation of cell proliferatio...	410	106	84.26	4.79E-03
GO:0043271	negative regulation of ion transport	55	20	11.3	4.83E-03
GO:0045577	regulation of B cell differentiation	21	10	4.32	4.96E-03
GO:0002720	positive regulation of cytokine producti...	21	10	4.32	4.96E-03
GO:0002920	regulation of humoral immune response	21	10	4.32	4.96E-03
GO:0035588	G-protein coupled purinergic receptor si...	15	8	3.08	5.03E-03
GO:0006882	cellular zinc ion homeostasis	15	8	3.08	5.03E-03
GO:0034694	response to prostaglandin	15	8	3.08	5.03E-03
GO:0002438	acute inflammatory response to antigenic...	15	8	3.08	5.03E-03
GO:0061082	myeloid leukocyte cytokine production	18	9	3.7	5.11E-03
GO:0050764	regulation of phagocytosis	41	16	8.43	5.11E-03
GO:0071772	response to BMP	18	9	3.7	5.11E-03

GO:0071773	cellular response to BMP stimulus	18	9	3.7	5.11E-03
GO:0007606	sensory perception of chemical stimulus	41	16	8.43	5.11E-03
GO:0033674	positive regulation of kinase activity	286	77	58.78	5.22E-03
GO:0006820	anion transport	269	73	55.28	5.22E-03
GO:0046849	bone remodeling	59	21	12.13	5.24E-03
GO:1901699	cellular response to nitrogen compound	329	87	67.61	5.31E-03
GO:0034284	response to monosaccharide	157	46	32.26	5.52E-03
GO:1903047	mitotic cell cycle process	412	106	84.67	5.60E-03
GO:0035725	sodium ion transmembrane transport	63	22	12.95	5.61E-03
GO:0009749	response to glucose	141	42	28.98	5.68E-03
GO:0046330	positive regulation of JNK cascade	45	17	9.25	5.83E-03
GO:0048869	cellular developmental process	2360	529	485	5.93E-03
GO:0002831	regulation of response to biotic stimulu...	56	20	11.51	6.09E-03
GO:0051174	regulation of phosphorus metabolic proce...	1258	293	258.53	6.11E-03
GO:0035924	cellular response to vascular endothelia...	28	12	5.75	6.17E-03
GO:0060538	skeletal muscle organ development	158	46	32.47	6.27E-03
GO:0000278	mitotic cell cycle	475	120	97.62	6.30E-03
GO:0072522	purine-containing compound biosynthetic ...	150	44	30.83	6.39E-03
GO:0009888	tissue development	1204	281	247.43	6.48E-03
GO:0009156	ribonucleoside monophosphate biosyntheti...	60	21	12.33	6.53E-03
GO:1901654	response to ketone	134	40	27.54	6.57E-03
GO:0045778	positive regulation of ossification	35	14	7.19	6.67E-03
GO:0030099	myeloid cell differentiation	242	66	49.73	6.78E-03
GO:0002702	positive regulation of production of mol...	25	11	5.14	6.82E-03
GO:0032846	positive regulation of homeostatic proce...	64	22	13.15	6.93E-03
GO:0060548	negative regulation of cell death	539	134	110.77	7.24E-03
GO:0002335	mature B cell differentiation	10	6	2.06	7.30E-03
GO:0034143	regulation of toll-like receptor 4 signa...	10	6	2.06	7.30E-03
GO:0072604	interleukin-6 secretion	10	6	2.06	7.30E-03
GO:0043129	surfactant homeostasis	10	6	2.06	7.30E-03
GO:0032743	positive regulation of interleukin-2 pro...	10	6	2.06	7.30E-03
GO:0045072	regulation of interferon-gamma biosynthe...	10	6	2.06	7.30E-03

GO:0002921	negative regulation of humoral immune re...	10	6	2.06	7.30E-03
GO:0002903	negative regulation of B cell apoptotic ...	10	6	2.06	7.30E-03
GO:0072677	eosinophil migration	10	6	2.06	7.30E-03
GO:0032682	negative regulation of chemokine product...	10	6	2.06	7.30E-03
GO:0009746	response to hexose	147	43	30.21	7.35E-03
GO:0019220	regulation of phosphate metabolic proces...	1253	291	257.5	7.37E-03
GO:0033500	carbohydrate homeostasis	143	42	29.39	7.42E-03
GO:0042593	glucose homeostasis	143	42	29.39	7.42E-03
GO:2000514	regulation of CD4-positive, alpha-beta T...	22	10	4.52	7.44E-03
GO:0035587	purinergic receptor signaling pathway	22	10	4.52	7.44E-03
GO:0098661	inorganic anion transmembrane transport	46	17	9.45	7.51E-03
GO:0006821	chloride transport	46	17	9.45	7.51E-03
GO:0003009	skeletal muscle contraction	32	13	6.58	7.59E-03
GO:0043367	CD4-positive, alpha-beta T cell differen...	32	13	6.58	7.59E-03
GO:0032755	positive regulation of interleukin-6 pro...	32	13	6.58	7.59E-03
GO:0002285	lymphocyte activation involved in immune...	76	25	15.62	7.85E-03
GO:0051899	membrane depolarization	76	25	15.62	7.85E-03
GO:0045823	positive regulation of heart contraction	19	9	3.9	7.96E-03
GO:0046006	regulation of activated T cell prolifera...	19	9	3.9	7.96E-03
GO:0001773	myeloid dendritic cell activation	19	9	3.9	7.96E-03
GO:0010646	regulation of cell communication	1837	416	377.52	8.06E-03
GO:0070230	positive regulation of lymphocyte apopto...	13	7	2.67	8.14E-03
GO:0002507	tolerance induction	13	7	2.67	8.14E-03
GO:0042832	defense response to protozoan	13	7	2.67	8.14E-03
GO:0007519	skeletal muscle tissue development	152	44	31.24	8.25E-03
GO:0034142	toll-like receptor 4 signaling pathway	16	8	3.29	8.26E-03
GO:0032731	positive regulation of interleukin-1 bet...	16	8	3.29	8.26E-03
GO:0002755	MyD88-dependent toll-like receptor signa...	16	8	3.29	8.26E-03
GO:0055069	zinc ion homeostasis	16	8	3.29	8.26E-03
GO:0001562	response to protozoan	16	8	3.29	8.26E-03
GO:0042346	positive regulation of NF-kappaB import ...	16	8	3.29	8.26E-03
GO:1902533	positive regulation of intracellular sig...	519	129	106.66	8.37E-03

GO:0045619	regulation of lymphocyte differentiation	88	28	18.08	8.43E-03
GO:0031589	cell-substrate adhesion	202	56	41.51	8.45E-03
GO:0030183	B cell differentiation	65	22	13.36	8.48E-03
GO:0014823	response to activity	65	22	13.36	8.48E-03
GO:0032964	collagen biosynthetic process	29	12	5.96	8.59E-03
GO:0033628	regulation of cell adhesion mediated by ...	29	12	5.96	8.59E-03
GO:0006879	cellular iron ion homeostasis	29	12	5.96	8.59E-03
GO:0010951	negative regulation of endopeptidase act...	132	39	27.13	8.67E-03
GO:0051216	cartilage development	124	37	25.48	8.78E-03
GO:0022904	respiratory electron transport chain	36	14	7.4	8.89E-03
GO:0034103	regulation of tissue remodeling	36	14	7.4	8.89E-03
GO:0030816	positive regulation of cAMP metabolic pr...	36	14	7.4	8.89E-03
GO:2000026	regulation of multicellular organismal d...	1046	245	214.96	9.14E-03
GO:0014065	phosphatidylinositol 3-kinase signaling	73	24	15	9.15E-03
GO:0023051	regulation of signaling	1836	415	377.32	9.21E-03
GO:0070542	response to fatty acid	58	20	11.92	9.41E-03
GO:0048522	positive regulation of cellular process	2673	592	549.33	9.56E-03
GO:0045669	positive regulation of osteoblast differ...	47	17	9.66	9.56E-03
GO:0065007	biological regulation	6206	1322	1275.39	9.65E-03
GO:0030890	positive regulation of B cell proliferat...	26	11	5.34	9.66E-03
GO:0010466	negative regulation of peptidase activit...	133	39	27.33	9.90E-03
GO:0061061	muscle structure development	411	104	84.46	1.00E-02
GO:0009206	purine ribonucleoside triphosphate biosy...	40	15	8.22	1.01E-02
GO:0002712	regulation of B cell mediated immunity	33	13	6.78	1.02E-02
GO:0002889	regulation of immunoglobulin mediated im...	33	13	6.78	1.02E-02
GO:0045670	regulation of osteoclast differentiation	33	13	6.78	1.02E-02
GO:0043279	response to alkaloid	109	33	22.4	1.02E-02
GO:0009124	nucleoside monophosphate biosynthetic pr...	66	22	13.56	1.03E-02
GO:0006468	protein phosphorylation	1012	237	207.98	1.04E-02
GO:0007610	behavior	438	110	90.01	1.04E-02
GO:0008015	blood circulation	281	74	57.75	1.06E-02
GO:0050710	negative regulation of cytokine secretio...	23	10	4.73	1.08E-02

GO:0032870	cellular response to hormone stimulus	329	85	67.61	1.10E-02
GO:0051223	regulation of protein transport	299	78	61.45	1.13E-02
GO:0090257	regulation of muscle system process	130	38	26.72	1.14E-02
GO:0003013	circulatory system process	282	74	57.95	1.16E-02
GO:0019932	second-messenger-mediated signaling	122	36	25.07	1.16E-02
GO:0001501	skeletal system development	308	80	63.3	1.16E-02
GO:1902476	chloride transmembrane transport	37	14	7.6	1.17E-02
GO:0042345	regulation of NF-kappaB import into nucl...	30	12	6.17	1.17E-02
GO:0042348	NF-kappaB import into nucleus	30	12	6.17	1.17E-02
GO:0043551	regulation of phosphatidylinositol 3-kin...	30	12	6.17	1.17E-02
GO:0051281	positive regulation of release of seques...	20	9	4.11	1.19E-02
GO:0097066	response to thyroid hormone	20	9	4.11	1.19E-02
GO:0051094	positive regulation of developmental pro...	664	160	136.46	1.20E-02
GO:0051301	cell division	418	105	85.9	1.20E-02
GO:0045580	regulation of T cell differentiation	67	22	13.77	1.25E-02
GO:0008037	cell recognition	52	18	10.69	1.28E-02
GO:0055072	iron ion homeostasis	52	18	10.69	1.28E-02
GO:0032732	positive regulation of interleukin-1 pro...	17	8	3.49	1.28E-02
GO:0051926	negative regulation of calcium ion trans...	17	8	3.49	1.28E-02
GO:0002861	regulation of inflammatory response to a...	17	8	3.49	1.28E-02
GO:0032673	regulation of interleukin-4 production	17	8	3.49	1.28E-02
GO:0009145	purine nucleoside triphosphate biosynthe...	41	15	8.43	1.29E-02
GO:0055093	response to hyperoxia	41	15	8.43	1.29E-02
GO:0036296	response to increased oxygen levels	41	15	8.43	1.29E-02
GO:0051338	regulation of transferase activity	508	125	104.4	1.30E-02
GO:2000377	regulation of reactive oxygen species me...	75	24	15.41	1.31E-02
GO:0070555	response to interleukin-1	79	25	16.24	1.33E-02
GO:0061041	regulation of wound healing	79	25	16.24	1.33E-02
GO:0002467	germinal center formation	11	6	2.26	1.33E-02
GO:0014854	response to inactivity	11	6	2.26	1.33E-02
GO:0002579	positive regulation of antigen processin...	11	6	2.26	1.33E-02
GO:0002691	regulation of cellular extravasation	11	6	2.26	1.33E-02

GO:0044712	single-organism catabolic process	1230	283	252.78	1.33E-02
GO:0001782	B cell homeostasis	27	11	5.55	1.33E-02
GO:0050706	regulation of interleukin-1 beta secreti...	14	7	2.88	1.34E-02
GO:0043567	regulation of insulin-like growth factor...	14	7	2.88	1.34E-02
GO:0010460	positive regulation of heart rate	14	7	2.88	1.34E-02
GO:0042104	positive regulation of activated T cell ...	14	7	2.88	1.34E-02
GO:0050907	detection of chemical stimulus involved ...	14	7	2.88	1.34E-02
GO:0071478	cellular response to radiation	83	26	17.06	1.35E-02
GO:0033627	cell adhesion mediated by integrin	34	13	6.99	1.35E-02
GO:0071901	negative regulation of protein serine/th...	91	28	18.7	1.37E-02
GO:0070588	calcium ion transmembrane transport	95	29	19.52	1.37E-02
GO:0006094	gluconeogenesis	45	16	9.25	1.40E-02
GO:0071417	cellular response to organonitrogen comp...	302	78	62.06	1.45E-02
GO:0061448	connective tissue development	161	45	33.09	1.46E-02
GO:0051347	positive regulation of transferase activ...	311	80	63.91	1.48E-02
GO:0090068	positive regulation of cell cycle proces...	128	37	26.31	1.49E-02
GO:0009266	response to temperature stimulus	128	37	26.31	1.49E-02
GO:0001894	tissue homeostasis	124	36	25.48	1.51E-02
GO:0042088	T-helper 1 type immune response	24	10	4.93	1.51E-02
GO:0002931	response to ischemia	24	10	4.93	1.51E-02
GO:0019752	carboxylic acid metabolic process	596	144	122.48	1.52E-02
GO:0042542	response to hydrogen peroxide	116	34	23.84	1.54E-02
GO:0050766	positive regulation of phagocytosis	31	12	6.37	1.56E-02
GO:0071356	cellular response to tumor necrosis fact...	80	25	16.44	1.57E-02
GO:0006814	sodium ion transport	104	31	21.37	1.58E-02
GO:0071347	cellular response to interleukin-1	53	18	10.89	1.58E-02
GO:0044281	small molecule metabolic process	1866	418	383.48	1.60E-02
GO:0035094	response to nicotine	42	15	8.63	1.64E-02
GO:0046942	carboxylic acid transport	158	44	32.47	1.67E-02
GO:0015849	organic acid transport	158	44	32.47	1.67E-02
GO:0048545	response to steroid hormone	383	96	78.71	1.68E-02

GO:0043648	dicarboxylic acid metabolic process	61	20	12.54	1.70E-02
GO:0034109	homotypic cell-cell adhesion	61	20	12.54	1.70E-02
GO:0048872	homeostasis of number of cells	175	48	35.96	1.70E-02
GO:2000108	positive regulation of leukocyte apoptot...	21	9	4.32	1.71E-02
GO:0033189	response to vitamin A	21	9	4.32	1.71E-02
GO:0046835	carbohydrate phosphorylation	21	9	4.32	1.71E-02
GO:0031069	hair follicle morphogenesis	21	9	4.32	1.71E-02
GO:0008038	neuron recognition	21	9	4.32	1.71E-02
GO:0042269	regulation of natural killer cell mediat...	21	9	4.32	1.71E-02
GO:0002715	regulation of natural killer cell mediat...	21	9	4.32	1.71E-02
GO:0050927	positive regulation of positive chemotax...	21	9	4.32	1.71E-02
GO:1903115	regulation of actin filament-based movem...	35	13	7.19	1.75E-02
GO:0009201	ribonucleoside triphosphate biosynthetic...	46	16	9.45	1.75E-02
GO:0050871	positive regulation of B cell activation	46	16	9.45	1.75E-02
GO:0014068	positive regulation of phosphatidylinosi...	46	16	9.45	1.75E-02
GO:0015711	organic anion transport	205	55	42.13	1.75E-02
GO:0050953	sensory perception of light stimulus	46	16	9.45	1.75E-02
GO:0030801	positive regulation of cyclic nucleotide...	46	16	9.45	1.75E-02
GO:0046058	cAMP metabolic process	69	22	14.18	1.78E-02
GO:0032615	interleukin-12 production	28	11	5.75	1.80E-02
GO:0050732	negative regulation of peptidyl-tyrosine...	28	11	5.75	1.80E-02
GO:0001659	temperature homeostasis	28	11	5.75	1.80E-02
GO:0050731	positive regulation of peptidyl-tyrosine...	105	31	21.58	1.81E-02
GO:0051302	regulation of cell division	163	45	33.5	1.82E-02
GO:0071322	cellular response to carbohydrate stimul...	85	26	17.47	1.84E-02
GO:0010941	regulation of cell death	1002	232	205.92	1.85E-02
GO:0043410	positive regulation of MAPK cascade	266	69	54.67	1.86E-02
GO:0043086	negative regulation of catalytic activit...	429	106	88.16	1.87E-02
GO:2000146	negative regulation of cell motility	134	38	27.54	1.87E-02
GO:0034349	glial cell apoptotic process	18	8	3.7	1.90E-02
GO:0002548	monocyte chemotaxis	18	8	3.7	1.90E-02
GO:0010880	regulation of release of sequestered cal...	18	8	3.7	1.90E-02
GO:0033762	response to glucagon	18	8	3.7	1.90E-02

GO:0032967	positive regulation of collagen biosynth...	18	8	3.7	1.90E-02
GO:0050918	positive chemotaxis	39	14	8.01	1.91E-02
GO:0090183	regulation of kidney development	39	14	8.01	1.91E-02
GO:0009142	nucleoside triphosphate biosynthetic pro...	54	18	11.1	1.92E-02
GO:0035690	cellular response to drug	54	18	11.1	1.92E-02
GO:0050886	endocrine process	54	18	11.1	1.92E-02
GO:0006793	phosphorus metabolic process	2569	565	527.95	1.98E-02
GO:0042886	amide transport	147	41	30.21	1.98E-02
GO:0048678	response to axon injury	58	19	11.92	1.99E-02
GO:0006112	energy reserve metabolic process	62	20	12.74	2.04E-02
GO:0043113	receptor clustering	32	12	6.58	2.04E-02
GO:0009612	response to mechanical stimulus	160	44	32.88	2.07E-02
GO:1902305	regulation of sodium ion transmembrane t...	25	10	5.14	2.07E-02
GO:0086009	membrane repolarization	25	10	5.14	2.07E-02
GO:0050869	negative regulation of B cell activation	25	10	5.14	2.07E-02
GO:0050704	regulation of interleukin-1 secretion	15	7	3.08	2.08E-02
GO:0042119	neutrophil activation	15	7	3.08	2.08E-02
GO:0034105	positive regulation of tissue remodeling	15	7	3.08	2.08E-02
GO:2000516	positive regulation of CD4-positive, alp...	15	7	3.08	2.08E-02
GO:0045822	negative regulation of heart contraction	15	7	3.08	2.08E-02
GO:0006909	phagocytosis	86	26	17.67	2.14E-02
GO:0071902	positive regulation of protein serine/th...	173	47	35.55	2.14E-02
GO:0030336	negative regulation of cell migration	131	37	26.92	2.14E-02
GO:0010038	response to metal ion	259	67	53.23	2.14E-02
GO:0006085	acetyl-CoA biosynthetic process	12	6	2.47	2.20E-02
GO:0010935	regulation of macrophage cytokine produc...	12	6	2.47	2.20E-02
GO:0032891	negative regulation of organic acid tran...	12	6	2.47	2.20E-02
GO:0007263	nitric oxide mediated signal transductio...	12	6	2.47	2.20E-02
GO:0010269	response to selenium ion	12	6	2.47	2.20E-02
GO:0060307	regulation of ventricular cardiac muscle...	12	6	2.47	2.20E-02
GO:0048266	behavioral response to pain	12	6	2.47	2.20E-02
GO:0031268	pseudopodium organization	12	6	2.47	2.20E-02
GO:0031269	pseudopodium assembly	12	6	2.47	2.20E-02
GO:0001660	fever generation	12	6	2.47	2.20E-02

GO:0009966	regulation of signal transduction	1655	371	340.12	2.23E-02
GO:1901264	carbohydrate derivative transport	36	13	7.4	2.24E-02
GO:0050729	positive regulation of inflammatory resp...	55	18	11.3	2.32E-02
GO:0043069	negative regulation of programmed cell d...	491	119	100.91	2.35E-02
GO:0009152	purine ribonucleotide biosynthetic proce...	136	38	27.95	2.35E-02
GO:0051271	negative regulation of cellular componen...	136	38	27.95	2.35E-02
GO:0050854	regulation of antigen receptor-mediated ...	29	11	5.96	2.37E-02
GO:0043278	response to morphine	22	9	4.52	2.37E-02
GO:0007608	sensory perception of smell	22	9	4.52	2.37E-02
GO:0050926	regulation of positive chemotaxis	22	9	4.52	2.37E-02
GO:0014066	regulation of phosphatidylinositol 3-kin...	59	19	12.13	2.38E-02
GO:0050673	epithelial cell proliferation	230	60	47.27	2.40E-02
GO:0007160	cell-matrix adhesion	128	36	26.31	2.45E-02
GO:0044070	regulation of anion transport	67	21	13.77	2.45E-02
GO:0030799	regulation of cyclic nucleotide metaboli...	67	21	13.77	2.45E-02
GO:0010942	positive regulation of cell death	327	82	67.2	2.53E-02
GO:0030810	positive regulation of nucleotide biosyn...	44	15	9.04	2.54E-02
GO:1900373	positive regulation of purine nucleotide...	44	15	9.04	2.54E-02
GO:0061035	regulation of cartilage development	44	15	9.04	2.54E-02
GO:0007601	visual perception	44	15	9.04	2.54E-02
GO:0050789	regulation of biological process	5920	1256	1216.62	2.57E-02
GO:0022408	negative regulation of cell-cell adhesio...	33	12	6.78	2.62E-02
GO:0006953	acute-phase response	33	12	6.78	2.62E-02
GO:0044723	single-organism carbohydrate metabolic p...	421	103	86.52	2.62E-02
GO:0050679	positive regulation of epithelial cell p...	112	32	23.02	2.63E-02
GO:0034110	regulation of homotypic cell-cell adhesi...	19	8	3.9	2.70E-02
GO:0010714	positive regulation of collagen metaboli...	19	8	3.9	2.70E-02
GO:0002544	chronic inflammatory response	19	8	3.9	2.70E-02
GO:0045597	positive regulation of cell differentiat...	480	116	98.64	2.71E-02

GO:0046364	monosaccharide biosynthetic process	52	17	10.69	2.72E-02
GO:0051781	positive regulation of cell division	52	17	10.69	2.72E-02
GO:1901652	response to peptide	306	77	62.89	2.73E-02
GO:0002228	natural killer cell mediated immunity	26	10	5.34	2.76E-02
GO:0042267	natural killer cell mediated cytotoxicit...	26	10	5.34	2.76E-02
GO:0061098	positive regulation of protein tyrosine ...	26	10	5.34	2.76E-02
GO:0042993	positive regulation of transcription fac...	26	10	5.34	2.76E-02
GO:0048015	phosphatidylinositol-mediated signaling	92	27	18.91	2.81E-02
GO:0048017	inositol lipid-mediated signaling	92	27	18.91	2.81E-02
GO:0070201	regulation of establishment of protein l...	333	83	68.43	2.82E-02
GO:0045576	mast cell activation	37	13	7.6	2.82E-02
GO:0051928	positive regulation of calcium ion trans...	60	19	12.33	2.83E-02
GO:0051606	detection of stimulus	84	25	17.26	2.86E-02
GO:0015833	peptide transport	142	39	29.18	2.86E-02
GO:0043066	negative regulation of apoptotic process	481	116	98.85	2.87E-02
GO:0001678	cellular glucose homeostasis	80	24	16.44	2.88E-02
GO:0050678	regulation of epithelial cell proliferat...	202	53	41.51	2.92E-02
GO:0051091	positive regulation of sequence-specific...	138	38	28.36	2.93E-02
GO:0043434	response to peptide hormone	285	72	58.57	2.94E-02
GO:0022900	electron transport chain	41	14	8.43	2.98E-02
GO:0061097	regulation of protein tyrosine kinase ac...	41	14	8.43	2.98E-02
GO:0050906	detection of stimulus involved in sensor...	41	14	8.43	2.98E-02
GO:0000280	nuclear division	272	69	55.9	2.98E-02
GO:0048738	cardiac muscle tissue development	134	37	27.54	3.00E-02
GO:0036230	granulocyte activation	16	7	3.29	3.05E-02
GO:0009214	cyclic nucleotide catabolic process	16	7	3.29	3.05E-02
GO:0043949	regulation of cAMP-mediated signaling	16	7	3.29	3.05E-02
GO:0043011	myeloid dendritic cell differentiation	16	7	3.29	3.05E-02
GO:0006898	receptor-mediated endocytosis	130	36	26.72	3.06E-02
GO:0002637	regulation of immunoglobulin production	30	11	6.17	3.07E-02
GO:0045582	positive regulation of T cell differenti...	45	15	9.25	3.10E-02

GO:0055117	regulation of cardiac muscle contraction	45	15	9.25	3.10E-02
GO:0002791	regulation of peptide secretion	101	29	20.76	3.12E-02
GO:0043200	response to amino acid	101	29	20.76	3.12E-02
GO:0006082	organic acid metabolic process	637	150	130.91	3.12E-02
GO:0007267	cell-cell signaling	537	128	110.36	3.15E-02
GO:0071822	protein complex subunit organization	992	227	203.87	3.16E-02
GO:0031401	positive regulation of protein modificat...	528	126	108.51	3.16E-02
GO:0070252	actin-mediated cell contraction	49	16	10.07	3.20E-02
GO:0019319	hexose biosynthetic process	49	16	10.07	3.20E-02
GO:0014072	response to isoquinoline alkaloid	23	9	4.73	3.20E-02
GO:0036075	replacement ossification	23	9	4.73	3.20E-02
GO:0001958	endochondral ossification	23	9	4.73	3.20E-02
GO:0055081	anion homeostasis	23	9	4.73	3.20E-02
GO:1902107	positive regulation of leukocyte differe...	89	26	18.29	3.25E-02
GO:0071384	cellular response to corticosteroid stim...	53	17	10.89	3.27E-02
GO:1900046	regulation of hemostasis	53	17	10.89	3.27E-02
GO:0030193	regulation of blood coagulation	53	17	10.89	3.27E-02
GO:0019233	sensory perception of pain	69	21	14.18	3.36E-02
GO:0006796	phosphate-containing compound metabolic ...	2541	555	522.2	3.39E-02
GO:0050716	positive regulation of interleukin-1 sec...	13	6	2.67	3.39E-02
GO:0050718	positive regulation of interleukin-1 bet...	13	6	2.67	3.39E-02
GO:0046629	gamma-delta T cell activation	13	6	2.67	3.39E-02
GO:0010934	macrophage cytokine production	13	6	2.67	3.39E-02
GO:2000573	positive regulation of DNA biosynthetic ...	13	6	2.67	3.39E-02
GO:0032928	regulation of superoxide anion generatio...	13	6	2.67	3.39E-02
GO:0032303	regulation of icosanoid secretion	13	6	2.67	3.39E-02
GO:0032487	regulation of Rap protein signal transdu...	13	6	2.67	3.39E-02
GO:0048485	sympathetic nervous system development	13	6	2.67	3.39E-02
GO:0042573	retinoic acid metabolic process	13	6	2.67	3.39E-02
GO:0007567	parturition	13	6	2.67	3.39E-02
GO:0043067	regulation of programmed cell death	943	216	193.8	3.42E-02

GO:0032386	regulation of intracellular transport	296	74	60.83	3.43E-02
GO:0043436	oxoacid metabolic process	630	148	129.47	3.46E-02
GO:0006801	superoxide metabolic process	38	13	7.81	3.50E-02
GO:0045453	bone resorption	38	13	7.81	3.50E-02
GO:0071695	anatomical structure maturation	38	13	7.81	3.50E-02
GO:0032880	regulation of protein localization	399	97	82	3.52E-02
GO:0048519	negative regulation of biological proces...	2638	575	542.13	3.52E-02
GO:0022402	cell cycle process	635	149	130.5	3.54E-02
GO:0044262	cellular carbohydrate metabolic process	200	52	41.1	3.57E-02
GO:0010712	regulation of collagen metabolic process	27	10	5.55	3.59E-02
GO:0032655	regulation of interleukin-12 production	27	10	5.55	3.59E-02
GO:0060350	endochondral bone morphogenesis	42	14	8.63	3.65E-02
GO:0001658	branching involved in ureteric bud morph...	42	14	8.63	3.65E-02
GO:0032677	regulation of interleukin-8 production	42	14	8.63	3.65E-02
GO:0050855	regulation of B cell receptor signaling ...	10	5	2.06	3.65E-02
GO:1900744	regulation of p38MAPK cascade	10	5	2.06	3.65E-02
GO:0071243	cellular response to arsenic-containing ...	10	5	2.06	3.65E-02
GO:0071467	cellular response to pH	10	5	2.06	3.65E-02
GO:0033275	actin-myosin filament sliding	10	5	2.06	3.65E-02
GO:0050857	positive regulation of antigen receptor-...	10	5	2.06	3.65E-02
GO:0010447	response to acidic pH	10	5	2.06	3.65E-02
GO:0031338	regulation of vesicle fusion	10	5	2.06	3.65E-02
GO:0006012	galactose metabolic process	10	5	2.06	3.65E-02
GO:0061081	positive regulation of myeloid leukocyte...	10	5	2.06	3.65E-02
GO:0002864	regulation of acute inflammatory respons...	10	5	2.06	3.65E-02
GO:0045066	regulatory T cell differentiation	10	5	2.06	3.65E-02
GO:0055119	relaxation of cardiac muscle	10	5	2.06	3.65E-02
GO:0046031	ADP metabolic process	10	5	2.06	3.65E-02
GO:0006026	aminoglycan catabolic process	10	5	2.06	3.65E-02
GO:0086019	cell-cell signaling involved in cardiac ...	10	5	2.06	3.65E-02
GO:0031652	positive regulation of heat generation	10	5	2.06	3.65E-02
GO:0048535	lymph node development	10	5	2.06	3.65E-02

GO:0090184	positive regulation of kidney developmen...	10	5	2.06	3.65E-02
GO:0032753	positive regulation of interleukin-4 pro...	10	5	2.06	3.65E-02
GO:0000082	G1/S transition of mitotic cell cycle	119	33	24.46	3.66E-02
GO:0060306	regulation of membrane repolarization	20	8	4.11	3.72E-02
GO:0048265	response to pain	20	8	4.11	3.72E-02
GO:0043536	positive regulation of blood vessel endo...	20	8	4.11	3.72E-02
GO:0008217	regulation of blood pressure	115	32	23.63	3.74E-02
GO:0071230	cellular response to amino acid stimulus	46	15	9.45	3.75E-02
GO:0051222	positive regulation of protein transport	179	47	36.79	3.77E-02
GO:0060359	response to ammonium ion	50	16	10.28	3.83E-02
GO:0046637	regulation of alpha-beta T cell differen...	31	11	6.37	3.90E-02
GO:0045668	negative regulation of osteoblast differ...	31	11	6.37	3.90E-02
GO:0002377	immunoglobulin production	58	18	11.92	3.91E-02
GO:0005977	glycogen metabolic process	58	18	11.92	3.91E-02
GO:0006073	cellular glucan metabolic process	58	18	11.92	3.91E-02
GO:0044042	glucan metabolic process	58	18	11.92	3.91E-02
GO:0048285	organelle fission	298	74	61.24	3.94E-02
GO:0090087	regulation of peptide transport	103	29	21.17	3.98E-02
GO:0045786	negative regulation of cell cycle	184	48	37.81	4.00E-02
GO:0044843	cell cycle G1/S phase transition	120	33	24.66	4.08E-02
GO:0072521	purine-containing compound metabolic pro...	1008	229	207.15	4.08E-02
GO:0031663	lipopolysaccharide-mediated signaling pa...	35	12	7.19	4.12E-02
GO:0009593	detection of chemical stimulus	35	12	7.19	4.12E-02
GO:0032787	monocarboxylic acid metabolic process	330	81	67.82	4.16E-02
GO:0033003	regulation of mast cell activation	24	9	4.93	4.22E-02
GO:0048730	epidermis morphogenesis	24	9	4.93	4.22E-02
GO:0044253	positive regulation of multicellular org...	24	9	4.93	4.22E-02
GO:0032094	response to food	24	9	4.93	4.22E-02
GO:0048585	negative regulation of response to stimu...	777	179	159.68	4.23E-02
GO:0032147	activation of protein kinase activity	146	39	30	4.29E-02
GO:0014850	response to muscle activity	17	7	3.49	4.29E-02
GO:0038084	vascular endothelial growth factor signa...	17	7	3.49	4.29E-02

GO:0031649	heat generation	17	7	3.49	4.29E-02
GO:0061217	regulation of mesonephros development	17	7	3.49	4.29E-02
GO:0050881	musculoskeletal movement	39	13	8.01	4.29E-02
GO:0050879	multicellular organismal movement	39	13	8.01	4.29E-02
GO:0002790	peptide secretion	129	35	26.51	4.32E-02
GO:0032872	regulation of stress-activated MAPK casc...	129	35	26.51	4.32E-02
GO:0052547	regulation of peptidase activity	242	61	49.73	4.39E-02
GO:0055008	cardiac muscle tissue morphogenesis	43	14	8.84	4.42E-02
GO:0071333	cellular response to glucose stimulus	75	22	15.41	4.46E-02
GO:0015718	monocarboxylic acid transport	75	22	15.41	4.46E-02
GO:0019722	calcium-mediated signaling	75	22	15.41	4.46E-02
GO:0008219	cell death	1235	277	253.8	4.50E-02
GO:0060675	ureteric bud morphogenesis	47	15	9.66	4.50E-02
GO:0043068	positive regulation of programmed cell d...	300	74	61.65	4.50E-02
GO:0050768	negative regulation of neurogenesis	71	21	14.59	4.51E-02
GO:0051346	negative regulation of hydrolase activit...	216	55	44.39	4.52E-02
GO:0007059	chromosome segregation	121	33	24.87	4.54E-02
GO:0060415	muscle tissue morphogenesis	51	16	10.48	4.55E-02
GO:0071385	cellular response to glucocorticoid stim...	51	16	10.48	4.55E-02
GO:0045981	positive regulation of nucleotide metabo...	63	19	12.95	4.57E-02
GO:1900544	positive regulation of purine nucleotide...	63	19	12.95	4.57E-02
GO:0000910	cytokinesis	63	19	12.95	4.57E-02
GO:0000079	regulation of cyclin-dependent protein s...	55	17	11.3	4.58E-02
GO:0042981	regulation of apoptotic process	932	212	191.53	4.58E-02
GO:0031099	regeneration	151	40	31.03	4.59E-02
GO:0000060	protein import into nucleus, translocati...	28	10	5.75	4.59E-02
GO:0052548	regulation of endopeptidase activity	234	59	48.09	4.67E-02
GO:0009260	ribonucleotide biosynthetic process	147	39	30.21	4.72E-02
GO:0070371	ERK1 and ERK2 cascade	147	39	30.21	4.72E-02
GO:0010721	negative regulation of cell development	92	26	18.91	4.75E-02
GO:0040013	negative regulation of locomotion	160	42	32.88	4.76E-02
GO:0070302	regulation of stress-activated protein k...	130	35	26.72	4.78E-02

GO:1901215	negative regulation of neuron death	130	35	26.72	4.78E-02
GO:0086004	regulation of cardiac muscle cell contra...	32	11	6.58	4.87E-02
GO:0007528	neuromuscular junction development	32	11	6.58	4.87E-02
GO:0030819	positive regulation of cAMP biosynthetic...	32	11	6.58	4.87E-02
GO:0003044	regulation of systemic arterial blood pr...	32	11	6.58	4.87E-02
GO:0048705	skeletal system morphogenesis	156	41	32.06	4.89E-02
GO:0006309	apoptotic DNA fragmentation	14	6	2.88	4.93E-02
GO:0032660	regulation of interleukin-17 production	14	6	2.88	4.93E-02
GO:0060999	positive regulation of dendritic spine d...	14	6	2.88	4.93E-02
GO:0007413	axonal fasciculation	14	6	2.88	4.93E-02
GO:0050901	leukocyte tethering or rolling	14	6	2.88	4.93E-02
GO:0006198	cAMP catabolic process	14	6	2.88	4.93E-02
GO:0032332	positive regulation of chondrocyte diffe...	14	6	2.88	4.93E-02
GO:0002320	lymphoid progenitor cell differentiation	14	6	2.88	4.93E-02
GO:0050930	induction of positive chemotaxis	14	6	2.88	4.93E-02
GO:0098581	detection of external biotic stimulus	14	6	2.88	4.93E-02
GO:0035590	purinergic nucleotide receptor signaling...	14	6	2.88	4.93E-02
GO:0002686	negative regulation of leukocyte migrati...	14	6	2.88	4.93E-02
GO:0043552	positive regulation of phosphatidylinosi...	21	8	4.32	4.95E-02
GO:0045124	regulation of bone resorption	21	8	4.32	4.95E-02
GO:0007157	heterophilic cell-cell adhesion	21	8	4.32	4.95E-02
GO:0035136	forelimb morphogenesis	21	8	4.32	4.95E-02
