# Supplementary material for: A robust neuromuscular system protects rat and human skeletal muscle from sarcopenia
Source: Aging (Albany NY). 2016 Mar 24;8(4):712–28. doi: 10.18632/aging.100926 (PMC4925824; doi:10.18632/aging.100926)
Supplement: Supplementary file 3 [file aging-08-712-s003.pdf]

2 by 3 factorial design of muscle RNAseq (Gastocnemius vs Triceps at 8m / 18m / 24m of age)
Model: Gene ~ b0 + b1MuscleType8m + b2Age18m + b3Age24m + b4Age18m:MuscleType + b5Age24m:MuscleType

Reference group: Adult 8m gastrocnemius

Gene ID	Gene Symbol	"log2FC MuscleType Triceps/Gastroc 8m"	log2FC Age_18m	log2FC Age_24m	"log2FC MuscleType:Age 8m-18m"	"log2FC MuscleType:Age 8m-24m"	adj. p-value MuscleType:Age 8m-24m
ENSRNOG00000007102	Acss1	-0.693	-0.539	-1.437	1.006	2.087	1.31E-06
ENSRNOG00000004932	Sel1l3	0.373	0.553	0.809	-0.759	-1.253	1.31E-06
ENSRNOG00000042980	Adam19	0.007	0.898	2.037	-1.032	-1.827	1.42E-06
ENSRNOG00000020369	Igf2	-0.196	0.966	2.475	-0.883	-2.132	1.49E-06
ENSRNOG00000047046	Plin4	-0.143	-0.442	-1.465	0.551	1.486	2.04E-06
ENSRNOG00000013674	Megf10	0.282	0.171	0.917	-0.518	-1.346	2.04E-06
ENSRNOG00000055089	Slc44a1	-0.020	0.480	1.077	-0.529	-0.856	4.42E-06
ENSRNOG00000019826	Sil1	0.049	0.584	1.254	-0.522	-1.228	4.42E-06
ENSRNOG00000000521	Cdkn1a	0.200	3.087	4.410	-2.429	-2.889	4.42E-06
ENSRNOG00000002451	Fndc3c1	0.690	0.972	2.208	-2.583	-3.520	4.42E-06
ENSRNOG00000000585	Amd1	0.026	-0.383	-0.905	0.318	0.798	7.91E-06
ENSRNOG00000001891	Gnb1l	0.220	0.484	0.848	-0.407	-0.985	1.64E-05
ENSRNOG00000046171	Mlxipl	-0.254	-0.626	-1.747	0.540	1.588	2.47E-05
ENSRNOG00000020779	Kcna7	0.189	-0.580	-1.460	0.543	1.303	2.47E-05
ENSRNOG00000003452	Asb11	0.204	-0.665	-1.510	0.633	1.295	2.47E-05
ENSRNOG00000024885	Asb10	-0.044	-0.482	-1.496	0.608	1.218	2.47E-05
ENSRNOG00000026679	Scn4b	0.052	-0.262	-1.164	0.256	1.139	2.47E-05
ENSRNOG00000007596	Rffl	-0.176	-0.179	-0.517	0.349	0.646	2.47E-05
ENSRNOG00000034198	Wbp5	0.388	0.277	0.959	-0.352	-0.926	2.47E-05
ENSRNOG00000009225	Copz2	0.175	0.876	1.474	-0.949	-1.387	2.47E-05

ENSRNOG00000024904	Pla2g4e	0.844	0.919	1.658	-1.408	-2.035	2.47E-05
ENSRNOG00000051548	Lmod1	0.920	-1.002	-2.567	1.142	2.127	2.83E-05
ENSRNOG00000057601	AABR07044080.2	-0.660	-0.275	-1.158	0.730	1.806	2.99E-05
ENSRNOG00000029490	LOC102553763	-0.116	-0.297	-0.831	0.278	0.674	2.99E-05
ENSRNOG00000060248	Chchd6	0.184	0.616	1.427	-0.917	-1.561	2.99E-05
ENSRNOG00000003832	Vash2	0.192	1.229	2.263	-1.061	-2.003	2.99E-05
ENSRNOG00000020194	Hes6	-0.081	0.418	0.874	-0.401	-0.931	3.37E-05
ENSRNOG00000053560	Rhou	-0.687	-0.350	-1.234	0.649	1.758	3.50E-05
ENSRNOG00000055408	Gcat	0.327	-0.312	-0.967	0.341	1.058	3.50E-05
ENSRNOG00000002028	Tmem50b	-0.109	-0.353	-1.180	0.282	0.928	3.50E-05
ENSRNOG00000002365	Itm2a	0.668	-0.107	0.315	-0.365	-1.043	3.50E-05
ENSRNOG00000016038	Mgmt	0.062	1.436	2.589	-1.347	-1.927	3.50E-05
ENSRNOG00000022723	RGD1562029	-0.540	-1.090	-2.380	0.896	2.178	4.08E-05
ENSRNOG00000014166	Smoc2	0.111	0.448	1.122	-0.603	-1.145	4.12E-05
ENSRNOG00000060972	Heca	-0.107	-0.218	-0.586	0.246	0.647	4.13E-05
ENSRNOG00000021014	Sec1	1.148	1.814	3.420	-2.126	-2.886	4.13E-05
ENSRNOG00000027300	Ttc7b	-0.066	-0.333	-1.052	0.287	0.769	4.19E-05
ENSRNOG00000002977	Ttc19	-0.028	-0.500	-1.235	0.399	0.943	4.44E-05
ENSRNOG00000017496	Cnp	-0.248	0.551	1.588	-0.420	-1.197	4.44E-05
ENSRNOG00000010283	Cd28	0.310	1.808	3.497	-2.260	-3.003	4.89E-05
ENSRNOG00000019205	Gnpat	0.010	-0.358	-0.823	0.368	0.764	5.05E-05
ENSRNOG00000033316	Foxo4	0.122	-0.308	-0.891	0.315	0.746	5.16E-05
ENSRNOG00000025705	Armcx2	-0.330	0.483	1.955	-0.295	-1.802	5.53E-05
ENSRNOG00000042189	Rab31	-0.072	0.686	1.534	-0.684	-1.163	6.16E-05
ENSRNOG00000011518	Dusp26	0.720	-0.504	-1.199	0.473	0.904	6.18E-05
ENSRNOG00000014241	Ece1	-0.027	-0.272	-0.689	0.285	0.801	6.18E-05
ENSRNOG00000050748	Dpp9	-0.093	-0.175	-0.608	0.226	0.550	6.18E-05

ENSRNOG00000015420	Stxbp1	-0.069	0.508	1.158	-0.370	-0.811	6.18E-05
ENSRNOG00000027756	RGD1560175	0.059	0.759	1.207	-0.797	-1.190	6.18E-05
ENSRNOG00000025418	Armc9	0.094	0.243	0.776	-0.278	-0.893	6.38E-05
ENSRNOG00000000560	Lrrc20	0.163	-0.385	-1.187	0.342	0.941	6.62E-05
ENSRNOG00000015019	Spg21	-0.101	0.735	1.594	-0.703	-1.137	6.71E-05
ENSRNOG00000004763	Sirpa	0.065	0.973	1.745	-0.752	-1.196	6.71E-05
ENSRNOG00000009745	Ppp3cc	0.163	-0.355	-0.837	0.280	0.650	6.92E-05
ENSRNOG00000031824	Slc44a2	0.200	0.364	0.568	-0.417	-0.625	6.92E-05
ENSRNOG00000006733	Srgap2	-0.039	0.268	0.809	-0.225	-0.729	6.97E-05
ENSRNOG00000043192	Hacd1	0.074	0.085	0.404	-0.494	-0.979	7.07E-05
ENSRNOG00000021365	Ybey	0.122	-0.443	-1.217	0.357	0.968	7.44E-05
ENSRNOG00000019773	AABR07044089.1	0.109	-0.336	-0.991	0.340	0.719	7.96E-05
ENSRNOG00000036664	Narf	0.082	-0.423	-0.823	0.223	0.589	7.97E-05
ENSRNOG00000031851	Ndufa4l2	-0.344	-0.636	-1.016	0.392	1.272	8.06E-05
ENSRNOG00000021200	Hfe2	0.193	-0.229	-1.122	0.272	0.992	8.06E-05
ENSRNOG00000004172	Pdk2	0.030	-0.314	-1.110	0.341	0.928	8.06E-05
ENSRNOG00000006756	Maged1	-0.140	0.285	0.787	-0.396	-0.727	8.06E-05
ENSRNOG00000008235	Mylk2	0.664	-0.607	-1.828	0.551	1.348	8.48E-05
ENSRNOG00000039463	Pcdhga1	0.218	-0.456	-1.558	0.366	1.434	9.03E-05
ENSRNOG00000018239	Dhrs4	-0.555	-0.262	-0.839	0.490	1.086	9.03E-05
ENSRNOG00000017548	Fam53a	-0.189	-0.346	-0.984	0.322	0.744	9.03E-05
ENSRNOG00000005046	Tspan13	0.832	0.628	1.015	-0.522	-1.053	9.03E-05
ENSRNOG00000026592	Rhbdl2	-0.191	2.191	3.508	-3.050	-4.315	9.03E-05
ENSRNOG00000005565	Traf3ip3	-0.127	1.330	2.067	-0.780	-1.390	9.24E-05
ENSRNOG00000017932	St3gal2	0.386	-0.458	-1.097	0.443	0.875	9.27E-05
ENSRNOG00000004882	Capn6	0.095	0.431	1.317	-0.525	-1.419	9.27E-05
ENSRNOG00000052307	Spats2	-0.308	0.205	1.174	-0.469	-1.143	9.31E-05

ENSRNOG00000046763	Adssl1	0.077	-0.472	-1.346	0.490	1.067	9.34E-05
ENSRNOG00000014051	Pgrmc2	-0.078	0.226	0.578	-0.164	-0.439	9.34E-05
ENSRNOG00000032922	Dclk1	-0.017	1.679	2.690	-1.718	-2.319	9.34E-05
ENSRNOG00000013346	Asb14	0.159	-0.338	-0.962	0.313	0.694	9.38E-05
ENSRNOG00000049681	Mapre2	0.029	-0.217	-0.456	0.194	0.432	9.90E-05
ENSRNOG00000003927	Cd55	0.434	0.022	-0.834	0.210	0.907	1.01E-04
ENSRNOG00000017801	Atf4	0.214	-0.227	-0.638	0.281	0.693	1.01E-04
ENSRNOG00000004442	RGD1311756	-0.487	-0.091	-0.751	0.267	1.042	1.03E-04
ENSRNOG00000001911	Map6d1	0.389	0.890	2.334	-1.963	-3.556	1.03E-04
ENSRNOG00000001924	Tp63	0.426	1.117	1.621	-1.108	-1.447	1.04E-04
ENSRNOG00000021440	Pptc7	-0.282	-0.528	-1.110	0.391	0.938	1.06E-04
ENSRNOG00000018797	Myrip	-0.101	-0.264	-0.661	0.421	0.957	1.12E-04
ENSRNOG00000006444	Fkbp4	0.046	-0.467	-1.076	0.455	0.808	1.12E-04
ENSRNOG00000006248	Trim37	0.098	0.865	1.469	-1.085	-1.282	1.13E-04
ENSRNOG00000012660	Postn	1.242	0.942	2.760	-1.615	-2.630	1.13E-04
ENSRNOG00000028356	Chchd10	-0.206	-0.477	-1.433	0.647	1.255	1.14E-04
ENSRNOG00000057042	Pcca	-0.118	-0.365	-0.733	0.247	0.687	1.14E-04
ENSRNOG00000059660	AABR07065531.5	0.236	0.014	1.229	-0.396	-2.068	1.14E-04
ENSRNOG00000057367	Glud1	-0.095	-0.204	-0.405	0.189	0.443	1.17E-04
ENSRNOG00000018552	Slc25a38	0.318	-0.254	-0.779	0.307	0.682	1.24E-04
ENSRNOG00000006450	Erbb2	-0.157	1.129	2.552	-1.913	-2.442	1.25E-04
ENSRNOG00000053537	AABR07070131.1	-0.184	0.222	1.514	-0.378	-1.845	1.28E-04
ENSRNOG00000045553	Proser2	0.008	-0.472	-1.287	0.581	1.060	1.37E-04
ENSRNOG00000011394	Kif3c	-0.109	0.892	1.292	-0.745	-1.129	1.39E-04
ENSRNOG00000031364	Kif1c	0.150	-0.298	-0.880	0.325	0.728	1.44E-04
ENSRNOG00000061429	Rph3al	-0.053	-0.427	-0.981	0.517	0.909	1.59E-04
ENSRNOG00000053691	Lama5	-0.064	0.643	1.170	-0.685	-1.013	1.64E-04

ENSRNOG00000056257	Cacng7	0.327	-0.458	-1.875	0.478	1.514	1.67E-04
ENSRNOG00000004726	Mapkapk2	-0.147	-0.305	-0.732	0.247	0.550	1.67E-04
ENSRNOG00000031275	AABR07054361.1	0.192	0.216	0.556	-0.328	-0.807	1.67E-04
ENSRNOG00000000165	Pfkfb1	-0.154	-0.769	-1.755	0.609	1.510	1.69E-04
ENSRNOG00000000168	Gatm	-0.083	0.166	0.989	-0.245	-0.863	1.70E-04
ENSRNOG00000016097	Ccbl1	-0.130	-0.343	-0.894	0.211	0.704	1.72E-04
ENSRNOG00000060550	Epn2	0.049	-0.096	-0.385	0.166	0.455	1.73E-04
ENSRNOG00000029637	Acot6	-0.221	2.106	4.771	-2.222	-4.853	1.84E-04
ENSRNOG00000022373	Dennd4b	-0.109	-0.259	-0.781	0.326	0.722	1.87E-04
ENSRNOG00000000503	Ppard	0.073	-0.122	-0.427	0.255	0.601	1.87E-04
ENSRNOG00000050035	Sall4	-0.251	-0.298	-1.541	0.537	1.559	1.96E-04
ENSRNOG00000048195	Nudt13	-0.263	-0.240	-0.770	0.207	0.716	1.99E-04
ENSRNOG00000050646	Fem1a	-0.161	-0.142	-0.734	0.199	0.684	1.99E-04
ENSRNOG00000020737	Cdc25a	0.015	0.477	1.112	-0.504	-0.913	1.99E-04
ENSRNOG00000020032	Impdh1	-0.142	0.690	1.255	-0.672	-0.974	2.07E-04
ENSRNOG00000013781	Kcnq5	-0.008	0.824	1.187	-0.651	-1.005	2.07E-04
ENSRNOG00000017876	Dnajc21	0.328	-0.520	-1.169	0.463	0.838	2.10E-04
ENSRNOG00000016863	Pnmal2	0.266	-0.257	0.468	-0.675	-1.608	2.14E-04
ENSRNOG00000025502	Arhgef37	-0.044	-0.358	-1.165	0.300	0.911	2.24E-04
ENSRNOG00000010813	Tspan14	-0.058	0.456	0.798	-0.389	-0.701	2.26E-04
ENSRNOG00000004040	Abca8	0.087	0.383	0.815	-0.747	-0.969	2.26E-04
ENSRNOG00000031890	Ncam1	-0.532	1.856	2.803	-1.881	-1.942	2.26E-04
ENSRNOG00000054765	Renbp	0.177	0.506	1.110	-0.510	-0.843	2.34E-04
ENSRNOG00000042496	Cyp4f17	-0.165	0.546	1.477	-0.647	-1.301	2.37E-04
ENSRNOG00000057988	Pfkm	0.193	-0.494	-1.613	0.498	1.198	2.38E-04
ENSRNOG00000008079	Ugp2	0.135	-0.443	-1.055	0.304	0.893	2.38E-04
ENSRNOG00000021318	Epas1	-0.057	-0.344	-0.900	0.362	0.850	2.38E-04

ENSRNOG00000014882	Fgf11	0.175	0.591	0.820	-0.765	-1.343	2.38E-04
ENSRNOG00000019688	Diaph1	-0.345	-0.161	-0.464	0.264	0.760	2.42E-04
ENSRNOG00000017226	Slc2a4	-0.005	-0.276	-0.897	0.282	0.727	2.59E-04
ENSRNOG00000001890	Txnrd2	-0.152	-0.212	-0.743	0.244	0.663	2.59E-04
ENSRNOG00000046705	Snx3	0.199	-0.258	-0.619	0.376	0.634	2.59E-04
ENSRNOG00000059500	Cdkn1c	-0.175	1.249	2.124	-0.909	-1.418	2.59E-04
ENSRNOG00000009580	Lca5	-0.290	0.760	2.222	-0.870	-2.065	2.59E-04
ENSRNOG00000029571	Coq10a	-0.185	-0.324	-0.862	0.247	0.777	2.87E-04
ENSRNOG00000015962	Nmnat1	-0.011	-0.234	-0.822	0.206	0.582	2.87E-04
ENSRNOG00000003494	Ppfia4	-0.167	0.681	1.064	-0.595	-1.030	2.87E-04
ENSRNOG00000008173	Sesn3	0.111	0.482	1.011	-0.661	-1.016	2.88E-04
ENSRNOG00000022691	St8sia5	0.212	-0.728	-2.116	0.737	1.516	2.93E-04
ENSRNOG00000047218	Clic5	-0.013	-0.196	-0.740	0.146	0.677	2.93E-04
ENSRNOG00000018985	Adrbk1	0.056	-0.199	-0.455	0.206	0.398	2.95E-04
ENSRNOG00000020783	Ntf4	0.089	-0.787	-1.409	0.670	1.140	2.99E-04
ENSRNOG00000021128	Kcnj11	0.255	-0.336	-1.157	0.360	0.805	2.99E-04
ENSRNOG00000019752	Slc29a1	-0.142	0.744	1.375	-0.664	-1.138	3.00E-04
ENSRNOG00000013040	Ddx31	0.156	0.232	0.601	-0.351	-0.532	3.02E-04
ENSRNOG00000001825	Pkp2	0.014	-0.264	-0.829	0.322	0.735	3.02E-04
ENSRNOG00000025324	Spire1	-0.031	0.229	0.996	-0.511	-1.005	3.07E-04
ENSRNOG00000004093	Rhot1	-0.088	0.160	0.589	-0.209	-0.549	3.14E-04
ENSRNOG00000023162	Car14	-0.151	-0.313	-0.932	0.287	0.844	3.16E-04
ENSRNOG00000020605	Pstk	-0.097	-0.300	-0.731	0.251	0.612	3.24E-04
ENSRNOG00000007632	Mss51	0.813	-0.641	-2.166	0.633	1.619	3.41E-04
ENSRNOG00000050630	RGD1565368	0.285	-0.232	-1.297	0.290	1.019	3.45E-04
ENSRNOG00000011145	Crhr2	0.498	0.742	1.546	-0.676	-1.576	3.45E-04
ENSRNOG00000052831	AABR07040629.1	0.737	0.822	1.456	-0.717	-1.683	3.45E-04

ENSRNOG00000025781	Sec31b	0.104	-0.282	-1.233	0.310	0.909	3.60E-04
ENSRNOG00000046439	Vps37c	-0.029	0.558	0.906	-0.482	-0.742	3.62E-04
ENSRNOG00000004820	Sar1b	0.434	-0.595	-1.481	0.512	1.115	3.63E-04
ENSRNOG00000001431	Rasa4	-0.054	-0.165	-0.705	0.348	0.746	3.63E-04
ENSRNOG00000007541	Fhl3	0.019	-0.208	-1.118	0.378	1.057	3.64E-04
ENSRNOG00000052236	AABR07004992.1	0.078	-0.388	-1.068	0.334	0.802	3.64E-04
ENSRNOG00000002418	Tgfb2	-0.107	0.589	0.848	-0.743	-0.978	3.64E-04
ENSRNOG00000060021	Txlnb	-0.034	-0.287	-0.855	0.261	0.728	3.81E-04
ENSRNOG00000000974	Zfp358	0.303	-0.405	-1.165	0.370	0.848	3.82E-04
ENSRNOG00000020372	Hdac4	-0.087	1.356	1.610	-1.271	-1.262	3.82E-04
ENSRNOG00000001130	Nos1	0.194	-0.305	-2.006	0.330	1.598	3.92E-04
ENSRNOG00000001422	Col26a1	1.093	0.652	2.009	-1.761	-2.677	3.93E-04
ENSRNOG00000007323	Ric8b	-0.035	-0.163	-0.544	0.180	0.506	3.93E-04
ENSRNOG00000011306	Myod1	0.487	0.582	1.281	-0.477	-1.402	3.93E-04
ENSRNOG00000048651	Nrtn	-0.916	-0.873	-1.708	1.453	2.184	4.14E-04
ENSRNOG00000012536	Sgms1	0.178	-0.277	-0.796	0.217	0.721	4.14E-04
ENSRNOG00000055226	Dab2ip	0.056	-0.139	-0.757	0.134	0.603	4.14E-04
ENSRNOG00000011543	Fam118b	-0.208	-0.332	-0.584	0.265	0.537	4.14E-04
ENSRNOG00000012601	Leprotl1	0.124	0.053	0.331	-0.197	-0.435	4.14E-04
ENSRNOG00000015192	Mrps5	-0.107	-0.214	-0.623	0.226	0.562	4.24E-04
ENSRNOG00000003388	Cenpf	0.700	0.925	1.932	-1.649	-2.557	4.36E-04
ENSRNOG00000020812	Gys1	-0.164	-0.312	-0.972	0.262	0.804	4.46E-04
ENSRNOG00000037247	Rras	0.009	0.286	0.867	-0.207	-0.670	4.70E-04
ENSRNOG00000008362	Zfp775	-0.158	-0.136	-0.566	0.223	0.526	4.74E-04
ENSRNOG00000003583	Smyd2	0.443	-0.413	-1.141	0.482	0.814	5.07E-04
ENSRNOG00000004757	Tmem158	-0.319	3.298	4.895	-2.760	-3.445	5.07E-04
ENSRNOG00000016043	Aqp4	0.357	-1.316	-2.065	1.250	1.994	5.11E-04

ENSRNOG00000020308	Ech1	-0.221	-0.296	-0.454	0.351	0.823	5.64E-04
ENSRNOG00000018404	Aars	-0.048	-0.135	-0.512	0.158	0.459	5.84E-04
ENSRNOG00000003847	Gid4	0.178	-0.361	-0.886	0.242	0.584	5.85E-04
ENSRNOG00000052398	AABR07042802.1	0.627	0.267	2.505	-1.100	-3.529	5.85E-04
ENSRNOG00000013057	Prc1	0.581	0.720	1.919	-1.350	-2.302	5.88E-04
ENSRNOG00000014119	Farsb	-0.062	0.540	0.963	-0.542	-0.849	6.00E-04
ENSRNOG00000016010	Mul1	0.066	-0.320	-0.908	0.293	0.616	6.04E-04
ENSRNOG00000010257	Cuedc1	0.166	-0.235	-0.849	0.286	0.666	6.06E-04
ENSRNOG00000019290	Pskh1	0.032	-0.145	-0.670	0.186	0.533	6.06E-04
ENSRNOG00000018106	Neu3	0.143	-0.512	-1.169	0.427	0.879	6.12E-04
ENSRNOG00000020027	Arhgef2	-0.013	0.382	0.986	-0.323	-0.712	6.19E-04
ENSRNOG00000007896	Klhl38	0.006	-0.345	-1.082	0.241	0.884	6.22E-04
ENSRNOG00000009425	Fgf7	0.278	0.623	1.304	-0.659	-1.175	6.22E-04
ENSRNOG00000054978	Hist1h1c	0.123	0.079	0.399	-0.309	-0.586	6.25E-04
ENSRNOG00000028910	RGD1565536	-0.215	0.061	-0.471	0.082	0.680	6.44E-04
ENSRNOG00000020828	Pde4a	-0.040	-0.598	-1.704	0.649	1.200	6.50E-04
ENSRNOG00000011622	Echdc1	-0.206	-0.230	-0.512	0.306	0.618	6.69E-04
ENSRNOG00000004377	Lpin1	0.022	-0.499	-1.685	0.345	1.208	6.86E-04
ENSRNOG00000019482	Gnao1	-0.294	-0.143	-0.848	0.289	0.991	6.86E-04
ENSRNOG00000057683	AABR07027212.1	-0.086	-0.326	-0.898	0.235	0.922	6.86E-04
ENSRNOG00000009536	Pgp	-0.050	-0.409	-1.282	0.307	0.921	6.86E-04
ENSRNOG00000019036	Ldhd	-0.149	-0.377	-0.981	0.347	0.890	6.86E-04
ENSRNOG00000048025	Cstf2	-0.109	0.181	0.542	-0.298	-0.627	6.86E-04
ENSRNOG00000055648	Haus7	0.010	0.231	0.614	-0.221	-0.671	6.86E-04
ENSRNOG00000011488	Sergef	-0.148	0.497	1.025	-0.404	-1.039	6.86E-04
ENSRNOG00000033567	Musk	-0.246	2.006	2.772	-1.575	-1.969	6.86E-04
ENSRNOG00000055171	AABR07048040.1	-0.203	0.935	3.653	-0.301	-3.117	6.86E-04

ENSRNOG00000015911	Lrp5	-0.093	-0.099	-0.550	0.283	0.657	6.93E-04
ENSRNOG00000016684	Wnk2	-0.243	-0.032	-0.877	0.133	0.907	6.95E-04
ENSRNOG00000003310	Tmem63a	-0.114	-0.003	-0.199	0.069	0.470	7.14E-04
ENSRNOG00000017123	B2m	-0.130	0.792	1.530	-0.523	-0.835	7.17E-04
ENSRNOG00000009045	Alg6	0.075	0.550	1.145	-0.785	-1.044	7.17E-04
ENSRNOG00000001584	Map3k7cl	-0.453	0.468	1.260	-0.519	-1.418	7.44E-04
ENSRNOG00000031431	Cdca8	0.488	0.594	1.109	-1.016	-1.392	7.78E-04
ENSRNOG00000003302	Flcn	-0.046	-0.220	-0.660	0.297	0.533	7.78E-04
ENSRNOG00000033090	Ltbp1	0.309	0.857	1.469	-0.600	-1.025	7.86E-04
ENSRNOG00000000774	Gabbr1	-0.073	0.366	0.549	-0.354	-0.626	7.89E-04
ENSRNOG00000005464	Lgalsl	0.201	-0.489	-1.038	0.338	0.723	7.96E-04
ENSRNOG00000004542	Ttc8	0.035	0.009	0.858	-0.325	-1.009	7.96E-04
ENSRNOG00000051306	Rn60_1_2682.1	-0.183	-0.369	-0.853	0.309	0.849	7.98E-04
ENSRNOG00000020607	Bckdha	-0.350	-0.230	-0.552	0.322	0.676	7.98E-04
ENSRNOG00000043199	Bves	0.295	0.342	0.320	-0.393	-0.537	7.98E-04
ENSRNOG00000014698	Chrnb1	0.166	0.444	0.515	-0.375	-0.619	7.98E-04
ENSRNOG00000011631	Fst	-0.183	1.257	2.298	-1.108	-1.799	7.98E-04
ENSRNOG00000022957	Ctxn3	-0.658	3.630	4.423	-3.335	-3.111	7.98E-04
ENSRNOG00000008911	Draxin	0.424	2.364	4.024	-2.640	-4.127	7.98E-04
ENSRNOG00000007069	Adhfe1	0.320	-0.346	-1.072	0.267	1.042	8.11E-04
ENSRNOG00000027622	Slc35e3	0.068	0.391	0.867	-0.386	-0.613	8.14E-04
ENSRNOG00000006365	Asb15	-0.298	-0.518	-1.400	0.448	1.047	8.16E-04
ENSRNOG00000027434	Fitm2	-0.229	-0.700	-1.315	0.537	1.022	8.22E-04
ENSRNOG00000016763	Fgfr4	0.404	0.412	0.813	-0.470	-0.856	8.22E-04
ENSRNOG00000038883	LOC294154	0.120	-0.387	-0.945	0.319	0.629	8.29E-04
ENSRNOG00000004162	Pfkfb2	-0.402	-0.205	-0.451	0.257	0.650	8.30E-04
ENSRNOG00000059659	Zfand3	0.300	-0.329	-0.784	0.377	0.527	8.30E-04

ENSRNOG00000009884	Lgals1	-0.002	0.534	0.932	-0.420	-0.865	8.50E-04
ENSRNOG00000059957	AABR07021734.2	0.176	0.170	0.553	-0.288	-0.821	8.54E-04
ENSRNOG00000014718	Acsl3	0.381	0.343	0.568	-0.410	-1.013	8.66E-04
ENSRNOG00000004537	Rxrg	0.198	-0.458	-1.649	0.443	1.060	8.86E-04
ENSRNOG00000027731	Ubxn10	0.060	-0.973	-2.583	0.866	2.307	8.92E-04
ENSRNOG00000017637	Fbp2	0.241	-0.281	-1.453	0.466	1.336	9.08E-04
ENSRNOG00000046280	Tceal5	0.741	0.820	1.274	-0.718	-1.396	9.08E-04
ENSRNOG00000003563	Blmh	0.051	0.074	0.243	-0.124	-0.368	9.17E-04
ENSRNOG00000026143	Ckap2l	0.718	0.310	1.519	-1.461	-2.684	9.19E-04
ENSRNOG00000022727	Tmem127	-0.037	0.214	0.444	-0.258	-0.463	9.23E-04
ENSRNOG00000017233	Mmachc	-0.244	-0.274	-0.710	0.207	0.621	9.28E-04
ENSRNOG00000053047	Top2a	0.155	0.908	1.865	-1.033	-2.137	9.28E-04
ENSRNOG00000005960	RGD1311744	0.132	0.706	1.899	-0.958	-1.465	9.35E-04
ENSRNOG00000025518	Lrrc16b	-0.955	-0.106	-0.830	0.782	1.702	9.40E-04
ENSRNOG00000012481	Ppm1j	-0.094	-0.354	-1.097	0.286	0.792	9.40E-04
ENSRNOG00000011589	Camk2d	0.565	0.172	0.327	-0.380	-0.772	9.40E-04
ENSRNOG00000029336	Zfp180	0.116	-0.348	-0.793	0.378	0.693	9.48E-04
ENSRNOG00000001769	Tnk2	0.041	-0.304	-1.152	0.455	0.991	9.48E-04
ENSRNOG00000055524	Trip10	-0.005	0.009	-0.378	0.138	0.473	9.59E-04
ENSRNOG00000013452	Rcn1	-0.048	0.418	1.095	-0.545	-0.846	9.60E-04
ENSRNOG00000002026	Dnajc28	-0.105	-0.349	-0.821	0.239	0.742	9.62E-04
ENSRNOG00000012524	Zfp91	0.059	-0.474	-0.889	0.375	0.648	9.64E-04
ENSRNOG00000002516	Mtm1	-0.037	-0.094	-0.457	0.109	0.425	9.82E-04
ENSRNOG00000020244	Perm1	-0.487	-0.672	-1.614	0.602	1.503	9.86E-04
ENSRNOG00000016829	Isoc2b	-0.271	-0.497	-0.914	0.416	0.862	9.99E-04
ENSRNOG00000012868	Uaca	0.096	-0.103	-0.569	0.165	0.558	1.01E-03
ENSRNOG00000059013	AABR07060519.1	0.169	0.171	-0.572	0.678	1.058	1.01E-03

ENSRNOG00000038597	Dlg1	0.115	-0.197	-0.629	0.256	0.594	1.01E-03
ENSRNOG00000001055	Rilpl1	-0.019	-0.357	-0.807	0.348	0.579	1.01E-03
ENSRNOG00000052343	Eif4e	-0.079	-0.265	-0.555	0.182	0.493	1.01E-03
ENSRNOG00000032664	RT1-O1	1.609	3.001	3.829	-2.568	-3.094	1.01E-03
ENSRNOG00000042536	Pde4d	-0.032	-0.317	-1.343	0.291	1.157	1.02E-03
ENSRNOG00000017752	Mccc2	-0.350	-0.153	-0.615	0.107	0.559	1.02E-03
ENSRNOG00000016090	Mtmr10	-0.035	-0.323	-0.551	0.235	0.475	1.03E-03
ENSRNOG00000060154	Ddx23	0.200	-0.283	-0.775	0.363	0.588	1.06E-03
ENSRNOG00000015986	Rassf8	-0.103	-0.202	-0.652	0.190	0.500	1.06E-03
ENSRNOG00000016054	Tab2	0.143	-0.370	-0.842	0.255	0.563	1.06E-03
ENSRNOG00000019645	Osbp2	-0.499	-0.118	-0.653	0.314	1.132	1.07E-03
ENSRNOG00000017117	Ybx2	0.090	-0.819	-1.483	0.802	1.089	1.07E-03
ENSRNOG00000011603	Cab39l	0.161	-0.334	-0.654	0.176	0.501	1.08E-03
ENSRNOG00000048169	Tuba8	-0.002	-0.366	-1.675	0.475	1.139	1.09E-03
ENSRNOG00000000954	Rpo1-3	0.135	0.226	0.598	-0.298	-0.665	1.09E-03
ENSRNOG00000022392	Hspb8	-0.030	0.560	1.089	-0.510	-0.922	1.10E-03
ENSRNOG00000015029	Dbt	-0.059	-0.099	-0.471	0.103	0.623	1.10E-03
ENSRNOG00000029441	Klhl2	-0.053	0.449	1.252	-0.444	-0.966	1.11E-03
ENSRNOG00000042915	Mxra7	-0.384	0.789	1.988	-1.013	-1.645	1.11E-03
ENSRNOG00000005916	Ppm1a	0.149	-0.332	-0.532	0.256	0.509	1.15E-03
ENSRNOG00000021025	Ppp2r5b	-0.039	-0.173	-0.446	0.241	0.441	1.15E-03
ENSRNOG00000017816	Pde6a	-0.216	2.659	4.313	-2.658	-3.957	1.16E-03
ENSRNOG00000002840	Atp5b	-0.227	-0.285	-0.780	0.276	0.687	1.17E-03
ENSRNOG00000025145	Rmdn1	-0.252	-0.466	-0.857	0.383	0.756	1.19E-03
ENSRNOG00000033361	Slc39a5	-0.203	-0.227	-0.709	0.172	0.698	1.19E-03
ENSRNOG00000010725	Cpa1	0.329	0.018	0.559	-0.553	-1.197	1.19E-03
ENSRNOG00000059840	Unc5a	0.719	-0.153	-1.639	0.622	1.723	1.19E-03

ENSRNOG00000014130	LOC100362620	0.651	1.567	2.391	-1.460	-1.937	1.21E-03
ENSRNOG00000006802	Lrrn1	0.146	0.280	1.662	-0.740	-1.797	1.22E-03
ENSRNOG00000054063	LOC100910872	-0.249	-0.185	-0.510	0.232	0.567	1.22E-03
ENSRNOG00000015225	Gramd3	-0.114	0.396	0.917	-0.388	-0.759	1.22E-03
ENSRNOG00000019048	Sod2	-0.301	-0.302	-0.742	0.282	0.627	1.24E-03
ENSRNOG00000004534	Spdya	-0.159	-0.326	-1.050	0.491	1.048	1.25E-03
ENSRNOG00000011648	Aqp1	-0.123	-0.318	-0.586	0.368	0.709	1.25E-03
ENSRNOG00000004834	Llgl2	-0.111	0.010	-1.104	0.254	1.069	1.25E-03
ENSRNOG00000037957	Aifm3	-0.187	0.029	-0.700	0.664	1.176	1.27E-03
ENSRNOG00000012091	Ppa2	-0.166	-0.437	-0.817	0.375	0.650	1.27E-03
ENSRNOG00000028357	Lrrc14b	0.137	-0.288	-0.992	0.253	0.638	1.27E-03
ENSRNOG00000049531	Usp19	-0.054	-0.191	-0.677	0.197	0.477	1.27E-03
ENSRNOG00000006865	Laptm4a	0.155	0.060	0.256	-0.108	-0.331	1.27E-03
ENSRNOG00000032929	Incenp	0.166	0.101	0.196	-0.162	-0.787	1.27E-03
ENSRNOG00000015582	Me2	-0.062	0.455	1.333	-0.310	-0.871	1.27E-03
ENSRNOG00000007483	Ccnf	0.639	0.935	1.861	-1.416	-2.585	1.27E-03
ENSRNOG00000024128	Aco2	-0.152	-0.269	-0.864	0.208	0.689	1.27E-03
ENSRNOG00000014368	Eif4g3	-0.014	-0.087	-0.454	0.127	0.496	1.27E-03
ENSRNOG00000030515	Nfasc	-0.683	-0.030	-1.093	0.594	1.308	1.28E-03
ENSRNOG00000010543	Srpr	-0.136	-0.177	-0.634	0.180	0.462	1.28E-03
ENSRNOG00000030225	Clpx	-0.249	-0.262	-0.454	0.188	0.579	1.32E-03
ENSRNOG00000053904	AABR07022113.1	-0.135	-0.991	-3.538	0.835	2.403	1.32E-03
ENSRNOG00000008592	Barx2	-0.222	0.149	-0.990	0.660	1.361	1.32E-03
ENSRNOG00000025415	Mettl11b	-0.844	-0.421	-1.299	0.400	1.171	1.32E-03
ENSRNOG00000007133	Plxnb2	0.237	0.313	0.534	-0.311	-0.553	1.32E-03
ENSRNOG00000018841	Sox8	0.321	-0.051	1.063	-0.546	-2.079	1.32E-03
ENSRNOG00000033335	Cenpi	-0.218	-0.044	1.142	0.099	-2.206	1.32E-03

ENSRNOG00000061934	AABR07044836.1	0.386	1.673	3.887	-1.363	-3.524	1.32E-03
ENSRNOG00000030963	LOC685186	0.300	-0.266	-1.250	0.277	0.940	1.33E-03
ENSRNOG00000016484	Gstk1	-0.135	-0.229	-0.536	0.278	0.632	1.33E-03
ENSRNOG00000007164	Cln6	0.220	0.005	0.329	-0.353	-0.657	1.33E-03
ENSRNOG00000011448	Eri1	0.104	0.126	0.512	-0.231	-0.560	1.35E-03
ENSRNOG00000022699	Rn60_10_0644.1	0.161	1.501	3.278	-1.218	-3.217	1.36E-03
ENSRNOG00000009427	Tbx21	1.055	0.994	1.416	-0.999	-1.738	1.38E-03
ENSRNOG00000000632	Cdk1	0.669	1.065	2.292	-1.394	-2.469	1.38E-03
ENSRNOG00000013211	Chchd3	0.010	-0.481	-0.906	0.414	0.694	1.38E-03
ENSRNOG00000000047	Cd82	-0.049	0.424	1.156	-0.272	-0.761	1.39E-03
ENSRNOG00000014948	Osgin1	-0.102	-0.104	-0.508	0.176	1.152	1.41E-03
ENSRNOG00000042634	Serpina12	0.383	2.849	4.219	-1.985	-2.692	1.41E-03
ENSRNOG00000027383	Sntn	0.313	1.653	3.614	-2.150	-3.481	1.44E-03
ENSRNOG00000010415	Atxn7l4	0.045	-0.290	-0.722	0.275	0.548	1.44E-03
ENSRNOG00000019181	Synpo	0.016	-0.376	-0.943	0.303	0.839	1.45E-03
ENSRNOG00000012759	Col19a1	0.265	2.176	3.839	-2.876	-3.567	1.47E-03
ENSRNOG00000008056	Ankrd9	0.167	-0.413	-1.684	0.462	1.142	1.47E-03
ENSRNOG00000020202	Asrgl1	0.392	-0.299	-0.723	0.269	0.610	1.52E-03
ENSRNOG00000053468	Tuba1b	-0.044	0.266	1.008	-0.340	-0.778	1.52E-03
ENSRNOG00000050473	Rps27l	0.361	0.331	0.721	-0.348	-0.836	1.52E-03
ENSRNOG00000019372	Pc	-0.056	-0.209	-0.906	0.316	0.748	1.53E-03
ENSRNOG00000004640	Mtfp1	-0.312	-0.411	-1.204	0.307	0.977	1.56E-03
ENSRNOG00000015829	Trpm1	0.079	-0.233	-1.910	0.574	1.489	1.56E-03
ENSRNOG00000000920	Phkg1	0.229	-0.336	-1.579	0.383	1.119	1.57E-03
ENSRNOG00000020371	Pgap2	0.007	0.173	0.365	-0.208	-0.379	1.57E-03
ENSRNOG00000034190	Ighm	-0.192	0.604	1.837	-0.507	-1.576	1.57E-03
ENSRNOG00000038366	Larp1b	0.005	-0.273	-0.759	0.183	0.520	1.58E-03

ENSRNOG00000022922	Slc25a12	-0.144	-0.328	-0.961	0.235	0.751	1.58E-03
ENSRNOG00000006068	Tmem117	0.176	0.091	0.311	-0.151	-0.587	1.58E-03
ENSRNOG00000003802	Pttg1	0.587	0.430	1.573	-0.568	-2.269	1.58E-03
ENSRNOG00000018630	Gapdh	0.277	-0.245	-1.188	0.269	0.915	1.58E-03
ENSRNOG00000012014	Fam160b2	-0.087	-0.227	-0.832	0.214	0.611	1.58E-03
ENSRNOG00000006030	Ptprz1	0.385	0.044	0.638	-0.731	-1.497	1.58E-03
ENSRNOG00000061851	Foxj3	-0.044	-0.255	-0.610	0.170	0.452	1.66E-03
ENSRNOG00000011425	Ptpn3	-0.501	-0.466	-1.168	0.463	1.181	1.68E-03
ENSRNOG00000010800	Hadhb	-0.391	-0.312	-0.417	0.300	0.735	1.68E-03
ENSRNOG00000000921	Ubl3	-0.038	-0.148	-0.264	0.116	0.305	1.68E-03
ENSRNOG00000022533	Micall2	0.012	0.913	1.894	-0.762	-1.526	1.68E-03
ENSRNOG00000059224	Reps1	-0.162	0.169	0.712	-0.133	-0.635	1.70E-03
ENSRNOG00000058003	Spon1	0.313	0.425	1.477	-0.811	-1.493	1.70E-03
ENSRNOG00000016292	Pdzd9	-0.245	-0.526	-0.989	0.345	0.876	1.71E-03
ENSRNOG00000016338	Fam92a1	0.236	-0.576	0.023	-0.435	-0.843	1.72E-03
ENSRNOG00000049033	Racgap1	0.355	1.048	1.780	-1.166	-2.769	1.72E-03
ENSRNOG00000020857	Ppfia1	-0.246	-0.138	-0.382	0.215	0.519	1.73E-03
ENSRNOG00000057284	Cenpb	0.017	-0.199	-0.434	0.223	0.384	1.73E-03
ENSRNOG00000004518	Cacnb1	0.301	0.383	0.526	-0.288	-0.740	1.74E-03
ENSRNOG00000017146	RGD1560225	-0.097	-0.168	-0.776	0.233	0.640	1.74E-03
ENSRNOG00000019930	Rhot2	-0.150	-0.119	-0.355	0.147	0.405	1.75E-03
ENSRNOG00000005877	Lrpprc	-0.198	-0.268	-0.674	0.205	0.616	1.76E-03
ENSRNOG00000011659	Alpk3	-0.092	-0.261	-0.705	0.190	0.605	1.76E-03
ENSRNOG00000020706	Kcnn3	-0.148	1.296	1.773	-1.048	-1.107	1.77E-03
ENSRNOG00000007955	Timp4	0.015	-0.348	-0.775	0.511	0.989	1.77E-03
ENSRNOG00000015180	Kdm8	0.056	-0.251	-0.679	0.220	0.511	1.77E-03
ENSRNOG00000016364	Gba2	-0.228	-0.249	-0.422	0.347	0.510	1.78E-03

ENSRNOG00000018415	Acot13	-0.148	-0.460	-1.019	0.378	0.824	1.81E-03
ENSRNOG00000014050	Ptges2	-0.174	-0.269	-0.718	0.280	0.589	1.82E-03
ENSRNOG00000054479	Zw10	0.255	0.352	0.633	-0.326	-0.496	1.82E-03
ENSRNOG00000028623	Agpat5	0.226	0.136	0.305	-0.349	-0.490	1.83E-03
ENSRNOG00000043128	Armcx4	-0.143	0.075	0.922	-0.197	-0.814	1.83E-03
ENSRNOG00000013961	Ghitm	-0.177	-0.256	-0.463	0.246	0.497	1.84E-03
ENSRNOG00000012051	Ncaph	0.158	0.106	1.581	-0.177	-2.175	1.84E-03
ENSRNOG00000052354	Arhgef40	-0.024	0.153	0.574	-0.216	-0.580	1.87E-03
ENSRNOG00000003334	Klhl21	0.264	-0.309	-1.018	0.345	0.742	1.88E-03
ENSRNOG00000001653	St3gal6	0.335	-0.542	-1.027	0.328	0.696	1.88E-03
ENSRNOG00000015774	Mreg	0.302	-0.256	-0.623	0.334	0.606	1.88E-03
ENSRNOG00000056216	Casp7	0.001	-0.297	-0.590	0.352	0.498	1.88E-03
ENSRNOG00000010873	Pithd1	-0.198	-0.134	-0.370	0.233	0.475	1.88E-03
ENSRNOG00000016150	Ifrd2	0.124	-0.136	-0.568	0.276	0.462	1.88E-03
ENSRNOG00000046227	Tprg1l	0.058	0.215	0.545	-0.203	-0.478	1.88E-03
ENSRNOG00000018286	Chrna1	-0.832	2.549	4.148	-2.599	-2.959	1.88E-03
ENSRNOG00000011881	Slc25a15	-0.192	-0.272	-0.431	0.388	0.588	1.88E-03
ENSRNOG00000002271	Slain2	0.003	0.450	0.726	-0.516	-0.629	1.88E-03
ENSRNOG00000057072	Slc12a3	-0.025	3.254	4.393	-3.891	-2.900	1.89E-03
ENSRNOG00000009421	Ivd	-0.139	-0.240	-0.518	0.223	0.618	1.91E-03
ENSRNOG00000003841	Kcnh1	-1.062	0.432	-1.489	1.273	2.206	1.93E-03
ENSRNOG00000021096	Tmem143	-0.211	-0.237	-0.668	0.278	0.614	1.94E-03
ENSRNOG00000019791	Sipa1l2	-0.055	-0.453	-0.944	0.373	0.772	1.94E-03
ENSRNOG00000053270	AABR07053741.1	-0.176	-0.292	-0.452	0.252	0.510	1.94E-03
ENSRNOG00000060728	Tuba1a	0.039	0.016	0.694	-0.107	-0.741	1.97E-03
ENSRNOG00000025997	Mrrf	-0.216	-0.075	-0.224	0.106	0.355	1.97E-03
ENSRNOG00000016695	Mmp2	0.298	0.283	0.991	-0.406	-0.942	1.97E-03

ENSRNOG00000052664	LOC100910717	-0.130	-0.398	-0.914	0.265	0.670	2.04E-03
ENSRNOG00000020596	Atp5sl	-0.091	-0.155	-0.460	0.149	0.419	2.05E-03
ENSRNOG00000028812	Trim80	0.058	2.420	3.985	-2.324	-3.662	2.07E-03
ENSRNOG00000056697	Kcnab1	0.803	0.276	0.858	-0.268	-1.449	2.09E-03
ENSRNOG00000038140	Whsc1	0.075	0.099	0.395	-0.432	-0.506	2.10E-03
ENSRNOG00000050675	Myl4	0.269	0.536	0.924	-0.445	-1.126	2.11E-03
ENSRNOG00000001710	Abcf3	-0.130	-0.179	-0.471	0.189	0.397	2.13E-03
ENSRNOG00000017993	Abcb10	-0.176	-0.159	-0.529	0.139	0.419	2.14E-03
ENSRNOG00000016509	Usp10	0.000	-0.140	-0.466	0.151	0.369	2.20E-03
ENSRNOG00000003242	Gulp1	-0.272	0.320	0.993	-0.395	-0.846	2.20E-03
ENSRNOG00000020164	Ifitm10	0.389	0.941	1.339	-0.694	-1.223	2.20E-03
ENSRNOG00000042344	Smim22	0.594	0.806	2.170	-0.940	-2.250	2.20E-03
ENSRNOG00000037801	LOC100360238	-0.649	-0.347	-1.251	-0.337	1.542	2.21E-03
ENSRNOG00000007459	Pcnx	-0.037	-0.013	-0.393	0.203	0.553	2.23E-03
ENSRNOG00000000804	Mrps18b	-0.117	-0.245	-0.481	0.317	0.513	2.23E-03
ENSRNOG00000004595	Pou6f1	-0.055	0.097	-0.382	0.297	0.566	2.24E-03
ENSRNOG00000038625	Sbk2	-0.690	-0.870	-1.756	0.791	1.834	2.27E-03
ENSRNOG00000005987	Suox	-0.221	-0.435	-0.590	0.367	0.708	2.27E-03
ENSRNOG00000008797	Tmpo	0.029	-0.178	-0.464	0.221	0.449	2.28E-03
ENSRNOG00000025167	Sema4b	0.122	0.384	0.844	-0.357	-0.680	2.30E-03
ENSRNOG00000004471	Polr3h	0.242	0.302	0.647	-0.111	-0.566	2.32E-03
ENSRNOG00000025295	Mavs	-0.567	-0.376	-0.803	0.377	0.823	2.32E-03
ENSRNOG00000019063	Fbxo38	0.070	0.160	0.226	-0.199	-0.317	2.33E-03
ENSRNOG00000009289	Galk2	-0.009	0.219	0.620	-0.216	-0.600	2.33E-03
ENSRNOG00000009863	Prrt3	0.431	0.766	1.638	-0.343	-1.411	2.33E-03
ENSRNOG00000005064	Ube2b	0.079	-0.182	-0.426	0.174	0.412	2.34E-03
ENSRNOG00000003777	Chrne	0.387	0.735	1.347	-1.405	-1.547	2.34E-03

ENSRNOG00000009431	Tbc1d4	-0.099	-0.401	-1.240	0.349	1.015	2.37E-03
ENSRNOG00000001276	Pcnt	-0.158	-0.150	-0.679	0.227	0.697	2.38E-03
ENSRNOG00000008040	Fam64a	1.122	0.744	2.633	-1.869	-3.163	2.41E-03
ENSRNOG00000000321	Cd24	0.560	-0.369	-1.421	0.444	1.188	2.44E-03
ENSRNOG00000015904	Wfdc1	0.051	-0.394	-1.309	0.358	0.819	2.45E-03
ENSRNOG00000000657	Nek7	0.119	-0.232	-0.743	0.270	0.545	2.46E-03
ENSRNOG00000032443	Lmod3	-0.122	-0.608	-0.966	0.462	0.677	2.47E-03
ENSRNOG00000030869	Aldoart2	0.379	-0.585	-1.557	0.654	1.307	2.48E-03
ENSRNOG00000008086	Dpf3	-0.004	-0.272	-1.155	0.265	0.852	2.49E-03
ENSRNOG00000007059	Atp1b4	-0.199	0.579	1.009	-0.732	-1.271	2.50E-03
ENSRNOG00000006305	Slc38a2	-0.058	-0.175	-0.658	0.218	0.731	2.59E-03
ENSRNOG00000010475	Casp3	0.088	0.392	0.918	-0.130	-0.747	2.62E-03
ENSRNOG00000016451	Cd1d1	0.038	-0.401	-1.285	0.463	1.063	2.62E-03
ENSRNOG00000009819	Vezf1	0.168	-0.240	-0.837	0.190	0.578	2.63E-03
ENSRNOG00000019585	Kat8	0.109	-0.222	-0.604	0.247	0.464	2.63E-03
ENSRNOG00000003887	Lgi2	0.163	-0.576	-2.222	0.449	1.728	2.66E-03
ENSRNOG00000005615	Gadd45a	-0.446	2.082	3.227	-1.766	-1.860	2.66E-03
ENSRNOG00000047236	AABR07008066.2	-0.019	-0.440	-1.334	0.271	1.177	2.66E-03
ENSRNOG00000009439	Eef1a1	0.024	0.424	1.372	-0.435	-0.853	2.67E-03
ENSRNOG00000014034	Olfml2a	0.002	0.043	0.685	-0.484	-0.918	2.67E-03
ENSRNOG00000008145	Traf3	0.075	0.163	0.346	-0.405	-0.606	2.69E-03
ENSRNOG00000030245	Tango2	0.014	-0.149	-0.636	0.278	0.632	2.71E-03
ENSRNOG00000056462	Tmf1	0.046	-0.261	-0.546	0.165	0.457	2.74E-03
ENSRNOG00000024795	Zfp862	-0.184	0.660	1.364	-0.689	-1.036	2.74E-03
ENSRNOG00000011588	Rnf146	0.025	-0.193	-0.379	0.123	0.374	2.75E-03
ENSRNOG00000010134	Acot2	-0.418	-0.114	-0.258	0.006	0.711	2.76E-03
ENSRNOG00000021139	Esrra	-0.234	-0.342	-0.793	0.355	0.670	2.76E-03

ENSRNOG00000014044	Pank4	-0.063	-0.225	-0.739	0.275	0.537	2.76E-03
ENSRNOG00000017871	Sidt2	0.042	-0.137	-0.299	0.205	0.385	2.76E-03
ENSRNOG00000007089	Lgmn	-0.055	0.323	0.906	-0.248	-0.580	2.76E-03
ENSRNOG00000013751	Prosc	0.071	-0.182	-0.423	0.157	0.335	2.79E-03
ENSRNOG00000012835	Espl1	-0.425	-0.707	-2.016	0.678	1.346	2.81E-03
ENSRNOG00000006931	Eepd1	0.227	-0.472	-1.620	0.533	1.121	2.81E-03
ENSRNOG00000014030	Synm	0.058	-0.288	-0.986	0.254	0.721	2.81E-03
ENSRNOG00000061468	AABR07042825.1	-0.033	1.864	3.222	-1.703	-2.876	2.81E-03
ENSRNOG00000021090	Pygm	0.244	-0.264	-1.428	0.317	1.042	2.81E-03
ENSRNOG00000031590	AABR07048308.1	0.274	-0.276	-1.208	0.375	1.008	2.81E-03
ENSRNOG00000043037	Zfp770	-0.025	-0.451	-0.757	0.293	0.826	2.81E-03
ENSRNOG00000011422	Atmin	0.016	-0.220	-0.817	0.145	0.613	2.81E-03
ENSRNOG00000013293	Mccc1	-0.104	-0.221	-0.557	0.192	0.529	2.81E-03
ENSRNOG00000025459	Mars	-0.076	-0.070	-0.260	0.147	0.314	2.81E-03
ENSRNOG00000060821	Rnf114	0.033	-0.091	-0.306	0.108	0.276	2.81E-03
ENSRNOG00000018020	Apbb1	0.073	0.399	0.588	-0.156	-0.540	2.81E-03
ENSRNOG00000017911	Tcaf1	-0.071	0.176	0.761	-0.236	-0.792	2.81E-03
ENSRNOG00000043300	LOC100912292	-0.475	1.078	2.092	-0.532	-1.336	2.81E-03
ENSRNOG00000042847	LOC687707	0.478	0.918	1.558	-1.633	-1.796	2.81E-03
ENSRNOG00000002828	Tob1	0.008	-0.346	-0.798	0.392	0.657	2.82E-03
ENSRNOG00000050843	Mut	-0.063	-0.342	-0.713	0.240	0.634	2.82E-03
ENSRNOG00000023452	LOC691354	-0.009	-0.509	-1.249	0.453	0.799	2.82E-03
ENSRNOG00000024972	Cox10	-0.226	-0.318	-0.867	0.223	0.763	2.85E-03
ENSRNOG00000020544	Sdr39u1	-0.151	-0.239	-0.619	0.395	0.619	2.85E-03
ENSRNOG00000048050	Tmem120b	-0.003	-0.062	-0.565	0.160	0.489	2.85E-03
ENSRNOG00000019648	Col6a3	0.054	0.153	0.918	-0.540	-1.035	2.85E-03
ENSRNOG00000001654	Cpox	-0.076	-0.189	-0.462	0.102	0.381	2.86E-03

ENSRNOG00000025000	Cep104	0.005	-0.122	-0.425	0.075	0.347	2.86E-03
ENSRNOG00000011821	S100a4	0.270	0.814	2.314	-1.008	-1.470	2.86E-03
ENSRNOG00000005214	Plek	1.569	0.288	0.493	-0.944	-1.234	2.91E-03
ENSRNOG00000021011	Fut2	0.420	0.907	1.487	-1.554	-1.519	2.93E-03
ENSRNOG00000017369	Mustn1	0.628	1.324	2.327	-1.168	-1.854	2.93E-03
ENSRNOG00000002449	Maged2	-0.126	0.186	0.526	-0.141	-0.536	2.96E-03
ENSRNOG00000010150	Strbp	0.131	-0.301	-0.994	0.281	0.712	3.02E-03
ENSRNOG00000010934	Spa17	0.233	-0.590	-1.279	0.533	0.907	3.02E-03
ENSRNOG00000001770	Ehhadh	0.122	-0.331	-0.665	0.351	0.758	3.02E-03
ENSRNOG00000017250	Gmpr	0.203	-0.268	-0.927	0.275	0.614	3.02E-03
ENSRNOG00000006096	Slc26a7	-0.172	1.236	2.671	-1.295	-3.062	3.02E-03
ENSRNOG00000020182	Mvp	0.105	0.716	0.887	-0.616	-0.793	3.02E-03
ENSRNOG00000001249	Col6a1	0.136	0.145	0.762	-0.413	-0.912	3.05E-03
ENSRNOG00000014935	Peo1	-0.027	-0.198	-0.420	0.169	0.389	3.06E-03
ENSRNOG00000013076	Csnk1e	0.034	-0.091	-0.424	0.176	0.387	3.06E-03
ENSRNOG00000039496	Plp2	0.168	0.423	0.800	-0.404	-0.620	3.06E-03
ENSRNOG00000024605	Fam220a	-0.040	-0.374	-0.937	0.291	0.727	3.07E-03
ENSRNOG00000018047	Gorasp1	0.016	-0.072	-0.504	0.132	0.411	3.07E-03
ENSRNOG00000015380	Jup	0.058	0.315	0.348	-0.242	-0.404	3.07E-03
ENSRNOG00000010664	Wdr73	0.147	0.251	0.498	-0.320	-0.637	3.08E-03
ENSRNOG00000019180	Acsl4	0.111	0.892	1.432	-0.846	-1.121	3.12E-03
ENSRNOG00000025460	Tmem71	0.226	0.970	1.758	-0.638	-1.404	3.12E-03
ENSRNOG00000060120	Selo	-0.076	-0.135	-0.518	0.234	0.457	3.12E-03
ENSRNOG00000014289	Arpc2	0.098	0.107	0.360	-0.182	-0.371	3.12E-03
ENSRNOG00000010553	Pnma1	0.705	1.635	2.377	-1.780	-1.919	3.12E-03
ENSRNOG00000000922	Sumf2	0.253	-0.341	-1.471	0.368	0.991	3.13E-03
ENSRNOG00000018825	Dcaf11	0.046	-0.144	-0.407	0.212	0.318	3.13E-03

ENSRNOG00000000809	Atat1	-0.093	0.373	0.610	-0.297	-0.561	3.13E-03
ENSRNOG00000018681	LOC100910255	0.005	0.300	1.432	-0.607	-1.167	3.13E-03
ENSRNOG00000006328	Wwp1	0.138	-0.398	-0.982	0.311	0.695	3.13E-03
ENSRNOG00000018657	Scrn3	-0.170	-0.365	-0.647	0.148	0.454	3.17E-03
ENSRNOG00000018097	Emc1	-0.052	-0.101	-0.474	0.093	0.415	3.19E-03
ENSRNOG00000018533	Iffo1	-0.074	0.450	0.939	-0.319	-0.609	3.19E-03
ENSRNOG00000001750	Chrd	-0.257	0.864	1.587	-1.075	-1.272	3.19E-03
ENSRNOG00000020644	Nsg2	0.188	0.555	2.135	-1.273	-2.278	3.19E-03
ENSRNOG00000013194	Rps6ka2	0.060	-0.369	-1.209	0.252	0.801	3.20E-03
ENSRNOG00000012663	Mfsd6	0.052	-0.183	-0.945	0.167	0.602	3.20E-03
ENSRNOG00000006985	Ccnk	0.068	-0.142	-0.472	0.190	0.440	3.20E-03
ENSRNOG00000028113	Whamm	-0.069	-0.005	-0.248	0.143	0.428	3.20E-03
ENSRNOG00000006731	Spc25	0.514	0.373	1.686	-0.641	-2.529	3.20E-03
ENSRNOG00000011154	Adgrf5	-0.154	-0.294	-0.549	0.328	0.762	3.22E-03
ENSRNOG00000005371	Klhl29	0.043	0.597	0.961	-0.270	-0.787	3.23E-03
ENSRNOG00000016242	Fzd1	-0.102	0.189	0.727	-0.280	-0.803	3.23E-03
ENSRNOG00000012443	Cpt2	-0.485	-0.331	-0.577	0.185	0.757	3.26E-03
ENSRNOG00000007862	Acat1	-0.380	-0.260	-0.631	0.234	0.602	3.28E-03
ENSRNOG00000008118	Sync	0.192	0.320	0.370	-0.264	-0.570	3.29E-03
ENSRNOG00000040257	Chmp2b	-0.055	0.093	0.389	-0.255	-0.414	3.30E-03
ENSRNOG00000013468	Fam213b	0.285	-0.388	-1.463	0.518	0.997	3.32E-03
ENSRNOG00000006947	Pdhx	-0.187	-0.314	-0.778	0.158	0.530	3.35E-03
ENSRNOG00000017352	Ctdnep1	0.000	-0.240	-0.528	0.252	0.414	3.35E-03
ENSRNOG00000003063	Phka1	0.141	-0.281	-1.616	0.184	1.044	3.36E-03
ENSRNOG00000004229	Tac3	0.215	1.460	2.928	-1.841	-3.515	3.37E-03
ENSRNOG00000017116	Zfp532	0.068	-0.169	-0.662	0.195	0.495	3.38E-03
ENSRNOG00000013618	Ankrd10	-0.159	0.242	0.609	-0.453	-0.563	3.38E-03

ENSRNOG00000042477	Clrn1	1.216	0.459	3.202	-0.171	-3.104	3.42E-03
ENSRNOG00000048704	RGD1562758	0.262	-0.309	-1.014	0.357	0.945	3.46E-03
ENSRNOG00000052498	Grb14	-0.060	-0.041	-0.614	0.124	0.616	3.46E-03
ENSRNOG00000015385	Pink1	0.007	-0.227	-0.668	0.291	0.577	3.46E-03
ENSRNOG00000003191	Bhlhb9	-0.153	0.181	0.843	-0.624	-0.856	3.46E-03
ENSRNOG00000006646	Vopp1	-0.167	0.090	1.296	-0.216	-0.994	3.47E-03
ENSRNOG00000008942	RGD1304963	-0.898	-0.360	-1.385	0.536	1.547	3.47E-03
ENSRNOG00000007949	Rgn	-0.407	-0.781	-1.830	0.660	1.216	3.47E-03
ENSRNOG00000024568	Ndufs7	-0.103	-0.330	-0.950	0.369	0.768	3.47E-03
ENSRNOG00000052025	Aes	0.111	-0.278	-0.798	0.359	0.591	3.47E-03
ENSRNOG00000015869	Pccb	-0.134	-0.263	-0.728	0.258	0.588	3.47E-03
ENSRNOG00000009217	Fbxo6	0.122	-0.250	-0.610	0.349	0.544	3.47E-03
ENSRNOG00000020892	Dpf2	0.014	-0.109	-0.430	0.155	0.361	3.47E-03
ENSRNOG00000017954	Slmo1	0.266	0.299	0.947	-0.577	-1.247	3.47E-03
ENSRNOG00000006236	Dsn1	0.487	0.650	1.306	-0.664	-1.556	3.47E-03
ENSRNOG00000025581	Vwa2	0.745	1.899	3.201	-2.391	-2.895	3.47E-03
ENSRNOG00000028844	Slc9a5	0.371	-0.425	-1.316	0.536	1.128	3.47E-03
ENSRNOG00000012860	Tmem184c	0.038	0.015	0.246	-0.359	-0.605	3.47E-03
ENSRNOG00000004768	Myf5	-0.242	0.429	1.643	-0.720	-1.379	3.47E-03
ENSRNOG00000014791	Peg3	0.061	-0.083	0.485	-0.377	-0.644	3.48E-03
ENSRNOG00000019276	RGD735029	-0.077	-0.124	-0.476	0.102	0.413	3.48E-03
ENSRNOG00000003809	Sat1	-0.137	0.957	1.515	-0.760	-1.149	3.49E-03
ENSRNOG00000050277	Uckl1	0.091	-0.167	-0.640	0.297	0.576	3.50E-03
ENSRNOG00000002278	Tec	0.108	0.561	1.621	-0.591	-1.049	3.51E-03
ENSRNOG00000022417	Prickle3	-0.206	-0.115	-0.416	0.179	0.460	3.51E-03
ENSRNOG00000060329	Emb	0.053	2.355	5.104	-1.897	-4.051	3.52E-03
ENSRNOG00000009571	Wipf3	0.405	-0.800	-1.488	0.659	0.969	3.53E-03

ENSRNOG00000030597	Ankrd52	-0.148	-0.194	-0.700	0.102	0.592	3.53E-03
ENSRNOG00000028063	Tmem38b	-0.091	0.501	0.912	-0.513	-0.719	3.57E-03
ENSRNOG00000031090	RT1-CE7	-0.077	0.828	1.353	-0.423	-0.821	3.60E-03
ENSRNOG00000017108	Syngr1	-0.012	-0.351	-0.759	0.488	0.660	3.61E-03
ENSRNOG00000006997	App	-0.065	0.390	0.879	-0.432	-0.659	3.65E-03
ENSRNOG00000008157	Syn2	0.037	-0.107	-0.476	0.255	1.198	3.66E-03
ENSRNOG00000059142	Dtwd2	-0.016	-0.268	-0.736	0.247	0.646	3.66E-03
ENSRNOG00000018281	Uqcrfs1	-0.217	-0.361	-0.736	0.259	0.644	3.67E-03
ENSRNOG00000056457	Gpd1	0.259	-0.488	-1.984	0.492	1.373	3.68E-03
ENSRNOG00000004195	Dtl	0.438	1.464	2.055	-1.824	-2.828	3.71E-03
ENSRNOG00000008921	Dynll2	-0.248	-0.236	-0.838	0.262	0.571	3.76E-03
ENSRNOG00000049985	Gprasp1	-0.072	0.252	0.682	-0.337	-0.586	3.78E-03
ENSRNOG00000024181	Tpsab1	-0.396	-0.326	-0.278	0.702	1.140	3.80E-03
ENSRNOG00000010029	Ubald2	0.173	-0.380	-1.164	0.400	0.824	3.80E-03
ENSRNOG00000018952	Sema3g	-0.020	-0.023	-0.439	0.110	0.638	3.82E-03
ENSRNOG00000013018	Eda2r	-0.100	2.077	2.944	-1.768	-1.820	3.82E-03
ENSRNOG00000011891	Atp6v1b2	-0.112	-0.114	-0.270	0.116	0.324	3.87E-03
ENSRNOG00000032297	Msmo1	-0.109	0.187	0.539	-0.269	-0.511	3.87E-03
ENSRNOG00000001242	Gstt3	0.179	1.089	1.891	-0.857	-1.192	3.87E-03
ENSRNOG00000009446	Rxra	0.210	-0.177	-0.781	0.288	0.615	3.88E-03
ENSRNOG00000000244	Coil	0.212	-0.265	-0.636	0.378	0.458	3.88E-03
ENSRNOG00000019634	Eif4e2	-0.010	0.097	0.219	-0.226	-0.382	3.88E-03
ENSRNOG00000012210	Sptlc2	-0.089	0.275	0.828	-0.383	-0.653	3.88E-03
ENSRNOG00000012031	St8sia2	0.219	2.054	2.732	-2.439	-3.078	3.88E-03
ENSRNOG00000019518	Pde4c	-0.046	-0.474	-0.920	0.478	0.808	3.89E-03
ENSRNOG00000017077	Snx7	0.015	0.111	0.733	-0.303	-0.656	3.96E-03
ENSRNOG00000060750	Rnf111	-0.156	-0.113	-0.294	0.154	0.445	3.97E-03

ENSRNOG00000020642	Flad1	-0.046	-0.165	-0.509	0.198	0.440	3.97E-03
ENSRNOG00000009414	Creld1	0.028	0.442	0.545	-0.337	-0.534	3.97E-03
ENSRNOG00000052802	Aldoa	0.385	-0.344	-1.436	0.378	0.980	3.98E-03
ENSRNOG00000019982	Ethe1	0.087	-0.184	-0.656	0.205	0.545	3.99E-03
ENSRNOG00000023150	Gpi	0.218	-0.297	-1.393	0.289	0.903	4.00E-03
ENSRNOG00000008058	Tmprss5	0.515	0.617	0.974	-0.566	-0.820	4.00E-03
ENSRNOG00000008600	Sytl1	-0.139	1.409	3.448	-1.116	-2.751	4.01E-03
ENSRNOG00000007600	Igsf1	-0.060	0.822	1.536	-0.906	-1.508	4.03E-03
ENSRNOG00000037720	Rita1	0.109	-0.268	-0.885	0.385	0.741	4.07E-03
ENSRNOG00000033921	Als2cl	-0.206	-0.117	-0.515	0.122	0.616	4.07E-03
ENSRNOG00000050625	LOC100911217	0.409	-0.377	-1.331	0.752	1.651	4.08E-03
ENSRNOG00000030880	Hs6st2	0.303	0.715	1.150	-0.802	-1.301	4.09E-03
ENSRNOG00000048719	Dtnbp1	0.097	-0.299	-0.758	0.434	0.587	4.14E-03
ENSRNOG00000054561	Isg20	0.014	0.958	1.486	-0.964	-1.234	4.16E-03
ENSRNOG00000010748	Mtus1	0.011	-0.218	-0.693	0.128	0.471	4.20E-03
ENSRNOG00000028137	Mki67	0.064	0.921	1.797	-1.261	-1.531	4.21E-03
ENSRNOG00000059215	Pqlc1	-0.207	-0.027	-0.288	0.224	0.538	4.25E-03
ENSRNOG00000011781	Oplah	-0.143	-0.288	-0.755	0.271	0.545	4.25E-03
ENSRNOG00000022331	Ccdc78	-0.080	-0.741	-1.863	0.979	2.060	4.27E-03
ENSRNOG00000028822	Ngfrap1	0.568	0.165	0.586	-0.168	-0.690	4.28E-03
ENSRNOG00000006689	Chd7	0.077	-0.209	-0.855	0.102	0.649	4.28E-03
ENSRNOG00000005963	Rab2a	0.188	-0.148	-0.523	0.235	0.525	4.28E-03
ENSRNOG00000056637	Abhd16a	0.129	-0.234	-0.537	0.309	0.460	4.28E-03
ENSRNOG00000014230	Map1a	-0.020	0.762	1.537	-1.376	-1.596	4.32E-03
ENSRNOG00000016190	Coq9	-0.223	-0.310	-0.875	0.311	0.720	4.33E-03
ENSRNOG00000007801	R3hdm2	0.191	-0.190	-0.558	0.296	0.471	4.33E-03
ENSRNOG00000001448	Hip1	0.138	0.151	0.323	-0.289	-0.482	4.33E-03

ENSRNOG00000033256	LOC691141	-0.064	0.470	1.019	-0.252	-1.089	4.33E-03
ENSRNOG00000020923	Tuft1	-0.026	0.841	1.737	-0.861	-1.214	4.33E-03
ENSRNOG00000003533	Clcn4	-0.171	-0.246	-1.282	0.120	0.888	4.34E-03
ENSRNOG00000008052	Atp13a2	0.108	-0.237	-0.708	0.269	0.536	4.35E-03
ENSRNOG00000013331	Sdha	-0.266	-0.336	-0.795	0.275	0.680	4.36E-03
ENSRNOG00000002045	Anxa3	-0.188	-0.390	-0.447	0.449	0.553	4.37E-03
ENSRNOG00000020753	Bnip1	0.124	-0.360	-0.726	0.419	0.523	4.37E-03
ENSRNOG00000018068	Rn60_2_0457.1	-0.122	-0.266	-0.642	0.334	0.624	4.37E-03
ENSRNOG00000054497	Taf4a	-0.138	-0.053	-0.329	0.118	0.445	4.37E-03
ENSRNOG00000005724	Map3k7	-0.038	0.480	1.072	-0.556	-0.931	4.42E-03
ENSRNOG00000015131	Ube2c	0.202	0.318	1.463	-0.648	-1.801	4.42E-03
ENSRNOG00000003357	Col3a1	0.556	0.195	0.879	-0.728	-1.312	4.43E-03
ENSRNOG00000003918	LOC100910173	-0.118	-0.286	-0.445	0.351	0.427	4.45E-03
ENSRNOG00000006901	Cd320	-0.011	-0.244	-0.403	0.225	0.456	4.46E-03
ENSRNOG00000030355	Mroh1	-0.157	-0.079	-0.346	0.140	0.435	4.46E-03
ENSRNOG00000015999	Cirbp	0.000	0.418	0.677	-0.400	-0.516	4.48E-03
ENSRNOG00000000435	Ppt2	0.170	-0.261	-0.767	0.362	0.537	4.48E-03
ENSRNOG00000018564	Nup93	-0.096	0.434	0.832	-0.515	-0.564	4.48E-03
ENSRNOG00000051838	Atmin	-0.139	-0.299	-0.860	0.279	0.848	4.48E-03
ENSRNOG00000012942	Satb1	0.059	-0.174	-0.860	0.240	0.593	4.48E-03
ENSRNOG00000003967	Vmp1	0.087	0.367	0.668	-0.330	-0.581	4.48E-03
ENSRNOG00000016645	Sfmbt1	0.260	0.484	0.870	-0.416	-0.676	4.48E-03
ENSRNOG00000021137	Kctd15	-0.561	-0.311	-0.938	0.363	0.780	4.49E-03
ENSRNOG00000027742	Adamtsl2	-0.980	1.082	2.240	-0.789	-1.874	4.49E-03
ENSRNOG00000009068	Phlda3	-0.227	0.749	1.709	-0.926	-1.194	4.53E-03
ENSRNOG00000057556	Pdzrn3	-0.061	-0.185	-1.024	0.158	0.646	4.55E-03
ENSRNOG00000061684	Ndufa9	-0.249	-0.297	-0.702	0.251	0.623	4.57E-03

ENSRNOG00000006420	Rbm38	0.007	-0.258	-0.609	0.323	0.509	4.58E-03
ENSRNOG00000020417	Gsk3a	-0.025	-0.126	-0.347	0.171	0.329	4.58E-03
ENSRNOG00000024707	Tp73	-0.274	2.839	6.029	-2.186	-4.613	4.58E-03
ENSRNOG00000031328	Zfp110	0.071	-0.182	-0.531	0.125	0.464	4.58E-03
ENSRNOG00000014336	Mcm5	0.028	0.334	1.224	-0.712	-1.521	4.59E-03
ENSRNOG00000016896	Rpl3	0.100	0.499	1.292	-0.380	-0.759	4.60E-03
ENSRNOG00000019598	Vegfa	-0.451	-0.624	-1.065	0.536	0.932	4.62E-03
ENSRNOG00000024212	Papd5	-0.009	-0.157	-0.497	0.192	0.422	4.62E-03
ENSRNOG00000021492	Rbbp4	0.041	0.110	0.379	-0.133	-0.395	4.62E-03
ENSRNOG00000058375	AABR07054461.1	0.168	-0.904	-1.580	1.151	1.803	4.63E-03
ENSRNOG00000009928	Bckdhb	-0.125	-0.223	-0.753	0.229	0.593	4.63E-03
ENSRNOG00000011533	Irx3	1.321	0.524	1.640	-0.046	-1.578	4.63E-03
ENSRNOG00000016647	Pla2g2c	-0.015	-0.507	-1.843	0.623	1.725	4.65E-03
ENSRNOG00000039860	AC126960.1	0.228	0.901	2.907	-1.175	-3.410	4.68E-03
ENSRNOG00000003815	Slc25a11	-0.104	-0.211	-0.620	0.266	0.534	4.70E-03
ENSRNOG00000006304	Mdm2	0.144	0.318	0.551	-0.295	-0.547	4.70E-03
ENSRNOG00000020546	Lipe	-0.412	-0.455	-0.468	0.536	0.919	4.73E-03
ENSRNOG00000018343	Isca1	0.111	-0.274	-0.647	0.187	0.477	4.74E-03
ENSRNOG00000005504	Pkp4	-0.139	0.352	0.997	-0.378	-0.706	4.81E-03
ENSRNOG00000001211	RGD1303003	-0.175	-0.337	-0.851	0.287	0.716	4.81E-03
ENSRNOG00000042480	Hoxd8	-2.613	-0.346	-0.752	0.356	1.031	4.86E-03
ENSRNOG00000055572	Idh3g	-0.241	-0.294	-0.748	0.252	0.628	4.86E-03
ENSRNOG00000004137	Ubtd2	0.268	0.177	0.522	-0.261	-0.471	4.86E-03
ENSRNOG00000010737	Mbnl2	-0.101	-0.234	-0.515	0.124	0.453	4.86E-03
ENSRNOG00000013714	Snrpd1	0.014	0.070	0.413	-0.116	-0.456	4.99E-03
ENSRNOG00000041744	AABR07065531.1	0.161	-0.211	1.174	-0.587	-2.122	4.99E-03
ENSRNOG00000006961	Snrpb	-0.047	-0.204	-0.487	0.163	0.364	5.00E-03

ENSRNOG00000004555	Sec14l4	-0.303	-0.339	-1.143	0.130	1.572	5.01E-03
ENSRNOG00000005711	Ptprd	0.070	0.884	1.114	-0.657	-0.853	5.04E-03
ENSRNOG00000005618	Fmc1	0.004	-0.237	-0.771	0.275	0.615	5.08E-03
ENSRNOG00000005802	Usp24	-0.106	-0.229	-0.778	0.210	0.608	5.09E-03
ENSRNOG00000061316	AABR07000121.1	0.033	-0.154	-0.520	0.409	1.033	5.13E-03
ENSRNOG00000011057	Mfn1	-0.169	-0.317	-0.576	0.162	0.481	5.13E-03
ENSRNOG00000061100	Tmem150a	0.074	-0.071	-0.525	0.262	0.474	5.15E-03
ENSRNOG00000000955	Lnx2	-0.167	0.370	0.839	-0.459	-0.718	5.15E-03
ENSRNOG00000036794	Asb16	0.110	-0.151	-0.839	0.074	0.537	5.26E-03
ENSRNOG00000015080	Wdfy1	0.210	0.305	0.423	-0.451	-0.477	5.29E-03
ENSRNOG00000010728	Stradb	0.057	-0.390	-0.787	0.179	0.470	5.30E-03
ENSRNOG00000023202	Usp15	0.133	-0.341	-0.781	0.166	0.498	5.31E-03
ENSRNOG00000019743	Tmem63b	-0.066	-0.047	-0.387	0.082	0.464	5.33E-03
ENSRNOG00000025076	AI314180	0.069	-0.149	-0.651	0.146	0.593	5.38E-03
ENSRNOG00000006336	Fyco1	-0.067	-0.105	-0.565	0.049	0.477	5.39E-03
ENSRNOG00000061582	Scamp1	0.018	0.102	0.206	-0.204	-0.296	5.41E-03
ENSRNOG00000030639	Usp13	-0.213	-0.052	-0.479	-0.007	0.554	5.43E-03
ENSRNOG00000016119	Fzd7	-0.123	-0.401	-1.091	0.315	0.802	5.43E-03
ENSRNOG00000033496	Igdcc4	-0.118	0.387	0.656	-0.343	-0.631	5.43E-03
ENSRNOG00000031766	Mt-cytb	-0.396	-0.323	-0.704	0.258	0.685	5.47E-03
ENSRNOG00000013123	Scube2	0.325	0.031	0.414	-0.522	-1.041	5.48E-03
ENSRNOG00000011202	Chrna4	0.884	1.502	3.172	-2.057	-3.216	5.50E-03
ENSRNOG00000005874	Tle2	0.129	-0.032	-0.744	0.254	0.629	5.55E-03
ENSRNOG00000045605	Uxs1	0.099	-0.006	0.137	-0.135	-0.316	5.55E-03
ENSRNOG00000019090	Cct3	-0.075	0.086	0.500	-0.102	-0.425	5.58E-03
ENSRNOG00000010966	Itgb1	-0.061	0.010	0.478	-0.201	-0.476	5.58E-03
ENSRNOG00000018317	Aak1	0.072	0.573	0.721	-0.704	-0.708	5.58E-03

ENSRNOG00000053679	AABR07060862.1	-0.213	1.041	2.108	-1.669	-1.800	5.58E-03
ENSRNOG00000058136	Vom1r90	-0.169	-0.550	-0.905	0.623	0.866	5.59E-03
ENSRNOG00000011794	Tcea3	0.079	-0.304	-0.918	0.375	0.653	5.59E-03
ENSRNOG00000056550	Epb41l4b	0.250	0.422	1.024	-0.636	-1.095	5.59E-03
ENSRNOG00000009334	Knstrn	0.678	1.164	2.303	-1.520	-2.115	5.59E-03
ENSRNOG00000027278	Pramef8	0.120	-0.182	-0.708	0.195	0.563	5.60E-03
ENSRNOG00000018462	Rabep2	0.504	-0.289	-1.255	0.318	0.913	5.60E-03
ENSRNOG00000018662	Amacr	-0.089	-0.206	-0.481	0.183	0.392	5.62E-03
ENSRNOG00000043076	AABR07025328.1	-0.247	0.375	0.805	-0.318	-0.632	5.62E-03
ENSRNOG00000055450	Ckmt2	-0.794	-0.622	-1.095	0.502	1.196	5.65E-03
ENSRNOG00000017032	Atp5a1	-0.159	-0.308	-0.732	0.273	0.607	5.65E-03
ENSRNOG00000019382	Zbtb47	0.009	-0.116	-0.495	0.184	0.400	5.65E-03
ENSRNOG00000014852	Fbxo30	-0.154	0.946	1.286	-0.917	-0.927	5.65E-03
ENSRNOG00000013309	Pik3ap1	-0.205	-0.312	-1.171	0.268	0.839	5.66E-03
ENSRNOG00000021125	Prdx5	-0.224	-0.245	-0.637	0.289	0.559	5.70E-03
ENSRNOG00000007687	Sema7a	0.198	-0.891	-2.518	0.681	1.433	5.72E-03
ENSRNOG00000018225	Tp53inp2	-0.080	-0.383	-0.938	0.201	0.586	5.76E-03
ENSRNOG00000018082	Slc26a2	-0.250	0.728	1.587	-0.958	-1.253	5.82E-03
ENSRNOG00000001989	Alcam	0.030	0.506	1.038	-0.429	-0.904	5.83E-03
ENSRNOG00000025302	Cdca2	0.553	0.443	1.647	-0.886	-2.400	5.83E-03
ENSRNOG00000047124	Atp2a1	0.528	-0.313	-1.373	0.316	0.928	5.86E-03
ENSRNOG00000017095	LOC679881	-0.233	-0.312	-0.805	0.152	0.592	5.86E-03
ENSRNOG00000043426	Ccbl2	0.020	-0.202	-0.442	0.231	0.624	5.88E-03
ENSRNOG00000060350	Iqsec1	-0.265	-0.283	-0.658	0.426	0.675	5.90E-03
ENSRNOG00000013555	Ankrd55	0.527	0.531	1.534	-0.856	-1.613	5.90E-03
ENSRNOG00000021260	Prnd	0.053	1.804	3.206	-2.247	-3.325	5.90E-03
ENSRNOG00000016459	Eif3j	0.137	-0.332	-0.623	0.290	0.496	5.92E-03

ENSRNOG00000001070	Cdk2ap1	0.214	0.106	0.367	-0.107	-0.484	5.93E-03
ENSRNOG00000009951	Aif1l	0.061	0.473	1.228	-0.229	-0.959	5.95E-03
ENSRNOG00000019522	Narfl	-0.268	-0.132	-0.613	0.155	0.544	5.96E-03
ENSRNOG00000015866	Hint2	-0.013	-0.168	-0.578	0.269	0.480	5.96E-03
ENSRNOG00000009555	Lrriq3	0.831	1.020	0.982	-1.580	-1.662	5.97E-03
ENSRNOG00000027891	Dhrs11	-0.581	-0.239	-0.888	0.333	0.917	5.99E-03
ENSRNOG00000020881	Frmd8	-0.025	0.307	0.651	-0.230	-0.457	6.03E-03
ENSRNOG00000029911	Cilp	-0.834	1.613	2.631	-1.678	-1.783	6.03E-03
ENSRNOG00000060899	Tsc22d3	0.026	-0.478	-1.258	0.360	0.695	6.05E-03
ENSRNOG00000009761	Tmod1	0.353	0.312	0.371	-0.260	-0.605	6.05E-03
ENSRNOG00000028649	Tox3	0.343	0.597	0.755	-0.686	-1.438	6.05E-03
ENSRNOG00000007700	Inhbc	0.280	-0.251	-0.777	0.527	0.999	6.06E-03
ENSRNOG00000003746	Gjb1	-1.171	-1.506	-1.879	1.148	2.847	6.07E-03
ENSRNOG00000059827	LOC691995	-0.247	0.748	1.957	-0.434	-1.276	6.07E-03
ENSRNOG00000023208	RGD1309821	0.119	-0.189	-0.970	0.198	0.694	6.10E-03
ENSRNOG00000007688	Sms	0.047	0.130	0.459	-0.214	-0.624	6.14E-03
ENSRNOG00000011014	Copg2	0.022	0.183	0.400	-0.246	-0.418	6.16E-03
ENSRNOG00000017671	Rasa3	0.082	0.421	0.882	-0.412	-0.589	6.21E-03
ENSRNOG00000021130	Abcc8	0.186	-0.002	-0.987	0.220	0.774	6.21E-03
ENSRNOG00000042482	Tatdn2	0.038	-0.240	-0.587	0.156	0.390	6.21E-03
ENSRNOG00000001254	Col6a2	0.190	0.153	0.693	-0.321	-0.744	6.21E-03
ENSRNOG00000013917	Igsf10	-0.787	0.344	1.695	-0.126	-1.234	6.22E-03
ENSRNOG00000037211	Kif14	0.240	0.869	2.367	-1.083	-2.748	6.23E-03
ENSRNOG00000026974	Dbndd1	-0.579	1.392	3.352	-2.031	-3.383	6.23E-03
ENSRNOG00000000940	Flt1	-0.147	-0.357	-0.720	0.308	0.819	6.27E-03
ENSRNOG00000021102	Scn1b	0.235	-0.172	-0.813	0.277	0.585	6.27E-03
ENSRNOG00000007290	Atp1a2	0.228	-0.273	-0.854	0.160	0.557	6.29E-03

ENSRNOG00000007364	Rab15	-0.300	0.497	2.609	-1.047	-2.595	6.33E-03
ENSRNOG00000000442	Notch4	-0.208	-0.105	-0.478	0.163	0.641	6.37E-03
ENSRNOG00000019363	Fam212a	0.338	1.039	1.526	-0.469	-2.041	6.37E-03
ENSRNOG00000054508	Foxp2	0.023	-0.184	-0.387	0.238	0.735	6.41E-03
ENSRNOG00000011507	Pick1	0.086	-0.198	-0.766	0.221	0.477	6.41E-03
ENSRNOG00000037148	Adap2	-0.168	0.843	1.924	-0.847	-1.555	6.43E-03
ENSRNOG00000027326	Tada2b	-0.043	-0.097	-0.429	0.109	0.355	6.43E-03
ENSRNOG00000042932	Ttc27	0.771	0.365	0.617	-0.462	-0.791	6.43E-03
ENSRNOG00000005333	Azin1	0.090	0.201	0.457	-0.282	-0.552	6.59E-03
ENSRNOG00000010676	Smarce1	0.024	0.162	0.326	-0.194	-0.310	6.69E-03
ENSRNOG00000019203	Eya2	-0.474	-0.023	0.026	0.520	0.736	6.69E-03
ENSRNOG00000032134	Uqcrc1	-0.323	-0.283	-0.720	0.246	0.643	6.69E-03
ENSRNOG00000050742	Dnmbp	-0.012	-0.155	-0.529	0.177	0.562	6.69E-03
ENSRNOG00000001440	Mdh2	-0.139	-0.270	-0.861	0.243	0.660	6.69E-03
ENSRNOG00000016419	Pdlim5	-0.006	-0.199	-0.458	0.146	0.507	6.69E-03
ENSRNOG00000012017	Otulin	-0.147	1.074	1.777	-0.984	-1.152	6.69E-03
ENSRNOG00000029941	Grina	0.176	0.383	0.488	-0.348	-0.483	6.76E-03
ENSRNOG00000030328	Fam98a	-0.012	-0.158	-0.382	0.099	0.330	6.77E-03
ENSRNOG00000014007	Gfod1	-0.253	0.028	-0.660	0.135	0.771	6.79E-03
ENSRNOG00000010258	Vhl	0.038	0.093	0.445	-0.251	-0.409	6.85E-03
ENSRNOG00000032946	Pdzd7	0.172	-0.552	-2.067	0.536	1.483	6.86E-03
ENSRNOG00000053735	Hebp2	-0.363	1.323	2.406	-0.929	-1.563	6.87E-03
ENSRNOG00000021144	Cebpg	0.090	-0.055	-0.393	0.093	0.402	6.88E-03
ENSRNOG00000018515	Det1	0.195	-0.404	-1.036	0.373	0.708	6.90E-03
ENSRNOG00000016706	Fanca	0.211	0.308	-1.223	0.980	2.384	6.90E-03
ENSRNOG00000049484	Atp9a	0.001	-0.011	-0.613	-0.002	0.424	6.97E-03
ENSRNOG00000014665	Dhdds	-0.328	0.540	0.937	-0.396	-0.561	6.97E-03

ENSRNOG00000003855	Dnaja3	-0.120	-0.173	-0.443	0.172	0.397	6.97E-03
ENSRNOG00000046984	St6galnac6	0.231	-0.111	-0.560	0.148	0.445	6.97E-03
ENSRNOG00000005929	Them6	0.116	-0.388	-1.005	0.643	0.737	7.01E-03
ENSRNOG00000015117	Lrrc39	-0.145	-0.426	-0.826	0.290	0.635	7.01E-03
ENSRNOG00000020865	Ano1	-0.678	0.401	1.129	-0.373	-0.841	7.01E-03
ENSRNOG00000012318	Aspm	0.186	0.779	1.418	-0.834	-1.403	7.01E-03
ENSRNOG00000004566	Arhgef15	-0.172	-0.271	-0.541	0.401	0.675	7.05E-03
ENSRNOG00000020751	Rdm1	0.126	-0.075	-0.265	0.199	0.422	7.08E-03
ENSRNOG00000007151	Cdk14	-0.062	0.108	0.936	-0.308	-0.737	7.11E-03
ENSRNOG00000010092	Magix	0.202	-0.225	-0.881	0.260	0.598	7.17E-03
ENSRNOG00000055756	Bcap31	0.093	0.290	0.583	-0.289	-0.558	7.17E-03
ENSRNOG00000011435	Osbpl10	0.113	0.093	0.845	-0.510	-1.025	7.17E-03
ENSRNOG00000018975	Atg9a	0.191	-0.192	-0.747	0.124	0.473	7.19E-03
ENSRNOG00000015654	Ghr	0.045	-0.246	-0.645	0.315	0.514	7.20E-03
ENSRNOG00000025806	Prr3	0.046	0.232	0.386	-0.366	-0.403	7.23E-03
ENSRNOG00000011632	Cct5	0.077	0.052	0.247	-0.081	-0.294	7.24E-03
ENSRNOG00000007254	Ttc9	0.304	0.395	0.940	-0.922	-0.880	7.24E-03
ENSRNOG00000010598	Hs3st1	-0.017	-0.247	-0.623	0.583	0.865	7.26E-03
ENSRNOG00000055761	Zfp574	-0.050	-0.186	-0.620	0.309	0.478	7.26E-03
ENSRNOG00000025338	Msi2	0.008	-0.178	-0.486	0.054	0.336	7.26E-03
ENSRNOG00000043233	LOC100912391	0.188	0.152	0.062	-0.228	-0.409	7.26E-03
ENSRNOG00000026212	Micall1	-0.101	0.256	0.473	-0.273	-0.433	7.26E-03
ENSRNOG00000011912	Tmem38a	0.314	-0.183	-0.782	0.191	0.513	7.30E-03
ENSRNOG00000016470	Ndufa10	-0.260	-0.247	-0.580	0.182	0.542	7.35E-03
ENSRNOG00000037850	mars.02	0.071	-0.209	-0.393	0.285	0.377	7.38E-03
ENSRNOG00000019384	Med11	0.071	0.231	0.676	-0.308	-0.568	7.45E-03
ENSRNOG00000021031	Grn	0.118	0.203	0.625	-0.135	-0.461	7.48E-03

ENSRNOG00000000812	RGD1302996	-0.084	-0.279	-0.826	0.304	0.680	7.57E-03
ENSRNOG00000027183	Zfyve9	0.051	-0.192	-0.568	0.075	0.451	7.58E-03
ENSRNOG00000007040	Timm17a	-0.051	-0.322	-0.779	0.276	0.531	7.59E-03
ENSRNOG00000009781	Dync1i2	-0.012	0.042	0.187	-0.139	-0.254	7.59E-03
ENSRNOG00000001762	Pcyt1a	0.165	-0.095	-0.415	0.112	0.416	7.66E-03
ENSRNOG00000015641	Exoc3l1	-0.064	-0.221	-0.690	0.384	0.742	7.66E-03
ENSRNOG00000008063	Hibadh	-0.161	-0.130	-0.232	0.158	0.421	7.66E-03
ENSRNOG00000010298	Xbp1	0.171	-0.142	-0.266	0.217	0.312	7.66E-03
ENSRNOG00000003736	Col5a2	0.080	0.100	0.721	-0.394	-0.795	7.66E-03
ENSRNOG00000016483	Myo16	-0.376	0.781	1.775	-1.427	-2.268	7.69E-03
ENSRNOG00000011944	Snx10	-0.016	1.080	1.906	-0.975	-1.312	7.73E-03
ENSRNOG00000005668	Ndufa8	-0.135	-0.325	-0.741	0.290	0.600	7.81E-03
ENSRNOG00000060464	Plxna3	-0.211	0.566	0.904	-0.376	-0.728	7.81E-03
ENSRNOG00000016021	Lims2	-0.136	-0.299	-0.418	0.439	0.681	7.83E-03
ENSRNOG00000008289	Slc25a3	-0.260	-0.305	-0.675	0.235	0.600	7.85E-03
ENSRNOG00000013589	Cxcl12	-0.127	-0.246	-0.596	0.279	0.724	7.86E-03
ENSRNOG00000020860	Tdrkh	-0.198	0.620	-0.013	0.674	1.187	7.87E-03
ENSRNOG00000017836	Rrp36	0.268	-0.281	-0.580	0.298	0.478	7.91E-03
ENSRNOG00000006375	Vdac1	-0.126	-0.310	-0.638	0.270	0.532	7.94E-03
ENSRNOG00000014076	Mbnl1	0.079	-0.036	-0.695	0.017	0.584	7.96E-03
ENSRNOG00000053428	Ccpg1	-0.041	-0.250	-0.471	0.229	0.467	7.98E-03
ENSRNOG00000017564	Mib2	-0.100	-0.063	-0.337	0.213	0.379	7.98E-03
ENSRNOG00000056060	AC094647.2	-0.524	2.005	3.615	-1.150	-3.079	8.02E-03
ENSRNOG00000007398	Zfp691	0.114	-0.372	-0.761	0.344	0.492	8.05E-03
ENSRNOG00000021129	RGD1308428	-0.224	-0.132	-0.353	0.119	0.387	8.05E-03
ENSRNOG00000026548	Dhrs7c	0.475	-0.523	-1.563	0.477	1.010	8.08E-03
ENSRNOG00000015517	Zfp444	-0.179	-0.205	-0.457	0.316	0.369	8.08E-03

ENSRNOG00000024085	Tmem237	0.000	0.138	0.226	-0.213	-0.362	8.08E-03
ENSRNOG00000039976	Ptk7	0.188	0.204	0.724	-0.759	-1.088	8.15E-03
ENSRNOG00000020696	Pmvk	-0.096	-0.215	-0.465	0.392	0.495	8.17E-03
ENSRNOG00000012710	Ubac2	-0.062	-0.116	-0.269	0.164	0.314	8.21E-03
ENSRNOG00000006735	Cdkn2b	0.035	1.957	2.930	-1.569	-2.024	8.21E-03
ENSRNOG00000018384	Adam12	0.199	0.342	1.075	-0.969	-1.665	8.23E-03
ENSRNOG00000020573	Efna1	-0.177	-0.084	-0.414	0.253	0.597	8.25E-03
ENSRNOG00000007713	Tmcc3	0.207	-0.322	-0.988	0.234	0.598	8.29E-03
ENSRNOG00000018119	Dhx32	-0.347	-0.112	-0.327	0.195	0.465	8.29E-03
ENSRNOG00000021133	Lsm14a	0.065	-0.184	-0.352	0.171	0.290	8.29E-03
ENSRNOG00000005258	Myef2	0.097	-0.028	0.168	-0.356	-0.364	8.29E-03
ENSRNOG00000058898	Nedd4	0.144	0.336	0.420	-0.342	-0.385	8.29E-03
ENSRNOG00000046472	Arfgap3	0.055	0.424	0.884	-0.330	-0.557	8.29E-03
ENSRNOG00000034150	Ftl1l1	0.214	0.335	1.607	-0.305	-1.458	8.30E-03
ENSRNOG00000055446	Amfr	0.166	-0.114	-0.495	0.135	0.349	8.35E-03
ENSRNOG00000008785	Klf5	0.126	0.529	1.282	-0.687	-1.061	8.35E-03
ENSRNOG00000002214	Klhl8	0.329	0.519	0.786	-0.421	-0.803	8.40E-03
ENSRNOG00000003720	Prrx1	0.135	0.241	0.283	-0.317	-0.432	8.41E-03
ENSRNOG00000033979	AABR07047140.1	0.221	-0.202	-0.458	0.276	0.432	8.42E-03
ENSRNOG00000033402	LOC501110	0.167	0.100	0.358	-0.280	-0.529	8.43E-03
ENSRNOG00000033736	Diras3	0.903	-0.357	0.740	-0.244	-1.057	8.43E-03
ENSRNOG00000046211	Fbxw4	0.075	-0.224	-0.399	0.353	0.358	8.44E-03
ENSRNOG00000046502	Lonp1	-0.161	-0.101	-0.348	0.126	0.344	8.48E-03
ENSRNOG00000019474	Rsph9	-0.291	-0.242	-0.864	0.401	1.096	8.54E-03
ENSRNOG00000013503	Cdh24	0.411	1.085	1.355	-0.919	-1.558	8.54E-03
ENSRNOG00000039551	Rdh14	0.164	-0.326	-0.723	0.250	0.435	8.55E-03
ENSRNOG00000004604	Slc38a10	0.132	0.216	0.412	-0.269	-0.376	8.55E-03

ENSRNOG00000050729	AABR07054265.1	0.282	0.084	0.221	-0.455	-0.813	8.57E-03
ENSRNOG00000009484	Ptcd3	-0.090	-0.227	-0.424	0.123	0.416	8.57E-03
ENSRNOG00000009994	Dlat	-0.223	-0.357	-0.839	0.156	0.585	8.58E-03
ENSRNOG00000019057	Prkcq	0.268	-0.145	-0.813	0.259	0.564	8.58E-03
ENSRNOG00000055391	Eif4ebp2	0.106	-0.077	-0.455	0.177	0.495	8.61E-03
ENSRNOG00000019962	Sars2	-0.126	-0.116	-0.711	0.205	0.500	8.65E-03
ENSRNOG00000059166	Ldb3	-0.117	-0.105	-0.670	0.099	0.533	8.69E-03
ENSRNOG00000012831	Slc25a26	-0.030	-0.294	-0.715	0.176	0.478	8.72E-03
ENSRNOG00000049437	Gpc1	0.401	0.119	0.164	-0.223	-0.418	8.82E-03
ENSRNOG00000018102	Coa5	-0.114	-0.195	-0.579	0.033	0.505	8.83E-03
ENSRNOG00000007427	Entpd6	0.108	-0.191	-0.853	0.260	0.606	8.85E-03
ENSRNOG00000008917	Ehbp1	-0.072	-0.119	-0.517	0.196	0.435	8.85E-03
ENSRNOG00000010941	Tifa	0.301	0.760	1.232	-0.567	-0.974	8.85E-03
ENSRNOG00000005679	Fap	0.346	0.280	0.967	-0.551	-0.826	8.86E-03
ENSRNOG00000019730	Inppl1	0.064	0.331	0.499	-0.314	-0.510	8.89E-03
ENSRNOG00000057352	AABR07054593.1	-0.013	0.339	0.741	-0.230	-0.700	8.89E-03
ENSRNOG00000016368	Ppp1r14c	-0.522	3.228	4.893	-2.876	-3.664	8.90E-03
ENSRNOG00000007964	Tp53inp1	-0.148	0.982	1.228	-0.846	-0.942	8.91E-03
ENSRNOG00000006224	Klhl31	0.126	-0.385	-1.325	0.270	0.799	9.02E-03
ENSRNOG00000010633	Acsl1	-0.607	-0.382	-0.630	0.303	0.788	9.02E-03
ENSRNOG00000047565	LOC100911186	-0.172	-0.152	-0.599	0.361	0.746	9.02E-03
ENSRNOG00000001288	Gpr146	-0.027	0.019	-0.358	0.234	0.723	9.02E-03
ENSRNOG00000033883	Stard8	-0.023	0.112	0.324	-0.242	-0.447	9.02E-03
ENSRNOG00000012794	Grhpr	0.328	-0.364	-1.254	0.373	0.808	9.04E-03
ENSRNOG00000014064	Ctsh	0.372	0.147	0.627	-0.350	-0.647	9.05E-03
ENSRNOG00000018951	Col4a5	0.146	0.036	0.316	-0.278	-0.605	9.09E-03
ENSRNOG00000015217	Ltv1	0.000	0.085	0.395	-0.068	-0.368	9.12E-03

ENSRNOG00000004430	Cep131	0.096	-0.100	-0.584	0.203	0.536	9.12E-03
ENSRNOG00000001580	Hoxd9	-3.201	-0.247	-0.654	0.515	1.213	9.14E-03
ENSRNOG00000019039	Inpp5e	-0.095	-0.203	-0.425	0.184	0.329	9.17E-03
ENSRNOG00000056069	Kif11	0.088	0.750	1.914	-1.080	-1.882	9.20E-03
ENSRNOG00000001682	Ttc3	0.054	0.075	0.371	-0.181	-0.444	9.23E-03
ENSRNOG00000013949	Idh2	-0.654	-0.190	-0.532	0.321	0.801	9.25E-03
ENSRNOG00000042449	LOC100362023	-0.013	0.339	0.495	-0.352	-0.356	9.28E-03
ENSRNOG00000026061	RGD1566226	-0.300	2.893	4.578	-2.629	-4.029	9.33E-03
ENSRNOG00000011222	Dynll1	0.493	0.312	0.928	-0.173	-0.793	9.35E-03
ENSRNOG00000059834	Cib2	0.945	-0.607	-1.804	0.585	1.137	9.36E-03
ENSRNOG00000033765	Eif1	0.203	-0.213	-0.482	0.298	0.442	9.36E-03
ENSRNOG00000024437	Blcap	0.053	-0.030	-0.504	0.074	0.414	9.36E-03
ENSRNOG00000019620	Pmf1	0.194	0.298	0.698	-0.189	-0.826	9.36E-03
ENSRNOG00000000387	Slc25a16	-0.106	0.215	0.672	-0.326	-0.521	9.39E-03
ENSRNOG00000019525	Hspa9	-0.237	-0.286	-0.536	0.167	0.514	9.39E-03
ENSRNOG00000003278	Map3k14	0.233	0.921	1.232	-0.548	-0.833	9.41E-03
ENSRNOG00000033261	Fam107a	-0.225	-0.292	-0.654	0.587	0.987	9.41E-03
ENSRNOG00000000979	Bri3bp	-0.049	0.409	0.509	-0.550	-0.720	9.41E-03
ENSRNOG00000022523	Fkbp5	0.159	-0.243	-0.781	0.294	0.930	9.49E-03
ENSRNOG00000018454	Apoe	0.244	0.569	1.550	-0.342	-1.056	9.55E-03
ENSRNOG00000008933	Plbd1	-0.081	-0.288	-0.489	0.181	0.373	9.55E-03
ENSRNOG00000010512	Yipf1	-0.030	0.283	0.503	-0.151	-0.336	9.59E-03
ENSRNOG00000028460	Lrrc8e	1.104	0.111	0.827	-2.436	-1.731	9.63E-03
ENSRNOG00000008897	Gga1	0.027	-0.037	-0.351	0.112	0.348	9.68E-03
ENSRNOG00000032048	Zfp462	0.309	-0.418	-0.800	0.345	0.740	9.70E-03
ENSRNOG00000023851	Igsf3	-0.145	0.144	0.691	-0.477	-0.744	9.70E-03
ENSRNOG00000047741	E2f2	0.312	0.272	0.482	-0.549	-0.887	9.70E-03

ENSRNOG00000052128	Clec2e	0.716	0.716	2.693	-0.954	-2.384	9.70E-03
ENSRNOG00000037137	Svip	0.101	-0.322	-0.754	0.270	0.487	9.78E-03
ENSRNOG00000003884	Acmsd	-2.216	-0.319	-1.812	1.338	1.912	9.79E-03
ENSRNOG00000017084	Hsd11b2	-0.524	1.624	3.188	-1.448	-2.224	9.84E-03
ENSRNOG00000001455	Kif13a	-0.168	-0.036	-0.454	0.065	0.435	9.85E-03
ENSRNOG00000020284	Prkar2a	0.115	-0.274	-0.803	0.182	0.515	9.85E-03
ENSRNOG00000040350	Mir675	0.264	0.881	1.185	-0.519	-1.028	9.90E-03
ENSRNOG00000018651	Agtpbp1	0.435	-0.300	-1.046	0.284	0.703	9.91E-03
ENSRNOG00000010697	Hadh	-0.506	-0.306	-0.575	0.323	0.731	9.92E-03
ENSRNOG00000004449	E2f6	-0.123	-0.261	-0.605	0.220	0.489	9.92E-03
ENSRNOG00000017166	Mycbp	-0.001	0.148	0.315	-0.224	-0.412	9.92E-03
ENSRNOG00000047656	Gaa	0.052	-0.003	-0.407	0.079	0.443	9.93E-03
ENSRNOG00000026519	AABR07044711.1	0.165	-0.309	-1.433	0.226	0.832	9.93E-03
ENSRNOG00000019843	St3gal3	0.234	-0.092	-0.523	0.245	0.450	9.93E-03
ENSRNOG00000039454	Pcdhgb7	-0.443	-0.212	-0.336	0.672	0.879	9.94E-03
ENSRNOG00000013514	Maf1	0.133	-0.176	-0.450	0.249	0.360	9.94E-03
ENSRNOG00000008829	Sorbs3	-0.242	-0.207	-0.421	0.239	0.619	9.96E-03
ENSRNOG00000012262	Depdc7	-0.184	0.517	1.421	-0.718	-1.169	9.96E-03
ENSRNOG00000055111	AABR07000658.1	0.243	0.421	1.627	-1.039	-2.254	9.96E-03
ENSRNOG00000040108	Cd36	-0.851	-0.618	-0.447	0.613	1.120	9.97E-03
ENSRNOG00000017513	Fam73b	-0.270	-0.304	-0.766	0.338	0.631	9.97E-03
ENSRNOG00000018425	Dym	0.379	-0.146	-0.756	0.201	0.537	9.97E-03
ENSRNOG00000007111	Nrd1	0.190	-0.133	-0.453	0.105	0.351	9.97E-03
ENSRNOG00000020713	Osbpl5	-0.056	0.171	0.547	-0.332	-0.449	9.97E-03
ENSRNOG00000033844	LOC100912195	0.188	0.981	1.907	-0.842	-1.307	9.97E-03
ENSRNOG00000039658	G4	-0.136	-0.234	-0.391	0.274	0.380	9.98E-03
ENSRNOG00000008103	Mdh1	-0.542	-0.453	-0.800	0.406	0.933	1.00E-02

ENSRNOG00000019674	Tmem180	0.077	-0.023	-0.391	0.216	0.479	1.00E-02
ENSRNOG00000021732	Hsf1	0.057	-0.147	-0.383	0.209	0.346	1.00E-02
ENSRNOG00000020837	Cd300lg	-0.205	-1.027	-1.400	1.141	1.890	1.00E-02
ENSRNOG00000057139	Myoz3	0.002	-0.335	-1.229	0.263	0.819	1.00E-02
ENSRNOG00000009191	Ccm2l	-0.290	-0.169	-0.521	0.405	0.680	1.00E-02
ENSRNOG00000057040	Spns2	-0.052	-0.158	-0.580	0.191	0.674	1.00E-02
ENSRNOG00000010830	Slc25a4	-0.039	-0.343	-0.833	0.261	0.567	1.00E-02
ENSRNOG00000030579	Zfp853	0.581	0.739	0.888	-0.870	-1.116	1.00E-02
ENSRNOG00000028082	Tal2	-0.170	1.884	3.203	-2.552	-2.533	1.00E-02
ENSRNOG00000003018	Olfml2b	0.036	0.030	0.794	-0.188	-0.851	1.00E-02
ENSRNOG00000031979	Mt-atp6	-0.436	-0.344	-0.648	0.295	0.682	1.01E-02
ENSRNOG00000019249	Ap3b2	0.451	0.841	1.442	-0.616	-2.502	1.01E-02
ENSRNOG00000025890	Opa3	-0.019	-0.124	-0.618	0.107	0.425	1.03E-02
ENSRNOG00000022189	Htatip2	-0.293	0.650	1.362	-0.618	-0.856	1.03E-02
ENSRNOG00000046005	Scd2	-0.206	1.079	1.412	-0.863	-1.133	1.03E-02
ENSRNOG00000037659	Mto1	-0.101	-0.391	-0.509	0.152	0.389	1.03E-02
ENSRNOG00000020087	Zfp90	0.023	0.381	0.854	-0.495	-0.915	1.04E-02
ENSRNOG00000027264	Dagla	0.079	-0.213	-0.941	0.196	0.722	1.04E-02
ENSRNOG00000023841	Cd99l2	0.303	0.599	0.830	-0.633	-1.168	1.04E-02
ENSRNOG00000010099	Asb8	0.088	-0.131	-0.454	0.097	0.349	1.04E-02
ENSRNOG00000009845	Acadm	-0.384	-0.485	-0.702	0.422	0.822	1.04E-02
ENSRNOG00000019590	Smg5	-0.007	-0.018	-0.227	0.037	0.277	1.04E-02
ENSRNOG00000005574	Adamts8	-0.137	-0.200	0.654	-0.986	-1.621	1.04E-02
ENSRNOG00000021789	RGD1565323	0.618	-0.718	-2.231	0.293	1.478	1.05E-02
ENSRNOG00000009373	AABR07069599.1	0.251	-0.310	-1.120	0.299	0.701	1.05E-02
ENSRNOG00000025742	Lmnb2	-0.007	0.958	1.714	-1.185	-1.546	1.05E-02
ENSRNOG00000045665	Amigo1	-0.093	-0.088	-0.380	0.084	0.461	1.05E-02

ENSRNOG00000000780	Ppp1r11	0.062	0.237	0.405	-0.151	-0.289	1.05E-02
ENSRNOG00000003661	Hn1	0.335	0.320	0.349	-0.309	-0.511	1.05E-02
ENSRNOG00000047712	Dcx	0.348	0.533	0.982	-1.096	-1.673	1.05E-02
ENSRNOG00000022710	Prrg4	-0.537	2.213	3.655	-2.244	-2.437	1.05E-02
ENSRNOG00000038372	Ndufs2	-0.267	-0.259	-0.643	0.253	0.584	1.05E-02
ENSRNOG00000019267	Dkk4	-0.073	-0.156	-0.346	0.172	0.390	1.05E-02
ENSRNOG00000005045	Ppp2r5e	0.008	-0.079	-0.276	0.087	0.266	1.06E-02
ENSRNOG00000005147	Ccdc157	-0.265	-0.242	-0.881	0.143	0.659	1.07E-02
ENSRNOG00000017215	RGD1308601	-0.038	-0.094	-0.512	0.052	0.393	1.07E-02
ENSRNOG00000006608	Tmem70	-0.175	-0.286	-0.613	0.229	0.493	1.09E-02
ENSRNOG00000002863	Cacna1e	-4.061	-1.366	-2.465	1.363	4.185	1.09E-02
ENSRNOG00000036677	Slc16a3	-0.015	-0.354	-1.740	0.344	1.063	1.09E-02
ENSRNOG00000009934	Emc3	0.171	0.379	0.484	-0.342	-0.457	1.09E-02
ENSRNOG00000007262	Ccdc134	0.107	0.313	0.647	-0.338	-0.628	1.09E-02
ENSRNOG00000046204	Tspan15	0.195	0.730	1.013	-0.589	-0.886	1.09E-02
ENSRNOG00000011329	Pkm	0.131	-0.320	-1.406	0.276	0.837	1.09E-02
ENSRNOG00000010227	Gpatch2l	0.051	0.181	0.304	-0.277	-0.341	1.09E-02
ENSRNOG00000055068	LOC100360977	0.072	0.279	0.723	-0.239	-0.522	1.10E-02
ENSRNOG00000021174	Macrod1	0.090	-0.298	-0.958	0.380	0.662	1.10E-02
ENSRNOG00000006238	Traf2	-0.041	0.466	0.679	-0.361	-0.487	1.10E-02
ENSRNOG00000020843	Ftl1	0.028	0.451	1.163	-0.208	-0.628	1.11E-02
ENSRNOG00000012574	LOC499339	0.051	0.401	0.666	-0.477	-0.508	1.11E-02
ENSRNOG00000048897	Vmac	-0.312	-0.220	-0.307	0.265	0.585	1.12E-02
ENSRNOG00000013431	Rsbn1l	-0.010	-0.335	-0.713	0.241	0.427	1.12E-02
ENSRNOG00000018412	Sfi1	-0.039	0.248	0.493	-0.275	-0.393	1.12E-02
ENSRNOG00000014371	Cdh13	0.061	0.567	0.849	-0.432	-0.580	1.14E-02
ENSRNOG00000004205	Pkdcc	-0.084	-0.458	-0.948	0.517	0.770	1.14E-02

ENSRNOG00000017079	Pgm2l1	0.148	-0.225	-0.778	0.292	0.724	1.14E-02
ENSRNOG00000019278	Fsd2	-0.039	-0.284	-1.074	0.227	0.615	1.14E-02
ENSRNOG00000015679	Dok4	0.135	-0.020	0.136	-0.114	-0.610	1.14E-02
ENSRNOG00000038091	Sohlh2	-0.070	3.667	5.055	-3.595	-5.284	1.14E-02
ENSRNOG00000045821	Slc41a3	-0.026	-0.644	-2.018	0.445	1.291	1.14E-02
ENSRNOG00000014761	Rasd2	-0.233	-0.147	-1.150	0.500	1.221	1.14E-02
ENSRNOG00000018009	Rab8b	0.117	0.209	0.461	-0.367	-0.402	1.14E-02
ENSRNOG00000020518	Fam189b	0.085	-0.309	-0.895	0.288	0.612	1.14E-02
ENSRNOG00000020167	Reep5	0.292	-0.072	-0.447	0.125	0.341	1.14E-02
ENSRNOG00000007325	Usp28	0.057	0.408	0.642	-0.440	-0.562	1.14E-02
ENSRNOG00000020853	Scap	-0.065	0.253	0.429	-0.192	-0.366	1.14E-02
ENSRNOG00000061326	AABR07054487.1	-2.095	-0.803	-2.010	-0.065	3.052	1.15E-02
ENSRNOG00000019318	Syt3	-0.567	0.154	-0.573	0.390	1.122	1.15E-02
ENSRNOG00000017149	Fam131b	0.102	-0.381	-1.065	0.357	0.727	1.15E-02
ENSRNOG00000006649	Thrb	-0.055	-0.221	-0.490	0.170	0.443	1.15E-02
ENSRNOG00000021232	Ddrgk1	0.135	-0.226	-0.571	0.286	0.393	1.15E-02
ENSRNOG00000052493	AABR07068327.1	-0.259	1.290	1.658	-1.052	-1.219	1.16E-02
ENSRNOG00000051420	Cables2	0.045	0.651	1.042	-0.524	-0.682	1.16E-02
ENSRNOG00000050510	LOC100363782	-0.005	-0.119	-0.397	0.096	0.298	1.17E-02
ENSRNOG00000055300	Ncapd2	0.110	0.204	0.485	-0.304	-0.529	1.17E-02
ENSRNOG00000000563	Adamts14	0.007	0.353	1.068	-0.416	-0.929	1.17E-02
ENSRNOG00000014128	Ecsit	-0.133	-0.249	-0.636	0.258	0.509	1.17E-02
ENSRNOG00000019557	Asb18	-0.834	-0.692	-1.643	0.583	1.285	1.17E-02
ENSRNOG00000001687	Crybg3	0.076	-0.247	-0.432	0.320	0.518	1.18E-02
ENSRNOG00000057834	AABR07013701.1	-0.103	0.412	1.625	-0.473	-2.175	1.18E-02
ENSRNOG00000051977	Mmrn2	-0.079	-0.007	-0.278	0.140	0.551	1.18E-02
ENSRNOG00000009104	Fnip1	0.040	-0.280	-0.745	0.117	0.574	1.18E-02

ENSRNOG00000048187	LOC691153	-0.485	0.052	-0.637	0.391	1.481	1.18E-02
ENSRNOG00000050210	RT1-CE10	-0.057	0.991	1.487	-0.351	-0.731	1.19E-02
ENSRNOG00000012181	Lpl	-0.947	-0.624	-0.402	0.597	1.044	1.19E-02
ENSRNOG00000020029	Fam195a	-0.230	-0.265	-0.789	0.225	0.761	1.19E-02
ENSRNOG00000047276	Pkm	0.162	-0.292	-1.416	0.248	0.820	1.19E-02
ENSRNOG00000027724	Plekhf1	-0.006	-0.392	-0.688	0.544	0.605	1.19E-02
ENSRNOG00000042668	Msrb3	0.048	-0.147	-0.542	0.082	0.355	1.19E-02
ENSRNOG00000014616	Iars	-0.033	-0.153	-0.364	0.084	0.324	1.19E-02
ENSRNOG00000018044	Phyh	-0.284	-0.262	-0.713	0.207	0.591	1.19E-02
ENSRNOG00000048567	Ankmy1	0.354	0.363	0.776	-1.005	-1.173	1.19E-02
ENSRNOG00000017243	Bnip3	0.071	-0.215	-0.437	0.077	0.474	1.19E-02
ENSRNOG00000028254	Tmem141	0.047	-0.213	-0.460	0.291	0.440	1.19E-02
ENSRNOG00000007060	Plin2	-0.334	0.430	0.899	-0.326	-0.744	1.20E-02
ENSRNOG00000054625	Fhod1	0.211	-0.190	-0.752	0.254	0.503	1.20E-02
ENSRNOG00000023023	Trpt1	0.046	-0.305	-0.858	0.328	0.600	1.20E-02
ENSRNOG00000053288	Ank3	0.138	-0.199	-0.654	0.069	0.486	1.20E-02
ENSRNOG00000002749	Spag9	-0.045	-0.176	-0.508	0.044	0.401	1.20E-02
ENSRNOG00000055000	Zkscan3	-0.182	-0.192	-0.398	0.194	0.264	1.21E-02
ENSRNOG00000007607	Nr4a1	-0.060	-0.699	-1.674	0.804	1.338	1.21E-02
ENSRNOG00000005326	Cnrip1	0.780	0.009	0.537	-0.324	-0.911	1.21E-02
ENSRNOG00000010940	Acad11	0.048	-0.337	-0.410	0.274	0.706	1.21E-02
ENSRNOG00000029042	Mt-nd6	-0.388	-0.277	-0.699	0.179	0.657	1.21E-02
ENSRNOG00000028384	Npepl1	0.093	-0.230	-0.612	0.239	0.416	1.21E-02
ENSRNOG00000009708	Tas1r1	-0.255	-0.570	-1.127	0.525	0.931	1.22E-02
ENSRNOG00000009155	Ndufs3	-0.118	-0.325	-0.829	0.270	0.571	1.22E-02
ENSRNOG00000019677	Arid3b	0.280	-0.341	-0.923	0.272	0.726	1.22E-02
ENSRNOG00000018114	Acadvl	-0.515	-0.289	-0.409	0.291	0.651	1.23E-02

ENSRNOG00000015191	Phc1	0.173	-0.261	-0.848	0.265	0.542	1.23E-02
ENSRNOG00000026493	Cdnf	0.354	-0.108	-1.091	0.145	0.750	1.24E-02
ENSRNOG00000021241	Gfra4	0.086	-0.284	-1.568	0.292	1.227	1.24E-02
ENSRNOG00000002258	Tmem150c	-0.247	-0.194	-0.858	0.178	0.855	1.24E-02
ENSRNOG00000020659	Mrpl4	-0.105	-0.216	-0.542	0.235	0.440	1.24E-02
ENSRNOG00000016032	Cnnm3	-0.098	-0.122	-0.534	0.237	0.411	1.24E-02
ENSRNOG00000016907	Ppp5c	-0.037	-0.157	-0.443	0.165	0.318	1.24E-02
ENSRNOG00000024494	AABR07056633.1	0.339	3.098	3.554	-2.168	-2.565	1.24E-02
ENSRNOG00000018755	Acss2	-0.256	-0.230	-0.703	0.367	0.712	1.24E-02
ENSRNOG00000007316	Idh3B	-0.236	-0.244	-0.559	0.208	0.540	1.24E-02
ENSRNOG00000005749	Foxred2	0.229	0.409	0.858	-0.413	-0.794	1.24E-02
ENSRNOG00000017120	Abhd2	-0.200	0.760	1.319	-0.988	-0.918	1.24E-02
ENSRNOG00000002303	Kcnj12	0.272	-0.356	-1.223	0.326	0.847	1.24E-02
ENSRNOG00000017035	Cep120	0.115	0.041	0.167	-0.198	-0.275	1.25E-02
ENSRNOG00000058289	AABR07027575.1	-0.045	1.088	1.593	-1.227	-1.500	1.25E-02
ENSRNOG00000020392	Stxbp3	0.117	-0.283	-0.566	0.194	0.433	1.26E-02
ENSRNOG00000008519	RGD1309079	-0.118	0.142	0.703	-0.215	-0.622	1.27E-02
ENSRNOG00000058456	AABR07051177.1	0.223	0.144	0.913	-0.246	-1.332	1.27E-02
ENSRNOG00000007461	Klhl41	0.049	0.414	0.693	-0.464	-0.526	1.27E-02
ENSRNOG00000001765	Slc51a	-0.809	2.850	3.918	-2.476	-2.629	1.27E-02
ENSRNOG00000021536	Plxdc1	-0.478	0.239	1.305	-0.321	-0.977	1.27E-02
ENSRNOG00000055608	Zfp94	-0.353	-0.481	-0.922	0.463	0.797	1.27E-02
ENSRNOG00000026286	Nhlrc1	-0.018	-0.142	-1.098	0.273	0.878	1.28E-02
ENSRNOG00000042179	Cfap20	0.025	0.040	0.413	-0.091	-0.352	1.28E-02
ENSRNOG00000003243	Wdr81	-0.059	0.204	0.446	-0.162	-0.398	1.28E-02
ENSRNOG00000007404	Ccdc23	0.012	0.321	0.517	-0.277	-0.548	1.29E-02
ENSRNOG00000012008	S100a3	-0.050	0.550	1.559	-0.894	-1.499	1.29E-02

ENSRNOG00000014316	Rapgef1	-0.099	-0.062	-0.363	0.034	0.395	1.30E-02
ENSRNOG00000013009	Ldha	0.239	-0.353	-1.098	0.344	0.702	1.33E-02
ENSRNOG00000023520	Cs	-0.221	-0.189	-0.578	0.111	0.457	1.33E-02
ENSRNOG00000038610	RGD1564688	0.294	-0.382	-1.290	0.357	1.026	1.33E-02
ENSRNOG00000025512	Mettl17	-0.026	-0.148	-0.369	0.170	0.377	1.33E-02
ENSRNOG00000004152	Lrp12	-0.003	0.120	0.254	-0.276	-0.434	1.33E-02
ENSRNOG00000011723	Slc44a3	0.530	0.803	1.875	-1.674	-1.436	1.33E-02
ENSRNOG00000018598	Ankrd1	-0.439	2.285	3.969	-2.338	-2.435	1.33E-02
ENSRNOG00000015239	Ginm1	0.327	-0.261	-0.649	0.267	0.531	1.33E-02
ENSRNOG00000023035	Smim8	0.110	-0.344	-0.628	0.298	0.453	1.33E-02
ENSRNOG00000003782	Acot9	-0.073	0.458	0.888	-0.480	-0.678	1.35E-02
ENSRNOG00000006116	Hk2	0.095	-0.309	-0.614	0.309	0.614	1.36E-02
ENSRNOG00000004473	Ppargc1a	-0.078	-0.390	-1.056	0.237	0.725	1.36E-02
ENSRNOG00000020281	Kif22	0.440	0.619	1.522	-0.555	-1.268	1.36E-02
ENSRNOG00000017940	Rere	0.040	-0.013	-0.611	0.132	0.558	1.36E-02
ENSRNOG00000034126	Pcdhga2	0.050	-0.338	-0.747	0.359	0.704	1.37E-02
ENSRNOG00000021106	Gramd1a	-0.247	-0.117	-0.323	0.113	0.360	1.37E-02
ENSRNOG00000030478	AY172581.9	-0.204	-0.286	-0.592	0.349	0.869	1.37E-02
ENSRNOG00000016327	Mcee	-0.266	-0.370	-0.663	0.434	0.629	1.37E-02
ENSRNOG00000009033	Cntn2	0.027	0.430	1.667	-0.527	-1.171	1.37E-02
ENSRNOG00000051943	AABR07030854.2	0.230	-0.192	-1.433	0.321	1.149	1.38E-02
ENSRNOG00000060905	AC111831.2	0.227	0.084	0.808	-0.671	-1.317	1.38E-02
ENSRNOG00000025679	Stk40	0.019	0.082	-0.292	-0.005	0.357	1.38E-02
ENSRNOG00000019907	Nfkbie	-0.126	1.030	1.597	-0.778	-0.811	1.39E-02
ENSRNOG00000046254	Adgre1	0.035	0.356	0.912	-0.375	-0.765	1.39E-02
ENSRNOG00000019387	Ifi30	-0.047	0.084	0.693	-0.089	-0.557	1.39E-02
ENSRNOG00000050015	LOC100910772	-0.083	0.446	1.687	-0.755	-1.900	1.39E-02

ENSRNOG00000033734	Tnnt2	-0.601	1.717	3.597	-2.478	-3.241	1.39E-02
ENSRNOG00000057129	AABR07049033.2	-0.057	-1.772	-3.185	0.894	2.265	1.39E-02
ENSRNOG00000019926	Ramp1	0.277	-0.710	-1.111	0.810	1.031	1.39E-02
ENSRNOG00000021314	Fdft1	-0.042	-0.235	-0.549	0.247	0.443	1.40E-02
ENSRNOG00000003219	Trim16	-0.336	-0.326	-0.520	0.282	0.540	1.41E-02
ENSRNOG00000001338	Hpd	-1.494	-0.473	-1.289	0.808	2.591	1.42E-02
ENSRNOG00000054390	AABR07050283.1	0.373	-0.687	-1.228	0.652	0.794	1.43E-02
ENSRNOG00000001177	Acads	-0.483	-0.260	-0.466	0.211	0.612	1.43E-02
ENSRNOG00000017608	C2cd3	0.108	0.399	0.542	-0.437	-0.527	1.43E-02
ENSRNOG00000002044	Gtpbp8	-0.084	-0.371	-0.615	0.348	0.403	1.43E-02
ENSRNOG00000015529	Cdca3	0.297	0.677	1.621	-1.536	-1.913	1.43E-02
ENSRNOG00000019219	Vamp1	-0.025	-0.163	-0.668	0.113	0.427	1.44E-02
ENSRNOG00000003895	Rgs1	1.632	1.275	1.952	-1.220	-2.050	1.44E-02
ENSRNOG00000017780	Akr7a2	-0.258	-0.106	-0.462	0.147	0.523	1.44E-02
ENSRNOG00000024577	Gamt	-0.130	-0.174	-0.732	0.242	0.617	1.44E-02
ENSRNOG00000008637	Strada	-0.014	0.172	0.368	-0.137	-0.316	1.44E-02
ENSRNOG00000043219	Fbn2	-0.424	0.015	1.251	-0.597	-1.678	1.44E-02
ENSRNOG00000014786	Ccne1	-0.185	0.124	1.444	-1.350	-1.887	1.44E-02
ENSRNOG00000009197	Asb4	0.225	-0.559	-1.590	0.309	0.953	1.45E-02
ENSRNOG00000050348	LOC684270	-0.023	-0.319	-0.535	0.244	0.424	1.45E-02
ENSRNOG00000031211	Acsm5	0.696	0.255	-0.775	0.363	1.355	1.45E-02
ENSRNOG00000005587	Suclg1	-0.136	-0.318	-0.661	0.255	0.480	1.45E-02
ENSRNOG00000005101	Ctage5	0.247	-0.477	-0.710	0.223	0.395	1.45E-02
ENSRNOG00000007300	C1qtnf6	0.339	0.042	0.800	-0.448	-1.035	1.45E-02
ENSRNOG00000018145	Crat	-0.074	-0.171	-0.461	0.120	0.442	1.45E-02
ENSRNOG00000059837	Cdkn2a	0.490	2.203	3.623	-1.313	-2.577	1.45E-02
ENSRNOG00000032703	Rasgrp3	0.554	-0.293	-1.014	0.269	0.788	1.45E-02

ENSRNOG00000000033	Tmcc2	0.243	-0.348	-1.108	0.307	0.643	1.45E-02
ENSRNOG00000012763	Cwf19l1	0.165	-0.228	-0.480	0.201	0.334	1.45E-02
ENSRNOG00000020567	Fibp	0.179	0.179	0.231	-0.176	-0.360	1.45E-02
ENSRNOG00000018716	Dennd2c	0.012	0.478	0.856	-0.628	-0.863	1.45E-02
ENSRNOG00000001915	Chodl	0.266	-0.213	0.746	-0.223	-1.177	1.45E-02
ENSRNOG00000031801	Ephb3	0.041	0.801	1.732	-0.404	-1.236	1.45E-02
ENSRNOG00000057221	Scn3b	-0.691	0.658	1.615	-0.841	-1.464	1.45E-02
ENSRNOG00000021713	Kif18b	0.036	1.405	2.132	-1.591	-2.331	1.45E-02
ENSRNOG00000014277	Zdhhc13	-0.129	0.250	0.770	-0.616	-0.692	1.45E-02
ENSRNOG00000008209	St3gal1	0.318	-0.259	-0.966	0.283	0.731	1.46E-02
ENSRNOG00000047499	Rbm15	-0.009	-0.137	-0.470	0.158	0.478	1.47E-02
ENSRNOG00000000439	Ager	-0.314	-0.225	-0.429	0.503	0.646	1.47E-02
ENSRNOG00000000913	Gusb	0.065	0.301	0.625	-0.207	-0.407	1.47E-02
ENSRNOG00000042944	Cenpw	0.098	0.082	1.941	-0.860	-2.421	1.47E-02
ENSRNOG00000019223	Atp5c1	-0.162	-0.343	-0.728	0.255	0.543	1.47E-02
ENSRNOG00000006419	Aven	0.313	-0.315	-0.715	0.316	0.500	1.47E-02
ENSRNOG00000021086	Dtx4	0.118	0.224	0.438	-0.352	-0.575	1.47E-02
ENSRNOG00000010890	Bmp1	-0.110	0.155	0.594	-0.293	-0.618	1.47E-02
ENSRNOG00000008806	Clip4	-0.068	-0.137	-0.510	0.065	0.408	1.48E-02
ENSRNOG00000016935	Mapk1ip1	0.077	-0.147	-0.492	0.181	0.441	1.48E-02
ENSRNOG00000016574	Fiz1	-0.009	-0.101	-0.401	0.053	0.314	1.48E-02
ENSRNOG00000049223	Vipas39	-0.193	0.181	0.495	-0.310	-0.418	1.48E-02
ENSRNOG00000052445	Kdm7a	-0.018	-0.314	-0.839	0.093	0.711	1.48E-02
ENSRNOG00000013585	Nmnat3	-0.148	-0.302	-0.699	0.446	0.688	1.48E-02
ENSRNOG00000016952	LOC686442	-0.087	-0.257	-0.653	0.301	0.567	1.48E-02
ENSRNOG00000015161	LOC688754	-0.104	-0.285	-0.640	0.246	0.506	1.48E-02
ENSRNOG00000014422	Gtf2e2	0.077	0.127	0.445	-0.113	-0.414	1.48E-02

ENSRNOG00000019667	Ppfibp2	-0.415	0.772	1.151	-0.565	-0.690	1.48E-02
ENSRNOG00000010947	Mmp14	0.025	0.366	1.267	-0.288	-0.922	1.48E-02
ENSRNOG00000007078	Wisp1	0.439	-0.052	1.521	-0.657	-1.447	1.48E-02
ENSRNOG00000008586	Aldh1l2	1.077	0.740	1.072	-1.593	-1.647	1.48E-02
ENSRNOG00000030102	Mboat4	0.659	1.455	2.097	-1.709	-2.579	1.48E-02
ENSRNOG00000039744	RT1-CE4	-0.062	0.811	1.165	-0.246	-0.645	1.48E-02
ENSRNOG00000003590	Tom1l2	-0.273	-0.219	-0.702	0.091	0.570	1.48E-02
ENSRNOG00000002238	Sema5b	-0.136	-0.188	-0.393	0.315	0.822	1.49E-02
ENSRNOG00000006206	Tmem106b	-0.369	-0.396	-0.483	0.253	0.621	1.49E-02
ENSRNOG00000000700	Tmem119	0.337	0.779	2.131	-1.029	-1.732	1.49E-02
ENSRNOG00000011702	Foxp3	0.398	-0.690	-2.136	0.706	1.418	1.49E-02
ENSRNOG00000016244	Mical2	0.040	0.288	-1.028	0.092	1.214	1.49E-02
ENSRNOG00000024410	Blvrb	-0.272	0.498	1.002	-0.319	-0.509	1.49E-02
ENSRNOG00000038622	Zfp628	-0.105	-0.141	-0.609	0.148	0.489	1.50E-02
ENSRNOG00000045816	Gria1	0.909	0.246	1.257	-1.408	-2.304	1.50E-02
ENSRNOG00000036742	Uqcrc2	-0.256	-0.309	-0.589	0.211	0.563	1.51E-02
ENSRNOG00000060707	Atg2a	-0.047	-0.105	-0.604	0.124	0.449	1.51E-02
ENSRNOG00000006858	Pyurfl1	-0.069	-0.268	-0.538	0.248	0.443	1.51E-02
ENSRNOG00000002908	Slc35a4	-0.062	-0.094	-0.411	0.085	0.308	1.51E-02
ENSRNOG00000012513	Pdk3	0.142	0.145	0.888	-0.186	-0.729	1.51E-02
ENSRNOG00000024178	Cenpt	0.574	0.952	1.594	-1.962	-2.011	1.51E-02
ENSRNOG00000002332	Mospd1	0.178	-0.232	-0.679	0.059	0.460	1.52E-02
ENSRNOG00000004612	Ppp1cb	0.060	-0.426	-0.693	0.240	0.527	1.52E-02
ENSRNOG00000004103	Trappc6b	0.228	-0.295	-0.569	0.247	0.364	1.53E-02
ENSRNOG00000033608	Cd276	-0.055	0.210	0.764	-0.323	-0.611	1.53E-02
ENSRNOG00000038406	Tmem132b	1.004	0.819	-1.623	0.064	2.149	1.54E-02
ENSRNOG00000007646	Sipa1l1	0.064	-0.096	-0.527	0.158	0.479	1.55E-02

ENSRNOG00000005016	Tmed4	0.157	-0.150	-0.381	0.173	0.316	1.55E-02
ENSRNOG00000019751	Cyb5r2	-0.240	0.593	0.921	-0.624	-0.664	1.55E-02
ENSRNOG00000029465	Slc26a10	-0.185	-0.276	-0.400	0.379	0.921	1.56E-02
ENSRNOG00000010984	Anxa11	-0.237	-0.041	-0.117	0.129	0.282	1.57E-02
ENSRNOG00000025808	Aars2	-0.107	-0.305	-0.829	0.235	0.551	1.57E-02
ENSRNOG00000011849	Ndufs1	-0.316	-0.307	-0.726	0.197	0.660	1.57E-02
ENSRNOG00000030700	Mt-cox3	-0.341	-0.361	-0.682	0.276	0.649	1.58E-02
ENSRNOG00000032398	RGD1562136	-0.058	-0.308	-0.835	0.202	0.496	1.58E-02
ENSRNOG00000011419	Aldh6a1	-0.177	-0.094	-0.369	0.059	0.478	1.58E-02
ENSRNOG00000050197	Pdia6	0.135	-0.017	0.141	-0.004	-0.275	1.58E-02
ENSRNOG00000017445	Tubb2b	-0.247	0.156	0.970	-0.229	-0.753	1.58E-02
ENSRNOG00000013720	Aebp1	0.748	0.361	1.198	-0.466	-0.969	1.58E-02
ENSRNOG00000052477	Tbrg4	-0.357	-0.202	-0.335	0.249	0.453	1.58E-02
ENSRNOG00000049768	Adcy9	0.034	-0.165	-0.905	0.051	0.672	1.58E-02
ENSRNOG00000016201	Mrps9	-0.289	-0.213	-0.420	0.202	0.399	1.58E-02
ENSRNOG00000012303	Apobec2	-0.435	-0.330	-0.525	0.333	0.700	1.59E-02
ENSRNOG00000013603	Dffa	-0.194	-0.383	-0.834	0.205	0.715	1.60E-02
ENSRNOG00000003873	Cpd	-0.148	-0.242	-0.657	0.244	0.608	1.60E-02
ENSRNOG00000050044	Ptp4a2	0.091	-0.179	-0.878	0.102	0.556	1.60E-02
ENSRNOG00000000513	Mapk14	0.006	-0.109	-0.537	0.198	0.465	1.60E-02
ENSRNOG00000003139	Smc1a	-0.063	0.181	0.318	-0.155	-0.275	1.60E-02
ENSRNOG00000037364	AABR07003049.1	0.057	0.441	0.544	-0.402	-0.499	1.61E-02
ENSRNOG00000059061	Uqcr10	-0.075	-0.317	-0.644	0.356	0.542	1.61E-02
ENSRNOG00000029799	Tomm34	-0.098	0.335	0.229	-0.345	-0.378	1.61E-02
ENSRNOG00000042869	LOC100360002	-0.080	-0.365	-0.674	0.249	0.576	1.62E-02
ENSRNOG00000007590	Eya1	0.249	-0.214	-1.067	0.137	0.603	1.62E-02
ENSRNOG00000024186	Eef1b2	0.153	0.062	0.463	-0.089	-0.459	1.62E-02

ENSRNOG00000012457	Cyc1	-0.282	-0.256	-0.695	0.223	0.578	1.63E-02
ENSRNOG00000057470	Pla2g12a	-0.742	-0.348	-0.568	0.402	1.034	1.63E-02
ENSRNOG00000003143	RGD1310166	0.000	1.808	3.205	-1.313	-2.618	1.64E-02
ENSRNOG00000003984	Apln	-0.110	0.073	0.756	-0.406	-1.128	1.64E-02
ENSRNOG00000015264	Lrrc38	0.332	-0.616	-1.856	0.689	1.443	1.64E-02
ENSRNOG00000019850	Speg	0.107	-0.096	-0.808	0.135	0.613	1.64E-02
ENSRNOG00000059461	B4galt1	-0.020	0.237	0.416	-0.252	-0.349	1.64E-02
ENSRNOG00000055471	Ywhah	0.039	0.101	0.242	-0.156	-0.306	1.64E-02
ENSRNOG00000034234	Mt-co1	-0.413	-0.386	-0.613	0.307	0.681	1.64E-02
ENSRNOG00000007837	Acot11	-0.627	-0.350	-0.908	0.587	1.212	1.65E-02
ENSRNOG00000056139	AABR07016621.1	0.135	-0.307	-1.157	0.522	0.986	1.65E-02
ENSRNOG00000000316	Sobp	0.255	-0.339	-0.952	0.345	0.663	1.65E-02
ENSRNOG00000014723	Cbfa2t3	-0.101	-0.181	-0.574	0.356	0.548	1.65E-02
ENSRNOG00000009049	Usp11	-0.307	0.449	0.967	-0.368	-0.703	1.65E-02
ENSRNOG00000023064	Catsper2	0.056	0.339	0.651	-0.653	-0.642	1.65E-02
ENSRNOG00000052574	Lbr	0.074	-0.056	-0.260	0.065	0.294	1.65E-02
ENSRNOG00000011161	Slc2a12	0.168	-0.263	-0.939	0.210	0.588	1.65E-02
ENSRNOG00000017156	Ankle1	0.357	0.965	1.713	-2.037	-2.288	1.66E-02
ENSRNOG00000019862	Pinlyp	0.512	0.724	1.170	-0.456	-1.164	1.66E-02
ENSRNOG00000021155	Ctsk	0.145	0.238	1.092	-0.373	-0.908	1.67E-02
ENSRNOG00000013727	Ndc80	0.316	0.378	1.528	-0.674	-1.410	1.68E-02
ENSRNOG00000029055	Ttk	0.131	0.794	1.312	-1.717	-2.223	1.69E-02
ENSRNOG00000036841	Cbx5	-0.033	0.087	0.293	-0.162	-0.359	1.69E-02
ENSRNOG00000000895	Rabgef1	0.301	-0.260	-0.985	0.179	0.610	1.69E-02
ENSRNOG00000022980	Sdhd	-0.124	-0.321	-0.644	0.276	0.515	1.69E-02
ENSRNOG00000037080	Adamts17	-0.187	0.708	1.833	-1.865	-2.437	1.69E-02
ENSRNOG00000022772	Prickle1	-0.026	-0.199	-0.344	0.293	0.454	1.70E-02

ENSRNOG00000021602	Zmynd10	-0.181	-0.170	-0.701	0.350	0.623	1.70E-02
ENSRNOG00000033389	Susd2	-0.127	-0.178	-0.364	0.332	0.600	1.71E-02
ENSRNOG00000029570	Fam222b	0.024	-0.204	-0.498	0.273	0.338	1.71E-02
ENSRNOG00000016686	Tmem167a	0.227	0.103	0.268	-0.186	-0.388	1.71E-02
ENSRNOG00000008329	Ndufb11	-0.052	-0.331	-0.712	0.356	0.530	1.71E-02
ENSRNOG00000016789	Actr1b	-0.011	0.056	0.315	-0.105	-0.339	1.72E-02
ENSRNOG00000014997	Igf2r	0.125	0.210	0.398	-0.279	-0.466	1.72E-02
ENSRNOG00000043381	Cyth1	-0.298	-0.096	-0.138	0.170	0.358	1.73E-02
ENSRNOG00000000987	Ptcd1	-0.099	-0.026	-0.192	0.094	0.280	1.73E-02
ENSRNOG00000008609	Capn3	0.047	-0.181	-0.511	0.090	0.357	1.73E-02
ENSRNOG00000015695	Sult5a1	0.839	0.344	0.739	-0.506	-1.221	1.73E-02
ENSRNOG00000011316	Fam167a	0.083	0.793	1.765	-0.532	-1.453	1.73E-02
ENSRNOG00000016356	Got1	-0.463	-0.408	-0.787	0.377	0.760	1.73E-02
ENSRNOG00000061216	Tubb5	-0.109	0.340	0.842	-0.287	-0.521	1.73E-02
ENSRNOG00000010881	Trak2	-0.142	-0.291	-0.754	0.107	0.529	1.73E-02
ENSRNOG00000012390	Npw	-0.331	0.293	2.191	-1.230	-2.234	1.73E-02
ENSRNOG00000002052	Ccdc80	0.693	0.613	1.297	-1.026	-1.147	1.73E-02
ENSRNOG00000031273	RGD1559972	0.134	0.031	0.588	-0.008	-0.502	1.74E-02
ENSRNOG00000043150	Arfgap1	0.164	-0.240	-0.412	0.256	0.307	1.75E-02
ENSRNOG00000014013	Map4k4	-0.097	0.271	0.555	-0.335	-0.413	1.75E-02
ENSRNOG00000000903	Asl	-0.101	0.253	0.446	-0.185	-0.405	1.77E-02
ENSRNOG00000030644	Mt-nd1	-0.395	-0.330	-0.706	0.331	0.738	1.77E-02
ENSRNOG00000018815	Plk1	0.358	0.975	1.276	-1.111	-1.627	1.77E-02
ENSRNOG00000011782	Got2	-0.267	-0.132	-0.445	0.167	0.456	1.77E-02
ENSRNOG00000021150	Plcb3	-0.201	-0.036	-0.161	0.194	0.328	1.77E-02
ENSRNOG00000002207	Guf1	-0.036	-0.463	-0.702	0.226	0.442	1.77E-02
ENSRNOG00000001149	Pxn	0.054	-0.033	-0.387	0.107	0.329	1.77E-02

ENSRNOG00000057676 Polr2m 0.039 -0.264 -0.655 0.180
0.375 1.77E-02
ENSRNOG00000024101 Phkb 0.193 -0.178 -1.142 0.116 0.720
1.78E-02
ENSRNOG00000009862 Olfm1 0.101 0.410 0.967 -0.240 -0.720
1.79E-02
ENSRNOG00000004629 Fkbp3 0.018 -0.350 -0.536 0.286 0.476
1.79E-02
ENSRNOG00000005865 Ssfa2 -0.146 -0.137 -0.171 0.181
0.344 1.79E-02
ENSRNOG00000024580 Mamstr 0.151 0.792 0.995 -0.531 -0.808
1.79E-02
ENSRNOG00000010277 Idh3a 0.003 -0.313 -0.933 0.180 0.570
1.80E-02
ENSRNOG00000049020 Tceal3 0.973 1.250 2.015 -1.325 -2.401
1.80E-02
ENSRNOG00000022500 Rwdd4 -0.060 -0.233 -0.415 0.142
0.290 1.81E-02
ENSRNOG00000015295 Ctdsp1 0.124 -0.109 -0.320 0.232
0.283 1.81E-02
ENSRNOG00000014658 Zfp423 0.118 -0.020 -0.631 0.101
0.536 1.81E-02
ENSRNOG00000014530 Nav2 -0.341 0.359 0.714 -0.239 -0.635
1.81E-02
ENSRNOG00000020075 Eef1g 0.208 0.053 0.187 -0.144 -0.365
1.81E-02
ENSRNOG00000017029 Snx1 -0.024 0.118 0.423 -0.172 -0.376
1.81E-02
ENSRNOG00000050655 P4ha1 0.196 -0.369 -0.695 0.306 0.433
1.82E-02
ENSRNOG00000051854 Enpep 0.046 -0.316 -0.448 0.163 0.585
1.82E-02
ENSRNOG00000032293 Polg -0.043 -0.126 -0.400 0.183
0.373 1.83E-02
ENSRNOG00000014691 Ric3 0.417 0.589 -0.216 0.025 0.766 1.83E-02

ENSRNOG00000017941 Optn 0.056 -0.030 -0.457 -0.041
0.315 1.83E-02
ENSRNOG00000020723 Pten 0.018 0.239 0.623 -0.245 -0.464
1.83E-02
ENSRNOG00000006142 Tmem8c -0.628 2.601 3.452 -2.567 -
2.518 1.84E-02
ENSRNOG00000023683 Nog 0.316 0.047 -0.724 0.287 1.033 1.84E-02

ENSRNOG00000061299 AC113925.2 -0.741 -0.619 -1.062
0.616 1.032 1.84E-02
ENSRNOG00000029996 LOC100360150 -0.004 0.213 1.374 -0.364
-0.798 1.84E-02
ENSRNOG00000018117 Ndufv1 -0.331 -0.213 -0.653
0.213 0.554 1.84E-02
ENSRNOG00000012258 Rras2 -0.108 0.221 0.701 -0.396 -0.680
1.84E-02
ENSRNOG00000013463 Kcnj8 -0.399 -0.302 -0.502 0.429
0.760 1.84E-02

ENSRNOG00000057759	AABR07027854.1	-0.035	0.080	0.469	-0.073	-0.385	1.84E-02
ENSRNOG00000051816	LOC502894	0.162	-0.291	-0.833	0.102	0.421	1.85E-02
ENSRNOG00000007895	Pdhb	-0.303	-0.277	-0.615	0.201	0.504	1.85E-02
ENSRNOG00000023337	Sema3a	0.811	1.150	2.506	-1.225	-1.850	1.85E-02
ENSRNOG00000029910	Golga4	-0.128	-0.118	-0.412	0.157	0.411	1.85E-02
ENSRNOG00000012495	Podxl	-0.104	-0.290	-0.526	0.203	0.576	1.85E-02
ENSRNOG00000009930	Pigo	-0.033	-0.214	-0.656	0.101	0.488	1.85E-02
ENSRNOG00000010673	Eral1	-0.051	-0.163	-0.467	0.161	0.318	1.86E-02
ENSRNOG00000009314	Ivl	0.026	-0.015	2.411	0.027	-2.457	1.86E-02
ENSRNOG00000007378	Acox2	-0.497	-0.303	-1.219	0.453	1.315	1.87E-02
ENSRNOG00000015540	Ppp6r3	-0.034	-0.110	-0.377	0.048	0.272	1.87E-02
ENSRNOG00000015794	Fam83d	0.449	0.801	2.393	-0.943	-2.594	1.87E-02
ENSRNOG00000019295	Rab12	0.090	0.153	0.369	-0.187	-0.357	1.87E-02
ENSRNOG00000016161	Lman2	0.068	-0.240	-0.693	0.275	0.439	1.87E-02
ENSRNOG00000015033	Mxd4	-0.002	0.203	0.473	-0.080	-0.272	1.87E-02
ENSRNOG00000022730	Zfp57	-0.023	0.186	1.995	-0.696	-2.018	1.87E-02
ENSRNOG00000019022	Fam89a	-0.227	-0.330	-0.479	0.577	0.813	1.88E-02
ENSRNOG00000009298	Fbxo44	-0.212	-0.291	-0.739	0.348	0.764	1.88E-02
ENSRNOG00000029971	Mt-nd5	-0.373	-0.310	-0.721	0.184	0.731	1.88E-02
ENSRNOG00000012873	Gfm1	-0.159	-0.250	-0.596	0.120	0.397	1.88E-02
ENSRNOG00000016917	Clcn1	0.166	-0.194	-1.067	0.193	0.700	1.88E-02
ENSRNOG00000014480	Sys1	0.202	-0.254	-0.730	0.384	0.529	1.88E-02
ENSRNOG00000031485	Dpp3	-0.020	0.279	0.398	-0.389	-0.348	1.89E-02
ENSRNOG00000013179	Tinagl1	-0.110	-0.346	-0.534	0.428	0.767	1.89E-02
ENSRNOG00000012494	Kctd14	0.288	0.156	0.765	-0.208	-0.828	1.89E-02
ENSRNOG00000016678	Angptl2	0.166	0.137	0.369	-0.223	-0.481	1.89E-02
ENSRNOG00000012434	Zfp598	0.113	-0.100	-0.400	0.131	0.332	1.90E-02

ENSRNOG00000014214	Rpl27a	0.100	0.079	0.485	-0.053	-0.458	1.90E-02
ENSRNOG00000008837	Ass1	-0.274	-0.330	-0.665	0.706	0.968	1.91E-02
ENSRNOG00000001707	Vwa5b2	-0.208	-0.227	-0.464	0.219	0.608	1.92E-02
ENSRNOG00000019485	Bckdk	-0.070	-0.172	-0.456	0.192	0.339	1.92E-02
ENSRNOG00000023278	Sec61a2	0.091	0.196	0.204	-0.133	-0.356	1.92E-02
ENSRNOG00000018276	Mgat4a	0.007	-0.055	-0.102	0.259	0.495	1.92E-02
ENSRNOG00000013837	Tp53bp1	-0.238	0.153	0.399	-0.441	-0.519	1.92E-02
ENSRNOG00000027087	Tmem186	-0.064	-0.109	-0.341	0.075	0.277	1.93E-02
ENSRNOG00000017342	Zdhhc7	-0.026	-0.045	-0.320	0.205	0.270	1.93E-02
ENSRNOG00000002517	Pdc	-0.280	-0.640	-2.379	0.878	1.990	1.93E-02
ENSRNOG00000038300	Gstt4	0.605	2.278	3.066	-1.224	-2.529	1.94E-02
ENSRNOG00000047306	Stap2	-0.041	1.021	1.810	-0.934	-1.162	1.95E-02
ENSRNOG00000003625	Ubxn4	-0.077	0.190	0.465	-0.258	-0.383	1.95E-02
ENSRNOG00000026941	Tril	-0.175	0.011	0.464	-0.168	-0.548	1.95E-02
ENSRNOG00000045604	AC139391.1	0.185	-0.598	-1.919	0.423	1.434	1.95E-02
ENSRNOG00000008051	Itpk1	-0.209	0.014	-0.300	0.255	0.518	1.95E-02
ENSRNOG00000017545	Mrs2	-0.180	-0.235	-0.599	0.129	0.440	1.95E-02
ENSRNOG00000012827	Mlf1	0.397	-0.653	-1.318	0.437	0.740	1.96E-02
ENSRNOG00000056894	St6galnac3	-0.321	-0.237	-0.606	0.368	0.691	1.96E-02
ENSRNOG00000003653	Fh	-0.249	-0.414	-0.732	0.190	0.529	1.96E-02
ENSRNOG00000015928	Dhx35	-0.217	-0.223	-0.676	0.230	0.462	1.96E-02
ENSRNOG00000022249	Mllt10	0.000	-0.071	-0.358	0.056	0.334	1.96E-02
ENSRNOG00000028674	Fbxw5	-0.161	-0.162	-0.485	0.124	0.332	1.96E-02
ENSRNOG00000026527	Snx32	0.099	0.602	0.765	-0.466	-0.653	1.97E-02
ENSRNOG00000058478	Mapk8ip1	0.237	-0.272	-0.659	0.386	0.537	1.97E-02
ENSRNOG00000002763	Ulk2	-0.015	-0.166	-0.460	0.061	0.359	1.97E-02
ENSRNOG00000001681	Dscr3	-0.012	0.232	0.503	-0.217	-0.413	1.97E-02

ENSRNOG00000022490	AC115341.1	-0.092	-0.344	-0.668	0.236	0.541	1.98E-02
ENSRNOG00000018371	Tubb6	-0.294	1.589	2.392	-1.285	-1.271	1.98E-02
ENSRNOG00000016740	Fam210a	-0.367	-0.327	-0.692	0.154	0.557	1.99E-02
ENSRNOG00000015456	Zfp787	-0.102	-0.170	-0.550	0.122	0.405	1.99E-02
ENSRNOG00000002713	Zfp672	-0.039	-0.165	-0.380	0.162	0.311	1.99E-02
ENSRNOG00000026049	Qrsl1	-0.110	-0.310	-0.761	0.223	0.519	2.00E-02
ENSRNOG00000014785	Ykt6	0.051	0.069	0.240	-0.201	-0.226	2.00E-02
ENSRNOG00000005496	Ing3	0.162	0.053	0.113	-0.307	-0.346	2.00E-02
ENSRNOG00000010331	Ctsb	0.000	0.202	0.705	-0.105	-0.387	2.00E-02
ENSRNOG00000053502	Arhgef17	0.169	0.229	0.323	-0.091	-0.396	2.00E-02
ENSRNOG00000034246	Rps27a-ps1	0.168	0.034	0.462	-0.127	-0.469	2.00E-02
ENSRNOG00000020440	Fads2	-0.061	-0.039	0.444	0.023	-0.598	2.00E-02
ENSRNOG00000020688	Ccdc51	-0.308	-0.232	-0.571	0.343	0.482	2.00E-02
ENSRNOG00000060934	Ptx4	0.526	-0.073	1.716	-0.931	-2.858	2.00E-02
ENSRNOG00000054896	Pex13	-0.007	-0.169	-0.535	0.206	0.397	2.01E-02
ENSRNOG00000018604	Tufm	-0.204	-0.173	-0.476	0.209	0.443	2.01E-02
ENSRNOG00000043094	Oxct1	-0.374	-0.571	-0.985	0.398	0.942	2.01E-02
ENSRNOG00000004107	Rpl23	0.128	0.059	0.424	-0.081	-0.397	2.02E-02
ENSRNOG00000056325	Mocs2	0.143	-0.383	-0.782	0.224	0.516	2.02E-02
ENSRNOG00000056714	Sla	0.130	0.357	0.889	-0.358	-0.712	2.03E-02
ENSRNOG00000021005	Mrpl16	-0.049	-0.332	-0.531	0.284	0.487	2.04E-02
ENSRNOG00000000281	Prodh	-0.140	-0.622	-0.936	0.421	1.028	2.04E-02
ENSRNOG00000012944	Casp9	-0.193	0.170	0.430	-0.309	-0.415	2.04E-02
ENSRNOG00000013934	St5	-0.332	0.303	0.656	-0.376	-0.493	2.04E-02
ENSRNOG00000023485	Ushbp1	-0.207	-0.223	-0.303	0.224	0.521	2.04E-02
ENSRNOG00000012645	Mecom	-0.209	-0.141	-0.283	0.195	0.477	2.05E-02
ENSRNOG00000015495	Slc25a37	-0.174	0.348	0.759	-0.580	-0.638	2.05E-02

ENSRNOG00000009785	Cdkn3	0.523	1.040	1.828	-1.125	-1.849	2.07E-02
ENSRNOG00000059878	Rad51ap1	0.124	0.193	0.479	-0.307	-0.717	2.08E-02
ENSRNOG00000026842	Nnt	-0.604	-0.227	-0.496	0.207	0.735	2.08E-02
ENSRNOG00000027900	Glt6d1	0.881	0.299	0.844	-0.982	-1.695	2.08E-02
ENSRNOG00000009719	C2cd2l	-0.012	-0.176	-0.401	0.186	0.390	2.08E-02
ENSRNOG00000007859	Cep83	0.048	-0.339	-0.351	0.192	0.289	2.08E-02
ENSRNOG00000001044	Aimp2	0.020	-0.240	-0.622	0.219	0.347	2.08E-02
ENSRNOG00000014522	Mlycd	-0.378	-0.278	-0.189	0.173	0.608	2.09E-02
ENSRNOG00000046330	Rnf130	0.201	-0.146	-0.328	0.227	0.284	2.09E-02
ENSRNOG00000042592	Rgs10	0.015	0.233	0.959	-0.056	-0.545	2.09E-02
ENSRNOG00000029449	Ror1	0.021	-0.168	0.480	-0.327	-0.622	2.09E-02
ENSRNOG00000016405	Pcsk4	-0.259	-0.062	-0.543	0.116	0.749	2.09E-02
ENSRNOG00000014963	Adgrg1	-0.125	-0.307	-0.501	0.241	0.621	2.09E-02
ENSRNOG00000012393	S100a13	0.072	0.270	0.485	-0.394	-0.506	2.09E-02
ENSRNOG00000053026	Shcbp1	-0.247	-0.176	1.318	-0.374	-1.831	2.10E-02
ENSRNOG00000003897	Col1a1	0.919	0.257	0.946	-0.912	-1.304	2.11E-02
ENSRNOG00000054017	Hist1h4b	0.287	0.318	0.620	-0.320	-0.644	2.12E-02
ENSRNOG00000002216	Srd5a3	0.127	0.207	0.381	-0.273	-0.314	2.14E-02
ENSRNOG00000054689	Cox7b	-0.157	-0.360	-0.640	0.294	0.597	2.14E-02
ENSRNOG00000057153	Pla1a	0.454	0.200	0.525	-0.224	-0.431	2.14E-02
ENSRNOG00000019745	Actn3	0.727	-0.289	-1.418	0.350	0.970	2.14E-02
ENSRNOG00000010838	Araf	0.089	-0.167	-0.329	0.121	0.281	2.15E-02
ENSRNOG00000015150	Spg7	-0.244	-0.073	-0.415	0.061	0.363	2.15E-02
ENSRNOG00000021292	AABR07028027.1	-0.014	0.087	0.394	-0.079	-0.267	2.15E-02
ENSRNOG00000003594	Tmem183a	0.080	0.073	0.326	-0.152	-0.352	2.15E-02
ENSRNOG00000001385	Plbd2	0.131	0.457	0.538	-0.414	-0.411	2.15E-02
ENSRNOG00000020657	Shc1	-0.056	0.240	0.489	-0.180	-0.281	2.15E-02

ENSRNOG00000017621	Spns1	0.274	-0.066	-0.620	0.251	0.484	2.16E-02
ENSRNOG00000006868	Ube2z	-0.080	0.109	0.380	-0.157	-0.276	2.16E-02
ENSRNOG00000006557	Cyfip2	-0.039	0.233	0.835	-0.320	-0.676	2.16E-02
ENSRNOG00000004686	Spop	0.236	-0.220	-0.574	0.113	0.293	2.17E-02
ENSRNOG00000053285	Mllt6	-0.105	-0.105	-0.399	0.204	0.485	2.18E-02
ENSRNOG00000010843	Nhlrc3	0.066	0.274	0.524	-0.352	-0.414	2.18E-02
ENSRNOG00000014742	Ankrd13b	-0.175	0.024	-0.423	-0.049	0.470	2.18E-02
ENSRNOG00000019662	Tm6sf1	-0.279	0.114	0.982	-0.130	-0.672	2.19E-02
ENSRNOG00000024879	Polrmt	-0.012	0.014	-0.371	0.077	0.389	2.19E-02
ENSRNOG00000054251	Clec7a	-0.203	0.895	2.029	-0.067	-1.178	2.19E-02
ENSRNOG00000013505	Vdac2	-0.005	-0.166	-0.460	0.182	0.357	2.19E-02
ENSRNOG00000018366	RGD1310819	0.198	2.137	3.033	-1.888	-2.693	2.19E-02
ENSRNOG00000046231	Cacna1s	0.424	-0.305	-1.005	0.238	0.557	2.20E-02
ENSRNOG00000017481	Sucla2	-0.161	-0.292	-0.626	0.148	0.481	2.21E-02
ENSRNOG00000018502	Mon1a	0.043	-0.059	-0.377	0.131	0.374	2.21E-02
ENSRNOG00000048321	Tnfsf8	1.480	1.160	1.699	-1.653	-1.687	2.22E-02
ENSRNOG00000017577	Bphl	-0.105	-0.674	-0.734	0.595	0.650	2.22E-02
ENSRNOG00000037238	Rnmtl1	-0.341	-0.121	-0.377	0.107	0.464	2.22E-02
ENSRNOG00000015177	Sun2	0.085	-0.015	-0.388	0.182	0.362	2.22E-02
ENSRNOG00000017419	Map3k4	-0.059	-0.178	-0.404	0.197	0.325	2.22E-02
ENSRNOG00000001834	Mzt2b	0.130	-0.180	-0.381	0.165	0.309	2.22E-02
ENSRNOG00000003890	Nap1l1	0.021	0.178	0.663	-0.180	-0.436	2.22E-02
ENSRNOG00000022953	Ccdc163	-0.065	0.136	0.827	-0.496	-0.961	2.22E-02
ENSRNOG00000033815	AC142181.1	0.282	0.604	2.041	-0.226	-1.494	2.22E-02
ENSRNOG00000004706	Vit	-0.044	-0.743	-0.693	0.967	1.242	2.22E-02
ENSRNOG00000027228	Mogat2	0.027	-0.223	-1.171	0.188	0.954	2.22E-02
ENSRNOG00000004078	Eno3	0.171	-0.281	-1.258	0.298	0.772	2.22E-02

ENSRNOG00000013048	Pde7a	0.061	1.015	1.260	-0.540	-0.863	2.22E-02
ENSRNOG00000006615	Mtap	0.143	0.009	0.238	-0.056	-0.363	2.22E-02
ENSRNOG00000013967	Blnk	-0.491	0.570	1.746	-0.351	-1.180	2.22E-02
ENSRNOG00000002823	Mapk9	0.084	-0.177	-0.478	0.109	0.331	2.23E-02
ENSRNOG00000052289	Ptdss1	0.048	-0.021	0.172	-0.123	-0.263	2.24E-02
ENSRNOG00000016891	Uimc1	0.096	0.145	0.354	-0.107	-0.341	2.24E-02
ENSRNOG00000014987	Mdfi	0.192	0.149	1.163	-0.636	-1.302	2.24E-02
ENSRNOG00000019306	Syt12	-0.016	0.538	2.542	-0.821	-2.516	2.26E-02
ENSRNOG00000002459	Fbxo40	0.159	0.084	-0.599	-0.051	0.445	2.26E-02
ENSRNOG00000060141	Epdr1	-0.467	-0.327	-0.592	0.215	0.490	2.27E-02
ENSRNOG00000008046	Tmem30b	0.417	0.079	1.507	-0.888	-2.275	2.27E-02
ENSRNOG00000016029	Rb1	0.116	0.260	0.531	-0.344	-0.497	2.27E-02
ENSRNOG00000045655	LOC100911774	0.200	0.426	0.726	-0.403	-0.880	2.28E-02
ENSRNOG00000009538	Etfdh	-0.404	-0.349	-0.383	0.171	0.563	2.29E-02
ENSRNOG00000015142	Timm21	-0.122	-0.216	-0.435	0.198	0.463	2.29E-02
ENSRNOG00000011158	Ppp2r2a	-0.115	-0.067	-0.234	0.060	0.232	2.29E-02
ENSRNOG00000010011	Osbpl3	-0.506	0.785	1.231	-0.963	-0.949	2.29E-02
ENSRNOG00000013437	LOC691083	-0.011	-0.616	-1.764	0.529	1.275	2.30E-02
ENSRNOG00000021380	Fads6	-0.103	0.101	-0.432	0.126	0.544	2.30E-02
ENSRNOG00000010309	Nfyb	0.065	-0.179	-0.478	0.068	0.359	2.30E-02
ENSRNOG00000009112	Pon2	-0.030	0.030	0.404	-0.272	-0.405	2.30E-02
ENSRNOG00000027730	Nxpe1	-0.121	-0.287	-0.456	0.308	0.597	2.31E-02
ENSRNOG00000018400	Golm1	0.115	0.059	-0.520	0.278	0.798	2.32E-02
ENSRNOG00000002959	Shroom4	-0.116	-0.249	-0.470	0.123	0.545	2.32E-02
ENSRNOG00000033639	Zfp777	0.061	-0.110	-0.535	0.184	0.351	2.32E-02
ENSRNOG00000024429	Peg12	0.755	0.483	1.199	-0.987	-1.430	2.32E-02
ENSRNOG00000057125	Ddr1	-0.010	0.472	0.766	-0.446	-0.576	2.33E-02

ENSRNOG00000014383	AABR07070456.1	0.095	-0.280	-1.090	0.511	0.883	2.33E-02
ENSRNOG00000020060	Atf5	0.063	-0.082	-0.329	0.186	0.560	2.33E-02
ENSRNOG00000007230	Kank3	-0.230	-0.270	-0.357	0.395	0.557	2.33E-02
ENSRNOG00000009222	Epha2	0.031	0.071	-0.204	0.120	0.525	2.33E-02
ENSRNOG00000048095	RGD1564801	0.025	0.065	0.497	0.083	-0.530	2.33E-02
ENSRNOG00000016475	Nt5c3b	-0.170	0.189	0.579	-0.162	-0.468	2.33E-02
ENSRNOG00000010719	Exosc10	-0.075	-0.028	-0.169	0.093	0.240	2.33E-02
ENSRNOG00000000451	RT1-Ba	0.281	0.654	0.991	-0.671	-0.849	2.33E-02
ENSRNOG00000029178	Abcc5	-0.148	-0.046	-0.649	0.133	0.476	2.34E-02
ENSRNOG00000015810	Trip13	0.523	0.598	1.307	-1.081	-1.720	2.34E-02
ENSRNOG00000032885	Cycs	-0.053	-0.452	-0.917	0.230	0.602	2.34E-02
ENSRNOG00000000893	Tmem248	0.376	-0.250	-0.763	0.246	0.462	2.36E-02
ENSRNOG00000030416	Zfp870	0.000	-0.225	-0.674	0.187	0.465	2.36E-02
ENSRNOG00000023086	Arpp19	-0.098	0.490	1.065	-0.577	-0.861	2.37E-02
ENSRNOG00000017826	Mtrr	0.057	-0.108	-0.286	0.040	0.252	2.38E-02
ENSRNOG00000028753	B9d2	-0.098	0.332	0.654	-0.265	-0.447	2.38E-02
ENSRNOG00000020995	Fut1	0.125	0.479	0.587	-0.892	-1.421	2.38E-02
ENSRNOG00000017235	Atp6v0d1	0.123	0.095	0.214	-0.070	-0.218	2.38E-02
ENSRNOG00000009000	Foxj2	-0.131	-0.046	-0.371	0.057	0.337	2.39E-02
ENSRNOG00000027455	RGD1564804	0.067	0.041	0.447	-0.021	-0.436	2.39E-02
ENSRNOG00000031641	Rpl35a	1.130	0.602	1.123	-2.044	-1.848	2.39E-02
ENSRNOG00000051401	AABR07049156.1	-0.464	2.178	3.405	-1.611	-2.725	2.39E-02
ENSRNOG00000017883	Camsap1	-0.010	-0.108	-0.406	0.191	0.323	2.40E-02
ENSRNOG00000008569	Ndufa6	-0.066	-0.305	-0.524	0.293	0.460	2.40E-02
ENSRNOG00000019940	Ssr2	0.186	0.019	0.232	-0.064	-0.307	2.40E-02
ENSRNOG00000025370	Zfp385c	-0.123	0.443	1.495	-0.286	-1.477	2.40E-02
ENSRNOG00000020299	Klc2	-0.041	-0.094	-0.432	0.161	0.291	2.41E-02

ENSRNOG00000004189	Helb	-0.096	0.264	0.535	-0.404	-0.521	2.41E-02
ENSRNOG00000011654	Plk4	0.225	0.857	1.307	-0.953	-1.129	2.41E-02
ENSRNOG00000020086	Aar2	-0.097	-0.085	-0.182	0.147	0.243	2.41E-02
ENSRNOG00000010119	Zmat3	-0.040	0.079	0.554	-0.399	-0.563	2.41E-02
ENSRNOG00000008830	Nfe2l1	0.084	0.563	0.573	-0.457	-0.435	2.41E-02
ENSRNOG00000008218	Atp6v0e2	0.120	0.233	0.257	-0.127	-0.290	2.41E-02
ENSRNOG00000019283	P2ry2	0.035	-0.288	-0.588	0.210	0.515	2.42E-02
ENSRNOG00000003689	Nono	-0.005	0.070	0.292	-0.062	-0.210	2.42E-02
ENSRNOG00000004773	Yaf2	0.121	-0.241	-0.536	0.143	0.307	2.42E-02
ENSRNOG00000012292	Alg8	-0.012	0.338	0.496	-0.371	-0.506	2.42E-02
ENSRNOG00000055642	AC107096.1	0.262	0.608	1.999	-1.119	-2.522	2.43E-02
ENSRNOG00000025252	Yars2	-0.084	-0.337	-0.590	0.185	0.412	2.43E-02
ENSRNOG00000023397	LOC102552732	-0.099	0.137	-0.158	0.368	0.709	2.43E-02
ENSRNOG00000002572	Cacybp	0.214	0.014	0.206	-0.147	-0.401	2.44E-02
ENSRNOG00000003127	Spryd4	-0.150	-0.162	-0.346	0.101	0.375	2.44E-02
ENSRNOG00000020280	Armc6	0.064	0.048	0.174	-0.089	-0.405	2.45E-02
ENSRNOG00000032472	Adgrg2	-0.535	-0.132	-0.106	0.420	0.911	2.46E-02
ENSRNOG00000058249	Pgk1	0.210	-0.261	-1.076	0.233	0.618	2.46E-02
ENSRNOG00000000860	Vwa7	-0.278	-0.191	-0.755	0.043	0.580	2.46E-02
ENSRNOG00000008546	Mrpl35	-0.119	-0.206	-0.452	0.168	0.372	2.46E-02
ENSRNOG00000002240	Dirc2	0.149	-0.186	-0.475	0.153	0.369	2.46E-02
ENSRNOG00000000464	Rxrb	-0.013	-0.071	-0.351	0.127	0.310	2.46E-02
ENSRNOG00000014080	Kif23	0.023	0.407	1.587	-0.345	-1.493	2.47E-02
ENSRNOG00000018886	Aaed1	0.155	-0.242	-0.552	0.216	0.362	2.47E-02
ENSRNOG00000009535	Stoml2	-0.057	-0.141	-0.363	0.191	0.327	2.47E-02
ENSRNOG00000028585	Tceal8	0.229	0.089	0.359	-0.233	-0.406	2.47E-02
ENSRNOG00000001134	Rfc5	0.294	0.330	0.504	-0.317	-0.450	2.47E-02

ENSRNOG00000036921	Tmsb10	0.129	0.241	1.192	-0.227	-0.890	2.48E-02
ENSRNOG00000025184	Prss35	0.784	1.612	2.876	-1.871	-2.286	2.48E-02
ENSRNOG00000049560	Glul	0.614	-0.127	-0.569	0.324	0.723	2.48E-02
ENSRNOG00000018522	Echs1	-0.020	-0.263	-0.558	0.181	0.603	2.48E-02
ENSRNOG00000016690	Idi1	-0.022	0.326	0.409	-0.415	-0.460	2.48E-02
ENSRNOG00000009325	Fuca1	0.245	0.406	0.895	-0.453	-0.552	2.48E-02
ENSRNOG00000000853	Aif1	0.149	0.367	0.870	-0.237	-0.640	2.48E-02
ENSRNOG00000001704	Runx1	-0.549	2.189	2.974	-1.653	-1.552	2.48E-02
ENSRNOG00000009364	Ndufb9	-0.159	-0.326	-0.683	0.264	0.540	2.48E-02
ENSRNOG00000010452	Cycs	-0.050	-0.485	-0.924	0.286	0.699	2.48E-02
ENSRNOG00000023919	Gbas	-0.087	-0.203	-0.642	0.137	0.424	2.48E-02
ENSRNOG00000016795	Rabl6	0.183	-0.222	-0.517	0.227	0.318	2.48E-02
ENSRNOG00000002711	Nuf2	0.082	0.723	1.369	-0.780	-1.633	2.49E-02
ENSRNOG00000019627	Mybpc2	0.351	-0.186	-1.484	0.266	0.953	2.49E-02
ENSRNOG00000005009	Zfp3	0.259	-0.303	-0.729	0.235	0.444	2.50E-02
ENSRNOG00000024221	Mettl24	-1.108	-0.543	-0.761	0.555	1.520	2.50E-02
ENSRNOG00000018932	Ccdc124	0.195	-0.161	-0.645	0.272	0.471	2.50E-02
ENSRNOG00000012830	Paqr8	-0.296	0.258	1.581	-0.146	-1.432	2.50E-02
ENSRNOG00000005301	Eif2b4	-0.076	-0.188	-0.448	0.177	0.313	2.51E-02
ENSRNOG00000057133	AABR07034455.2	-0.069	-1.238	-2.405	1.133	1.696	2.51E-02
ENSRNOG00000032856	Tssk1b	-0.945	-0.714	-0.793	0.627	1.608	2.51E-02
ENSRNOG00000022748	Tmem126a	-0.061	-0.428	-0.677	0.232	0.481	2.51E-02
ENSRNOG00000017037	Otud3	0.045	-0.124	-0.732	0.159	0.558	2.52E-02
ENSRNOG00000024629	Hadha	-0.520	-0.286	-0.256	0.227	0.542	2.52E-02
ENSRNOG00000055295	Pecr	-0.122	-0.332	-0.829	0.354	0.505	2.52E-02
ENSRNOG00000009545	Polr1a	-0.056	0.322	0.584	-0.431	-0.412	2.52E-02
ENSRNOG00000017741	Nudt5	-0.078	0.350	0.819	-0.190	-0.509	2.52E-02

ENSRNOG00000010438	Cpt1b	-0.380	-0.067	-0.210	0.028	0.422	2.52E-02
ENSRNOG00000017172	Mvb12b	0.305	-0.274	-0.855	0.297	0.499	2.53E-02
ENSRNOG00000002935	Ankrd40	0.005	-0.179	-0.513	0.141	0.317	2.53E-02
ENSRNOG00000036576	Zdhhc6	-0.022	0.034	0.332	0.007	-0.318	2.53E-02
ENSRNOG00000010326	Col20a1	0.465	0.573	0.800	-1.000	-0.982	2.53E-02
ENSRNOG00000018113	Anlnl1	0.353	0.694	1.943	-0.818	-1.747	2.53E-02
ENSRNOG00000048256	Fam160a1	0.125	-0.217	-0.986	0.040	0.602	2.54E-02
ENSRNOG00000016301	Dmrt2	-0.277	-0.618	-1.013	0.156	0.531	2.54E-02
ENSRNOG00000033057	Gapdh-ps2	0.377	-0.403	-1.113	0.187	0.766	2.54E-02
ENSRNOG00000042740	Mrpl42	0.073	-0.443	-0.807	0.317	0.490	2.55E-02
ENSRNOG00000001088	Rfc3	0.102	-0.001	0.288	-0.102	-0.461	2.56E-02
ENSRNOG00000009495	Src	0.004	0.274	0.992	0.110	-0.628	2.57E-02
ENSRNOG00000014571	Dbndd2	0.323	-0.142	-0.563	0.227	0.447	2.57E-02
ENSRNOG00000009912	Fgr	0.142	0.836	1.263	-0.631	-0.799	2.58E-02
ENSRNOG00000058472	AABR07049292.1	0.030	0.178	-0.330	0.458	0.820	2.61E-02
ENSRNOG00000003749	Xk	0.231	-0.267	-0.567	0.230	0.395	2.61E-02
ENSRNOG00000047247	Ptprs	0.061	-0.262	-0.795	0.241	0.491	2.61E-02
ENSRNOG00000052247	Manba	0.204	0.360	0.630	-0.239	-0.500	2.61E-02
ENSRNOG00000004011	Nedd1	0.043	0.042	-0.556	0.125	0.451	2.61E-02
ENSRNOG00000045749	LOC100361879	-0.047	-0.322	-0.685	0.403	0.651	2.62E-02
ENSRNOG00000010274	Smc4	0.284	-0.416	-0.073	-0.211	-0.557	2.62E-02
ENSRNOG00000026649	Dnmt3a	0.196	-0.406	-0.851	0.348	0.532	2.62E-02
ENSRNOG00000017912	Atp2a3	-0.089	-0.078	-0.274	0.068	0.440	2.62E-02
ENSRNOG00000005695	Mgp	-0.354	0.349	1.255	-0.437	-0.773	2.62E-02
ENSRNOG00000007637	Acer2	-0.190	-0.136	-0.244	0.433	0.791	2.63E-02
ENSRNOG00000002373	Akap1	-0.226	-0.274	-0.605	0.162	0.466	2.63E-02
ENSRNOG00000052840	Tanc2	-0.058	0.225	0.647	-0.465	-0.669	2.64E-02

ENSRNOG00000013783	Efhd2	-0.015	0.331	0.864	-0.164	-0.490	2.64E-02
ENSRNOG00000058324	AC098750.5	-0.161	-0.328	-0.590	0.346	0.598	2.66E-02
ENSRNOG00000012502	Stk17b	0.040	-0.139	0.518	-0.061	-0.566	2.67E-02
ENSRNOG00000008639	Pabpc1	0.011	0.265	0.787	-0.294	-0.430	2.67E-02
ENSRNOG00000007540	Msc	-0.125	0.706	1.444	-0.798	-1.142	2.68E-02
ENSRNOG00000001847	Ccdc91	0.201	-0.184	-0.771	0.148	0.482	2.68E-02
ENSRNOG00000002112	Zfp644	-0.154	-0.152	-0.295	0.186	0.329	2.68E-02
ENSRNOG00000018509	Cx3cr1	0.055	0.450	1.553	-0.580	-1.657	2.68E-02
ENSRNOG00000003837	Mif4gd	-0.045	-0.149	-0.470	0.247	0.431	2.69E-02
ENSRNOG00000005061	Dlst	-0.272	-0.193	-0.504	0.123	0.430	2.69E-02
ENSRNOG00000046832	LOC100363642	0.053	-0.286	-0.436	0.228	0.284	2.71E-02
ENSRNOG00000017737	Dgkz	-0.071	-0.047	-0.411	0.210	0.534	2.71E-02
ENSRNOG00000012685	Adck1	0.035	-0.188	-0.551	0.131	0.390	2.71E-02
ENSRNOG00000015134	Map3k12	-0.044	-0.122	-0.313	0.163	0.296	2.71E-02
ENSRNOG00000029995	Herpud2	0.126	-0.131	-0.292	0.132	0.237	2.73E-02
ENSRNOG00000005130	Ogdh	-0.163	-0.152	-0.678	0.068	0.575	2.74E-02
ENSRNOG00000017259	Tacc3	-0.039	0.304	0.836	-0.395	-0.624	2.74E-02
ENSRNOG00000010711	Dicer1	0.063	-0.175	-0.490	0.104	0.351	2.74E-02
ENSRNOG00000028047	Mecr	-0.139	-0.187	-0.576	0.190	0.506	2.74E-02
ENSRNOG00000016460	Clu	0.274	0.006	-0.067	0.341	0.456	2.74E-02
ENSRNOG00000026928	AABR07008097.1	0.176	-0.218	-1.084	0.282	0.645	2.75E-02
ENSRNOG00000018126	Abca1	0.254	0.526	1.214	-0.284	-0.768	2.75E-02
ENSRNOG00000010375	Slc39a2	0.293	1.029	2.266	-1.297	-2.040	2.76E-02
ENSRNOG00000011981	Slc39a13	0.131	0.120	0.124	-0.095	-0.291	2.76E-02
ENSRNOG00000048088	Mest	0.025	0.213	1.272	-0.325	-1.140	2.77E-02
ENSRNOG00000037658	Gprasp2	0.059	0.004	0.762	0.133	-0.639	2.77E-02
ENSRNOG00000016249	Cep85	-0.078	-0.103	-0.412	0.103	0.300	2.78E-02

ENSRNOG00000024365	Ect2	-0.271	0.384	1.633	0.221	-1.831	2.78E-02
ENSRNOG00000031335	Ankrd37	0.026	-0.716	-0.688	0.642	0.903	2.79E-02
ENSRNOG00000006364	Dld	-0.204	-0.360	-0.574	0.195	0.479	2.79E-02
ENSRNOG00000000858	Sapcd1	-0.277	-0.364	-0.622	0.291	0.460	2.79E-02
ENSRNOG00000009713	Oxa1l	-0.155	-0.154	-0.357	0.151	0.326	2.79E-02
ENSRNOG00000047321	Hba2	-0.742	-0.519	-1.032	0.567	1.024	2.79E-02
ENSRNOG00000011952	Samm50	-0.057	-0.206	-0.574	0.169	0.375	2.79E-02
ENSRNOG00000014727	Fahd1	-0.268	-0.302	-0.504	0.148	0.473	2.79E-02
ENSRNOG00000016888	Pask	0.051	0.334	1.164	-1.041	-1.466	2.79E-02
ENSRNOG00000046168	Ppm1l	0.083	-0.187	-0.849	0.155	0.522	2.80E-02
ENSRNOG00000025155	Lmtk2	0.043	-0.018	-0.487	0.045	0.427	2.80E-02
ENSRNOG00000004668	Slc25a35	0.158	-0.182	-0.374	0.243	0.404	2.80E-02
ENSRNOG00000008256	Mrpl38	-0.119	-0.156	-0.496	0.210	0.428	2.80E-02
ENSRNOG00000016214	Agl	0.108	-0.276	-1.207	0.212	0.764	2.80E-02
ENSRNOG00000015603	Prkce	-0.002	-0.047	-0.758	0.071	0.656	2.81E-02
ENSRNOG00000020541	Nprl3	0.117	0.028	-0.400	0.174	0.403	2.82E-02
ENSRNOG00000014811	Cyhr1	0.079	-0.150	-0.575	0.213	0.339	2.82E-02
ENSRNOG00000012864	Ern1	0.028	0.051	-0.395	0.063	0.345	2.84E-02
ENSRNOG00000029980	Zbtb16	-0.190	-0.383	-0.911	0.252	0.863	2.84E-02
ENSRNOG00000005568	Pcnxl4	-0.272	-0.067	-0.363	0.027	0.492	2.84E-02
ENSRNOG00000019024	Trim67	-0.618	0.595	-1.335	-0.232	2.635	2.84E-02
ENSRNOG00000047393	Krt18	-0.793	5.346	7.621	-4.023	-4.400	2.84E-02
ENSRNOG00000029886	Hba1	-0.768	-0.580	-1.049	0.581	1.026	2.86E-02
ENSRNOG00000017307	Prss23	0.000	0.227	0.350	-0.238	-0.311	2.86E-02
ENSRNOG00000011831	Nudt18	0.371	0.254	0.194	-0.125	-0.483	2.86E-02
ENSRNOG00000005762	Rab22a	0.179	-0.216	-0.466	0.162	0.245	2.88E-02
ENSRNOG00000025349	Maneal	0.267	0.103	-0.295	0.777	1.111	2.88E-02

ENSRNOG00000002187	Ropn1	-2.261	-1.422	-2.283	1.675	2.840	2.88E-02
ENSRNOG00000060703	Troap	-0.008	-0.006	1.460	-0.240	-2.135	2.88E-02
ENSRNOG00000051471	AABR07063346.1	0.566	1.309	1.457	-1.749	-1.327	2.89E-02
ENSRNOG00000015858	Hyal1	-0.411	-0.530	-1.039	0.286	0.963	2.89E-02
ENSRNOG00000009205	Lmo4	-0.037	-0.082	-0.406	0.267	0.393	2.89E-02
ENSRNOG00000001544	Cyyr1	-0.045	-0.262	-0.478	0.229	0.580	2.89E-02
ENSRNOG00000018288	Ncoa6	-0.011	-0.055	-0.295	0.117	0.289	2.90E-02
ENSRNOG00000031706	AABR07027388.1	0.242	-0.290	-1.126	0.253	0.579	2.90E-02
ENSRNOG00000007805	Mybl2	-0.101	0.932	1.773	-1.502	-1.711	2.90E-02
ENSRNOG00000006086	Lynx1	0.031	-0.422	-1.311	0.205	0.637	2.90E-02
ENSRNOG00000013742	Large	-0.513	-0.086	-0.391	-0.013	0.621	2.90E-02
ENSRNOG00000006326	Atraid	0.073	0.004	0.210	-0.049	-0.307	2.91E-02
ENSRNOG00000006763	Rbm18	-0.011	-0.078	-0.224	0.041	0.205	2.92E-02
ENSRNOG00000023850	Tbrg1	0.216	0.166	0.321	-0.258	-0.337	2.92E-02
ENSRNOG00000016617	Wwtr1	0.049	0.406	0.551	-0.523	-0.498	2.92E-02
ENSRNOG00000040300	Raet1c	0.189	0.158	-0.583	0.240	1.403	2.93E-02
ENSRNOG00000018611	Tpsb2	-0.157	-0.084	-0.004	0.493	0.645	2.93E-02
ENSRNOG00000000661	Hps4	-0.084	-0.171	-0.487	0.125	0.368	2.93E-02
ENSRNOG00000028255	Usp36	-0.026	0.018	-0.309	0.006	0.304	2.93E-02
ENSRNOG00000015109	Ubxn8	-0.132	0.312	0.673	-0.338	-0.520	2.93E-02
ENSRNOG00000016348	Tat	-1.298	-1.480	-1.600	1.496	2.247	2.93E-02
ENSRNOG00000015357	Bmper	0.349	0.087	0.219	-0.133	-0.524	2.93E-02
ENSRNOG00000032948	Tyk2	-0.066	0.269	0.458	-0.126	-0.259	2.94E-02
ENSRNOG00000047322	Crb3	0.095	-0.411	-1.128	0.389	0.665	2.94E-02
ENSRNOG00000018057	Mrpl2	-0.098	-0.152	-0.448	0.151	0.330	2.94E-02
ENSRNOG00000002496	Stxbp5l	-0.917	1.823	3.423	-1.666	-2.557	2.94E-02
ENSRNOG00000013669	Pik3r4	-0.054	0.191	0.332	-0.222	-0.307	2.95E-02

ENSRNOG00000002778	Aatf	0.029	0.101	0.417	-0.113	-0.381	2.95E-02
ENSRNOG00000050669	LOC100911515	0.096	-0.481	-1.112	0.450	0.779	2.96E-02
ENSRNOG00000031769	Chchd7	-0.127	-0.300	-0.423	0.295	0.475	2.96E-02
ENSRNOG00000016311	Slc6a2	0.540	-0.114	-1.196	0.500	1.276	2.97E-02
ENSRNOG00000017495	Klhdc3	0.287	-0.399	-0.840	0.295	0.492	2.97E-02
ENSRNOG00000055667	AC119111.1	-0.006	0.320	0.869	-0.522	-1.184	2.97E-02
ENSRNOG00000031127	Snrpe	0.168	0.054	0.366	-0.115	-0.393	2.97E-02
ENSRNOG00000033262	Reep6	-0.344	0.158	-0.417	0.162	0.759	2.97E-02
ENSRNOG00000007500	Top1mt	-0.112	-0.261	-0.667	0.244	0.413	2.97E-02
ENSRNOG00000046150	AABR07008805.1	-0.143	-0.086	-0.401	0.381	0.632	2.98E-02
ENSRNOG00000016923	Clptm1l	-0.008	0.051	0.292	-0.057	-0.232	2.98E-02
ENSRNOG00000016358	Apba2	-0.653	-0.155	-0.774	0.070	1.746	2.98E-02
ENSRNOG00000023803	Cmya5	0.267	-0.267	-1.061	0.171	0.718	2.98E-02
ENSRNOG00000025689	Abhd1	-0.151	-0.018	-0.055	0.169	0.464	2.98E-02
ENSRNOG00000058096	AABR07021704.1	-0.260	-0.173	-0.759	-0.122	0.695	3.00E-02
ENSRNOG00000057661	AABR07029863.2	-0.173	-0.380	-0.765	0.506	0.630	3.01E-02
ENSRNOG00000057701	Myom1	-0.113	-0.024	-0.564	0.081	0.452	3.01E-02
ENSRNOG00000007405	Krba1	-0.407	-0.140	-0.339	0.157	0.366	3.01E-02
ENSRNOG00000042492	Rfwd2	0.235	-0.263	-0.599	0.163	0.337	3.01E-02
ENSRNOG00000047023	Aldh1l1	-0.026	0.163	-0.314	-0.040	0.334	3.01E-02
ENSRNOG00000029205	Zfp354c	0.143	0.001	0.334	0.011	-0.378	3.01E-02
ENSRNOG00000020813	Ltbp3	0.183	0.149	0.217	-0.123	-0.385	3.01E-02
ENSRNOG00000007235	Atp5g1	-0.185	-0.369	-0.773	0.289	0.614	3.02E-02
ENSRNOG00000004457	RGD1560617	0.137	0.157	0.703	-0.239	-0.500	3.02E-02
ENSRNOG00000018570	C1qtnf3	0.355	0.831	2.055	-1.191	-1.738	3.02E-02
ENSRNOG00000012209	Cdadc1	0.175	-0.361	-0.582	0.233	0.410	3.02E-02
ENSRNOG00000018179	Glt8d1	0.103	0.168	0.465	-0.296	-0.418	3.02E-02

ENSRNOG00000047194	Arl13b	0.025	0.154	0.605	-0.274	-0.525	3.02E-02
ENSRNOG00000019445	Msln	-2.356	-0.918	-1.274	0.491	2.304	3.04E-02
ENSRNOG00000017933	Ccdc3	-0.405	-0.355	-0.754	0.712	1.573	3.04E-02
ENSRNOG00000059440	Rn60_9_0975.2	-0.250	-0.410	-0.889	0.578	0.786	3.05E-02
ENSRNOG00000022971	LOC102555919	0.114	-0.045	-0.543	0.150	0.667	3.05E-02
ENSRNOG00000015233	Etfa	-0.444	-0.302	-0.356	0.179	0.533	3.06E-02
ENSRNOG00000021248	Cdc25b	-0.356	-0.041	-0.038	0.275	0.561	3.08E-02
ENSRNOG00000018232	Srf	-0.007	-0.106	-0.466	0.142	0.374	3.08E-02
ENSRNOG00000002580	Trmt1l	-0.147	-0.131	-0.520	0.063	0.288	3.08E-02
ENSRNOG00000003715	Srpx2	0.618	0.961	1.914	-0.552	-1.128	3.08E-02
ENSRNOG00000007650	Cd63	0.185	0.360	0.727	-0.239	-0.429	3.09E-02
ENSRNOG00000005277	Ptprv	-0.312	0.508	2.588	-1.185	-2.014	3.09E-02
ENSRNOG00000004027	Aldh9a1	0.002	0.052	-0.277	0.055	0.228	3.10E-02
ENSRNOG00000014758	Lgi1	-2.254	-1.214	-1.986	1.150	2.445	3.10E-02
ENSRNOG00000010537	Dis3l	0.105	-0.159	-0.534	0.177	0.302	3.10E-02
ENSRNOG00000048736	Mllt1	-0.099	-0.009	-0.165	0.105	0.290	3.10E-02
ENSRNOG00000027220	Pcdhgb8	-0.165	0.160	-0.232	-0.049	0.633	3.11E-02
ENSRNOG00000042499	Tmsb10	0.105	0.182	0.987	-0.165	-0.701	3.11E-02
ENSRNOG00000013034	Dupd1	0.578	0.196	-0.199	-0.334	-0.732	3.11E-02
ENSRNOG00000050949	Ttc39c	-0.229	0.100	0.432	-0.126	-0.465	3.11E-02
ENSRNOG00000051682	Zcchc9	0.053	0.096	0.304	-0.102	-0.303	3.11E-02
ENSRNOG00000018053	Fech	-0.088	-0.130	-0.405	0.100	0.329	3.12E-02
ENSRNOG00000017589	Gnat1	0.283	0.952	1.795	-1.341	-1.672	3.14E-02
ENSRNOG00000004483	Ptprr	-0.119	-0.324	-0.393	0.501	0.748	3.16E-02
ENSRNOG00000021685	Cdk5r1	0.101	0.985	1.949	-1.772	-1.694	3.16E-02
ENSRNOG00000027623	LOC680039	-0.023	-0.079	-0.368	0.006	0.342	3.16E-02
ENSRNOG00000052990	Cers5	-0.070	0.188	0.697	-0.194	-0.373	3.17E-02

ENSRNOG00000019349	Sf3a2	-0.083	-0.032	-0.247	0.153	0.325	3.18E-02
ENSRNOG00000027901	Mrm1	-0.131	-0.033	-0.474	0.114	0.484	3.18E-02
ENSRNOG00000004404	Tac4	-0.176	2.825	4.996	-2.553	-3.498	3.18E-02
ENSRNOG00000015026	Arhgef11	-0.076	-0.095	-0.372	0.105	0.314	3.18E-02
ENSRNOG00000019311	Nfkb2	-0.027	0.907	1.084	-0.602	-0.597	3.18E-02
ENSRNOG00000014871	Zic4	1.780	1.854	1.747	-1.312	-2.219	3.19E-02
ENSRNOG00000047235	Vps26b	-0.080	-0.094	-0.186	0.153	0.170	3.19E-02
ENSRNOG00000008843	Eci1	-0.258	-0.061	-0.123	0.132	0.447	3.20E-02
ENSRNOG00000040122	Myoz1	0.631	-0.281	-1.121	0.368	0.744	3.21E-02
ENSRNOG00000013653	Pdlim7	0.078	-0.106	-0.858	0.194	0.534	3.21E-02
ENSRNOG00000001878	Klhl22	0.160	-0.070	-0.399	0.145	0.332	3.21E-02
ENSRNOG00000004681	Eny2	-0.098	0.139	0.658	-0.178	-0.465	3.21E-02
ENSRNOG00000002831	Wfikkn2	-0.114	0.265	0.664	-0.313	-0.759	3.21E-02
ENSRNOG00000045637	Rnf14	-0.010	-0.130	-0.270	0.074	0.259	3.21E-02
ENSRNOG00000039076	LOC688452	-0.005	0.234	0.878	-0.197	-0.607	3.22E-02
ENSRNOG00000015982	Wnt11	-0.239	-0.329	-0.531	0.254	0.568	3.23E-02
ENSRNOG00000036692	Gcgr	-1.678	-0.839	-1.461	0.811	1.486	3.24E-02
ENSRNOG00000017405	Raly	-0.141	-0.068	-0.295	0.206	0.319	3.24E-02
ENSRNOG00000052365	3082ex4-5	0.045	-0.023	-0.338	0.211	0.628	3.26E-02
ENSRNOG00000060511	Tcap	0.330	-0.098	-0.630	0.193	0.531	3.26E-02
ENSRNOG00000008246	Emilin1	-0.048	0.165	0.678	-0.064	-0.502	3.26E-02
ENSRNOG00000046261	Acp5	0.314	0.291	1.128	-0.419	-0.952	3.26E-02
ENSRNOG00000003330	Acsf2	-0.334	0.062	-0.007	0.287	0.543	3.26E-02
ENSRNOG00000043143	Eml1	0.047	-0.134	-0.860	0.195	0.503	3.28E-02
ENSRNOG00000002232	Aff1	-0.086	-0.032	-0.657	0.095	0.537	3.28E-02
ENSRNOG00000021420	Tirap	0.140	-0.114	-0.377	0.057	0.429	3.28E-02
ENSRNOG00000003835	Slc43a2	-0.035	0.518	0.775	-0.392	-0.586	3.28E-02

ENSRNOG00000013328	Rbpms	-0.230	-0.229	-0.185	0.443	0.541	3.29E-02
ENSRNOG00000036659	Fn3k	0.548	-0.263	-1.217	0.335	0.742	3.29E-02
ENSRNOG00000014065	Zfp653	-0.061	-0.089	-0.318	0.231	0.376	3.29E-02
ENSRNOG00000012204	LOC100294508	0.291	-0.102	-0.639	0.123	0.374	3.29E-02
ENSRNOG00000031827	Arih2	0.040	-0.095	-0.409	0.058	0.261	3.29E-02
ENSRNOG00000033110	Svep1	0.260	0.215	0.704	-0.445	-0.751	3.29E-02
ENSRNOG00000025383	LOC100365902	-0.166	-0.248	-0.559	0.166	0.383	3.29E-02
ENSRNOG00000002461	Nid1	-0.030	0.116	0.615	-0.287	-0.607	3.29E-02
ENSRNOG00000059579	Gpt2	0.311	-0.356	-1.531	0.357	0.934	3.30E-02
ENSRNOG00000018164	Spcs2	0.254	-0.308	-0.507	0.254	0.290	3.30E-02
ENSRNOG00000009183	Szrd1	0.059	-0.080	-0.379	0.078	0.258	3.32E-02
ENSRNOG00000060846	Morc4	-0.037	0.170	0.522	-0.251	-0.435	3.32E-02
ENSRNOG00000017609	Cnga4	0.889	1.178	1.532	-1.064	-1.674	3.32E-02
ENSRNOG00000008451	Fut8	0.107	0.177	0.388	-0.122	-0.400	3.34E-02
ENSRNOG00000000341	Nid2	0.088	0.024	0.210	-0.096	-0.559	3.34E-02
ENSRNOG00000024809	Ntmt1	-0.227	-0.254	-0.586	0.206	0.410	3.34E-02
ENSRNOG00000004333	Flywch1	-0.072	-0.045	-0.310	0.132	0.360	3.35E-02
ENSRNOG00000032788	Dysf	-0.063	0.376	0.460	-0.378	-0.374	3.35E-02
ENSRNOG00000023465	LOC500300	-0.137	-0.777	-1.094	0.673	0.907	3.35E-02
ENSRNOG00000007097	Gpatch3	-0.077	-0.219	-0.443	0.386	0.446	3.36E-02
ENSRNOG00000012952	Lrig1	0.090	-0.217	-0.911	0.206	0.514	3.37E-02
ENSRNOG00000010169	Atpaf1	-0.170	-0.361	-0.787	0.184	0.493	3.37E-02
ENSRNOG00000027480	Tmem116	-0.575	-0.223	-0.898	0.112	0.873	3.37E-02
ENSRNOG00000008279	Mrps15	0.142	-0.234	-0.610	0.238	0.379	3.37E-02
ENSRNOG00000023923	Fastkd2	-0.035	-0.288	-0.531	0.147	0.383	3.37E-02
ENSRNOG00000021669	Mybl1	-0.095	0.338	0.875	-0.248	-0.617	3.37E-02
ENSRNOG00000002734	AABR07042077.1	0.876	1.064	2.624	-1.306	-2.107	3.38E-02

ENSRNOG00000060087	Adra1b	-1.528	2.443	3.892	-2.296	-2.521	3.38E-02
ENSRNOG00000059698	Gpr141	-0.293	1.471	2.692	-1.578	-1.862	3.38E-02
ENSRNOG00000025676	Fam198a	0.245	0.534	1.254	-0.240	-0.791	3.39E-02
ENSRNOG00000052810	Cyp2c11	-1.325	-0.967	-1.736	1.704	2.340	3.40E-02
ENSRNOG00000001258	Snx8	-0.048	0.217	0.758	-0.089	-0.466	3.40E-02
ENSRNOG00000025643	Ccdc13	0.059	-0.073	0.844	0.056	-1.772	3.41E-02
ENSRNOG00000046443	Mthfsd	-0.049	-0.265	-0.645	0.202	0.483	3.42E-02
ENSRNOG00000002669	Cluh	-0.311	-0.144	-0.526	0.082	0.437	3.43E-02
ENSRNOG00000015242	Fbxl8	0.133	0.340	0.568	-0.455	-0.625	3.43E-02
ENSRNOG00000051619	Asb2	-0.099	-0.261	-1.377	0.268	0.892	3.44E-02
ENSRNOG00000017321	Trub1	-0.149	-0.136	-0.444	0.139	0.376	3.45E-02
ENSRNOG00000017416	Ppic	0.216	0.183	1.044	-0.394	-0.845	3.45E-02
ENSRNOG00000010096	Azi2	0.087	-0.110	-0.229	0.135	0.209	3.45E-02
ENSRNOG00000029172	LOC100361944	-0.027	-0.303	-0.721	0.336	0.507	3.45E-02
ENSRNOG00000028711	Dgat1	-0.023	-0.094	-0.304	0.033	0.291	3.45E-02
ENSRNOG00000056559	AABR07034669.1	-0.014	0.269	0.614	-0.196	-0.529	3.45E-02
ENSRNOG00000020835	Hipk4	0.068	0.228	-0.429	0.013	0.686	3.45E-02
ENSRNOG00000007869	Wscd1	-0.168	-0.569	-0.608	0.190	0.643	3.45E-02
ENSRNOG00000002848	Maoa	-0.014	-0.137	-0.396	0.036	0.298	3.45E-02
ENSRNOG00000003946	Tor1aip1	0.190	-0.013	-0.315	0.061	0.293	3.45E-02
ENSRNOG00000008673	Arpc3	-0.088	0.181	0.517	-0.080	-0.320	3.45E-02
ENSRNOG00000020903	Oraov1	0.447	0.347	0.241	-0.662	-0.436	3.45E-02
ENSRNOG00000004273	Ifitm1	-0.160	0.435	1.678	-0.357	-0.877	3.45E-02
ENSRNOG00000009348	Nos3	-0.192	-0.223	-0.380	0.312	0.527	3.46E-02
ENSRNOG00000003163	Sdhc	-0.188	-0.267	-0.638	0.204	0.464	3.46E-02
ENSRNOG00000040273	AABR07003017.1	0.196	0.080	0.529	-0.184	-0.451	3.47E-02
ENSRNOG00000052296	Shank3	-0.242	-0.095	-0.414	0.261	0.579	3.48E-02

ENSRNOG00000047219	Ube3b	0.051	-0.127	-0.552	0.078	0.355	3.49E-02
ENSRNOG00000054854	AC110387.1	0.190	0.061	0.472	-0.133	-0.565	3.49E-02
ENSRNOG00000053061	Ubl4a	-0.132	-0.207	-0.521	0.119	0.325	3.51E-02
ENSRNOG00000011253	RGD1304810	-0.491	-0.627	-1.040	-0.270	1.109	3.51E-02
ENSRNOG00000005433	Shq1	0.276	0.497	0.815	-0.691	-0.713	3.52E-02
ENSRNOG00000012347	Gata2	-0.079	-0.214	-0.276	0.299	0.463	3.53E-02
ENSRNOG00000008214	Fbxo9	0.014	-0.126	-0.407	0.061	0.291	3.54E-02
ENSRNOG00000015409	LOC100911959	-0.212	0.334	0.496	-0.352	-0.370	3.54E-02
ENSRNOG00000002273	Naaa	-0.025	0.280	0.810	-0.164	-0.557	3.54E-02
ENSRNOG00000020339	Neurl1	0.284	-0.133	-0.733	0.259	0.508	3.54E-02
ENSRNOG00000018650	Ap3m2	0.110	0.399	0.348	-0.480	-0.452	3.54E-02
ENSRNOG00000057991	7SK	-1.005	-0.697	-3.474	1.104	3.002	3.56E-02
ENSRNOG00000004823	Dock4	0.000	-0.320	-0.879	0.044	0.578	3.56E-02
ENSRNOG00000010091	Efcab14	0.149	-0.149	-0.443	0.040	0.286	3.56E-02
ENSRNOG00000024066	Fundc2	-0.066	-0.135	-0.846	0.332	0.540	3.56E-02
ENSRNOG00000029535	Nrbp2	-0.182	0.371	0.713	-0.438	-0.488	3.56E-02
ENSRNOG00000061379	C7	0.219	0.240	1.125	-0.438	-1.129	3.57E-02
ENSRNOG00000013867	Fgf1	-1.284	-0.446	-0.578	0.344	1.189	3.57E-02
ENSRNOG00000023381	Proca1	-0.437	-0.226	-0.463	0.203	0.570	3.57E-02
ENSRNOG00000047928	AABR07048653.1	-0.268	0.332	1.489	-0.494	-1.229	3.57E-02
ENSRNOG00000042962	Pdss2	-0.261	-0.225	-0.476	0.131	0.392	3.58E-02
ENSRNOG00000039388	Prrt4	-0.771	3.372	4.152	-2.517	-2.575	3.58E-02
ENSRNOG00000016827	Slc38a3	-0.330	-0.284	-0.633	0.298	0.506	3.58E-02
ENSRNOG00000058987	Rn60_20_0049.10	0.111	0.030	1.134	0.013	-1.292	3.58E-02
ENSRNOG00000049918	Lrg1	-0.103	-0.094	-0.286	0.190	0.644	3.59E-02
ENSRNOG00000004234	Mgat2	0.085	0.102	0.550	-0.070	-0.352	3.60E-02
ENSRNOG00000015902	Cpxm2	-0.283	-0.351	-0.612	0.272	1.276	3.61E-02

ENSRNOG00000007136	Anxa7	0.158	0.279	0.621	-0.215	-0.427	3.61E-02
ENSRNOG00000010655	Mttp	0.011	0.247	0.590	0.006	-0.812	3.61E-02
ENSRNOG00000061857	Mgst2	-0.167	1.217	2.202	-1.052	-1.327	3.61E-02
ENSRNOG00000037871	Sfxn5	0.113	-0.391	-1.563	0.310	0.879	3.62E-02
ENSRNOG00000051615	Hmgn2	-0.324	0.286	0.753	-0.190	-0.513	3.62E-02
ENSRNOG00000017523	H6pd	0.112	0.447	0.629	-0.447	-0.473	3.62E-02
ENSRNOG00000010910	Gmeb1	0.039	-0.022	-0.130	0.065	0.232	3.62E-02
ENSRNOG00000033615	Mt-nd3	-0.254	-0.396	-0.609	0.296	0.581	3.64E-02
ENSRNOG00000037190	Cd101	-0.030	1.049	1.876	-0.628	-1.044	3.66E-02
ENSRNOG00000019058	Gstm3	0.037	1.959	2.272	-1.693	-1.755	3.66E-02
ENSRNOG00000061046	AABR07059632.3	-0.009	-0.158	-0.306	0.199	0.314	3.66E-02
ENSRNOG00000000456	Psmb8	0.170	0.686	1.152	-0.303	-0.641	3.66E-02
ENSRNOG00000016360	Gps2	0.344	-0.141	-0.458	0.201	0.384	3.66E-02
ENSRNOG00000012081	Txn1	0.015	0.251	0.644	-0.276	-0.438	3.67E-02
ENSRNOG00000001880	Dgcr6	0.166	-0.251	-0.834	0.325	0.553	3.67E-02
ENSRNOG00000011020	Eif3l	0.006	0.125	0.351	-0.100	-0.249	3.67E-02
ENSRNOG00000025075	Relt	-0.005	0.625	0.761	-0.712	-0.753	3.67E-02
ENSRNOG00000027489	Mn1	0.449	-0.333	-1.205	0.181	0.924	3.68E-02
ENSRNOG00000024376	Zfp111	0.076	-0.163	-0.400	0.258	0.299	3.69E-02
ENSRNOG00000052725	RGD1566029	-0.147	0.240	0.758	-0.641	-1.084	3.70E-02
ENSRNOG00000051605	SNORD86	-0.393	0.256	-0.389	0.211	1.402	3.70E-02
ENSRNOG00000046918	Apoo	0.076	-0.341	-0.772	0.211	0.467	3.70E-02
ENSRNOG00000002194	Coq2	-0.006	-0.150	-0.493	0.176	0.383	3.70E-02
ENSRNOG00000021660	Nprl2	0.002	-0.150	-0.269	0.226	0.261	3.70E-02
ENSRNOG00000020893	Snx27	-0.032	-0.075	-0.349	0.075	0.248	3.70E-02
ENSRNOG00000006766	Laptm4b	-0.008	0.105	0.394	-0.233	-0.266	3.70E-02
ENSRNOG00000014369	Slc27a4	0.006	0.267	0.363	-0.302	-0.349	3.70E-02

ENSRNOG00000010370	Tnip1	0.004	0.296	0.400	-0.153	-0.358	3.70E-02
ENSRNOG00000016587	Ninj1	-0.002	0.501	1.123	-0.336	-0.592	3.70E-02
ENSRNOG00000025895	Sdpr	-0.190	-0.420	-0.403	0.407	0.521	3.71E-02
ENSRNOG00000021123	AC098622.1	-0.173	-0.331	-0.772	0.346	0.625	3.72E-02
ENSRNOG00000027894	Iqgap3	0.342	0.785	1.231	-0.626	-0.996	3.72E-02
ENSRNOG00000001161	Gatc	0.090	-0.205	-0.476	0.158	0.329	3.72E-02
ENSRNOG00000019842	Artn	0.267	2.482	3.505	-2.471	-1.887	3.72E-02
ENSRNOG00000046629	Zfp37	0.581	0.337	0.875	-0.541	-1.404	3.73E-02
ENSRNOG00000012378	Dbnl	-0.084	0.274	0.403	-0.231	-0.275	3.74E-02
ENSRNOG00000026914	Dnah1	-0.559	0.737	1.587	-0.263	-1.376	3.74E-02
ENSRNOG00000011949	Ndufb5	-0.217	-0.345	-0.630	0.269	0.525	3.75E-02
ENSRNOG00000052734	AABR07051310.1	-0.490	0.263	-0.902	0.259	1.922	3.76E-02
ENSRNOG00000016362	Gpr4	-0.136	-0.100	-0.471	0.346	0.699	3.76E-02
ENSRNOG00000008523	Faf1	0.080	-0.250	-0.490	0.203	0.382	3.76E-02
ENSRNOG00000013517	Phtf2	-0.259	-0.331	-0.428	0.156	0.424	3.76E-02
ENSRNOG00000020251	Art1	0.024	-0.090	-0.484	0.110	0.383	3.76E-02
ENSRNOG00000026793	Nt5dc3	-0.044	-0.114	-0.684	-0.009	0.608	3.76E-02
ENSRNOG00000042320	Slc41a1	-0.300	-0.132	-0.287	0.271	0.382	3.76E-02
ENSRNOG00000022274	Slc2a8	0.215	-0.093	-0.528	0.197	0.349	3.77E-02
ENSRNOG00000018148	Cd99l2	0.179	0.581	0.998	-0.499	-0.991	3.78E-02
ENSRNOG00000002338	Dgke	-0.052	-0.281	-0.553	0.205	0.503	3.78E-02
ENSRNOG00000020905	Hdac5	-0.132	-0.048	-0.301	0.176	0.364	3.79E-02
ENSRNOG00000028064	Fhad1	-0.214	1.543	3.873	-2.562	-2.925	3.80E-02
ENSRNOG00000027456	Cdc42bpg	-0.122	-0.077	-0.217	0.237	0.518	3.80E-02
ENSRNOG00000016631	Zfp276	-0.016	0.122	-0.210	0.211	0.499	3.80E-02
ENSRNOG00000019127	Zfp606	-0.136	-0.320	-0.597	0.308	0.445	3.80E-02
ENSRNOG00000023152	Tmem201	0.120	-0.092	-0.702	0.139	0.425	3.80E-02

ENSRNOG00000043141	Ap3s2	0.087	0.130	0.170	-0.213	-0.286	3.80E-02
ENSRNOG00000050547	Syngr2	0.192	-0.083	-0.575	0.144	0.325	3.80E-02
ENSRNOG00000021839	Cep126	0.182	0.167	0.943	-0.602	-1.113	3.80E-02
ENSRNOG00000016813	Tia1	0.018	0.180	0.252	-0.119	-0.283	3.81E-02
ENSRNOG00000017707	Abi2	0.084	0.023	0.183	-0.233	-0.411	3.81E-02
ENSRNOG00000015773	Il21r	0.083	0.707	1.737	-0.243	-1.044	3.81E-02
ENSRNOG00000002693	Nme1	0.016	0.140	0.448	-0.139	-0.478	3.81E-02
ENSRNOG00000054609	AABR07002683.1	0.094	0.117	0.368	-0.243	-0.380	3.81E-02
ENSRNOG00000000302	Sesn1	0.104	-0.220	-0.608	0.219	0.371	3.82E-02
ENSRNOG00000046799	Phb	-0.094	-0.181	-0.454	0.243	0.351	3.82E-02
ENSRNOG00000005486	AABR07058267.1	0.127	-0.337	-0.756	0.128	0.519	3.84E-02
ENSRNOG00000001169	Slc37a1	0.064	-0.018	-0.431	0.145	0.439	3.85E-02
ENSRNOG00000020908	LOC303566	0.368	0.985	1.115	-1.362	-1.368	3.85E-02
ENSRNOG00000030124	Ptpn11	0.146	-0.114	-0.518	0.036	0.282	3.85E-02
ENSRNOG00000007818	Slc45a4	-0.066	0.367	0.199	-0.450	-0.367	3.85E-02
ENSRNOG00000012415	Mpc1	-0.189	-0.423	-0.726	0.225	0.565	3.85E-02
ENSRNOG00000008061	Nuak1	-0.250	0.105	-0.082	0.130	0.410	3.86E-02
ENSRNOG00000014052	Ccna1	-0.021	-0.360	-0.670	0.296	0.465	3.86E-02
ENSRNOG00000005447	LOC100364791	-0.202	0.488	0.725	-0.331	-0.807	3.88E-02
ENSRNOG00000051541	AC133316.1	-0.742	-0.288	-2.164	0.486	2.082	3.88E-02
ENSRNOG00000027491	Vldlr	0.199	-0.324	-0.763	0.157	0.399	3.88E-02
ENSRNOG00000007172	Map4k3	-0.031	-0.014	-0.246	0.123	0.300	3.88E-02
ENSRNOG00000032844	RT1-Da	0.311	0.664	0.976	-0.646	-0.821	3.88E-02
ENSRNOG00000052880	Prph	-0.129	-0.454	-0.612	0.650	0.838	3.88E-02
ENSRNOG00000042848	Jam2	-0.058	-0.242	-0.246	0.188	0.397	3.88E-02
ENSRNOG00000013875	Fbxl17	-0.072	-0.054	-0.458	0.061	0.287	3.88E-02
ENSRNOG00000019073	Ikbkb	-0.368	0.000	-0.165	0.022	0.274	3.88E-02

ENSRNOG00000009450	Hcn4	-1.965	-0.978	-1.400	1.131	1.867	3.89E-02
ENSRNOG00000009323	Fam214b	-0.282	0.580	1.063	-0.572	-0.677	3.89E-02
ENSRNOG00000017102	Cmtr2	0.037	-0.150	-0.435	0.012	0.416	3.91E-02
ENSRNOG00000030127	Eml2	-0.175	0.372	0.455	-0.316	-0.334	3.91E-02
ENSRNOG00000002917	Sec14l5	0.323	-0.678	-2.008	0.374	1.340	3.92E-02
ENSRNOG00000020451	Cd81	0.124	0.093	0.228	-0.114	-0.265	3.93E-02
ENSRNOG00000004312	Tmbim4	0.134	0.081	0.315	-0.062	-0.317	3.94E-02
ENSRNOG00000007799	Fam151a	-0.366	-0.415	-0.900	0.580	0.957	3.94E-02
ENSRNOG00000048222	Nlrc5	-0.559	1.103	1.905	-0.637	-0.822	3.94E-02
ENSRNOG00000010812	Osbpl6	-0.001	-0.258	-0.904	0.217	0.568	3.95E-02
ENSRNOG00000027115	Zc2hc1c	-0.209	-0.312	-0.731	0.240	0.780	3.96E-02
ENSRNOG00000026880	Usp38	0.055	-0.170	-0.572	0.111	0.391	3.97E-02
ENSRNOG00000008159	Msantd3	0.189	-0.004	0.155	-0.151	-0.294	3.97E-02
ENSRNOG00000020590	Adam15	-0.051	0.065	-0.016	0.082	0.425	3.97E-02
ENSRNOG00000021085	Sf1	-0.068	-0.013	-0.174	0.117	0.256	3.98E-02
ENSRNOG00000016033	Endog	-0.222	-0.248	-0.868	0.251	0.657	3.98E-02
ENSRNOG00000029152	Tmem69	-0.084	-0.252	-0.371	0.103	0.337	3.98E-02
ENSRNOG00000046028	Haus6	0.036	0.411	0.909	-0.522	-0.631	3.98E-02
ENSRNOG00000014946	Thumpd1	-0.141	-0.144	-0.183	0.083	0.247	3.98E-02
ENSRNOG00000021004	Rasip1	-0.164	-0.125	-0.139	0.234	0.436	3.98E-02
ENSRNOG00000017090	RGD1308706	0.331	-0.248	-0.672	0.201	0.382	3.99E-02
ENSRNOG00000010467	Cpa5	0.925	0.352	0.771	-1.652	-2.128	3.99E-02
ENSRNOG00000052533	AABR07052521.1	0.187	-0.413	-1.002	0.473	1.493	3.99E-02
ENSRNOG00000010204	Tmem9	-0.609	0.432	1.470	-0.256	-0.919	4.00E-02
ENSRNOG00000010819	Hspa4l	-0.269	0.143	-0.118	0.601	0.862	4.02E-02
ENSRNOG00000007129	Cd8b	-0.027	0.818	2.160	-0.258	-1.912	4.04E-02
ENSRNOG00000012723	Trim55	0.017	0.345	0.487	-0.240	-0.361	4.04E-02

ENSRNOG00000015121	N4bp1	0.026	-0.038	-0.275	0.065	0.354	4.05E-02
ENSRNOG00000015118	Cpped1	0.034	-0.128	-0.653	0.103	0.350	4.05E-02
ENSRNOG00000023021	Msl2	-0.022	-0.149	-0.402	0.134	0.364	4.07E-02
ENSRNOG00000047816	Ccs	-0.123	-0.103	-0.403	0.287	0.357	4.07E-02
ENSRNOG00000014287	Stk11	-0.108	-0.128	-0.312	0.191	0.296	4.07E-02
ENSRNOG00000020277	Cntnap1	0.025	0.328	0.437	-0.377	-0.647	4.07E-02
ENSRNOG00000018207	Dynlt1	-0.051	0.348	1.052	-0.383	-0.689	4.07E-02
ENSRNOG00000036876	RGD1308923	0.022	0.521	0.960	-0.436	-0.697	4.07E-02
ENSRNOG00000003949	Phka2	0.458	0.417	1.114	-0.693	-1.518	4.07E-02
ENSRNOG00000012664	Polr1e	0.104	0.349	0.476	-0.405	-0.464	4.08E-02
ENSRNOG00000050318	Fsd1	-0.360	1.008	1.795	-1.315	-1.097	4.08E-02
ENSRNOG00000013647	Polm	0.544	-0.176	-0.861	0.233	0.535	4.09E-02
ENSRNOG00000057837	AABR07017236.1	0.458	-0.532	-0.841	0.260	0.887	4.09E-02
ENSRNOG00000061786	Rn60_18_0774.1	0.293	-0.303	-0.935	0.438	0.700	4.09E-02
ENSRNOG00000028208	Mief2	-0.301	-0.205	-0.575	0.141	0.416	4.10E-02
ENSRNOG00000032917	Zfand2a	-0.025	0.935	1.537	-0.806	-0.910	4.10E-02
ENSRNOG00000024930	Smim19	0.192	-0.353	-0.514	0.307	0.321	4.11E-02
ENSRNOG00000042939	Nrip2	-0.146	0.068	-0.493	-0.124	0.806	4.11E-02
ENSRNOG00000014330	Pcmt1	0.026	-0.163	-0.482	0.130	0.281	4.11E-02
ENSRNOG00000019100	Kif2c	-0.261	0.025	1.399	-0.206	-1.613	4.11E-02
ENSRNOG00000020296	Usmg5	-0.080	-0.373	-0.615	0.275	0.562	4.11E-02
ENSRNOG00000019659	Aspa	-0.066	-0.114	0.207	-0.521	-0.842	4.12E-02
ENSRNOG00000000588	Slc16a10	0.142	-0.020	-0.775	0.071	0.603	4.12E-02
ENSRNOG00000033719	Pcdhb12	0.118	-0.118	0.066	-0.449	-0.646	4.12E-02
ENSRNOG00000001736	Bdh1	-1.071	-0.637	-0.912	0.567	2.305	4.13E-02
ENSRNOG00000014461	Galns	0.175	-0.011	-0.775	0.186	0.550	4.13E-02
ENSRNOG00000003086	Cenpv	0.170	-0.013	-0.406	0.050	0.372	4.13E-02

ENSRNOG00000009756	Pacsin2	-0.204	-0.042	-0.126	0.118	0.251	4.13E-02
ENSRNOG00000017213	Cdk11b	0.031	-0.054	-0.207	0.121	0.219	4.13E-02
ENSRNOG00000002773	Rgs4	-0.132	-0.324	-0.658	0.411	0.638	4.13E-02
ENSRNOG00000042503	Ndufv2	-0.099	-0.333	-0.653	0.252	0.478	4.13E-02
ENSRNOG00000017506	Cltb	0.140	-0.004	-0.478	0.155	0.387	4.13E-02
ENSRNOG00000011189	Acy1	-0.050	-0.079	-0.238	0.186	0.361	4.13E-02
ENSRNOG00000006399	Synj2bp	0.021	-0.281	-0.538	0.148	0.327	4.13E-02
ENSRNOG00000014637	Atxn10	-0.061	0.036	0.221	-0.089	-0.241	4.13E-02
ENSRNOG00000061348	Fam53b	-0.254	0.291	0.377	-0.327	-0.354	4.13E-02
ENSRNOG00000037331	Cd33	0.091	0.549	1.259	-0.314	-0.830	4.13E-02
ENSRNOG00000008431	Gabbr2	0.146	0.520	1.114	-0.665	-0.977	4.13E-02
ENSRNOG00000039110	Lsmem1	0.273	-0.359	-0.987	0.264	0.600	4.14E-02
ENSRNOG00000009549	Fbxo3	-0.059	-0.219	-0.412	0.097	0.324	4.15E-02
ENSRNOG00000050042	Myh8	0.292	0.611	0.920	-0.670	-1.096	4.16E-02
ENSRNOG00000003150	Mpc2	-0.234	-0.385	-0.627	0.334	0.568	4.16E-02
ENSRNOG00000004968	Ncapg2	0.108	0.220	0.855	-0.580	-0.761	4.16E-02
ENSRNOG00000038044	Tsc22d2	-0.001	-0.094	-0.520	0.080	0.393	4.17E-02
ENSRNOG00000005012	Utp14a	-0.050	0.040	0.349	-0.127	-0.305	4.18E-02
ENSRNOG00000059350	Ppp1r3a	0.192	-0.348	-0.920	0.171	0.641	4.19E-02
ENSRNOG00000027574	AABR07053879.1	0.335	-0.173	-1.187	0.105	1.151	4.20E-02
ENSRNOG00000033906	Zfp667	0.160	-0.375	-0.615	0.219	0.513	4.20E-02
ENSRNOG00000014964	Hp	-0.365	-0.262	-0.539	0.322	0.852	4.20E-02
ENSRNOG00000005667	Astn1	-0.169	-0.314	-0.453	-0.028	0.727	4.20E-02
ENSRNOG00000013747	Sh3bp2	-0.008	0.640	1.248	-0.462	-0.744	4.20E-02
ENSRNOG00000034038	AABR07005844.1	-0.271	-0.312	-0.470	0.167	0.936	4.20E-02
ENSRNOG00000006832	Zdhhc5	0.054	-0.115	-0.367	0.054	0.222	4.20E-02
ENSRNOG00000014132	Mypop	0.079	-0.068	-0.793	0.419	0.619	4.21E-02

ENSRNOG00000049920	Psma2	0.047	0.071	0.319	-0.153	-0.315	4.21E-02
ENSRNOG00000011882	Gab2	-0.019	0.250	0.523	-0.536	-0.477	4.21E-02
ENSRNOG00000001647	Ets2	0.624	0.645	0.719	-0.551	-0.632	4.21E-02
ENSRNOG00000021151	Ppp1r14b	-0.109	0.358	0.547	-0.230	-0.531	4.21E-02
ENSRNOG00000015701	Rreb1	-0.144	-0.066	-0.476	0.035	0.429	4.22E-02
ENSRNOG00000003786	Tspan6	0.176	-0.076	0.348	0.015	-0.333	4.22E-02
ENSRNOG00000002860	Ccdc181	0.203	-0.324	-0.812	0.305	0.536	4.23E-02
ENSRNOG00000000130	Dnajb5	-0.198	-0.116	-0.535	0.085	0.493	4.24E-02
ENSRNOG00000003504	Rnaseh2a	0.013	-0.184	-0.469	0.287	0.402	4.24E-02
ENSRNOG00000008711	Med24	-0.003	-0.122	-0.460	0.164	0.282	4.24E-02
ENSRNOG00000020039	Slc5a2	-0.456	0.654	1.600	-0.189	-1.494	4.25E-02
ENSRNOG00000019693	Clpb	-0.045	-0.140	-0.398	0.164	0.330	4.25E-02
ENSRNOG00000017311	Me3	-0.130	-0.203	-0.706	0.195	0.512	4.26E-02
ENSRNOG00000005686	Suclg2	-0.275	-0.112	-0.279	0.094	0.280	4.26E-02
ENSRNOG00000043167	Itga9	-0.034	0.150	1.316	-0.311	-0.870	4.26E-02
ENSRNOG00000017074	Rab28	0.163	-0.226	-0.555	0.204	0.338	4.26E-02
ENSRNOG00000056964	LOC102549158	0.020	0.227	0.481	-0.281	-0.320	4.26E-02
ENSRNOG00000003669	Myocd	-0.786	-0.622	-1.068	0.261	0.918	4.26E-02
ENSRNOG00000019473	Dcun1d2	-0.075	-0.060	-0.872	0.295	0.867	4.26E-02
ENSRNOG00000011060	Unc119	-0.283	-0.249	-0.606	0.344	0.484	4.26E-02
ENSRNOG00000009031	Gucy2c	-0.403	-0.720	-1.585	0.970	0.920	4.26E-02
ENSRNOG00000009419	Ptprg	-0.094	-0.084	-0.492	0.041	0.552	4.27E-02
ENSRNOG00000011026	Irf2bpl	-0.163	-0.141	-0.555	0.121	0.469	4.27E-02
ENSRNOG00000048989	Larp7	-0.102	0.048	0.199	-0.158	-0.323	4.27E-02
ENSRNOG00000007246	Atxn7	-0.016	-0.022	-0.516	-0.052	0.454	4.29E-02
ENSRNOG00000007628	Ptp4a3	0.183	-0.153	-0.434	0.322	0.437	4.29E-02
ENSRNOG00000009280	Mkrn1	-0.094	0.100	0.232	-0.192	-0.310	4.30E-02

ENSRNOG00000021149	Ush1c	0.138	-0.110	-1.388	0.205	1.151	4.30E-02
ENSRNOG00000058593	Zbtb1	-0.030	0.145	0.344	-0.222	-0.400	4.33E-02
ENSRNOG00000014215	Klf9	0.082	-0.184	-0.725	0.069	0.575	4.33E-02
ENSRNOG00000052539	Prpf40b	-0.106	-0.061	-0.357	0.108	0.291	4.33E-02
ENSRNOG00000010975	Adnp	-0.096	-0.108	-0.262	0.078	0.272	4.34E-02
ENSRNOG00000017693	Slc2a5	-0.282	0.197	-0.070	0.366	0.940	4.34E-02
ENSRNOG00000001603	N6amt1	-0.070	-0.251	-0.576	0.256	0.363	4.34E-02
ENSRNOG00000061108	Arcn1	0.073	-0.113	-0.164	0.100	0.191	4.34E-02
ENSRNOG00000018865	Adamts12	0.147	-0.002	0.478	-0.271	-0.642	4.34E-02
ENSRNOG00000057659	AABR07067080.1	0.090	-0.143	-1.218	0.160	0.932	4.37E-02
ENSRNOG00000028330	AABR07067600.1	0.114	-0.161	-0.355	0.020	0.348	4.37E-02
ENSRNOG00000001711	Hrasls	0.070	-0.497	-0.765	0.310	0.320	4.37E-02
ENSRNOG00000000457	Tap1	-0.068	0.575	1.055	-0.281	-0.511	4.37E-02
ENSRNOG00000002343	Uchl1	-0.509	1.369	2.775	-1.532	-1.714	4.37E-02
ENSRNOG00000011847	Grk4	-0.015	-0.337	-0.424	0.539	0.398	4.37E-02
ENSRNOG00000009686	Aqp7	-1.910	-1.044	-0.421	1.360	1.722	4.38E-02
ENSRNOG00000034078	Mxi1	0.266	-0.160	-0.426	0.262	0.338	4.38E-02
ENSRNOG00000050067	LOC100364509	0.023	0.015	0.379	0.057	-0.341	4.38E-02
ENSRNOG00000013583	Tbc1d8	0.052	0.378	0.453	-0.207	-0.386	4.38E-02
ENSRNOG00000053850	Rdh5	-0.382	0.608	1.704	-0.449	-1.410	4.38E-02
ENSRNOG00000001628	Pcp4	-0.203	1.419	3.210	-1.481	-2.476	4.38E-02
ENSRNOG00000015813	Ubr2	-0.012	-0.187	-0.565	0.132	0.409	4.38E-02
ENSRNOG00000000576	Anapc16	0.264	-0.104	-0.765	0.129	0.461	4.39E-02
ENSRNOG00000020460	Banf1	0.164	-0.340	-0.641	0.287	0.384	4.39E-02
ENSRNOG00000049511	Safb2	-0.079	-0.126	-0.335	0.154	0.286	4.39E-02
ENSRNOG00000007971	Wbp2	0.032	-0.032	-0.294	0.132	0.242	4.41E-02
ENSRNOG00000047783	Tmem200a	-0.382	0.673	2.311	-1.576	-2.152	4.41E-02

ENSRNOG00000013358	Aqp11	-0.002	0.525	1.126	-0.654	-1.319	4.42E-02
ENSRNOG00000001706	Kalrn	0.067	-0.385	-0.830	0.379	0.562	4.42E-02
ENSRNOG00000007578	Zfp830	-0.094	-0.219	-0.366	0.164	0.316	4.42E-02
ENSRNOG00000028752	Tmem81	0.027	-0.317	-0.444	-0.170	0.719	4.42E-02
ENSRNOG00000042533	Accsl	-0.832	-0.803	-0.737	0.720	1.311	4.43E-02
ENSRNOG00000018159	Anxa4	0.206	0.245	0.471	-0.172	-0.334	4.43E-02
ENSRNOG00000003717	Cnih4	0.125	-0.066	0.200	-0.049	-0.290	4.43E-02
ENSRNOG00000000306	Smpd2	0.112	0.138	0.319	-0.260	-0.351	4.43E-02
ENSRNOG00000011936	Abhd14a	-0.062	-0.209	-0.460	0.289	0.406	4.43E-02
ENSRNOG00000007827	Cox4i2	-0.133	-0.284	-0.515	0.171	0.617	4.43E-02
ENSRNOG00000031269	RGD1304694	0.042	0.083	-0.100	-0.007	0.229	4.44E-02
ENSRNOG00000012473	Cflar	0.004	-0.015	-0.274	0.061	0.335	4.44E-02
ENSRNOG00000049537	Rn60_13_0828.1	0.190	0.120	0.428	-0.145	-0.340	4.44E-02
ENSRNOG00000010208	Timp1	0.193	0.629	1.549	-0.958	-1.013	4.46E-02
ENSRNOG00000013461	Ralbp1	0.152	-0.129	-0.354	0.159	0.224	4.46E-02
ENSRNOG00000000571	Psap	0.126	-0.015	-0.404	0.051	0.301	4.46E-02
ENSRNOG00000018809	Psmd5	-0.057	0.312	0.635	-0.335	-0.391	4.47E-02
ENSRNOG00000011863	Gins3	0.065	0.210	1.032	-0.328	-1.097	4.47E-02
ENSRNOG00000036672	Sectm1a	0.279	1.762	2.922	-1.359	-1.649	4.50E-02
ENSRNOG00000016544	Arhgef28	-0.028	0.024	-0.270	0.084	0.498	4.50E-02
ENSRNOG00000008934	Tmem65	-0.301	-0.339	-0.664	0.257	0.657	4.51E-02
ENSRNOG00000013683	S1pr1	0.028	-0.341	-0.594	0.266	0.527	4.51E-02
ENSRNOG00000007351	Ggh	-0.019	-0.288	0.195	0.082	-0.579	4.51E-02
ENSRNOG00000052298	AABR07014974.1	-0.199	-0.223	-0.712	0.184	0.771	4.51E-02
ENSRNOG00000010213	Fgd5	-0.180	-0.177	-0.335	0.271	0.441	4.51E-02
ENSRNOG00000002055	Noa1	-0.021	-0.159	-0.388	0.131	0.329	4.51E-02
ENSRNOG00000014209	Utp6	0.099	0.087	0.173	-0.168	-0.266	4.51E-02

ENSRNOG00000008424	Aagab	0.110	0.280	0.238	-0.389	-0.312	4.51E-02
ENSRNOG00000017003	Plxna1	-0.055	0.243	0.494	-0.206	-0.409	4.51E-02
ENSRNOG00000016561	Ns5atp9	0.366	0.261	1.627	-1.279	-1.919	4.51E-02
ENSRNOG00000014117	Hmox1	0.043	0.912	1.545	-0.998	-0.889	4.51E-02
ENSRNOG00000001047	Map2k7	-0.001	-0.053	-0.407	0.083	0.286	4.52E-02
ENSRNOG00000003600	Pnpt1	-0.119	-0.260	-0.302	0.133	0.316	4.53E-02
ENSRNOG00000015188	LOC100909998	0.009	-0.228	-0.661	0.376	0.649	4.53E-02
ENSRNOG00000019511	Mrps18a	-0.040	-0.151	-0.417	0.196	0.346	4.54E-02
ENSRNOG00000061358	AC129365.1	0.053	-0.149	0.452	0.149	-0.501	4.55E-02
ENSRNOG00000018760	Mpp7	-0.469	-1.100	-1.153	0.762	1.251	4.55E-02
ENSRNOG00000022060	Magi1	-0.518	-0.012	-0.363	0.126	0.439	4.55E-02
ENSRNOG00000001514	Cdca7	0.268	0.259	0.995	-0.243	-1.145	4.55E-02
ENSRNOG00000017869	Irf8	-0.399	0.771	1.627	-0.237	-0.772	4.55E-02
ENSRNOG00000017502	Sike1	0.163	-0.083	0.085	-0.112	-0.279	4.56E-02
ENSRNOG00000002710	Cnst	0.223	-0.071	-1.088	0.185	0.634	4.57E-02
ENSRNOG00000018349	Polr1b	0.065	-0.143	-0.378	0.069	0.364	4.57E-02
ENSRNOG00000007710	Usp20	-0.318	0.342	0.692	-0.309	-0.445	4.58E-02
ENSRNOG00000019996	Slc16a1	-0.410	-0.445	-0.668	0.320	0.762	4.58E-02
ENSRNOG00000011140	Fam213a	-0.416	-0.097	-0.399	0.231	0.843	4.58E-02
ENSRNOG00000021954	Spg11	-0.036	-0.070	-0.406	0.163	0.338	4.59E-02
ENSRNOG00000056246	Gls	-0.010	-0.197	-0.354	0.097	0.288	4.59E-02
ENSRNOG00000009029	Nptn	0.054	-0.118	-0.382	0.052	0.220	4.59E-02
ENSRNOG00000042960	Rgcc	-0.279	0.220	1.440	-0.314	-1.114	4.59E-02
ENSRNOG00000003846	Pitpna	0.098	0.065	0.117	-0.153	-0.238	4.59E-02
ENSRNOG00000024503	Nbas	0.000	-0.046	-0.402	-0.013	0.302	4.59E-02
ENSRNOG00000016494	Tmem204	-0.110	-0.177	-0.327	0.259	0.471	4.60E-02
ENSRNOG00000015972	Ano5	0.160	-0.299	-1.092	0.093	0.696	4.60E-02

ENSRNOG00000014104	Myo5b	-0.378	1.759	4.398	-1.431	-2.350	4.60E-02
ENSRNOG00000013586	Snai3	-0.370	-1.177	-0.909	0.887	1.061	4.62E-02
ENSRNOG00000015735	Zswim1	0.061	-0.196	-0.548	0.228	0.438	4.63E-02
ENSRNOG00000004459	Sdr9c7	-0.978	2.582	4.413	-2.514	-3.117	4.64E-02
ENSRNOG00000012386	Zbtb38	0.274	0.018	-0.701	-0.086	0.477	4.65E-02
ENSRNOG00000054391	Snurf	-0.001	-0.321	-1.638	0.375	1.494	4.65E-02
ENSRNOG00000021143	Setdb1	-0.027	0.136	0.193	-0.098	-0.223	4.65E-02
ENSRNOG00000012134	Scn4a	0.203	-0.081	-0.818	0.034	0.541	4.66E-02
ENSRNOG00000037638	Timm50	-0.118	-0.103	-0.334	0.124	0.275	4.66E-02
ENSRNOG00000003229	Tspan7	0.201	-0.003	0.182	-0.120	-0.289	4.66E-02
ENSRNOG00000003931	Arsg	-0.015	0.270	0.374	-0.278	-0.331	4.66E-02
ENSRNOG00000047984	Mrps22	-0.069	-0.188	-0.412	0.155	0.301	4.67E-02
ENSRNOG00000046479	Chaf1a	0.024	0.146	0.363	-0.274	-0.461	4.67E-02
ENSRNOG00000056941	AABR07066529.3	-0.725	-0.385	-0.444	0.140	0.874	4.67E-02
ENSRNOG00000027049	Atp5j2	0.000	-0.322	-0.567	0.237	0.435	4.67E-02
ENSRNOG00000033663	P4ha2	0.187	-0.285	-0.495	0.317	0.396	4.67E-02
ENSRNOG00000019840	Mdp1	-0.055	-0.326	-0.518	0.265	0.396	4.67E-02
ENSRNOG00000013598	Melk	0.132	0.625	2.352	-1.220	-1.602	4.68E-02
ENSRNOG00000010361	Kif3b	-0.027	0.206	0.269	-0.296	-0.308	4.69E-02
ENSRNOG00000003280	Grin2c	-0.058	1.481	2.663	-2.045	-1.931	4.69E-02
ENSRNOG00000023668	Scyl1	0.090	-0.087	-0.382	0.148	0.263	4.69E-02
ENSRNOG00000027799	Tmie	0.083	-0.188	-0.494	0.072	0.610	4.70E-02
ENSRNOG00000048193	Hnrnph3	-0.143	-0.007	-0.283	0.059	0.436	4.70E-02
ENSRNOG00000007442	Ubl7	0.076	-0.051	-0.352	0.133	0.259	4.70E-02
ENSRNOG00000011134	Lama2	-0.049	0.037	0.333	-0.353	-0.535	4.70E-02
ENSRNOG00000002423	Fam114a2	0.064	-0.125	-0.303	0.068	0.200	4.70E-02
ENSRNOG00000027503	Rps25	0.199	0.031	0.421	-0.053	-0.445	4.70E-02

ENSRNOG00000017392	Fgf2	0.270	-0.153	-0.915	-0.059	0.804	4.70E-02
ENSRNOG00000008062	Dqx1	-0.211	-0.289	-0.392	0.196	0.559	4.70E-02
ENSRNOG00000020848	Adck4	-0.155	0.352	0.584	-0.266	-0.403	4.70E-02
ENSRNOG00000017206	Igfbp5	-0.149	0.626	0.955	-0.415	-0.632	4.70E-02
ENSRNOG00000049105	AC095947.2	0.147	0.148	0.509	-0.061	-0.449	4.70E-02
ENSRNOG00000010239	Cnbp	-0.073	-0.201	-0.387	0.113	0.274	4.71E-02
ENSRNOG00000014190	Hint3	0.057	-0.288	-0.584	0.242	0.366	4.72E-02
ENSRNOG00000008807	Rp1	-2.068	-1.454	-1.419	1.725	2.664	4.72E-02
ENSRNOG00000019811	LOC100362432	0.129	-0.284	-0.669	0.248	0.389	4.72E-02
ENSRNOG00000017133	LOC306766	0.014	-0.136	-0.460	0.075	0.310	4.72E-02
ENSRNOG00000016321	Entpd4	0.092	0.065	0.075	-0.159	-0.270	4.72E-02
ENSRNOG00000002353	Rab3gap2	-0.075	0.259	0.385	-0.290	-0.300	4.72E-02
ENSRNOG00000023269	AABR07021759.1	0.194	-0.010	0.182	-0.082	-0.327	4.72E-02
ENSRNOG00000010950	Cep41	0.205	0.085	0.414	-0.370	-0.634	4.72E-02
ENSRNOG00000015278	Myl12a	-0.541	0.401	1.239	-0.462	-0.781	4.72E-02
ENSRNOG00000045888	AABR07030798.1	0.751	0.780	1.207	-0.470	-1.339	4.72E-02
ENSRNOG00000016957	Igfbp2	0.412	1.735	2.177	-1.036	-2.111	4.72E-02
ENSRNOG00000020384	Fam13b	-0.037	0.207	0.364	-0.279	-0.318	4.72E-02
ENSRNOG00000020689	Cpeb3	-0.022	-0.018	-0.681	-0.048	0.493	4.73E-02
ENSRNOG00000011853	Mbd2	0.176	-0.219	-0.477	0.184	0.304	4.73E-02
ENSRNOG00000043503	Ehd1	-0.013	-0.105	-0.377	0.121	0.275	4.73E-02
ENSRNOG00000042576	Tcp11l1	-0.027	0.995	1.364	-0.939	-0.776	4.73E-02
ENSRNOG00000032339	Rasl12	-0.272	-0.244	-0.428	0.418	0.495	4.73E-02
ENSRNOG00000024197	AABR07049149.1	0.190	0.093	0.358	-0.172	-0.563	4.74E-02
ENSRNOG00000054049	Prelid2	-0.503	0.372	1.245	-0.141	-0.692	4.74E-02
ENSRNOG00000039985	Ube2a	-0.043	-0.152	-0.364	0.160	0.258	4.75E-02
ENSRNOG00000017466	Kif5b	0.066	0.268	0.274	-0.341	-0.367	4.75E-02

ENSRNOG00000005043	Cpeb2	0.120	-0.193	-0.903	0.056	0.587	4.76E-02
ENSRNOG00000012264	Alkbh1	-0.077	-0.095	-0.212	0.016	0.263	4.80E-02
ENSRNOG00000054360	Tspan11	0.102	0.229	0.502	-0.362	-0.560	4.80E-02
ENSRNOG00000012808	Tmem259	-0.019	-0.057	-0.309	0.129	0.286	4.80E-02
ENSRNOG00000010064	Abcc4	0.189	-0.143	-0.828	0.061	0.559	4.82E-02
ENSRNOG00000002839	Slc19a2	-0.417	0.098	0.165	-0.001	0.511	4.83E-02
ENSRNOG00000021812	Scx	0.506	0.179	0.439	-0.194	-0.662	4.83E-02
ENSRNOG00000016443	Vps13d	0.061	-0.146	-0.700	0.042	0.492	4.83E-02
ENSRNOG00000017962	Serpinb6	0.010	0.158	0.488	-0.163	-0.357	4.85E-02
ENSRNOG00000002695	Tfb2m	-0.042	-0.310	-0.413	0.239	0.395	4.86E-02
ENSRNOG00000028415	Cdc20	0.205	0.266	0.649	-0.367	-0.760	4.86E-02
ENSRNOG00000060988	Kcnc4	0.605	0.292	0.003	-0.499	-0.634	4.86E-02
ENSRNOG00000003076	Rbms2	-0.053	-0.010	-0.071	0.046	0.286	4.87E-02
ENSRNOG00000009974	Coq3	-0.214	-0.437	-0.597	0.245	0.495	4.87E-02
ENSRNOG00000019760	Oxnad1	-0.318	-0.308	-0.435	0.243	0.445	4.90E-02
ENSRNOG00000010609	Abcf2	0.131	-0.056	-0.379	0.079	0.274	4.90E-02
ENSRNOG00000002205	Ociad1	-0.051	-0.158	-0.357	0.104	0.247	4.90E-02
ENSRNOG00000058588	Nfxl1	0.066	0.127	0.328	-0.089	-0.313	4.90E-02
ENSRNOG00000019785	Phtf1	-0.023	0.109	0.340	-0.317	-0.325	4.90E-02
ENSRNOG00000023881	Skap1	0.032	0.257	1.337	-0.051	-1.318	4.90E-02
ENSRNOG00000049385	Adamtsl4	-0.240	-0.114	-0.230	0.273	0.604	4.91E-02
ENSRNOG00000058710	AABR07021465.2	-0.119	0.483	1.384	-0.618	-0.748	4.91E-02
ENSRNOG00000011684	Auh	0.113	-0.178	-0.662	0.217	0.403	4.91E-02
ENSRNOG00000003603	Arhgap44	-0.019	0.002	-0.477	-0.101	0.351	4.92E-02
ENSRNOG00000018599	Gga2	0.106	0.135	0.433	-0.262	-0.322	4.92E-02
ENSRNOG00000021068	Pip5k1a	0.095	-0.096	-0.434	0.078	0.276	4.93E-02
ENSRNOG00000015718	RGD1307461	0.189	-0.309	-1.072	0.486	0.739	4.94E-02

ENSRNOG00000023227	AABR07052585.2	-0.012	-0.031	-0.549	0.154	0.578	4.95E-02
ENSRNOG00000050315	Dcxr	0.223	-0.221	-0.647	0.254	0.523	4.95E-02
ENSRNOG00000017420	Nudt6	-0.143	-0.174	-0.504	0.173	0.382	4.96E-02
ENSRNOG00000005707	Bfsp1	-0.033	0.415	1.128	-0.209	-0.897	4.97E-02
ENSRNOG00000017446	Ndufs8	-0.100	-0.325	-0.709	0.350	0.486	4.97E-02
ENSRNOG00000032150	Adcy2	0.367	0.399	0.462	-0.213	-0.380	4.97E-02
ENSRNOG00000009462	Ccdc90b	-0.108	0.083	0.471	-0.146	-0.415	4.97E-02
ENSRNOG00000001516	Rapgef4	-0.130	-0.195	-0.502	0.215	0.467	4.99E-02
ENSRNOG00000022309	Frem1	-0.171	0.262	0.841	-0.403	-0.945	4.99E-02
ENSRNOG00000042682	Grpel2	0.008	-0.009	0.096	-0.016	-0.232	4.99E-02
