# Supplementary material for: A robust neuromuscular system protects rat and human skeletal muscle from sarcopenia
Source: Aging (Albany NY). 2016 Mar 24;8(4):712–28. doi: 10.18632/aging.100926 (PMC4925824; doi:10.18632/aging.100926)
Supplement: Supplementary file 4 [file aging-08-712-s004.pdf]

BIOLOGICAL PROCESSES						CELLULAR COMPARTMENT					
Gene Ontology ID	Gene Ontology term (Biological Process)	Number of annotated genes	Number of significant genes	Number of expected genes	p-value (Fisher test)	Gene Ontology ID	Gene Ontology term (Cellular Compartment)	Number of annotated genes	Number of significant genes	Number of expected genes	p-value (Fisher test)
GO:0006091	generation of precursor metabolites and ...	202	42	16.52	1.20E-08	GO:0044429	mitochondrial part	575	84	46.68	5.60E-08
GO:0055114	oxidation-reduction process	636	92	52.02	2.80E-08	GO:0005740	mitochondrial envelope	462	69	37.51	4.00E-07
GO:0009062	fatty acid catabolic process	64	20	5.23	8.80E-08	GO:0031966	mitochondrial membrane	436	66	35.4	4.40E-07
GO:0006006	glucose metabolic process	136	30	11.12	3.90E-07	GO:0031012	extracellular matrix	251	44	20.38	7.70E-07
GO:0007584	response to nutrient	190	37	15.54	5.00E-07	GO:0005739	mitochondrion	1339	156	108.7	8.40E-07
GO:0016054	organic acid catabolic process	132	29	10.8	6.60E-07	GO:0019866	organelle inner membrane	339	54	27.52	1.00E-06
GO:0046395	carboxylic acid catabolic process	132	29	10.8	6.60E-07	GO:0005743	mitochondrial inner membrane	310	50	25.17	1.80E-06
GO:0072329	monocarboxylic acid catabolic process	72	20	5.89	7.50E-07	GO:1902495	transmembrane transporter complex	138	26	11.2	4.00E-05
GO:0044699	single-organism process	7957	704	650.81	8.10E-07	GO:1990351	transporter complex	141	26	11.45	5.80E-05
GO:0005996	monosaccharide metabolic process	173	34	14.15	1.20E-06	GO:0070469	respiratory chain	74	17	6.01	6.80E-05
GO:0019318	hexose metabolic process	161	32	13.17	1.90E-06	GO:0034702	ion channel complex	120	23	9.74	8.30E-05
GO:0015980	energy derivation by oxidation of organi...	146	30	11.94	1.90E-06	GO:0031967	organelle envelope	699	85	56.75	8.30E-05
GO:0032787	monocarboxylic acid metabolic process	331	52	27.07	3.10E-06	GO:0031975	envelope	702	85	56.99	9.60E-05
GO:0042493	response to drug	395	59	32.31	3.60E-06	GO:0005578	proteinaceous extracellular matrix	172	29	13.96	1.20E-04
GO:0019752	carboxylic acid metabolic process	599	80	48.99	6.10E-06	GO:0005759	mitochondrial matrix	171	28	13.88	2.60E-04
GO:0044242	cellular lipid catabolic process	110	24	9	6.80E-06	GO:0000777	condensed chromosome kinetochore	43	11	3.49	4.80E-04
GO:0044723	single-organism carbohydrate metabolic p...	422	61	34.52	7.10E-06	GO:0005886	plasma membrane	2235	218	181.44	1.00E-03
GO:0009991	response to extracellular stimulus	371	55	30.34	9.60E-06	GO:0071944	cell periphery	2310	224	187.53	1.14E-03
GO:0019395	fatty acid oxidation	72	18	5.89	1.30E-05	GO:0000779	condensed chromosome, centromeric region	48	11	3.9	1.31E-03

GO:0005975 carbohydrate metabolic process 478 66 39.1 1.40E-05
GO:0005746 mitochondrial respiratory chain 64 13 5.2
1.63E-03
GO:0044282 small molecule catabolic process 170 31 13.9 1.70E-05
GO:0005614 interstitial matrix 12 5 0.97 1.70E-03
GO:0031667 response to nutrient levels 351 52 28.71 1.70E-05
GO:0005576 extracellular region 2592 246 210.42 2.07E-03
GO:0006635 fatty acid beta-oxidation 54 15 4.42 1.80E-05
GO:0044459 plasma membrane part 1103 115 89.54 2.37E-03
GO:0034440 lipid oxidation 74 18 6.05 2.00E-05 GO:0005615
extracellular space 667 74 54.15 3.20E-03
GO:0006090 pyruvate metabolic process 62 16 5.07 2.60E-05
GO:0072686 mitotic spindle 32 8 2.6 3.31E-03
GO:0043436 oxoacid metabolic process 633 81 51.77 2.60E-05
GO:0005874 microtubule 220 30 17.86 3.35E-03
GO:0044262 cellular carbohydrate metabolic process 200 34 16.36
3.10E-05 GO:0030173 integral component of Golgi membrane
27 7 2.19 4.76E-03
GO:0006082 organic acid metabolic process 640 81 52.35 3.90E-05
GO:0042383 sarcolemma 115 18 9.34 5.08E-03
GO:0034220 ion transmembrane transport 494 66 40.4 4.00E-05
GO:0044304 main axon 42 9 3.41 5.70E-03
GO:0007155 cell adhesion 627 79 51.28 5.70E-05 GO:0031594
neuromuscular junction 42 9 3.41 5.70E-03
GO:0009719 response to endogenous stimulus 1045 119 85.47 8.10E-05
GO:0000776 kinetochore 82 14 6.66 5.89E-03
GO:0022610 biological adhesion 634 79 51.86 8.30E-05
GO:0001917 photoreceptor inner segment 16 5 1.3 7.14E-03
GO:0006631 fatty acid metabolic process 227 36 18.57 8.40E-05
GO:0031228 intrinsic component of Golgi membrane 29 7 2.35
7.25E-03
GO:0022904 respiratory electron transport chain 36 11 2.94
9.20E-05 GO:0009897 external side of plasma membrane 175
24 14.21 7.61E-03
GO:0045333 cellular respiration 83 18 6.79 1.00E-04
GO:0044420 extracellular matrix part 85 14 6.9 8.12E-03
GO:1901698 response to nitrogen compound 649 80 53.08 1.10E-04
GO:0034703 cation channel complex 77 13 6.25 8.58E-03
GO:0098660 inorganic ion transmembrane transport 348 49 28.46
1.10E-04 GO:0070069 cytochrome complex 17 5 1.38
9.45E-03
GO:0006811 ion transport 846 99 69.2 1.20E-04 GO:0098552
side of membrane 227 29 18.43 9.64E-03
GO:0070482 response to oxygen levels 295 43 24.13 1.30E-04
GO:0097458 neuron part 781 81 63.4 1.18E-02
GO:0090276 regulation of peptide hormone secretion 100 20 8.18
1.40E-04 GO:0043025 neuronal cell body 332 39
26.95 1.20E-02
GO:0010243 response to organonitrogen compound 607 75 49.65
1.70E-04 GO:1990204 oxidoreductase complex 81 13 6.58
1.30E-02
GO:0006085 acetyl-CoA biosynthetic process 12 6 0.98 1.80E-04
GO:0000775 chromosome, centromeric region 109 16 8.85
1.45E-02

GO:0044707 single-multicellular organism process 3667 348 299.93
1.90E-04 GO:0009986 cell surface 484 53 39.29
1.48E-02
GO:0002791 regulation of peptide secretion 102 20 8.34 1.90E-04
GO:0042579 microbody 103 15 8.36 1.88E-02
GO:0042221 response to chemical 2119 214 173.32 2.30E-04
GO:0005777 peroxisome 103 15 8.36 1.88E-02
GO:0006096 glycolytic process 46 12 3.76 2.40E-04
GO:0044425 membrane part 3151 283 255.8 1.93E-02
GO:0090087 regulation of peptide transport 104 20 8.51 2.50E-04
GO:0042611 MHC protein complex 35 7 2.84 2.05E-02
GO:0031589 cell-substrate adhesion 204 32 16.69 2.50E-04
GO:0033267 axon part 123 17 9.99 2.09E-02
GO:0016042 lipid catabolic process 161 27 13.17 2.50E-04
GO:0044455 mitochondrial membrane part 152 20 12.34 2.13E-02
GO:0098655 cation transmembrane transport 370 50 30.26 2.60E-04
GO:0035371 microtubule plus-end 14 4 1.14 2.23E-02
GO:0044763 single-organism cellular process 7096 624 580.39
2.80E-04 GO:0008076 voltage-gated potassium channel complex
28 6 2.27 2.26E-02
GO:0007017 microtubule-based process 390 52 31.9 2.80E-04
GO:0005753 mitochondrial proton-transporting ATP sy... 21 5
1.7 2.37E-02
GO:0003008 system process 866 99 70.83 3.00E-04 GO:0016528
sarcoplasm 53 9 4.3 2.55E-02
GO:0014070 response to organic cyclic compound 691 82 56.52
3.10E-04 GO:0044421 extracellular region part 2431 221
197.35 2.59E-02
GO:0002790 peptide secretion130 23 10.63 3.20E-04 GO:0034705
potassium channel complex 29 6 2.35 2.66E-02
GO:0022900 electron transport chain 41 11 3.35 3.30E-04
GO:0016529 sarcoplasmic reticulum 45 8 3.65 2.67E-02
GO:0032501 multicellular organismal process 3788 356 309.83
3.40E-04 GO:0030424 axon 288 33 23.38 2.72E-02
GO:0046883 regulation of hormone secretion 131 23 10.71 3.60E-04
GO:0031253 cell projection membrane 156 20 12.66
2.73E-02
GO:0010675 regulation of cellular carbohydrate meta... 107 20 8.75
3.70E-04 GO:0033178 proton-transporting two-sector ATPase
co... 15 4 1.22 2.86E-02
GO:0055085 transmembrane transport 686 81 56.11 4.00E-04
GO:0000793 condensed chromosome 109 15 8.85 2.98E-02
GO:1901700 response to oxygen-containing compound 1033 114 84.49
4.10E-04 GO:0030672 synaptic vesicle membrane 31 6
2.52 3.59E-02
GO:0010033 response to organic substance 1657 171 135.53
4.20E-04 GO:0043235 receptor complex 171 21 13.88
3.66E-02
GO:0030072 peptide hormone secretion 126 22 10.31 5.20E-04
GO:0005604 basement membrane66 10 5.36 3.91E-02
GO:0042593 glucose homeostasis 143 24 11.7 5.40E-04
GO:0009925 basal plasma membrane 24 5 1.95 4.05E-02

GO:0033500 carbohydrate homeostasis 143 24 11.7 5.40E-04
GO:0045259 proton-transporting ATP synthase complex 24 5
1.95 4.05E-02
GO:0001666 response to hypoxia 259 37 21.18 5.70E-04
GO:0005875 microtubule associated complex 85 12 6.9
4.11E-02
GO:0060537 muscle tissue development 298 41 24.37 6.40E-04
GO:0045178 basal part of cell 32 6 2.6 4.12E-02
GO:0043129 surfactant homeostasis 10 5 0.82 6.40E-04
GO:0033268 node of Ranvier 10 3 0.81 4.15E-02
GO:0006812 cation transport 635 75 51.94 6.50E-04 GO:0005583
fibrillar collagen trimer 10 3 0.81 4.15E-02
GO:0007267 cell-cell signaling 546 66 44.66 7.40E-04
GO:0015630 microtubule cytoskeleton 706 70 57.31 4.39E-02
GO:0014823 response to activity 66 14 5.4 7.50E-04
GO:0045211 postsynaptic membrane 105 14 8.52 4.41E-02
GO:0006109 regulation of carbohydrate metabolic pro... 113 20 9.24
7.70E-04 GO:0036477 somatodendritic compartment 485 50
39.37 4.58E-02
GO:0071616 acyl-CoA biosynthetic process 15 6 1.23 7.70E-04
GO:0042612 MHC class I protein complex 25 5 2.03
4.74E-02
GO:0035384 thioester biosynthetic process 15 6 1.23 7.70E-04
GO:0001750 photoreceptor outer segment 25 5 2.03
4.74E-02
GO:0006094 gluconeogenesis 45 11 3.68 7.80E-04 GO:0005819
spindle 187 22 15.18 4.94E-02
GO:0046364 monosaccharide biosynthetic process 52 12 4.25
8.00E-04
GO:0036293 response to decreased oxygen levels 264 37 21.59
8.20E-04
GO:0007626 locomotory behavior 122 21 9.98 8.30E-04

GO:0015672 monovalent inorganic cation transport 283 39 23.15
8.40E-04
GO:0048878 chemical homeostasis 600 71 49.07 8.60E-04

GO:0042886 amide transport 148 24 12.11 8.90E-04

GO:0023061 signal release 219 32 17.91 8.90E-04

GO:0030336 negative regulation of cell migration 132 22 10.8
9.90E-04
GO:0072358 cardiovascular system development 676 78 55.29 1.01E-03

GO:0072359 circulatory system development 676 78 55.29 1.01E-03

GO:0048875 chemical homeostasis within a tissue 11 5 0.9
1.10E-03
GO:0009214 cyclic nucleotide catabolic process 16 6 1.31
1.15E-03
GO:0009888 tissue development 1217 128 99.54 1.24E-03

GO:0006979	response to oxidative stress	308	41	25.19	1.24E-03
GO:0098662	inorganic cation transmembrane transport	308	41	25.19	1.24E-03
GO:0015833	peptide transport	143	23	11.7	1.26E-03
GO:0007162	negative regulation of cell adhesion	85	16	6.95	1.28E-03
GO:0043648	dicarboxylic acid metabolic process	62	13	5.07	1.28E-03
GO:2000146	negative regulation of cell motility	135	22	11.04	1.34E-03
GO:0044724	single-organism carbohydrate catabolic p...	86	16	7.03	1.45E-03
GO:0050796	regulation of insulin secretion	86	16	7.03	1.45E-03
GO:0048285	organelle fission	301	40	24.62	1.46E-03
GO:0009725	response to hormone	664	76	54.31	1.47E-03
GO:0009060	aerobic respiration	35	9	2.86	1.59E-03
GO:0009409	response to cold	35	9	2.86	1.59E-03
GO:0031960	response to corticosteroid	181	27	14.8	1.61E-03
GO:0071407	cellular response to organic cyclic comp...	274	37	22.41	1.63E-03
GO:0051271	negative regulation of cellular componen...	137	22	11.21	1.63E-03
GO:0019319	hexose biosynthetic process	49	11	4.01	1.66E-03
GO:0035094	response to nicotine	42	10	3.44	1.67E-03
GO:0007274	neuromuscular synaptic transmission	12	5	0.98	1.76E-03
GO:0042044	fluid transport	12	5	0.98	1.76E-03
GO:0048513	organ development	2083	204	170.37	1.76E-03
GO:0016052	carbohydrate catabolic process	88	16	7.2	1.87E-03
GO:0046879	hormone secretion	165	25	13.5	1.89E-03
GO:0051602	response to electrical stimulus	50	11	4.09	1.98E-03
GO:0060359	response to ammonium ion	50	11	4.09	1.98E-03
GO:0048731	system development	2727	259	223.04	2.11E-03
GO:0010677	negative regulation of cellular carbohyd...	18	6	1.47	2.32E-03
GO:0030155	regulation of cell adhesion	251	34	20.53	2.35E-03

GO:0007275	multicellular organismal development	2978	280	243.57	2.35E-03
GO:0009914	hormone transport	168	25	13.74	2.43E-03
GO:0009605	response to external stimulus	1230	127	100.6	2.57E-03
GO:0014706	striated muscle tissue development	282	37	23.07	2.71E-03
GO:0051301	cell division	421	51	34.43	2.77E-03
GO:0048856	anatomical structure development	3187	297	260.67	2.80E-03
GO:0051384	response to glucocorticoid	170	25	13.9	2.86E-03
GO:0009611	response to wounding	493	58	40.32	2.87E-03
GO:0040013	negative regulation of locomotion	161	24	13.17	2.87E-03
GO:0061061	muscle structure development	412	50	33.7	2.93E-03
GO:0006119	oxidative phosphorylation	38	9	3.11	2.93E-03
GO:0030198	extracellular matrix organization	135	21	11.04	3.06E-03
GO:0045912	negative regulation of carbohydrate meta...	25	7	2.04	3.10E-03
GO:0031099	regeneration	153	23	12.51	3.12E-03
GO:0006911	phagocytosis, engulfment	19	6	1.55	3.15E-03
GO:0032502	developmental process	3478	321	284.47	3.18E-03
GO:0000280	nuclear division	275	36	22.49	3.19E-03
GO:0008037	cell recognition	53	11	4.33	3.23E-03
GO:0051046	regulation of secretion	364	45	29.77	3.27E-03
GO:0043062	extracellular structure organization	136	21	11.12	3.35E-03
GO:0071822	protein complex subunit organization	997	105	81.55	3.35E-03
GO:0035383	thioester metabolic process	46	10	3.76	3.44E-03
GO:0006637	acyl-CoA metabolic process	46	10	3.76	3.44E-03
GO:0006818	hydrogen transport	102	17	8.34	3.57E-03
GO:0050877	neurological system process	510	59	41.71	3.86E-03
GO:0006309	apoptotic DNA fragmentation	14	5	1.15	3.87E-03
GO:0006198	cAMP catabolic process	14	5	1.15	3.87E-03

GO:0097503	sialylation	14	5	1.15	3.87E-03
GO:0043434	response to peptide hormone	288	37	23.56	3.87E-03
GO:0006112	energy reserve metabolic process	62	12	5.07	3.93E-03
GO:0098661	inorganic anion transmembrane transport	47	10	3.84	4.05E-03
GO:1901652	response to peptide	309	39	25.27	4.19E-03
GO:0071456	cellular response to hypoxia	87	15	7.12	4.33E-03
GO:0044767	single-organism developmental process	3460	318	283	4.43E-03
GO:0007059	chromosome segregation	122	19	9.98	4.65E-03
GO:0034284	response to monosaccharide	158	23	12.92	4.69E-03
GO:0010324	membrane invagination	27	7	2.21	4.96E-03
GO:1903047	mitotic cell cycle process	413	49	33.78	5.01E-03
GO:0006639	acylglycerol metabolic process	64	12	5.23	5.14E-03
GO:0006754	ATP biosynthetic process	34	8	2.78	5.17E-03
GO:0036294	cellular response to decreased oxygen le...	89	15	7.28	5.39E-03
GO:0001525	angiogenesis	294	37	24.05	5.45E-03
GO:0001503	ossification	294	37	24.05	5.45E-03
GO:0031069	hair follicle morphogenesis	21	6	1.72	5.48E-03
GO:0055017	cardiac muscle tissue growth	49	10	4.01	5.54E-03
GO:1902600	hydrogen ion transmembrane transport	81	14	6.63	5.62E-03
GO:0000226	microtubule cytoskeleton organization	275	35	22.49	5.67E-03
GO:0071453	cellular response to oxygen levels	98	16	8.02	5.68E-03
GO:0001754	eye photoreceptor cell differentiation	28	7	2.29	6.15E-03
GO:0007271	synaptic transmission, cholinergic	10	4	0.82	6.25E-03
GO:0009187	cyclic nucleotide metabolic process	99	16	8.1	6.27E-03
GO:0065008	regulation of biological quality	1978	190	161.78	6.29E-03
GO:1901342	regulation of vasculature development	162	23	13.25	6.38E-03
GO:0033993	response to lipid	660	72	53.98	6.46E-03

GO:0010906	regulation of glucose metabolic process	74	13	6.05	6.50E-03
GO:0048678	response to axon injury	58	11	4.74	6.63E-03
GO:0006073	cellular glucan metabolic process	58	11	4.74	6.63E-03
GO:0000086	G2/M transition of mitotic cell cycle	58	11	4.74	6.63E-03
GO:0005977	glycogen metabolic process	58	11	4.74	6.63E-03
GO:0044042	glucan metabolic process	58	11	4.74	6.63E-03
GO:0006638	neutral lipid metabolic process	66	12	5.4	6.63E-03
GO:0006084	acetyl-CoA metabolic process	22	6	1.8	7.02E-03
GO:0009063	cellular amino acid catabolic process	59	11	4.83	7.56E-03
GO:0015992	proton transport	101	16	8.26	7.62E-03
GO:0043279	response to alkaloid	110	17	9	7.77E-03
GO:0030258	lipid modification	137	20	11.21	7.79E-03
GO:0007517	muscle organ development	261	33	21.35	7.82E-03
GO:0016051	carbohydrate biosynthetic process	128	19	10.47	7.84E-03
GO:0044281	small molecule metabolic process	1874	180	153.28	7.98E-03
GO:0071482	cellular response to light stimulus	44	9	3.6	8.21E-03
GO:0048589	developmental growth	282	35	23.07	8.35E-03
GO:0009628	response to abiotic stimulus	808	85	66.09	8.35E-03
GO:0009152	purine ribonucleotide biosynthetic proce...	138	20	11.29	8.43E-03
GO:0007067	mitotic nuclear division	204	27	16.69	8.53E-03
GO:0051781	positive regulation of cell division	52	10	4.25	8.55E-03
GO:0044839	cell cycle G2/M phase transition	60	11	4.91	8.60E-03
GO:0006641	triglyceride metabolic process	60	11	4.91	8.60E-03
GO:0055088	lipid homeostasis	60	11	4.91	8.60E-03
GO:0071322	cellular response to carbohydrate stimul...	85	14	6.95	8.64E-03
GO:0051259	protein oligomerization	333	40	27.24	8.68E-03
GO:0006921	cellular component disassembly involved ...	23	6	1.88	8.86E-03

GO:0006099	tricarboxylic acid cycle	23	6	1.88	8.86E-03
GO:0030262	apoptotic nuclear changes	23	6	1.88	8.86E-03
GO:0071331	cellular response to hexose stimulus	77	13	6.3	9.10E-03
GO:0046676	negative regulation of insulin secretion	30	7	2.45	9.17E-03
GO:0045076	regulation of interleukin-2 biosynthetic...	11	4	0.9	9.19E-03
GO:0033194	response to hydroperoxide	11	4	0.9	9.19E-03
GO:0044851	hair cycle phase	11	4	0.9	9.19E-03
GO:0006107	oxaloacetate metabolic process	11	4	0.9	9.19E-03
GO:0070208	protein heterotrimerization	11	4	0.9	9.19E-03
GO:0014059	regulation of dopamine secretion	11	4	0.9	9.19E-03
GO:0014046	dopamine secretion	11	4	0.9	9.19E-03
GO:0030900	forebrain development	274	34	22.41	9.27E-03
GO:0044765	single-organism transport	2124	201	173.72	9.32E-03
GO:0060538	skeletal muscle organ development	158	22	12.92	9.42E-03
GO:0009743	response to carbohydrate	177	24	14.48	9.52E-03
GO:0045765	regulation of angiogenesis	149	21	12.19	9.65E-03
GO:0051926	negative regulation of calcium ion trans...	17	5	1.39	9.75E-03
GO:0060419	heart growth	53	10	4.33	9.78E-03
GO:0009168	purine ribonucleoside monophosphate bios...	53	10	4.33	9.78E-03
GO:0009127	purine nucleoside monophosphate biosynth...	53	10	4.33	9.78E-03
GO:0030073	insulin secretion	104	16	8.51	1.01E-02
GO:0051047	positive regulation of secretion	197	26	16.11	1.01E-02
GO:0033002	muscle cell proliferation	122	18	9.98	1.01E-02
GO:0097485	neuron projection guidance	113	17	9.24	1.01E-02
GO:0007411	axon guidance	113	17	9.24	1.01E-02
GO:0006813	potassium ion transport	113	17	9.24	1.01E-02
GO:0071326	cellular response to monosaccharide stim...	78	13	6.38	1.01E-02

GO:0032526	response to retinoic acid	87	14	7.12	1.06E-02
GO:0048545	response to steroid hormone	389	45	31.82	1.08E-02
GO:0002931	response to ischemia	24	6	1.96	1.10E-02
GO:0048730	epidermis morphogenesis	24	6	1.96	1.10E-02
GO:0043589	skin morphogenesis	31	7	2.54	1.10E-02
GO:0061326	renal tubule development	31	7	2.54	1.10E-02
GO:0035914	skeletal muscle cell differentiation	54	10	4.42	1.12E-02
GO:0000302	response to reactive oxygen species	170	23	13.9	1.13E-02
GO:0048514	blood vessel morphogenesis	359	42	29.36	1.13E-02
GO:0021700	developmental maturation	161	22	13.17	1.16E-02
GO:0001764	neuron migration	88	14	7.2	1.17E-02
GO:0030154	cell differentiation	2229	209	182.31	1.18E-02
GO:0010632	regulation of epithelial cell migration	106	16	8.67	1.20E-02
GO:0072522	purine-containing compound biosynthetic ...	152	21	12.43	1.20E-02
GO:0007519	skeletal muscle tissue development	152	21	12.43	1.20E-02
GO:0044712	single-organism catabolic process	1232	122	100.77	1.21E-02
GO:0001678	cellular glucose homeostasis	80	13	6.54	1.25E-02
GO:1900543	negative regulation of purine nucleotide...	18	5	1.47	1.26E-02
GO:0015985	energy coupled proton transport, down el...	18	5	1.47	1.26E-02
GO:0015986	ATP synthesis coupled proton transport	18	5	1.47	1.26E-02
GO:0046903	secretion	572	62	46.78	1.26E-02
GO:0043271	negative regulation of ion transport	55	10	4.5	1.27E-02
GO:0009065	glutamine family amino acid catabolic pr...	12	4	0.98	1.29E-02
GO:0098656	anion transmembrane transport	98	15	8.02	1.30E-02
GO:0003009	skeletal muscle contraction	32	7	2.62	1.31E-02
GO:0090278	negative regulation of peptide hormone s...	32	7	2.62	1.31E-02
GO:0060612	adipose tissue development	25	6	2.04	1.35E-02

GO:0042773	ATP synthesis coupled electron transport	25	6	2.04	1.35E-02
GO:0045785	positive regulation of cell adhesion	135	19	11.04	1.36E-02
GO:0050678	regulation of epithelial cell proliferat...	202	26	16.52	1.37E-02
GO:0030534	adult behavior	81	13	6.63	1.38E-02
GO:0007268	synaptic transmission	343	40	28.05	1.39E-02
GO:0042391	regulation of membrane potential	272	33	22.25	1.41E-02
GO:0042542	response to hydrogen peroxide	117	17	9.57	1.41E-02
GO:0009206	purine ribonucleoside triphosphate biosy...	40	8	3.27	1.42E-02
GO:0010810	regulation of cell-substrate adhesion	108	16	8.83	1.42E-02
GO:0006164	purine nucleotide biosynthetic process	145	20	11.86	1.42E-02
GO:0006629	lipid metabolic process	728	76	59.54	1.46E-02
GO:0032940	secretion by cell	491	54	40.16	1.47E-02
GO:0051179	localization	3282	297	268.44	1.54E-02
GO:0002456	T cell mediated immunity	65	11	5.32	1.55E-02
GO:0002792	negative regulation of peptide secretion	33	7	2.7	1.55E-02
GO:0001676	long-chain fatty acid metabolic process	33	7	2.7	1.55E-02
GO:0060038	cardiac muscle cell proliferation	33	7	2.7	1.55E-02
GO:0071310	cellular response to organic substance	1096	109	89.64	1.55E-02
GO:2000378	negative regulation of reactive oxygen s...	19	5	1.55	1.60E-02
GO:2001222	regulation of neuron migration	19	5	1.55	1.60E-02
GO:0045980	negative regulation of nucleotide metabo...	19	5	1.55	1.60E-02
GO:0009746	response to hexose	147	20	12.02	1.64E-02
GO:0009064	glutamine family amino acid metabolic pr...	41	8	3.35	1.64E-02
GO:0014855	striated muscle cell proliferation	41	8	3.35	1.64E-02
GO:0036296	response to increased oxygen levels	41	8	3.35	1.64E-02
GO:0009145	purine nucleoside triphosphate biosynthe...	41	8	3.35	1.64E-02
GO:0055093	response to hyperoxia	41	8	3.35	1.64E-02

GO:0050896	response to stimulus	4636	409	379.18	1.67E-02
GO:0008016	regulation of heart contraction	101	15	8.26	1.69E-02
GO:0001667	ameboidal cell migration	206	26	16.85	1.73E-02
GO:0001649	osteoblast differentiation	167	22	13.66	1.73E-02
GO:0007600	sensory perception	226	28	18.48	1.75E-02
GO:0042094	interleukin-2 biosynthetic process	13	4	1.06	1.75E-02
GO:0014912	negative regulation of smooth muscle cel...	13	4	1.06	1.75E-02
GO:0046689	response to mercury ion	13	4	1.06	1.75E-02
GO:0048820	hair follicle maturation	13	4	1.06	1.75E-02
GO:0015718	monocarboxylic acid transport	75	12	6.13	1.80E-02
GO:0071333	cellular response to glucose stimulus	75	12	6.13	1.80E-02
GO:0031100	organ regeneration	75	12	6.13	1.80E-02
GO:0022402	cell cycle process	637	67	52.1	1.82E-02
GO:0042130	negative regulation of T cell proliferat...	34	7	2.78	1.82E-02
GO:0071478	cellular response to radiation	84	13	6.87	1.83E-02
GO:0046888	negative regulation of hormone secretion	50	9	4.09	1.87E-02
GO:0009260	ribonucleotide biosynthetic process	149	20	12.19	1.88E-02
GO:0071495	cellular response to endogenous stimulus	638	67	52.18	1.88E-02
GO:0014910	regulation of smooth muscle cell migrati...	42	8	3.44	1.89E-02
GO:0042060	wound healing	268	32	21.92	1.91E-02
GO:0001101	response to acid chemical	258	31	21.1	1.92E-02
GO:0021988	olfactory lobe development	27	6	2.21	1.96E-02
GO:0072080	nephron tubule development	27	6	2.21	1.96E-02
GO:0008045	motor neuron axon guidance	20	5	1.64	1.99E-02
GO:0071804	cellular potassium ion transport	85	13	6.95	2.01E-02
GO:0071805	potassium ion transmembrane transport	85	13	6.95	2.01E-02
GO:0033273	response to vitamin	94	14	7.69	2.01E-02

GO:0045766	positive regulation of angiogenesis	94	14	7.69	2.01E-02
GO:0030001	metal ion transport	457	50	37.38	2.02E-02
GO:0009749	response to glucose	141	19	11.53	2.08E-02
GO:0032963	collagen metabolic process	51	9	4.17	2.11E-02
GO:0046129	purine ribonucleoside biosynthetic proce...	68	11	5.56	2.13E-02
GO:0042451	purine nucleoside biosynthetic process	68	11	5.56	2.13E-02
GO:0040007	growth	642	67	52.51	2.13E-02
GO:0046390	ribose phosphate biosynthetic process	151	20	12.35	2.14E-02
GO:0050673	epithelial cell proliferation	230	28	18.81	2.16E-02
GO:0032879	regulation of localization	1337	129	109.36	2.18E-02
GO:2000026	regulation of multicellular organismal d...	1054	104	86.21	2.20E-02
GO:0044711	single-organism biosynthetic process	864	87	70.67	2.22E-02
GO:0009156	ribonucleoside monophosphate biosyntheti...	60	10	4.91	2.26E-02
GO:0007507	heart development	365	41	29.85	2.27E-02
GO:0050930	induction of positive chemotaxis	14	4	1.15	2.29E-02
GO:0032488	Cdc42 protein signal transduction	14	4	1.15	2.29E-02
GO:0032489	regulation of Cdc42 protein signal trans...	14	4	1.15	2.29E-02
GO:1901701	cellular response to oxygen-containing c...	590	62	48.26	2.30E-02
GO:0061448	connective tissue development	162	21	13.25	2.33E-02
GO:0043933	macromolecular complex subunit organizat...	1136	111	92.91	2.36E-02
GO:0010631	epithelial cell migration	143	19	11.7	2.38E-02
GO:0090132	epithelium migration	143	19	11.7	2.38E-02
GO:0001935	endothelial cell proliferation	78	12	6.38	2.39E-02
GO:0002367	cytokine production involved in immune r...	44	8	3.6	2.45E-02
GO:0046686	response to cadmium ion	36	7	2.94	2.46E-02
GO:0051048	negative regulation of secretion	115	16	9.41	2.46E-02
GO:0051302	regulation of cell division	163	21	13.33	2.47E-02

GO:0006810	transport	2553	233	208.81	2.49E-02
GO:0008344	adult locomotory behavior	61	10	4.99	2.51E-02
GO:0090130	tissue migration	144	19	11.78	2.54E-02
GO:1903035	negative regulation of response to wound...	70	11	5.73	2.59E-02
GO:0007126	meiotic nuclear division	88	13	7.2	2.61E-02
GO:0044255	cellular lipid metabolic process	540	57	44.17	2.63E-02
GO:0048738	cardiac muscle tissue development	135	18	11.04	2.64E-02
GO:0030334	regulation of cell migration	422	46	34.52	2.66E-02
GO:0042246	tissue regeneration	53	9	4.33	2.66E-02
GO:0048661	positive regulation of smooth muscle cel...	53	9	4.33	2.66E-02
GO:0000278	mitotic cell cycle	476	51	38.93	2.70E-02
GO:0006461	protein complex assembly	772	78	63.14	2.74E-02
GO:0010812	negative regulation of cell-substrate ad...	29	6	2.37	2.74E-02
GO:0030199	collagen fibril organization	29	6	2.37	2.74E-02
GO:0002711	positive regulation of T cell mediated i...	45	8	3.68	2.78E-02
GO:0014909	smooth muscle cell migration	45	8	3.68	2.78E-02
GO:0044236	multicellular organismal metabolic proce...	62	10	5.07	2.79E-02
GO:0050728	negative regulation of inflammatory resp...	62	10	5.07	2.79E-02
GO:0006941	striated muscle contraction	98	14	8.02	2.79E-02
GO:0060541	respiratory system development	175	22	14.31	2.81E-02
GO:0060996	dendritic spine development	37	7	3.03	2.82E-02
GO:0046530	photoreceptor cell differentiation	37	7	3.03	2.82E-02
GO:0051896	regulation of protein kinase B signaling	89	13	7.28	2.83E-02
GO:0070271	protein complex biogenesis	774	78	63.31	2.89E-02
GO:0030574	collagen catabolic process	15	4	1.23	2.93E-02
GO:0043567	regulation of insulin-like growth factor...	15	4	1.23	2.93E-02
GO:0007413	axonal fasciculation	15	4	1.23	2.93E-02

GO:0002369	T cell cytokine production	15	4	1.23	2.93E-02
GO:0002699	positive regulation of immune effector p...	108	15	8.83	2.93E-02
GO:0008038	neuron recognition	22	5	1.8	2.95E-02
GO:0044259	multicellular organismal macromolecule m...	54	9	4.42	2.97E-02
GO:0001501	skeletal system development	309	35	25.27	3.00E-02
GO:0042592	homeostatic process	933	92	76.31	3.07E-02
GO:0015698	inorganic anion transport	63	10	5.15	3.08E-02
GO:0001508	action potential	157	20	12.84	3.10E-02
GO:0051234	establishment of localization	2638	239	215.77	3.12E-02
GO:0046058	cAMP metabolic process	72	11	5.89	3.12E-02
GO:0044264	cellular polysaccharide metabolic proces...	72	11	5.89	3.12E-02
GO:0007605	sensory perception of sound	72	11	5.89	3.12E-02
GO:0097194	execution phase of apoptosis	46	8	3.76	3.13E-02
GO:0090277	positive regulation of peptide hormone s...	46	8	3.76	3.13E-02
GO:0014068	positive regulation of phosphatidylinosi...	46	8	3.76	3.13E-02
GO:0009201	ribonucleoside triphosphate biosynthetic...	46	8	3.76	3.13E-02
GO:1901699	cellular response to nitrogen compound	331	37	27.07	3.13E-02
GO:0015909	long-chain fatty acid transport	30	6	2.45	3.20E-02
GO:0060341	regulation of cellular localization	645	66	52.76	3.21E-02
GO:0060047	heart contraction	119	16	9.73	3.27E-02
GO:0003015	heart process	119	16	9.73	3.27E-02
GO:0000079	regulation of cyclin-dependent protein s...	55	9	4.5	3.31E-02
GO:0050886	endocrine process	55	9	4.5	3.31E-02
GO:0007160	cell-matrix adhesion	129	17	10.55	3.35E-02
GO:0098602	single organism cell adhesion	270	31	22.08	3.36E-02
GO:0008015	blood circulation	281	32	22.98	3.45E-02
GO:0006928	cellular component movement	995	97	81.38	3.50E-02

GO:0015908	fatty acid transport	47	8	3.84	3.51E-02
GO:0002793	positive regulation of peptide secretion	47	8	3.84	3.51E-02
GO:0007043	cell-cell junction assembly	47	8	3.84	3.51E-02
GO:0050433	regulation of catecholamine secretion	23	5	1.88	3.53E-02
GO:0055081	anion homeostasis	23	5	1.88	3.53E-02
GO:0009266	response to temperature stimulus	130	17	10.63	3.58E-02
GO:0003013	circulatory system process	282	32	23.07	3.60E-02
GO:0044243	multicellular organismal catabolic proce...	16	4	1.31	3.66E-02
GO:0002052	positive regulation of neuroblast prolif...	16	4	1.31	3.66E-02
GO:0010762	regulation of fibroblast migration	16	4	1.31	3.66E-02
GO:0022029	telencephalon cell migration	39	7	3.19	3.66E-02
GO:0050881	musculoskeletal movement	39	7	3.19	3.66E-02
GO:0050879	multicellular organismal movement	39	7	3.19	3.66E-02
GO:0006576	cellular biogenic amine metabolic proces...	56	9	4.58	3.66E-02
GO:0009653	anatomical structure morphogenesis	1582	148	129.39	3.69E-02
GO:0060986	endocrine hormone secretion	31	6	2.54	3.70E-02
GO:0031670	cellular response to nutrient	31	6	2.54	3.70E-02
GO:0045668	negative regulation of osteoblast differ...	31	6	2.54	3.70E-02
GO:0046887	positive regulation of hormone secretion	65	10	5.32	3.73E-02
GO:0051239	regulation of multicellular organismal p...	1583	148	129.48	3.76E-02
GO:0071417	cellular response to organonitrogen comp...	304	34	24.86	3.76E-02
GO:0015850	organic hydroxy compound transport	102	14	8.34	3.77E-02
GO:0001568	blood vessel development	411	44	33.62	3.84E-02
GO:0048869	cellular developmental process	2380	216	194.66	3.85E-02
GO:1990267	response to transition metal nanoparticl...	93	13	7.61	3.89E-02
GO:0050679	positive regulation of epithelial cell p...	112	15	9.16	3.89E-02
GO:1901606	alpha-amino acid catabolic process	48	8	3.93	3.92E-02

GO:0050953	sensory perception of light stimulus	48	8	3.93	3.92E-02
GO:0001944	vasculature development	434	46	35.5	4.03E-02
GO:0009124	nucleoside monophosphate biosynthetic pr...	66	10	5.4	4.08E-02
GO:0044710	single-organism metabolic process	3517	311	287.66	4.17E-02
GO:1902692	regulation of neuroblast proliferation	24	5	1.96	4.17E-02
GO:0042129	regulation of T cell proliferation	94	13	7.69	4.19E-02
GO:0048699	generation of neurons	856	84	70.01	4.19E-02
GO:0045906	negative regulation of vasoconstriction	10	3	0.82	4.23E-02
GO:0045072	regulation of interferon-gamma biosynthe...	10	3	0.82	4.23E-02
GO:0002719	negative regulation of cytokine producti...	10	3	0.82	4.23E-02
GO:0001573	ganglioside metabolic process	10	3	0.82	4.23E-02
GO:0033275	actin-myosin filament sliding	10	3	0.82	4.23E-02
GO:0017004	cytochrome complex assembly	10	3	0.82	4.23E-02
GO:0033591	response to L-ascorbic acid	10	3	0.82	4.23E-02
GO:0061036	positive regulation of cartilage develop...	10	3	0.82	4.23E-02
GO:0019985	translesion synthesis	10	3	0.82	4.23E-02
GO:0071361	cellular response to ethanol	10	3	0.82	4.23E-02
GO:0070293	renal absorption	10	3	0.82	4.23E-02
GO:0006595	polyamine metabolic process	10	3	0.82	4.23E-02
GO:0060219	camera-type eye photoreceptor cell diffe...	10	3	0.82	4.23E-02
GO:0048240	sperm capacitation	10	3	0.82	4.23E-02
GO:0071242	cellular response to ammonium ion	10	3	0.82	4.23E-02
GO:0071243	cellular response to arsenic-containing ...	10	3	0.82	4.23E-02
GO:0051260	protein homooligomerization	203	24	16.6	4.25E-02
GO:2000179	positive regulation of neural precursor ...	32	6	2.62	4.25E-02
GO:0009887	organ morphogenesis	578	59	47.28	4.28E-02
GO:0051146	striated muscle cell differentiation	193	23	15.79	4.29E-02

GO:0051049	regulation of transport	960	93	78.52	4.41E-02
GO:2000177	regulation of neural precursor cell prol...	58	9	4.74	4.46E-02
GO:0051897	positive regulation of protein kinase B ...	58	9	4.74	4.46E-02
GO:0006007	glucose catabolic process	17	4	1.39	4.48E-02
GO:0035235	ionotropic glutamate receptor signaling ...	17	4	1.39	4.48E-02
GO:0014850	response to muscle activity	17	4	1.39	4.48E-02
GO:0043618	regulation of transcription from RNA pol...	17	4	1.39	4.48E-02
GO:0090190	positive regulation of branching involve...	17	4	1.39	4.48E-02
GO:0090189	regulation of branching involved in uret...	17	4	1.39	4.48E-02
GO:0061138	morphogenesis of a branching epithelium	154	19	12.6	4.61E-02
GO:0021885	forebrain cell migration	41	7	3.35	4.65E-02
GO:0097306	cellular response to alcohol	86	12	7.03	4.65E-02
GO:0008284	positive regulation of cell proliferatio...	548	56	44.82	4.67E-02
GO:0010817	regulation of hormone levels	257	29	21.02	4.71E-02
GO:0008217	regulation of blood pressure	115	15	9.41	4.75E-02
GO:0045665	negative regulation of neuron differenti...	50	8	4.09	4.84E-02
GO:0022408	negative regulation of cell-cell adhesio...	33	6	2.7	4.85E-02
GO:0032663	regulation of interleukin-2 production	33	6	2.7	4.85E-02
GO:0051937	catecholamine transport	33	6	2.7	4.85E-02
GO:0007215	glutamate receptor signaling pathway	33	6	2.7	4.85E-02
GO:0009250	glucan biosynthetic process	33	6	2.7	4.85E-02
GO:0005978	glycogen biosynthetic process	33	6	2.7	4.85E-02
GO:0021772	olfactory bulb development	25	5	2.04	4.87E-02
GO:0050432	catecholamine secretion	25	5	2.04	4.87E-02
GO:1902305	regulation of sodium ion transmembrane t...	25	5	2.04	4.87E-02
GO:0034599	cellular response to oxidative stress	155	19	12.68	4.87E-02
GO:0014066	regulation of phosphatidylinositol 3-kin...	59	9	4.83	4.90E-02
