# Supplementary material for: A robust neuromuscular system protects rat and human skeletal muscle from sarcopenia
Source: Aging (Albany NY). 2016 Mar 24;8(4):712–28. doi: 10.18632/aging.100926 (PMC4925824; doi:10.18632/aging.100926)
Supplement: Supplementary file 5 [file aging-08-712-s005.pdf]

Gene Ontology ID	Gene Ontology term (Biological Process)	Number of annotated genes	Number of significant genes	Number of expected genes	p-value (Fisher test)
GO:0002376	immune system process	1210	38	15.96	1.80E-07
GO:0006952	defense response	617	24	8.14	1.50E-06
GO:0002252	immune effector process	331	17	4.37	1.50E-06
GO:0006955	immune response	598	22	7.89	1.00E-05
GO:0001775	cell activation	525	20	6.92	1.70E-05
GO:0002456	T cell mediated immunity	65	7	0.86	2.20E-05
GO:0046649	lymphocyte activation	372	16	4.91	3.00E-05
GO:0006950	response to stress	2004	46	26.43	4.70E-05
GO:0001932	regulation of protein phosphorylation	662	22	8.73	5.00E-05
GO:0043903	regulation of symbiosis, encompassing mu...	104	8	1.37	6.60E-05
GO:0045321	leukocyte activation	443	17	5.84	7.00E-05
GO:0042592	homeostatic process	933	27	12.31	7.10E-05
GO:0044699	single-organism process	7957	122	104.94	7.10E-05
GO:0048878	chemical homeostasis	600	20	7.91	1.10E-04
GO:0051338	regulation of transferase activity	508	18	6.7	1.20E-04
GO:0050792	regulation of viral process	87	7	1.15	1.40E-04
GO:0042110	T cell activation	257	12	3.39	1.40E-04
GO:0043549	regulation of kinase activity	472	17	6.23	1.50E-04
GO:0019058	viral life cycle	117	8	1.54	1.50E-04
GO:0044763	single-organism cellular process	7096	112	93.59	1.80E-04
GO:0031399	regulation of protein modification proce...	826	24	10.89	1.80E-04
GO:0042325	regulation of phosphorylation	837	24	11.04	2.20E-04
GO:0051146	striated muscle cell differentiation	193	10	2.55	2.30E-04
GO:0002367	cytokine production involved in immune r...	44	5	0.58	2.70E-04
GO:0010033	response to organic substance	1657	38	21.85	2.90E-04
GO:0000768	syncytium formation by plasma membrane f...	25	4	0.33	3.00E-04
GO:0000096	sulfur amino acid metabolic process	25	4	0.33	3.00E-04
GO:0070887	cellular response to chemical stimulus	1363	33	17.98	3.10E-04
GO:0007050	cell cycle arrest	100	7	1.32	3.40E-04
GO:0034097	response to cytokine	415	15	5.47	3.60E-04
GO:0006949	syncytium formation	27	4	0.36	4.00E-04
GO:0044419	interspecies interaction between organis...	170	9	2.24	4.10E-04
GO:0044403	symbiosis, encompassing mutualism throug...	170	9	2.24	4.10E-04
GO:0043901	negative regulation of multi-organism pr...	76	6	1	4.80E-04

GO:0048525	negative regulation of viral process	51	5	0.67	5.30E-04
GO:0016032	viral process	141	8	1.86	5.40E-04
GO:0002449	lymphocyte mediated immunity	142	8	1.87	5.70E-04
GO:0032268	regulation of cellular protein metabolic...	1060	27	13.98	5.70E-04
GO:0048002	antigen processing and presentation of p...	52	5	0.69	5.90E-04
GO:0002709	regulation of T cell mediated immunity	52	5	0.69	5.90E-04
GO:0007568	aging	258	11	3.4	6.00E-04
GO:0045859	regulation of protein kinase activity	436	15	5.75	6.10E-04
GO:0006811	ion transport	846	23	11.16	6.40E-04
GO:0019882	antigen processing and presentation	81	6	1.07	6.80E-04
GO:2000278	regulation of DNA biosynthetic process	31	4	0.41	6.90E-04
GO:0002682	regulation of immune system process	639	19	8.43	6.90E-04
GO:0044764	multi-organism cellular process	148	8	1.95	7.40E-04
GO:0009615	response to virus	149	8	1.97	7.80E-04
GO:0003009	skeletal muscle contraction	32	4	0.42	7.80E-04
GO:0001816	cytokine production	354	13	4.67	7.90E-04
GO:0034341	response to interferon-gamma	56	5	0.74	8.20E-04
GO:0045087	innate immune response	269	11	3.55	8.40E-04
GO:0002369	T cell cytokine production	15	3	0.2	9.10E-04
GO:0002474	antigen processing and presentation of p...	34	4	0.45	9.90E-04
GO:0071345	cellular response to cytokine stimulus	321	12	4.23	1.08E-03
GO:0090279	regulation of calcium ion import	16	3	0.21	1.11E-03
GO:0002706	regulation of lymphocyte mediated immuni...	90	6	1.19	1.18E-03
GO:2000107	negative regulation of leukocyte apoptot...	36	4	0.47	1.23E-03
GO:0007159	leukocyte cell-cell adhesion	36	4	0.47	1.23E-03
GO:0006875	cellular metal ion homeostasis	282	11	3.72	1.24E-03
GO:0010959	regulation of metal ion transport	199	9	2.62	1.25E-03
GO:0055065	metal ion homeostasis	328	12	4.33	1.30E-03
GO:0065008	regulation of biological quality	1978	41	26.09	1.30E-03
GO:0002440	production of molecular mediator of immu...	92	6	1.21	1.33E-03
GO:0010942	positive regulation of cell death	330	12	4.35	1.37E-03
GO:0051607	defense response to virus	93	6	1.23	1.40E-03
GO:0080134	regulation of response to stress	626	18	8.26	1.42E-03
GO:0002250	adaptive immune response	164	8	2.16	1.45E-03

GO:0070374	positive regulation of ERK1 and ERK2 cas...	95	6	1.25	1.57E-03
GO:0032897	negative regulation of viral transcripti...	18	3	0.24	1.58E-03
GO:0006812	cation transport	635	18	8.38	1.66E-03
GO:0050881	musculoskeletal movement	39	4	0.51	1.66E-03
GO:0050879	multicellular organismal movement	39	4	0.51	1.66E-03
GO:0050776	regulation of immune response	339	12	4.47	1.71E-03
GO:0051240	positive regulation of multicellular org...	435	14	5.74	1.76E-03
GO:0055082	cellular chemical homeostasis	388	13	5.12	1.81E-03
GO:0006941	striated muscle contraction	98	6	1.29	1.84E-03
GO:0042692	muscle cell differentiation	254	10	3.35	1.93E-03
GO:0051924	regulation of calcium ion transport	134	7	1.77	1.94E-03
GO:0001819	positive regulation of cytokine producti...	173	8	2.28	2.03E-03
GO:0002819	regulation of adaptive immune response	100	6	1.32	2.04E-03
GO:0006915	apoptotic process	1156	27	15.25	2.11E-03
GO:0034612	response to tumor necrosis factor	101	6	1.33	2.14E-03
GO:0002684	positive regulation of immune system pro...	396	13	5.22	2.16E-03
GO:0043068	positive regulation of programmed cell d...	303	11	4	2.19E-03
GO:0002697	regulation of immune effector process	176	8	2.32	2.26E-03
GO:0043900	regulation of multi-organism process	176	8	2.32	2.26E-03
GO:0030003	cellular cation homeostasis	305	11	4.02	2.31E-03
GO:0048871	multicellular organismal homeostasis	177	8	2.33	2.34E-03
GO:0001817	regulation of cytokine production	306	11	4.04	2.37E-03
GO:0006816	calcium ion transport	220	9	2.9	2.50E-03
GO:0002443	leukocyte mediated immunity	179	8	2.36	2.51E-03
GO:0009605	response to external stimulus	1230	28	16.22	2.51E-03
GO:0030282	bone mineralization	72	5	0.95	2.55E-03
GO:0098542	defense response to other organism	180	8	2.37	2.60E-03
GO:0006873	cellular ion homeostasis	311	11	4.1	2.68E-03
GO:0042221	response to chemical	2119	42	27.95	2.71E-03
GO:0030001	metal ion transport	457	14	6.03	2.77E-03
GO:0012501	programmed cell death	1180	27	15.56	2.83E-03
GO:0002711	positive regulation of T cell mediated i...	45	4	0.59	2.84E-03
GO:0030595	leukocyte chemotaxis	107	6	1.41	2.86E-03
GO:0050801	ion homeostasis	409	13	5.39	2.86E-03

GO:0043067	regulation of programmed cell death	948	23	12.5	2.92E-03
GO:0055080	cation homeostasis	362	12	4.77	2.95E-03
GO:0010941	regulation of cell death	1007	24	13.28	2.96E-03
GO:0045786	negative regulation of cell cycle	184	8	2.43	2.98E-03
GO:0002703	regulation of leukocyte mediated immunit...	108	6	1.42	3.00E-03
GO:0034113	heterotypic cell-cell adhesion	23	3	0.3	3.28E-03
GO:0070229	negative regulation of lymphocyte apopto...	23	3	0.3	3.28E-03
GO:0055076	transition metal ion homeostasis	78	5	1.03	3.62E-03
GO:0001960	negative regulation of cytokine-mediated...	24	3	0.32	3.71E-03
GO:0032269	negative regulation of cellular protein ...	374	12	4.93	3.84E-03
GO:0070838	divalent metal ion transport	236	9	3.11	3.98E-03
GO:0002460	adaptive immune response based on somati...	153	7	2.02	4.08E-03
GO:0001910	regulation of leukocyte mediated cytotox...	50	4	0.66	4.17E-03
GO:0045214	sarcomere organization	25	3	0.33	4.17E-03
GO:0071310	cellular response to organic substance	1096	25	14.46	4.30E-03
GO:0072511	divalent inorganic cation transport	241	9	3.18	4.57E-03
GO:0006954	inflammatory response	334	11	4.41	4.61E-03
GO:0009408	response to heat	83	5	1.09	4.73E-03
GO:0055072	iron ion homeostasis	52	4	0.69	4.80E-03
GO:0030099	myeloid cell differentiation	243	9	3.2	4.82E-03
GO:0042098	T cell proliferation	119	6	1.57	4.84E-03
GO:0050900	leukocyte migration	159	7	2.1	5.03E-03
GO:0046916	cellular transition metal ion homeostasi...	53	4	0.7	5.14E-03
GO:0046688	response to copper ion	27	3	0.36	5.21E-03
GO:0060761	negative regulation of response to cytok...	27	3	0.36	5.21E-03
GO:0043269	regulation of ion transport	340	11	4.48	5.26E-03
GO:0042981	regulation of apoptotic process	937	22	12.36	5.36E-03
GO:0031214	biomineral tissue development	86	5	1.13	5.49E-03
GO:0031341	regulation of cell killing	54	4	0.71	5.50E-03
GO:0030335	positive regulation of cell migration	249	9	3.28	5.64E-03
GO:0008219	cell death	1242	27	16.38	5.72E-03
GO:0014902	myotube differentiation	87	5	1.15	5.77E-03
GO:0045123	cellular extravasation	28	3	0.37	5.77E-03
GO:0050732	negative regulation of peptidyl-tyrosine...	28	3	0.37	5.77E-03
GO:0046651	lymphocyte proliferation	164	7	2.16	5.95E-03
GO:0016265	death	1247	27	16.45	6.04E-03

GO:0043065	positive regulation of apoptotic process	299	10	3.94	6.15E-03
GO:0032943	mononuclear cell proliferation	165	7	2.18	6.15E-03
GO:0071897	DNA biosynthetic process	56	4	0.74	6.26E-03
GO:0001894	tissue homeostasis	126	6	1.66	6.37E-03
GO:0006879	cellular iron ion homeostasis	29	3	0.38	6.38E-03
GO:0046782	regulation of viral transcription	29	3	0.38	6.38E-03
GO:2000147	positive regulation of cell motility	254	9	3.35	6.40E-03
GO:0010035	response to inorganic substance	401	12	5.29	6.65E-03
GO:0097529	myeloid leukocyte migration	91	5	1.2	6.96E-03
GO:0019725	cellular homeostasis	455	13	6	6.98E-03
GO:0030154	cell differentiation	2229	42	29.4	7.00E-03
GO:0006826	iron ion transport	30	3	0.4	7.02E-03
GO:0019080	viral gene expression	30	3	0.4	7.02E-03
GO:0019083	viral transcription	30	3	0.4	7.02E-03
GO:0002700	regulation of production of molecular me...	58	4	0.76	7.08E-03
GO:0050670	regulation of lymphocyte proliferation	129	6	1.7	7.13E-03
GO:0070661	leukocyte proliferation	170	7	2.24	7.20E-03
GO:0050667	homocysteine metabolic process	10	2	0.13	7.25E-03
GO:0043922	negative regulation by host of viral tra...	10	2	0.13	7.25E-03
GO:0048305	immunoglobulin secretion	10	2	0.13	7.25E-03
GO:1900744	regulation of p38MAPK cascade	10	2	0.13	7.25E-03
GO:1900027	regulation of ruffle assembly	10	2	0.13	7.25E-03
GO:0006898	receptor-mediated endocytosis	130	6	1.71	7.40E-03
GO:0032944	regulation of mononuclear cell prolifera...	130	6	1.71	7.40E-03
GO:0010038	response to metal ion	260	9	3.43	7.42E-03
GO:0030097	hemopoiesis	513	14	6.77	7.64E-03
GO:0001933	negative regulation of protein phosphory...	216	8	2.85	7.75E-03
GO:0051272	positive regulation of cellular componen...	262	9	3.46	7.79E-03
GO:0040017	positive regulation of locomotion	262	9	3.46	7.79E-03
GO:0070663	regulation of leukocyte proliferation	132	6	1.74	7.95E-03
GO:0048584	positive regulation of response to stimu...	1030	23	13.58	8.02E-03
GO:0051347	positive regulation of transferase activ...	311	10	4.1	8.02E-03
GO:0002822	regulation of adaptive immune response b...	95	5	1.25	8.32E-03

GO:2000106	regulation of leukocyte apoptotic proces...	61	4	0.8	8.45E-03
GO:0071621	granulocyte chemotaxis	61	4	0.8	8.45E-03
GO:0002708	positive regulation of lymphocyte mediat...	61	4	0.8	8.45E-03
GO:0001776	leukocyte homeostasis	61	4	0.8	8.45E-03
GO:0048872	homeostasis of number of cells	176	7	2.32	8.65E-03
GO:0060249	anatomical structure homeostasis	176	7	2.32	8.65E-03
GO:0065007	biological regulation	6249	96	82.42	8.74E-03
GO:0010831	positive regulation of myotube different...	11	2	0.15	8.78E-03
GO:0014874	response to stimulus involved in regulat...	11	2	0.15	8.78E-03
GO:1902187	negative regulation of viral release fro...	11	2	0.15	8.78E-03
GO:0038066	p38MAPK cascade	11	2	0.15	8.78E-03
GO:0048875	chemical homeostasis within a tissue	11	2	0.15	8.78E-03
GO:0002724	regulation of T cell cytokine production	11	2	0.15	8.78E-03
GO:0002687	positive regulation of leukocyte migrati...	62	4	0.82	8.94E-03
GO:0002824	positive regulation of adaptive immune r...	62	4	0.82	8.94E-03
GO:0045860	positive regulation of protein kinase ac...	268	9	3.53	8.97E-03
GO:0048524	positive regulation of viral process	33	3	0.44	9.17E-03
GO:0019221	cytokine-mediated signaling pathway	178	7	2.35	9.17E-03
GO:0001934	positive regulation of protein phosphory...	420	12	5.54	9.45E-03
GO:0001909	leukocyte mediated cytotoxicity	63	4	0.83	9.45E-03
GO:0002705	positive regulation of leukocyte mediate...	63	4	0.83	9.45E-03
GO:0002821	positive regulation of adaptive immune r...	63	4	0.83	9.45E-03
GO:0070372	regulation of ERK1 and ERK2 cascade	137	6	1.81	9.46E-03
GO:0003012	muscle system process	225	8	2.97	9.79E-03
GO:0044033	multi-organism metabolic process	34	3	0.45	9.96E-03
GO:0030239	myofibril assembly	34	3	0.45	9.96E-03
GO:0097530	granulocyte migration	64	4	0.84	9.98E-03
GO:0051249	regulation of lymphocyte activation	226	8	2.98	1.00E-02
GO:0014854	response to inactivity	12	2	0.16	1.05E-02
GO:0060333	interferon-gamma-mediated signaling path...	12	2	0.16	1.05E-02
GO:0060307	regulation of ventricular cardiac muscle...	12	2	0.16	1.05E-02

GO:0045070	positive regulation of viral genome repl...	12	2	0.16	1.05E-02
GO:0051248	negative regulation of protein metabolic...	426	12	5.62	1.05E-02
GO:0001916	positive regulation of T cell mediated c...	35	3	0.46	1.08E-02
GO:0002718	regulation of cytokine production involv...	35	3	0.46	1.08E-02
GO:0045069	regulation of viral genome replication	35	3	0.46	1.08E-02
GO:0048534	hematopoietic or lymphoid organ developm...	536	14	7.07	1.10E-02
GO:0033500	carbohydrate homeostasis	143	6	1.89	1.15E-02
GO:0042593	glucose homeostasis	143	6	1.89	1.15E-02
GO:1901264	carbohydrate derivative transport	36	3	0.47	1.17E-02
GO:0001914	regulation of T cell mediated cytotoxici...	36	3	0.47	1.17E-02
GO:0002286	T cell activation involved in immune res...	36	3	0.47	1.17E-02
GO:0060326	cell chemotaxis	144	6	1.9	1.19E-02
GO:0031400	negative regulation of protein modificat...	281	9	3.71	1.20E-02
GO:0031347	regulation of defense response	281	9	3.71	1.20E-02
GO:0060009	Sertoli cell development	13	2	0.17	1.22E-02
GO:0002820	negative regulation of adaptive immune r...	13	2	0.17	1.22E-02
GO:0071900	regulation of protein serine/threonine k...	282	9	3.72	1.23E-02
GO:0050730	regulation of peptidyl-tyrosine phosphor...	145	6	1.91	1.23E-02
GO:0019220	regulation of phosphate metabolic proces...	1258	26	16.59	1.27E-02
GO:0072503	cellular divalent inorganic cation homeo...	236	8	3.11	1.28E-02
GO:0007229	integrin-mediated signaling pathway	69	4	0.91	1.29E-02
GO:0006468	protein phosphorylation	1015	22	13.39	1.32E-02
GO:0048869	cellular developmental process	2380	43	31.39	1.32E-02
GO:0051174	regulation of phosphorus metabolic proce...	1263	26	16.66	1.33E-02
GO:0033674	positive regulation of kinase activity	286	9	3.77	1.33E-02
GO:0048518	positive regulation of biological proces...	3007	52	39.66	1.35E-02
GO:0070371	ERK1 and ERK2 cascade	148	6	1.95	1.35E-02
GO:2000379	positive regulation of reactive oxygen s...	38	3	0.5	1.35E-02
GO:0006801	superoxide metabolic process	38	3	0.5	1.35E-02
GO:0070509	calcium ion import	38	3	0.5	1.35E-02
GO:0002699	positive regulation of immune effector p...	108	5	1.42	1.40E-02

GO:0060338	regulation of type I interferon-mediated...	14	2	0.18	1.42E-02
GO:0000097	sulfur amino acid biosynthetic process	14	2	0.18	1.42E-02
GO:0055094	response to lipoprotein particle	14	2	0.18	1.42E-02
GO:0070233	negative regulation of T cell apoptotic ...	14	2	0.18	1.42E-02
GO:0007569	cell aging	71	4	0.94	1.42E-02
GO:0051246	regulation of protein metabolic process	1334	27	17.59	1.44E-02
GO:0071156	regulation of cell cycle arrest	39	3	0.51	1.45E-02
GO:0043902	positive regulation of multi-organism pr...	39	3	0.51	1.45E-02
GO:0001913	T cell mediated cytotoxicity	39	3	0.51	1.45E-02
GO:0043491	protein kinase B signaling	109	5	1.44	1.45E-02
GO:0051094	positive regulation of developmental pro...	669	16	8.82	1.48E-02
GO:0001906	cell killing	72	4	0.95	1.49E-02
GO:0055007	cardiac muscle cell differentiation	72	4	0.95	1.49E-02
GO:0042632	cholesterol homeostasis	40	3	0.53	1.55E-02
GO:0071887	leukocyte apoptotic process	73	4	0.96	1.56E-02
GO:0001503	ossification	294	9	3.88	1.57E-02
GO:0048583	regulation of response to stimulus	2065	38	27.24	1.58E-02
GO:0030574	collagen catabolic process	15	2	0.2	1.62E-02
GO:0061028	establishment of endothelial barrier	15	2	0.2	1.62E-02
GO:0086005	ventricular cardiac muscle cell action p...	15	2	0.2	1.62E-02
GO:0055092	sterol homeostasis	41	3	0.54	1.66E-02
GO:0071346	cellular response to interferon-gamma	41	3	0.54	1.66E-02
GO:0019079	viral genome replication	41	3	0.54	1.66E-02
GO:0002520	immune system development	566	14	7.46	1.70E-02
GO:0072507	divalent inorganic cation homeostasis	249	8	3.28	1.72E-02
GO:0030029	actin filament-based process	404	11	5.33	1.77E-02
GO:0051704	multi-organism process	1044	22	13.77	1.78E-02
GO:0023014	signal transduction by phosphorylation	460	12	6.07	1.83E-02
GO:0060008	Sertoli cell differentiation	16	2	0.21	1.84E-02
GO:0044243	multicellular organismal catabolic proce...	16	2	0.21	1.84E-02
GO:0097178	ruffle assembly	16	2	0.21	1.84E-02
GO:0002707	negative regulation of lymphocyte mediat...	16	2	0.21	1.84E-02
GO:0070228	regulation of lymphocyte apoptotic proce...	43	3	0.57	1.89E-02
GO:0032970	regulation of actin filament-based proce...	207	7	2.73	1.97E-02

GO:1902533	positive regulation of intracellular sig...	521	13	6.87	1.99E-02
GO:0051817	modification of morphology or physiology...	44	3	0.58	2.00E-02
GO:0071479	cellular response to ionizing radiation	44	3	0.58	2.00E-02
GO:0061061	muscle structure development	412	11	5.43	2.02E-02
GO:0002521	leukocyte differentiation	307	9	4.05	2.02E-02
GO:0043921	modulation by host of viral transcriptio...	17	2	0.22	2.06E-02
GO:1902186	regulation of viral release from host ce...	17	2	0.22	2.06E-02
GO:0060337	type I interferon signaling pathway	17	2	0.22	2.06E-02
GO:0006471	protein ADP-ribosylation	17	2	0.22	2.06E-02
GO:0052472	modulation by host of symbiont transcrip...	17	2	0.22	2.06E-02
GO:0002704	negative regulation of leukocyte mediate...	17	2	0.22	2.06E-02
GO:0071357	cellular response to type I interferon	17	2	0.22	2.06E-02
GO:0051926	negative regulation of calcium ion trans...	17	2	0.22	2.06E-02
GO:0051707	response to other organism	468	12	6.17	2.06E-02
GO:0043207	response to external biotic stimulus	468	12	6.17	2.06E-02
GO:0042326	negative regulation of phosphorylation	258	8	3.4	2.08E-02
GO:0055002	striated muscle cell development	120	5	1.58	2.11E-02
GO:0002526	acute inflammatory response	80	4	1.06	2.12E-02
GO:0046330	positive regulation of JNK cascade	45	3	0.59	2.13E-02
GO:0043502	regulation of muscle adaptation	45	3	0.59	2.13E-02
GO:0031401	positive regulation of protein modificat...	528	13	6.96	2.19E-02
GO:0002690	positive regulation of leukocyte chemota...	46	3	0.61	2.25E-02
GO:0044092	negative regulation of molecular functio...	588	14	7.76	2.29E-02
GO:0070555	response to interleukin-1	82	4	1.08	2.30E-02
GO:0071356	cellular response to tumor necrosis fact...	82	4	1.08	2.30E-02
GO:0002694	regulation of leukocyte activation	263	8	3.47	2.30E-02
GO:0046640	regulation of alpha-beta T cell prolifer...	18	2	0.24	2.30E-02
GO:0048247	lymphocyte chemotaxis	18	2	0.24	2.30E-02
GO:0052312	modulation of transcription in other org...	18	2	0.24	2.30E-02
GO:0051354	negative regulation of oxidoreductase ac...	18	2	0.24	2.30E-02

GO:1901016	regulation of potassium ion transmembran...	18	2	0.24	2.30E-02
GO:0019076	viral release from host cell	18	2	0.24	2.30E-02
GO:0051239	regulation of multicellular organismal p...	1583	30	20.88	2.30E-02
GO:0072593	reactive oxygen species metabolic proces...	123	5	1.62	2.32E-02
GO:0030500	regulation of bone mineralization	47	3	0.62	2.39E-02
GO:0001912	positive regulation of leukocyte mediate...	47	3	0.62	2.39E-02
GO:0031032	actomyosin structure organization	47	3	0.62	2.39E-02
GO:0035821	modification of morphology or physiology...	47	3	0.62	2.39E-02
GO:0044765	single-organism transport	2124	38	28.01	2.41E-02
GO:0032956	regulation of actin cytoskeleton organiz...	169	6	2.23	2.43E-02
GO:0043410	positive regulation of MAPK cascade	267	8	3.52	2.49E-02
GO:0006790	sulfur compound metabolic process	170	6	2.24	2.49E-02
GO:0042127	regulation of cell proliferation	956	20	12.61	2.54E-02
GO:0007520	myoblast fusion	19	2	0.25	2.55E-02
GO:0042092	type 2 immune response	19	2	0.25	2.55E-02
GO:0045736	negative regulation of cyclin-dependent ...	19	2	0.25	2.55E-02
GO:0034340	response to type I interferon	19	2	0.25	2.55E-02
GO:0071359	cellular response to dsRNA	19	2	0.25	2.55E-02
GO:0032412	regulation of ion transmembrane transpor...	85	4	1.12	2.58E-02
GO:0002685	regulation of leukocyte migration	85	4	1.12	2.58E-02
GO:0051092	positive regulation of NF-kappaB transcr...	85	4	1.12	2.58E-02
GO:0009607	response to biotic stimulus	484	12	6.38	2.60E-02
GO:0006936	muscle contraction	172	6	2.27	2.62E-02
GO:0006575	cellular modified amino acid metabolic p...	127	5	1.68	2.62E-02
GO:0055001	muscle cell development	127	5	1.68	2.62E-02
GO:0045088	regulation of innate immune response	127	5	1.68	2.62E-02
GO:0046634	regulation of alpha-beta T cell activati...	49	3	0.65	2.66E-02
GO:0006810	transport	2553	44	33.67	2.68E-02
GO:0050671	positive regulation of lymphocyte prolif...	86	4	1.13	2.68E-02
GO:0035051	cardiocyte differentiation	86	4	1.13	2.68E-02
GO:0009888	tissue development	1217	24	16.05	2.77E-02
GO:0032946	positive regulation of mononuclear cell ...	87	4	1.15	2.78E-02

GO:0032872	regulation of stress-activated MAPK casc...	129	5	1.7	2.78E-02
GO:0007160	cell-matrix adhesion	129	5	1.7	2.78E-02
GO:0030593	neutrophil chemotaxis	50	3	0.66	2.80E-02
GO:0015748	organophosphate ester transport	50	3	0.66	2.80E-02
GO:0070167	regulation of biomineral tissue developm...	50	3	0.66	2.80E-02
GO:0002478	antigen processing and presentation of e...	20	2	0.26	2.81E-02
GO:0060306	regulation of membrane repolarization	20	2	0.26	2.81E-02
GO:0030947	regulation of vascular endothelial growt...	20	2	0.26	2.81E-02
GO:0006874	cellular calcium ion homeostasis	223	7	2.94	2.81E-02
GO:0034765	regulation of ion transmembrane transpor...	175	6	2.31	2.82E-02
GO:0009266	response to temperature stimulus	130	5	1.71	2.86E-02
GO:0090257	regulation of muscle system process	130	5	1.71	2.86E-02
GO:0070302	regulation of stress-activated protein k...	130	5	1.71	2.86E-02
GO:0051090	regulation of sequence-specific DNA bind...	224	7	2.95	2.87E-02
GO:0065009	regulation of molecular function	1614	30	21.29	2.92E-02
GO:1990266	neutrophil migration	51	3	0.67	2.95E-02
GO:0002260	lymphocyte homeostasis	51	3	0.67	2.95E-02
GO:0031343	positive regulation of cell killing	51	3	0.67	2.95E-02
GO:0016310	phosphorylation	1354	26	17.86	2.97E-02
GO:0051896	regulation of protein kinase B signaling	89	4	1.17	2.99E-02
GO:0070665	positive regulation of leukocyte prolife...	89	4	1.17	2.99E-02
GO:0022898	regulation of transmembrane transporter ...	89	4	1.17	2.99E-02
GO:0030307	positive regulation of cell growth	89	4	1.17	2.99E-02
GO:0046633	alpha-beta T cell proliferation	21	2	0.28	3.08E-02
GO:0002720	positive regulation of cytokine producti...	21	2	0.28	3.08E-02
GO:0086002	cardiac muscle cell action potential inv...	21	2	0.28	3.08E-02
GO:0045446	endothelial cell differentiation	52	3	0.69	3.10E-02
GO:0001959	regulation of cytokine-mediated signalin...	52	3	0.69	3.10E-02
GO:0050777	negative regulation of immune response	52	3	0.69	3.10E-02

GO:0002573	myeloid leukocyte differentiation	133	5	1.75	3.12E-02
GO:0000165	MAPK cascade	441	11	5.82	3.12E-02
GO:0006942	regulation of striated muscle contractio...	53	3	0.7	3.26E-02
GO:0050790	regulation of catalytic activity	1301	25	17.16	3.27E-02
GO:0014706	striated muscle tissue development	282	8	3.72	3.30E-02
GO:0055074	calcium ion homeostasis	231	7	3.05	3.31E-02
GO:2000514	regulation of CD4-positive, alpha-beta T...	22	2	0.29	3.36E-02
GO:0045071	negative regulation of viral genome repl...	22	2	0.29	3.36E-02
GO:0010563	negative regulation of phosphorus metabo...	283	8	3.73	3.36E-02
GO:0045936	negative regulation of phosphate metabol...	283	8	3.73	3.36E-02
GO:0001558	regulation of cell growth	232	7	3.06	3.38E-02
GO:0071560	cellular response to transforming growth...	136	5	1.79	3.39E-02
GO:0044707	single-multicellular organism process	3667	59	48.36	3.44E-02
GO:0032270	positive regulation of cellular protein ...	622	14	8.2	3.47E-02
GO:0022603	regulation of anatomical structure morph...	564	13	7.44	3.50E-02
GO:0009169	purine ribonucleoside monophosphate cata...	286	8	3.77	3.54E-02
GO:0009158	ribonucleoside monophosphate catabolic p...	286	8	3.77	3.54E-02
GO:0050865	regulation of cell activation	286	8	3.77	3.54E-02
GO:0042129	regulation of T cell proliferation	94	4	1.24	3.55E-02
GO:0034762	regulation of transmembrane transport	185	6	2.44	3.56E-02
GO:0071559	response to transforming growth factor b...	138	5	1.82	3.57E-02
GO:0002688	regulation of leukocyte chemotaxis	55	3	0.73	3.58E-02
GO:0043271	negative regulation of ion transport	55	3	0.73	3.58E-02
GO:0000079	regulation of cyclin-dependent protein s...	55	3	0.73	3.58E-02
GO:0070227	lymphocyte apoptotic process	55	3	0.73	3.58E-02
GO:0042327	positive regulation of phosphorylation	508	12	6.7	3.58E-02
GO:0009128	purine nucleoside monophosphate cataboli...	287	8	3.79	3.60E-02
GO:0009628	response to abiotic stimulus	808	17	10.66	3.66E-02
GO:0009125	nucleoside monophosphate catabolic proce...	289	8	3.81	3.73E-02

GO:0000041	transition metal ion transport	56	3	0.74	3.75E-02
GO:0071347	cellular response to interleukin-1	56	3	0.74	3.75E-02
GO:0032409	regulation of transporter activity	96	4	1.27	3.79E-02
GO:0070588	calcium ion transmembrane transport	96	4	1.27	3.79E-02
GO:0043408	regulation of MAPK cascade	399	10	5.26	3.81E-02
GO:0071396	cellular response to lipid	291	8	3.84	3.86E-02
GO:0050789	regulation of biological process	5958	89	78.58	3.88E-02
GO:0032874	positive regulation of stress-activated ...	57	3	0.75	3.92E-02
GO:0060759	regulation of response to cytokine stimu...	57	3	0.75	3.92E-02
GO:0051247	positive regulation of protein metabolic...	693	15	9.14	3.93E-02
GO:0016049	cell growth	292	8	3.85	3.93E-02
GO:0071158	positive regulation of cell cycle arrest	24	2	0.32	3.94E-02
GO:1901379	regulation of potassium ion transmembran...	24	2	0.32	3.94E-02
GO:0051851	modification by host of symbiont morphol...	24	2	0.32	3.94E-02
GO:0086001	cardiac muscle cell action potential	24	2	0.32	3.94E-02
GO:0006469	negative regulation of protein kinase ac...	142	5	1.87	3.96E-02
GO:0006796	phosphate-containing compound metabolic ...	2548	43	33.61	3.97E-02
GO:0051897	positive regulation of protein kinase B ...	58	3	0.76	4.09E-02
GO:0070304	positive regulation of stress-activated ...	58	3	0.76	4.09E-02
GO:0000086	G2/M transition of mitotic cell cycle	58	3	0.76	4.09E-02
GO:0070482	response to oxygen levels	295	8	3.89	4.13E-02
GO:0042113	B cell activation	144	5	1.9	4.17E-02
GO:0048588	developmental cell growth	99	4	1.31	4.17E-02
GO:1902305	regulation of sodium ion transmembrane t...	25	2	0.33	4.25E-02
GO:0070534	protein K63-linked ubiquitination	25	2	0.33	4.25E-02
GO:0051702	interaction with symbiont	25	2	0.33	4.25E-02
GO:0070296	sarcoplasmic reticulum calcium ion trans...	25	2	0.33	4.25E-02
GO:0086009	membrane repolarization	25	2	0.33	4.25E-02
GO:0002702	positive regulation of production of mol...	25	2	0.33	4.25E-02
GO:0070232	regulation of T cell apoptotic process	25	2	0.33	4.25E-02
GO:0019884	antigen processing and presentation of e...	25	2	0.33	4.25E-02

GO:0051155	positive regulation of striated muscle c...	25	2	0.33	4.25E-02
GO:0042787	protein ubiquitination involved in ubiqu...	59	3	0.78	4.27E-02
GO:0042770	signal transduction in response to DNA d...	59	3	0.78	4.27E-02
GO:0060537	muscle tissue development	298	8	3.93	4.34E-02
GO:0051403	stress-activated MAPK cascade	146	5	1.93	4.38E-02
GO:0032501	multicellular organismal process	3788	60	49.96	4.42E-02
GO:0051234	establishment of localization	2638	44	34.79	4.45E-02
GO:0006641	triglyceride metabolic process	60	3	0.79	4.45E-02
GO:0044839	cell cycle G2/M phase transition	60	3	0.79	4.45E-02
GO:0055088	lipid homeostasis	60	3	0.79	4.45E-02
GO:0030316	osteoclast differentiation	60	3	0.79	4.45E-02
GO:0051258	protein polymerization	147	5	1.94	4.49E-02
GO:0030890	positive regulation of B cell proliferat...	26	2	0.34	4.57E-02
GO:0030501	positive regulation of bone mineralizati...	26	2	0.34	4.57E-02
GO:0045429	positive regulation of nitric oxide bios...	26	2	0.34	4.57E-02
GO:0050798	activated T cell proliferation	26	2	0.34	4.57E-02
GO:0043200	response to amino acid	102	4	1.35	4.57E-02
GO:0051251	positive regulation of lymphocyte activa...	148	5	1.95	4.60E-02
GO:0006793	phosphorus metabolic process	2576	43	33.97	4.67E-02
GO:0008285	negative regulation of cell proliferatio...	414	10	5.46	4.69E-02
GO:0030036	actin cytoskeleton organization	358	9	4.72	4.71E-02
GO:0031098	stress-activated protein kinase signalin...	149	5	1.97	4.71E-02
GO:1901701	cellular response to oxygen-containing c...	590	13	7.78	4.74E-02
GO:0050793	regulation of developmental process	1349	25	17.79	4.75E-02
GO:0003158	endothelium development	62	3	0.82	4.83E-02
GO:0043500	muscle adaptation	62	3	0.82	4.83E-02
GO:0051928	positive regulation of calcium ion trans...	62	3	0.82	4.83E-02
GO:0006778	porphyrin-containing compound metabolic ...	27	2	0.36	4.89E-02
GO:0032410	negative regulation of transporter activ...	27	2	0.36	4.89E-02
GO:0070169	positive regulation of biomineral tissue...	27	2	0.36	4.89E-02
GO:0031529	ruffle organization	27	2	0.36	4.89E-02

GO:0071622	regulation of granulocyte chemotaxis	27	2	0.36	4.89E-02
GO:0045773	positive regulation of axon extension	27	2	0.36	4.89E-02
GO:0001782	B cell homeostasis	27	2	0.36	4.89E-02
GO:0030101	natural killer cell activation	27	2	0.36	4.89E-02
GO:0014911	positive regulation of smooth muscle cel...	27	2	0.36	4.89E-02
GO:0009967	positive regulation of signal transducti...	776	16	10.23	4.90E-02
GO:0045926	negative regulation of growth	151	5	1.99	4.94E-02
